# Clinical, humanistic and economic burden associated with recurrence among patients with early-stage cancers: a systematic literature review

**DOI:** 10.3389/fonc.2025.1575813

**Published:** 2025-05-26

**Authors:** Raquel Aguiar-Ibáñez, Yves P.V. Mbous, Sugandh Sharma, Evanka Chawla, Deepshikha Pawar

**Affiliations:** ^1^ Merck Canada Inc., Kirkland, QC, Canada; ^2^ Merck & Co., Inc., Rahway, NJ, United States; ^3^ Parexel International, Chandigarh, India; ^4^ Parexel International, Mohali, India

**Keywords:** cancer recurrence, survival, clinical burden, humanistic burden, economic burden, cost of disease, quality of life

## Abstract

**Introduction:**

While cancer recurrences have been reported as negatively affecting patients’ prognosis and imposing an economic burden to healthcare systems, there is no comprehensive summary of evidence on how frequently recurrence occurs across early-stage cancers. The goal of this study was to assess recurrence rates and their resulting clinical, humanistic and economic burden in patients with early-stage cancers.

**Methods:**

A narrative, systematic literature review was conducted including non-interventional studies evaluating adult patients diagnosed with cancer at early-stages (including: melanoma, triple negative breast cancer, non-small cell lung cancer, renal-cell carcinoma, gastric cancer, head and neck cancer, and bladder cancer). Selected studies were identified through electronic database searches, conference proceedings, and grey literature sources. Outcomes of interest included recurrence rates, post-recurrence survival, and the humanistic and economic burden associated with recurrences.

**Results:**

Among 82 studies included, 75 reported recurrence rates, eight investigated post-recurrence survival, five evaluated post-recurrence patient-reported outcomes, and seven examined the post-recurrence economic burden. Across most cancer types, recurrences occurred frequently, with later stages at diagnosis being associated with higher recurrence rates and shorter time to recurrence compared to earlier stages at diagnosis. Cancer recurrence was associated with lower survival, reduced health-related quality of life (HRQoL), worsening cancer-related symptoms and higher healthcare resource utilization. These outcomes were also more pronounced among patients diagnosed at later stages. Among cancer survivors, most patients experienced moderate fear of cancer recurrence (FCR). Patients with clinically relevant FCR had worse cancer-related symptoms and reduced HRQoL compared to those without. Direct costs in recurrent patients (predominantly in the form of inpatient and outpatient costs) were the main drivers for the total healthcare costs incurred, irrespective of the cancer types and stages.

**Conclusion:**

This study highlights the high recurrence rates experienced by patients diagnosed with early-stage cancer, particularly if diagnosed at later stages (Stage III), and their clinical, humanistic and economic impact. Cancer stage at the time of diagnosis is a key indicator of recurrence risk and post-recurrence outcomes, emphasizing the importance of earlier diagnosis and the need for therapies that prevent recurrences to better mitigate their clinical, humanistic and economic burden.

## 1 Introduction

Cancer recurrence remains one of the pressing concerns of patients and their caregivers even after receiving treatments with curative intent. With the majority of patients experiencing at least some form of moderate to severe fear of recurrence, recurrence represents a serious unmet need among patients with early-stage cancer (1). The intent of therapy for patients with solid tumors in early stage is typically curative, as the cancer remains localized and surgical resection and/or definitive radiation are used to remove all cancer cells. However, even after treatment with standard treatment options, many patients diagnosed with early-stage cancers are still at high risk of recurrence (1, 2). There is an unmet need for effective and well-tolerated treatments, given that recurrence rates with current treatment options remain high (3, 4).

Patients who recur following complete resection are at an increased risk of death compared to patients without recurrence (5). Among patients with renal cell carcinoma (RCC), those with recurrence have been shown to have an increased risk of death compared to patients without recurrence. Poorer disease-free survival (DFS) rates have also been observed among patients with high risk RCC compared to those with intermediate-high risk (6). In non-small cell lung cancer (NSCLC), patients who experience recurrence have a significantly lower chance of survival compared to those who do not experience recurrence at different landmark time points post-surgery (7, 8).

In addition to the impact on patient survival, recurrence and fear of recurrence lead to substantial humanistic burden among patients diagnosed at early stages (1, 9). The heterogeneity and aggressiveness of early-stage cancers increase the fear of recurrence among patients, which has been associated with increased functional limitation, reduced quality of life, and high rates of mental health conditions across several types of cancers (10, 11). Previous studies have also estimated a substantial economic burden on patients with early-stage cancer experiencing recurrence, due to the additional costs and healthcare resource utilization (HCRU) incurred compared to patients who remain in remission (1, 12–14). Upon recurrence, early-stage patients and their caregivers experienced higher work impairment, lower work productivity, reduced employment, higher HCRU, and higher costs. These impacts are more pronounced for patients experiencing distant recurrence compared with locoregional recurrences. However, the existing evidence on recurrence and its impact, wherever found, has been typically limited to investigations of recurrence patterns and/or one type of relevant post-recurrence outcome (clinical, humanistic, or economic) within a specific tumor type (4, 12, 13, 15–22).

According to the American Cancer Society, statistics on the risk of cancer recurrence are limited. This is caused, in part, because clinicians are not required to report cancer recurrences (23). Using data from five tumor types (thyroid, colon, melanoma, pancreas, and breast cancers) and more than 700,000 patients, a study uncovered that recurrence information was incomplete in 18.2% to 34.8% of cases. In particular, hospitals provide incomplete recurrence information in 56.7% to 66.7% of cases (24). This translates into a partial and incomplete understanding of the burden of cancer recurrence. A recent systemic literature review (SLR) reported solely on patterns of recurrence among patients with early-stage (stage I-III) cutaneous melanoma (25), whereas another added the clinical dimension, albeit in a different cancer type, through the examination of recurrence rates and post-recurrence survival in head and neck cancers (HNC) (26). On a humanistic level, several SLRs have limited their appraisal of recurrence outcomes to the emotional and psychological distress, the treatment type and prognosis, the quality of life (QoL), and a host of severe physical symptoms that arise among young and adult cancer survivors (27–29). With respect to the economic consequences of recurrence, published studies have focused on a single tumor, as evidenced by studies evaluating financial burden (health resource utilization, healthcare costs) between recurrent and non-recurrent patients diagnosed with triple negative breast cancer (TNBC), NSCLC, and RCC (4, 13, 30, 31).

Thus, recurrence rates have not been comprehensively assessed across different tumor types despite their negative impact on patient’s prognosis and their economic burden on health systems. Additionally, many aspects remain under-evaluated when examining the differences in reported recurrence rates in early-cancer stages, including the tumor type, the time to recurrence and the stage at initial diagnosis. Given the lack of exhaustive evidence from a pan-tumor perspective on the burden of recurrence, this study aims to fills this gap by assessing recurrence rates and post-recurrence outcomes in seven tumors of high interest.

The objective of our study was to conduct a narrative SLR to systematically summarize published estimates of recurrence rates and related outcomes reporting on the clinical, humanistic and economic burden of recurrences across patients diagnosed with different types of early-stage cancers, in particular melanoma, TNBC, NSCLC, RCC, gastric cancer, HNC and bladder cancer (**Table 1** in Section 2.2).

**Table 1:** Summary of the inclusion/exclusion criteria for the burden of recurrence review.

PICOTS	Inclusion Criteria
**Population(s)**	• Adult (≥18 years) patients of any gender or race, diagnosed at early-stage (i.e., in general, stages I to III, excluding unresectable stage III, unless otherwise specified below) with any of the following tumor types: ∘ Melanoma ∘ TNBC ∘ NSCLC (Squamous and non-squamous), including: • Surgically treated early-stage patients with stage IB-IIIA NSCLC (AJCC V7), corresponding to stage II, IIIA, and Resectable IIIB (T3-4N2) NSCLC (AJCC V8) • Surgically ineligible early-stage patients receiving radiation therapy in Stage I or II (T1 to limited T3, N0, M0) NSCLC (AJCC V8) ∘ RCC (Stage I-IV; T1-T4, N0/M0) ∘ Gastric cancer: including patients diagnosed with early gastric cancer/gastroesophageal junction, stages I-IVA ∘ Head and neck cancer (stages I-IVA, including locally advanced) ∘ Bladder cancer (Muscle invasive and non-muscle invasive)
**Interventions**	No restriction
**Comparisons**	No restriction
**Outcomes**	Key outcomes are listed below (not exhaustive; indicative list): • Clinical review ∘ Recurrence rates, annualized hazards of recurrence at different timepoints, incidence or prevalence of recurrences, stage and/or type of recurrence, factors associated with recurrence ∘ Different recurrence outcomes, (i.e., RFS, DFS, and EFS (median, mean, hazard rates, and rates of survival at different timepoints)) ∘ Definitions of recurrences and outcomes (i.e., RFS, DFS, and EFS) ∘ Among patients who experience a recurrence, the impact of recurrence rates on survival outcomes or outcomes after recurrences, compared with patients who did not experience recurrence ∘ Different survival outcomes, i.e., OS and PFS (median, mean, hazard rates, and rates of survival at different timepoints) • Economic review ∘ Direct medical costs for outpatients, including visit fees, tests, treatments, consultations or follow-ups with a specialist, routine exams/check-ups, medical screenings, healthcare professionals [primary care physicians and specialists (physician assistants, physical therapists, etc.)] ∘ Direct medical costs for inpatients, including hospitalization costs, serious illnesses or medical issues that require substantial monitoring, management of complications, treatment administration, length of stay, medications, hospital room, laboratory tests, pharmacy usage, healthcare professionals [specialists and larger group of caretakers (nurse practitioners, surgeons, technicians, social-care workers, physical therapists)], monitoring equipment/supplies, administrative/operational charges ∘ Direct non-medical costs, including travel, accommodation, and meals ∘ Indirect costs for patients and caregivers (e.g., productivity loss costs including lost wages, absenteeism, early retirement, etc.) • Humanistic review (impact on patients and families/caregivers) ∘ Mental, emotional, social well-being (either qualitative or quantitative), FCR, FCR prevalence, FCR severity, factors associated with higher FCR (e.g., demographic, clinical, psychological), qualitative lived experience of FCR ∘ General patient-reported outcomes (e.g., 0–100 scales/questionnaires) • Pre-treatment/baseline scores, post-treatment scores at different time points, change from baseline (absolute differences), percentage change from baseline (fraction differences), p-value, 95% CI, proportion of patients reporting an improvement or deterioration
**Time**	May 2012 to May 2022
**Study design**	Study designs included were: • Clinical review ∘ Retrospective observational studies ∘ Prospective observational studies ∘ Case-control/cohort studies ∘ Cross-sectional studies ∘ Database/registry-based studies ∘ Non-randomized studies ∘ Relevant reviews for bibliographic searching/validation of results • Economic review ∘ Cost studies/surveys/analyses ∘ Cost/economic burden of illness ∘ Database studies collecting cost data (e.g., claims databases) ∘ Studies providing resource use data ∘ Relevant reviews for bibliographic searching/validation of results • Humanistic review ∘ Retrospective observational studies ∘ Prospective observational studies ∘ Case-control/cohort studies ∘ Cross-sectional studies ∘ Database/registry-based studies ∘ Non-randomized studies ∘ Questionnaires/surveys/qualitative research ∘ Relevant reviews for bibliographic searching/validation of results
Country	Global
Other (Language)	Studies with full texts published in English language only will be included

Section 2 of this manuscript reports the methods followed to implement this systematic review. Additionally, section 3 reports on the study findings, including: how included studies were selected (section 3.1); the characteristics of the included studies and the corresponding patient populations (sections 3.2 and 3.3, respectively); the recurrence rates reported across studies for each cancer type, along with time to recurrence, recurrence sites, and post-recurrence survival (section 3.4); the humanistic and economic burden associated with recurrence (sections 3.5 and 3.6 respectively); and the assessment of the risk of bias across studies (section 3.7). The discussion and conclusions are respectively reported in sections 4 and 5.

## 2 Methods

### 2.1 Data sources and search strategy

An SLR was conducted following the methodology listed by the Cochrane Handbook and the Centre for Reviews and Dissemination (32, 33). The reporting of this SLR followed the guidelines of the Preferred Reporting Items for Systematic Reviews and Meta-Analysis (PRISMA) (34). MEDLINE^®^, Embase^®^, Cochrane Database of Systematic Reviews and the Cochrane Controlled Register of Trials and PubMed (solely to capture in-process citations) were screened to retrieve peer-reviewed material published between 2012 and 2022. Grey literature sources included the Science Citation Index (SCI), Turning Research into Practice (TRIP) Medical database and Open Grey. Additional searches included conference repositories such as the American Society of Clinical Oncology (ASCO), the European Society for Medical Oncology (ESMO) and the International Society for Pharmacoeconomics and Outcomes Research (ISPOR) (2018–2022, inclusive). Reference harvesting from past SLRs was also conducted. The search strategy is provided in the **Supplementary Appendix S1**.

### 2.2 Eligibility criteria for study selection

Eligible studies featured adult patients (≥18 years) diagnosed at early-stage (stages I-III, excluding unresectable stage III disease) with either melanoma, TNBC, and NSCLC (both squamous and non-squamous), RCC (Stages I-IV; T1-T4, N0/M0), HNC (stages I-IVA, including locally advanced), bladder cancer (muscle invasive and non-muscle invasive), gastric cancer (including stages I-IVA gastric or gastroesophageal junction cancer) or any combination thereof, provided that the results were provided per individual tumor type. The NSCLC population of interest included surgically treated early-stage patients with stage IB-IIIA NSCLC (American Joint Committee on Cancer [AJCC] V7), corresponding to stage II, IIIA, and resectable IIIB (T3, N2) NSCLC (AJCC V8); as well as surgically ineligible early-stage patients receiving radiation therapy in Stage I or II (T1 to limited T3, N0, M0) NSCLC (AJCC V8). A detailed summary of all the inclusion criteria based on Population, Interventions, Comparisons, Outcomes, Time and Study design criteria (PICOTS) is provided in **Table 1**.

Studies reporting on the following outcomes were to be included: 1) Recurrence rates (e.g., overall recurrence rate and recurrence rate by type, such as localized vs. distant recurrence) and the clinical impact of recurrence, including OS in routine clinical practice. 2) The humanistic impact of recurrence, including impacts on the QoL of both patients and caregivers, such as the fear of recurrence, challenges and distress after recurrence, and additional measures using both generic and disease-specific instruments. 3) The economic burden of recurrences, including HCRU and cost implications related to patients experiencing recurrences (both relating to direct and indirect costs, and work productivity loss from multiple perspectives, including those of patients, health systems, insurers, and caregivers).

### 2.3 Study search, selection and abstraction

**Figure 1:**
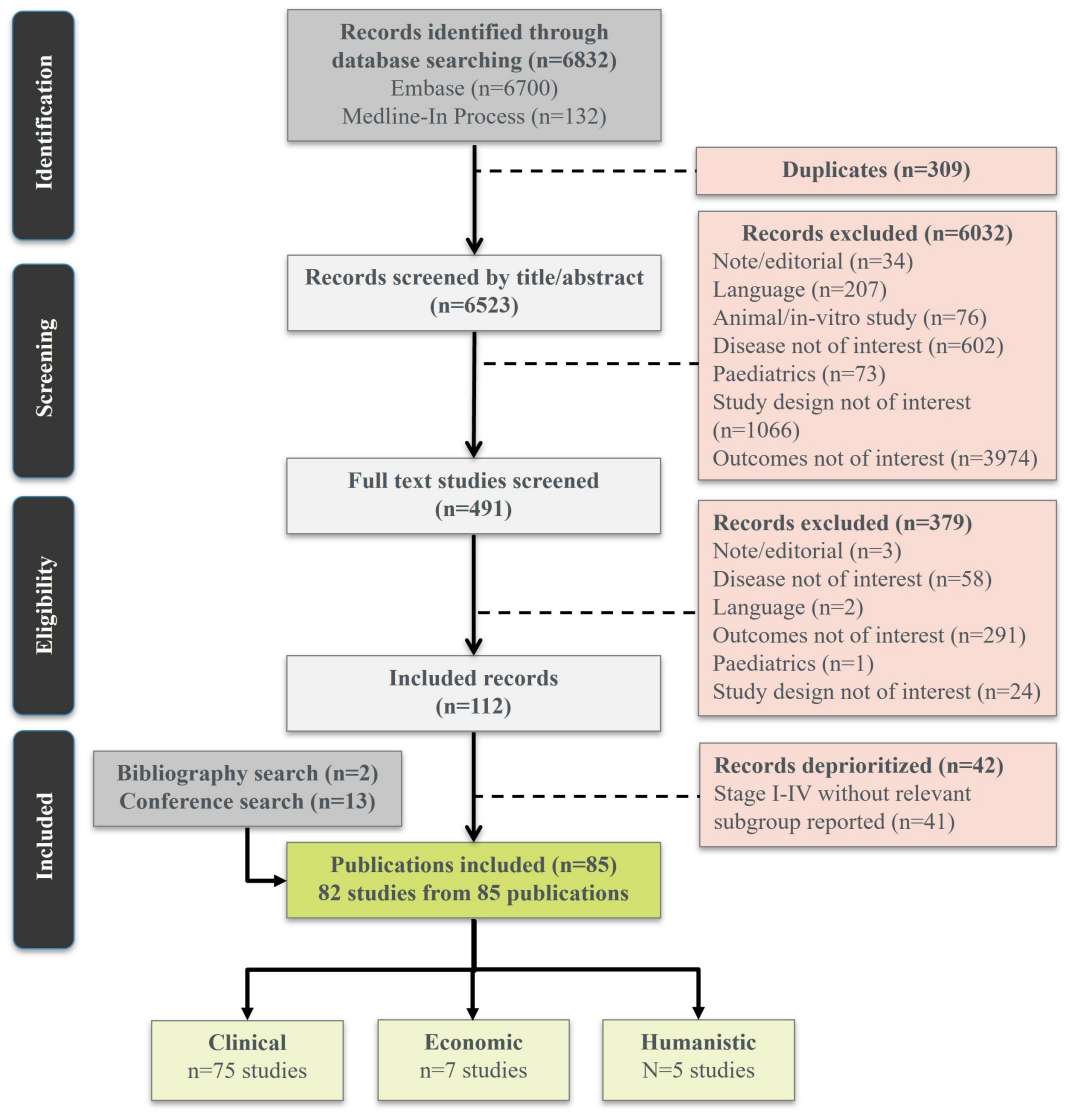
PRISMA flow diagram. PRISMA: preferred reporting items for systematic reviews and meta-analyses.

To identify relevant studies for inclusion, screening of titles and abstracts, followed by reviews of full-text articles, were undertaken by two independent reviewers. A third independent reviewer was involved to resolve any discrepancies. For final studies that were included, data were extracted into a pre-defined data extraction form, ensuring uniformity across studies. Data extraction was conducted by two reviewers and validated by an independent reviewer. If the exact time at measurement for recurrence rates was not mentioned, the studies were excluded from the final pool, albeit still portrayed in the PRISMA flow diagram at earlier steps (**Figure 1**).

Although various outcomes were abstracted from the final pool of studies (**Supplementary Appendix 2**), for the purpose of homogeneity, only the outcome metrics that were similar across the final pool of studies were reported. Clinical outcomes included recurrence rates, post recurrence OS and time to recurrence (**Supplementary Appendix S3**); humanistic outcomes included patient reported outcomes (PROs), such as fear of cancer recurrence (FCR) and cancer-related symptoms (**Supplementary Appendix S4**); HCRU and healthcare costs were selected as the relevant economic outcome metrics (**Supplementary Appendix S5**). Additional outcome metrics collected have been reported in **Supplementary Appendix S6**. Definitions of recurrence provided in different studies (if available) were provided in **Supplementary Appendix S2**.

### 2.4 Risk of bias, strength and certainty of evidence

The quality assessment of the included observational studies, in particular, cohort and cross-sectional studies, was conducted using the Newcastle-Ottawa scale (NOS). The Larg-Moss quality assessment scale was used for the assessment of the cost of illness studies (35, 36).

### 2.5 Data analysis

Outcomes were reported according to their type (clinical, humanistic, or economic), and were further stratified according to tumor type and the stage of patients at diagnosis.

Recurrence rates were summarized either ‘at discrete timepoints’ or ‘at study follow-up’. Recurrence rates ‘at discrete timepoints’ referred to rates reported at assigned discrete timepoints, e.g., at 1-, 2-, 3-, 5-, 10-years. Recurrence rates ‘at study follow-up’ described the proportions of patients who recurred during the follow-up of the studies.

To assess the distribution of recurrences by type, the denominator used was based on the total number of patients who experienced a recurrence. Consistently throughout the reporting of findings, the denominator for assessing the percentage of site of recurrence was kept as the total number of patients with a specific type of recurrence.

To maintain homogeneity of the results, only the OS is reported here as the metric for post-recurrence survival. Other measures can be found in the **Supplementary Appendix S6**. Where possible, the 3-year and 5-year recurrence rates or OS rates were extracted from the Kaplan-Meier curves provided in the studies using a Web plot digitizer. For prognostic or risk factors in scenarios where studies reported both multi and univariate analyses, only the multivariate results have been retained.

## 3 Results

### 3.1 Study selection

In total 82 studies were included, with the majority of evidence reporting recurrence-related clinical outcomes (n=75), and relatively sparse evidence identified for humanistic burden (n=5) and economic outcomes (n=7) (**Figure 1**). A summary of included studies is provided in the **Supplementary Appendix S7**.

### 3.2 Study characteristics

Of the 82 included studies, the vast majority investigated melanoma (n=25) (37–61), TNBC (n=19) (53, 62–79) and bladder cancer (n=17) (80–96). The remaining investigated HNC (n=9) (97–105), NSCLC (n=7) (106–112), RCC (n=3) (113–115) and gastric cancer (n=2) (116, 117). These studies were conducted primarily in Europe (n=26: Albania n =1 (116), Croatia n=1 (64), Denmark n=2 (118), France n=2 (85, 92), Italy n=2 (54, 101), Ireland n=1 (41), Germany n=2 (59, 74), Netherlands n=4 (42, 53, 56, 119), Norway n=1 (102), Spain n=3 (43, 49, 61), Sweden n=4 (57, 89, 114, 115), UK n=3 (83, 96, 99)), the US (n=24) (46, 47, 52, 55, 65, 67, 70, 72, 73, 77, 78, 84, 93–95, 104, 109, 112, 120), APAC (n=19: Australia n=3 (39, 48, 60), India n=1 (63), Israel n=1 (79), Japan n=1 (40), New Zealand n=1 (75), Republic of Korea n=4 (88, 100, 105, 117), Singapore n=2 (86, 87), Taiwan n=2 (37, 103), Thailand n=1 (81), Turkey n=3 (44, 50, 68)), South America (n=2: Brazil n=1 (62), Mexico n=1 (66)), Africa (n=1: Tunisia n=1 (107)), multinational (n=3 (76, 108, 110)), and in unspecified locations (n=9 (45, 51, 69, 71, 80, 97, 106, 111, 113)).The study design of choice was retrospective observational design (n=69) (37, 39–47, 49–57, 59, 62–65, 67–74, 76–79, 81, 83–89, 92, 94–97, 99, 100, 102, 103, 105–113, 116, 118), with a minority adopting prospective longitudinal observational designs (n=10) (48, 66, 75, 80, 101, 114, 115, 120), cross-sectional (n=4) (38, 93, 117, 119), case control designs (n=2) (60, 104), and a cost of illness study (n=1) (61). The entire data collection spanned a period between 1953 and 2020 (39, 41, 51, 80, 88), with median follow-up ranging from 11.4 months to 150.1 months (76, 106) (mean range: 23.4–87 months (83, 85)).

For full details of study characteristics refer to **Supplementary Appendix S7**.

### 3.3 Patient population characteristics

Of the studies reporting age, the mean age of patients ranged from 33-88.34 years (63, 85), whereas the median age ranged from 36-74.8 years (67, 77). The sample size ranged from 20-30,834 (37, 118). Male proportion per sample size varied between 14.7-95.5% of the total sample size of the included studies (103, 107), except for TNBC, where women formed the totality of the study samples (53, 62–79) and one study of patients with melanoma which was conducted in women only (38). Race and ethnicity were reported across 18 studies (38, 39, 46, 49, 55, 61, 62, 67, 71, 73, 77, 87, 90, 93–95, 108, 109, 120). Caucasian typically made up the majority of the sample (range: 29.0-100.0% (38, 108)), followed by African Americans (range: 0.7-47.1%) (71, 93). In a single study, Chinese patients made up 77.0% of the sample size. Overall, 19 studies reported stage distribution by tumor, nodes, and metastases (TNM) stage (37, 46, 53, 64, 71, 75, 81, 85, 89, 90, 95, 96, 100, 102, 107, 108, 114, 115), and 17 studies reported stage distribution according to AJCC criteria (39, 40, 43, 44, 46–49, 52, 54, 56, 57, 59, 60, 63, 69, 73, 98, 102, 108). Three studies reported staging according to International Classification of Diseases (ICD) 9/10 (67, 70, 72) and one study each reported staging according to The World Health Organization (WHO) grade (89), Mayo staging (88) and Furhman grade (115). Study numbers are not mutually exclusive as some studies use multiple different staging systems.

For full details of patient characteristics refer to **Supplementary Appendix S7**.

### 3.4 Recurrence rates and post-recurrence overall survival

A total of 75 studies provided evidence for recurrence rates and post-recurrence OS. Availability of data for recurrence rates according to stage of disease at initial diagnosis varied by tumor type, as well as across patient subgroups within each type of cancer. In the identified studies, recurrence was mostly measured from date/time of primary diagnosis or from initial treatment including surgery to the recurrence of the tumor. A comprehensive overview of all study findings can be found in the **Supplementary Appendix S3**.

#### 3.4.1 Recurrence rates

Recurrence rates were reported across each of the seven different tumor types, with most evidence identified for melanoma (18 studies), followed by bladder cancer (14 studies), TNBC (14 studies), HNC (five studies), NSCLC (four studies), RCC (three studies), and gastric cancer (one study).

**Figure 2:**
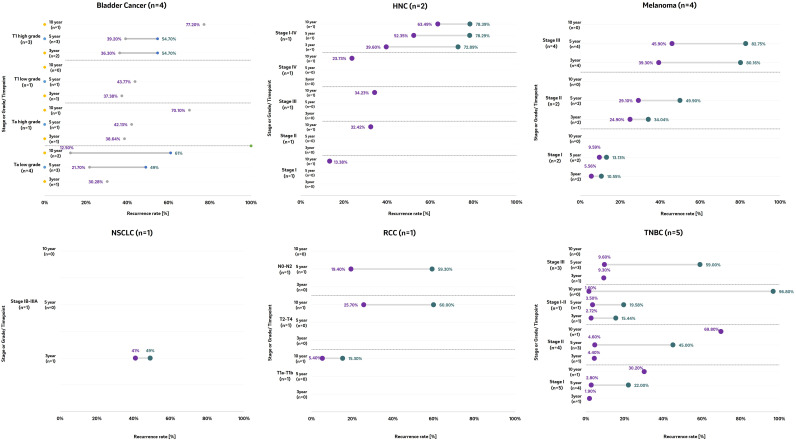
Recurrence rates by tumor type at discrete timepoints according to stage an/or grade at diagnosis. Note: The horizontal axis represents the proportion of patients experiencing recurrences for included studies, while the vertical axis reflects the time period for which recurrence rates were reported across the included studies, with the number of studies reporting recurrence rates for that specific time point in brackets. The references of the studies represented in the figures are as follows: Bladder cancer (86, 90–92); Head and neck cancer (100); Melanoma (40, 46, 56, 57); Non-small cell lung cancer (110); Renal cell carcinoma (114); Triple negative breast cancer (20, 69, 73, 77, 78). HNC, Head and Neck cancer; NSCLC, Non-Smal Cell Lung Cancer; RCC, Renal Cell Carcinoma; TNBC, Triple Negative Breast Cancer; T, tumor size; N0, No cancer near the lymph-nodes; NX, Cancer in nearby lymph nodes cannot be measured; TX, Main tumor cannot be measured.3.4.2 Time to recurrence.

Across tumor types and discrete timepoints, recurrence was common across early stage cancer types, as demonstrated by high recurrence rates (**Figure 2**).

In patients diagnosed with low grade Ta non-muscle invasive bladder cancer (NMIBC), the identified 3-year recurrence rate was 30.28% (94), and ranged from 21.7% to 49% at 5 years post diagnosis (87, 91, 94). In patients diagnosed with high grade T1 NMIBC, the 3-year recurrence rate ranged from 36.28% to 54.71%, and the 5-year recurrence rate ranged between 39.2% and 54.68% (90, 94).

In patients diagnosed with HNC, the 10-year recurrence rate increased with increasing stage as shown in patients diagnosed with stage I (13.38%), stage II (32.42%) and stage III (34.23%) (102).

In patients diagnosed with melanoma, when stratified according to disease stage, the 3-year recurrence rates ranged from 5.6% to 10.6% in patients diagnosed at stage I (46, 57). In patients diagnosed with stage II melanoma, the 3-year recurrence rate ranged from 39.3% to 80.2% (56, 57).

At 3-year post diagnosis, the recurrence rate in patients diagnosed with stage I TNBC was 1.90%, whereas at 5-year post-diagnosis, the recurrence rate in the same patient population ranged from 2.8% to 22%. In patients diagnosed with stage III TNBC, the 5-year recurrence rate ranged from 9.6% to 59% (63, 73, 78). At 10-years post-diagnosis, recurrence rates ranged from 30.2% in stage I TNBC patients to 69.8% in stage II patients (69).

In patients diagnosed with RCC, the 10-year recurrence rate in patients diagnosed with tumor size T1a-T1b ranged from 5.40% to 15.3%, and in patients diagnosed with tumor sizes T2-T4, it ranged from 25.7% to 60% (114). In patients diagnosed with nodal status N0-N2, the 5-year recurrence rate ranged from 19.4% to 59.3% (114).

When assessed according to recurrence rates at study follow-up, the same trend was observed. The recurrence rate increased over time and with more advanced disease stage and/or tumor size.

In patients diagnosed with primary oral squamous cell carcinoma and followed for at least 5 years, recurrence rates were higher among patients diagnosed at stage III (25%) compared to those diagnosed at stage II (22%) and stages 0-1 (13%) (99). At a mean follow-up of 34.7 months, the recurrence rate increased with tumor size in patients diagnosed with HNC. Recurrence rate at follow-up increased from 13.8% in patients diagnosed at T1 HNC, to 24.7% in T2 HNC and 28.8% in T4 HNC (105).

In patients diagnosed with cutaneous malignant melanoma, overall recurrence rate was 24.1% at a median follow-up of 4.43 years (0-9.8). Stage-wise, the recurrence rates in patients diagnosed at stages I, II, and III respectively were 14.6%, 39.9%, and 82.4% at a median follow-up of 53.2 months (57).

At a mean follow up of 25.3 months, the recurrence rate in patients diagnosed with NSCLC mostly followed an increasing trend from stage IA at diagnosis to stage IIIB at diagnosis (IA: 10%, IB: 0%, IIA: 50%, IIB: 38.9%, IIIA: 52% and IIIB: 100%) (107).

In patients diagnosed with TNBC, recurrence rates were 16.4% in those diagnosed at stage I, 26.8% in those diagnosed at stage II, and 53.7% in those diagnosed at stage III at a mean follow-up time of 68.2 months (71). In patients diagnosed with early stage (stage I-II) TNBC, recurrence rates were at least twice as high in patients diagnosed at stage II compared to stage I (69.8% vs 30.2%) (69).

An exception to the trend was observed among patients diagnosed with stage IIB/C melanoma, who had higher recurrence rates (range: 94.7% - 100%) compared to patients diagnosed with stage IIIA melanoma (83.3%). Full details of recurrence rates at study follow-up can be found in **Supplementary Appendix S3**.

Recurrence rates at study follow-up also increased over time within the same cancer stage. At a median of 37.7 months, the recurrence rate for patients diagnosed with T1 high grade NMIBC patients was 34.5% (standard deviation; SD: 45.4) (94). At a median follow-up of 40.1 months, the recurrence rate increased to 55.7% in a similar population (T1 high grade NMIBC) (81).

#### 3.4.2 Time to recurrence

**Figure 3:**
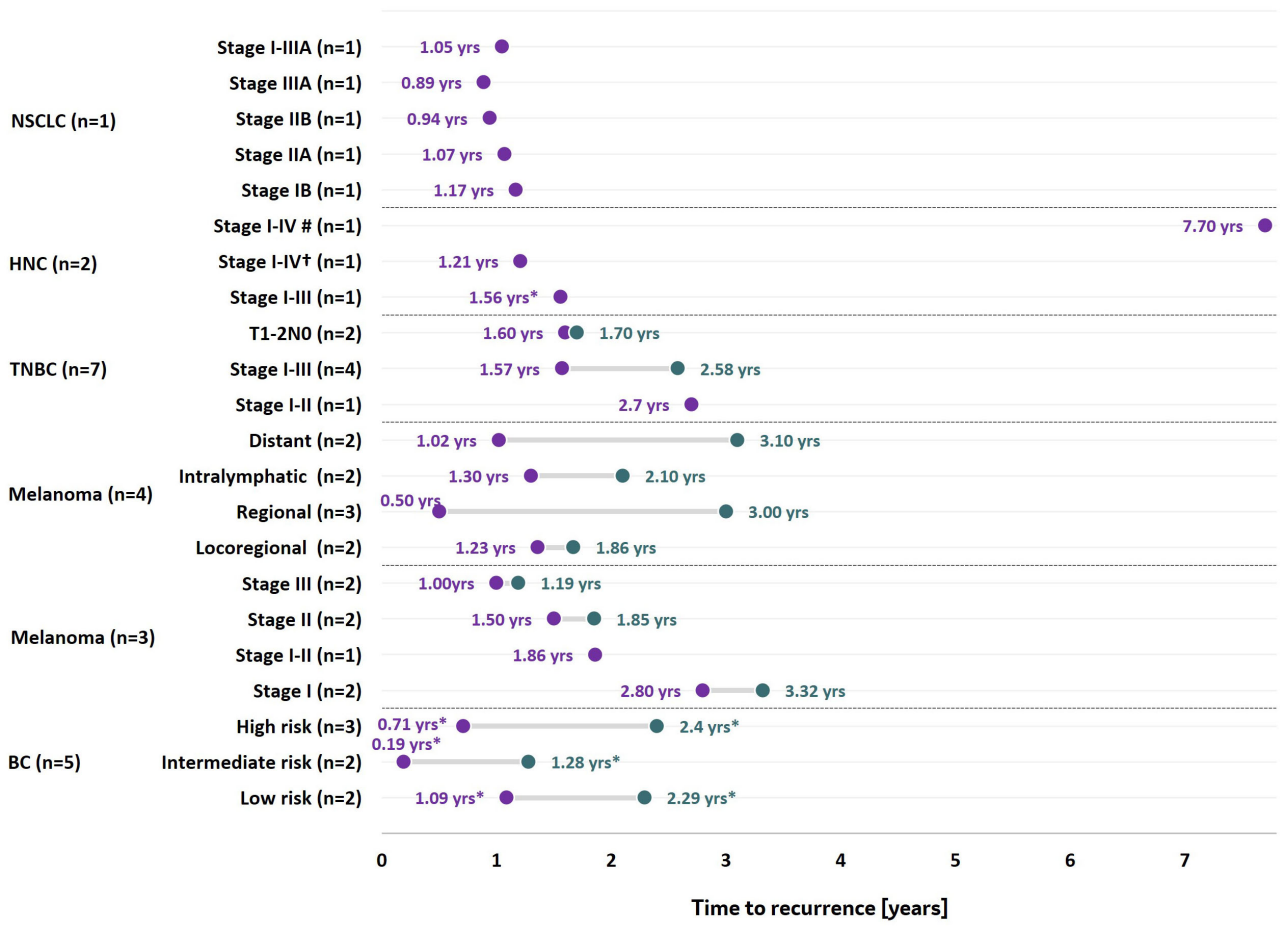
Time to recurrence by tumor type. *Refers to mean time to recurrence, while the remaining data points refer to median times. # refers to late recurrence; that is recurrence that occurred 5 years post-treatment/diagnosis; †: refers to early recurrence; that is recurrence that occurred less than 5 years post-treatment/diagnosis. The horizontal axis represents the median or mean time to recurrence as reported in included studies, while the vertical axis reflects the tumor stage, tumor risk (low, intermediate or high risk of recurrence) or the type of recurrence reported (e.g. locoregional, regional or distant), with the number of studies reporting time to recurrence for that specific group identified in brackets. BC, Bladder cancer; HNC, Head and neck cancer; NSCLC, Non-small cell lung cancer; TNBC, triple-negative breast cancer (48–50, 56, 81, 83, 89–91, 100, 104, 109, 114).

Across studies, the time to recurrence (TTR) varied across tumor types although, in general, it tended to decrease with more advanced stages of disease at the time of diagnosis and/or treatment (**Figure 3**). In patients diagnosed with NSCLC, recurrence occurred approximately one year after treatment or diagnosis, as the median TTR varied from 1.17 years in patients diagnosed with stage IB NSCLC to 0.89 years in patients diagnosed with stage IIIA NSCLC (109).

In patients diagnosed with HNC, recurrence occurred at a mean TTR of 1.60 years post-treatment or diagnosis in patients diagnosed with stages I-III (104). Late recurrence, defined as recurrences occurring at least 5 years post-treatment or diagnosis in patients diagnosed with HNC, had a median TTR of 7.70 years (100). Early recurrence, defined as recurrences occurring less than 5 years post-treatment or diagnosis in patients diagnosed with HNC, had a median TTR of 1.21 years (100).

In the remaining cancers, the range of TTR was wider. In patients diagnosed with stage I melanoma, the TTR ranged from 2.80 years (56) to 3.32 years (49), whereas in patients diagnosed with stage III melanoma, it ranged from 1.00 year (56) to 1.19 years (49).

In patients diagnosed with NMIBC, the TTR ranged from 1.07 years (83) in patients with low risk of developing muscle invasive disease to 2.29 years among patients with low risk of developing muscle-invasive disease and G1pTa stage (91, 121). However, it ranged from 0.19 years in more advance disease (low risk, G2pTa stage) to 1.28 years in those diagnosed with intermediate-risk disease (83, 121).

#### 3.4.3 Site of recurrence

In total, 22 studies (bladder cancer n=2, HNC n=2, melanoma n=6, NSCLC n=1, RCC n=3, TNBC n=8) reported on the site of cancer recurrence, with varying levels of detail provided regarding time of occurrence, type and location of recurrence.

The most common sites of recurrence across tumor types were the lung, the brain, and the bone. The proportion of lung metastases ranged from 5.9% in patients diagnosed with high-grade urothelial carcinoma (at 24 months post-diagnosis) (82) to 88.9% in patients diagnosed with salivary gland HNC patients (at 92.5 months post diagnosis) (100).

Eight studies reported the brain as one of the most common sites of metastasis in patients diagnosed with melanoma, NSCLC and TNBC (37, 47, 48, 54, 68, 74, 76, 110).

Another study conducted in patients diagnosed with TNBC showed a high incidence of developing brain metastases as the first site of recurrence over time and with advanced stages. Significant differences in the incidence of brain metastases were reported for patients with stages I, II and III, with the 2-year cumulative incidence being 0.8%, 3.1%, and 8% (p<0.0001), and the 5-year cumulative incidence reported as 2.8%, 4.6%, and 9.6% (p<0.0001), respectively (77).

#### 3.4.4 Type of recurrence

A total of 35 studies reported on the type of recurrence (melanoma n=16 (37, 39, 40, 43–50, 54–56, 58, 59), TNBC n=11 (53, 63, 67–69, 71, 73–76), HNC n=3 (98, 100, 102), NSCLC n=3 (109, 110, 112), RCC n=2 (113, 114). The proportion of recurrences that were reported as local ranged from 17.3-47.8%, whereas the proportion of recurrences that were distant ranged from 52.2%-67.7% (**Supplementary Appendix S3**) (37, 71, 100, 113). An exception to this trend was observed in patients diagnosed with melanoma at stage I-III who experienced recurrences, among whom a higher proportion experienced locoregional recurrences (range: 43.6%-59.6%) compared to those who experienced distant recurrences (range: 29%-43.1%) (49).

Of the 5 studies reporting the type of recurrence according to stage of disease at diagnosis, the disease stage of the recurrence increased with more advanced disease stage at diagnosis (46, 49, 50, 56, 114). The proportion of recurrences that were local ranged from 2.4% in patients diagnosed with TNBC at stage T1-2N0 to 75.0% in patients diagnosed with oral tongue squamous cell carcinoma at stages T1N0-T3N0 (53, 98). The proportion of recurrences that were regional ranged from 1.0% in patients diagnosed with stage T1-2N0 TNBC to 60.0% in patients that were diagnosed with oral tongue squamous cell carcinoma at stage T1N0-T3N0 (53, 98). The proportion of recurrences that were distant ranged from 2.0% in patients diagnosed with melanoma in stages I/II (who experienced recurrences at least 20 years post-diagnosis) to 67.7% in patients diagnosed with salivary gland cancer (stage I-IV without distant metastasis) (54, 100). This trend was further supported by a study in patients diagnosed with RCC reporting the type of recurrence according to the size and extent of the primary tumor at the time of diagnosis. The proportion of patients experiencing distant or local recurrence was 5.4% in patients diagnosed with T1a RCC, 15.3% in patients diagnosed with T1b RCC, 25.7% in patients diagnosed with at T2 RCC, 42.1% in patients diagnosed with T3 RCC, and 60.0% in patients diagnosed with T4 RCC (114).

The proportion of recurrences that were locoregional ranged from 3.5% in patients diagnosed with laryngeal carcinoma (T1-T4, N0-N2+, stage I-IV non-metastatic) to 70.3% in patients diagnosed with melanoma at stages IA-IIC (59, 102).

Further details related to the type of recurrence experienced by patients as reported by the included studies can be found in **Supplementary Appendix S3**.

#### 3.4.5 Post-recurrence overall survival

A total of eight studies reported post-recurrence OS outcomes (bladder n=1, HNC n=1, melanoma n= 4, TNBC n=2), with higher survival rates observed in patients who recurred at local stages compared to those that recurred at distant stages, and in patients who did not experience disease progression.

Longer OS was observed among recurrent patients diagnosed with earlier stage of disease compared to those diagnosed at a later stage. The median OS (mOS) post-recurrence was 1.9 years (95%CI: 0.8-3.2 years) for patients diagnosed with melanoma at stage IB, 1.5 years (95% CI: 1.1-2.1 years) for those at stage II, and 1.1 years (95%CI: 0.6-2.2 years), for those at stage III (43).

A longer post-recurrence OS was observed in patients with melanoma and TNBC who experienced locoregional recurrence compared to those who experienced distant metastases (p<0.05). The post-recurrence mOS in patients diagnosed with melanoma was 3.9 years (95% CI: 2.5-NR years), 2.8 years (95% CI: 1.9-4.6 years), and 0.5 years (95% CI: 0.3-0.6 years) among those who developed regional lymph node metastases, intralymphatic metastases and distant metastases, respectively (56).

In patients diagnosed with TNBC who experienced locoregional recurrences, the OS rates at 1-, 2-, 3- and 5-year post-recurrence were 80.3%, 73.1%, 65.8% and 65.9%, respectively. By comparison, the OS rates among patients with TNBC who recurred with distant metastasis were 47.8%, 28.2%, 23.4% and 20.3% at 1-, 2-, 3- and 5-year post-recurrence, respectively (71).

An additional indicator for improved OS included the absence of disease progression. Among patients with disease progression, the mOS was 18.2 months, while in patients without disease progression, the mOS was 45.2 months (81). In patients diagnosed with HNC, the mOS was lower in patients who recurred ≤5 years after initial treatment (mOS: 19.7 months) compared to patients who recurred >5 years after initial treatment (mOS: 79.7 months) (100). No data on OS were available for gastric cancer, RCC or NSCLC.

### 3.5 Humanistic burden

Five studies reported humanistic outcomes in patients diagnosed with melanoma (n=3), gastric cancer (n=1), and bladder cancer (n=2), reported in **Supplementary Appendix S4**.

Among the included studies, cancer survivors diagnosed with gastric cancer and melanoma experienced moderate to high level of fear of cancer recurrence (FCR), which resulted in lower health-related quality of life (HRQoL), higher levels of anxiety and depression, and worsening cancer-related symptoms compared to those with low or no FCR (38, 93, 117, 119, 120). Of the included studies reporting FCR, three studies did not explicitly specify the recurrence status of patients (38, 117, 119), and one study stated that, while patients did not have recurrence at the time of the survey, recurrence status during the follow-up period was not recorded (120).

In patients diagnosed with bladder cancer, 61.8% reported moderate FCR, while 11.2% reported high FCR, as measured by the Fear of Cancer Recurrence Inventory-Severity subscale (FCRI-s) and the Cancer Problems in Living Scales (CIPLS) (120).

In patients diagnosed with melanoma, 62.6% experienced moderate FCR, 33.1% low FCR, and 4.3% high FCR (120). Patients diagnosed with localized melanoma showed moderate FCR (mean: 3.16 out of 4, SE: 0.13) on the Concerns About Recurrence Scale (CARS) (38).

Patients diagnosed with early-stage melanoma scored a mean of 2.69/5 (SD: 0.96) on the Impact of Cancer scale-Health Worries subscale (IOC-HWS) (119).

Patients diagnosed with gastric cancer who showed clinical FCR had significantly higher FCRI subscale scores than those with non-clinical FCR (78.1 vs 45.5, SD: 20 vs 17.2; p< 0.001) across triggers such as severity, psychological distress, functioning impairments, insight, and reassurance subscales (117).

Higher FCRI scores were associated with lower HRQoL and higher levels of anxiety and depression, as measured by the EuroQol-Visual Analogue Scale (EQ-VAS), global health status/quality of life scale of the European Organization for the Research and Treatment of Cancer Quality of Life Questionnaire (EORTC QLQ–C30) and Hospital Anxiety and the Depression Scale (HADS) (38, 117, 120).

Patients diagnosed with melanoma reported increasing FCR with more advanced disease (119, 120). In patients diagnosed with bladder cancer, QoL scores were lower with recurrent NMIBC (72.8), with MIBC (72.8), and with metastatic MIBC (64.3) compared to patients with non-recurrent NMIBC (75.1) (95).

The majority of the cancer-related symptoms such as fatigue, nausea/vomiting and pain were worse in recurrent vs non-recurrent cancer, as measured by the EORTC QLQ-C30. Mean fatigue scores were reported as 23.9 vs. 20.3 in patients with recurrent NMIBC vs. patients with non-recurrent NMIBC, while nausea and vomiting scores were 3.5 vs. 2.8, respectively and pain scores were 24.1 vs. 21.2, respectively, (with EORTC symptom scale scores ranging between 0 and 100, and with higher symptom scores indicating a higher level of symptomatology) (93).

### 3.6 Economic burden

Of the seven included studies that reported outcomes relating to the economic burden of recurrences, five reported on HCRU associated with cancer recurrence across HNC (n=1), melanoma (n=2) and TNBC (n=2), and all seven included studies reported healthcare associated costs (42, 52, 58, 67, 70, 104) (**Table 1**). Patients with recurrence had higher economic burden compared to non-recurrent patients. Among patients who experienced recurrence, those who were diagnosed at later stages incurred higher treatment costs and increased HCRU compared to those diagnosed at earlier stages. Healthcare cost patterns varied significantly among different types of recurrence, with the economic burden increasing with the severity of recurrence type. The main cost drivers differed based on the stage of cancer at the time of diagnosis and the type of recurrence experienced, with hospital visits, admissions, surgeries, and pharmacotherapy being prominent contributors to healthcare costs and resource utilization (42, 58, 104).

**Figure 4:**
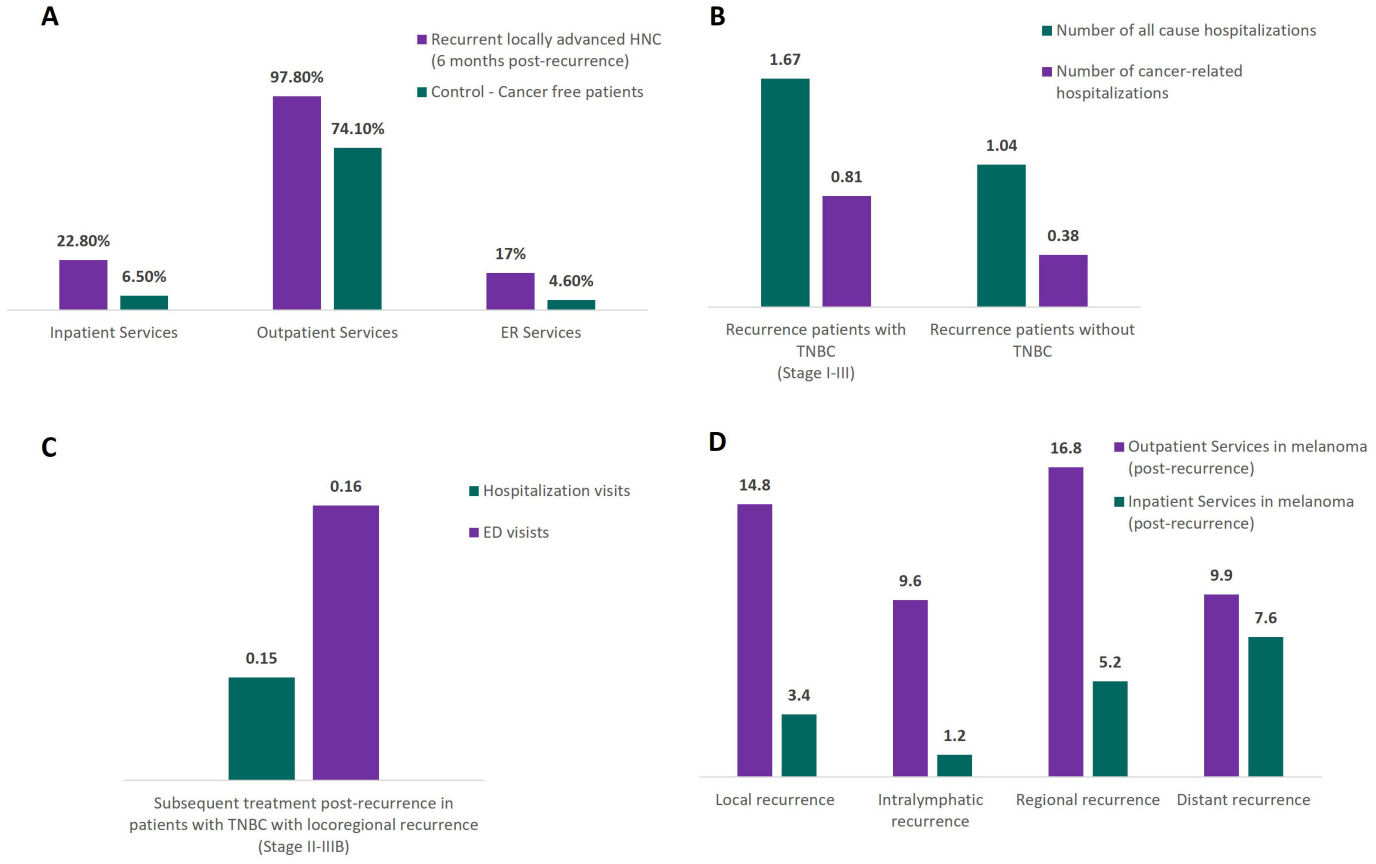
Distribution of HCRU in patients with and without recurrence, and different recurrence types, across tumor types (HNC, melanoma, TNBC). **(A)** HCRU for 6-months post-recurrence in patients with HNC (104); **(B)** HCRU, number of hospitalizations in patients with and without TNBC (70); **(C)** HCRU, subsequent treatment post-recurrence in patients with TNBC (72); **(D)** HCRU, subsequent treatment post-recurrence in patients with melanoma (42). ED, emergency department; HCRU, Healthcare resource utilization; HNC, Head and neck cancer; TNBC, Triple negative breast cancer.

Recurrence led to higher overall HCRU and variations in healthcare service use for patients with locoregional and distant recurrence (**Figure 4**). Patients with recurrent HNC had greater use of outpatient services (97.8% vs. 74.1%; p-value not reported), and hospitalization, including inpatient visits (22.8% vs. 6.5%; p<0.0001), compared to a cancer-free control group (**Figure 4A**) (104). Similarly, patients diagnosed with TNBC who experienced recurrence had a higher number of all-cause hospitalizations (mean: 1.67 vs. 1.04, p=0.019) and of cancer-related hospitalizations (0.81 vs. 0.38, p=0.007), compared to patients who were not diagnosed with TNBC (70) (**Figure 4B**). Patients diagnosed with locoregional recurrence of TNBC had a median of 0.15 hospitalizations per month and a median of 0.16 emergency department (ED) visits per month (**Figure 4C**) (72). Overall, patients diagnosed with melanoma who recurred had lower outpatient clinic visits (mean 14 visits vs. 16 visits, with and without disease recurrence, respectively) and longer hospital stays (6 days vs. 4 days, with and without recurrence, respectively) during the initial treatment episode. Average utilization of inpatient services also increased with higher disease stage, with patients requiring 3.4 days if recurrence was local, and 7.6 days if patients recurred at distant stages (**Figure 4D**) (42).

There were distinct patterns in healthcare cost burden observed between patients experiencing recurrence when compared to recurrence-free patients, or between patients experiencing different types of recurrence. In patients diagnosed with localized melanoma, hospital visits were the main cost driver (48% and 52% of the costs in patients with and without disease recurrence having at least one hospital visit, respectively), followed by hospital admissions (24% and 21%, respectively) and surgery (15% and 12%, respectively). In patients diagnosed with regionally advanced melanoma, hospital admissions related to surgical procedures were the main cost driver of the mean episode costs in patients with and without recurrence (39% and 33%, respectively of the mean episode costs), followed by surgery (27% and 33%, respectively) and hospital visits (19% and 24%, respectively) (42).

In patients diagnosed with operable non-metastatic melanoma and experiencing a locoregional recurrence episode (with a mean duration of the episode of 2.4 months), 88.4% of the average all-cause total healthcare costs were attributed to medical costs (total inpatient, emergency department and outpatient costs, equal to US$2,340). Of the average costs associated with medical services for melanoma (including treatments and disease monitoring costs that were melanoma-specific), 47.5% (US$730) was attributable to surgery (58).

Similarly, for patients diagnosed with melanoma who experienced distant recurrence, medical costs contributed to 89.3% (US$11,549) of the average mean total all-cause healthcare cost per patient per month (PPPM).The mean episode duration for these patients was 13.4 months. Considering the average melanoma-specific cost, 66.2% (US$5,195) of costs were attributable to pharmacotherapy (58). Skin surgery and monitoring (laboratory) were the major contributors to melanoma-specific HCRU, used by 84.6% and 40.0% of the patients diagnosed with melanoma who locoregional recurrence, respectively (58). In patients diagnosed with melanoma who experienced distant recurrence, the most important components of melanoma-specific HCRU were monitoring and pharmacological treatments, with 93.1% and 73.5% of the patients using these resources, respectively (58).

Studies reporting on total healthcare costs in patients with recurrence found that total costs were substantially higher in patients with disease recurrence compared to those without, and in advanced disease stage when compared to earlier stages after patients experienced recurrence (42, 58, 72, 104). The highest difference was observed in patients diagnosed with melanoma in the US, with costs 12 times higher for patients who experienced recurrence compared to those who did not (US$1,076 PPPM vs. US$12,940 PPPM) (58). Similarly, patients with recurrent TNBC had higher all-cause inpatient cost compared to those without recurrence ($28,105 vs. $13,505) (70), while the total direct cost for patients with recurrent HNC over a 6-month period was nearly nine times higher than that of the controls ($25,837 vs $2,752) (104).

When stratified according to the type of recurrence, the economic burden increased with the severity of the recurrence among studies including patients with melanoma. The lowest costs were incurred by patients experiencing local recurrence (US$1,537 PPPM), and the highest by patients experiencing distant metastases (US$7,845 PPPM) (58). In another study, the mean total all cause healthcare cost for recurrence episodes of melanoma was higher for distant recurrences (€10,393 or $12,986) compared to locoregional recurrences (€4,414 or $5,515) (42). A summary of the total costs by tumor type is provided in **Table 2**. Full data from the economic burden review is provided in the **Supplementary Appendix S5**.

**Table 2:** Total healthcare costs among patients with HNC, melanoma and TNBC.

Study name	Patient population	Stage at diagnosis	Sample size (N)	Currency	Description of cost	Cost item	Cost	p-value
HNC								
Kim 2012 (104)	Recurrent locally advanced head and neck cancer	Locally advanced	324	USD	Direct healthcare costs per patient for 6-months	Total cost	25837	<0.0001
	Control (Cancer free patients)						2752	
	Adjusted difference between head and neck cancer and controls						Difference: 21141	
Melanoma								
Leeneman 2021 (42)	Localized- patients without recurrence	Localized melanoma	54	Euro	Healthcare costs for full disease course	Total cost	Mean (SD): 3032 (2338) Median (IQR): 2579 (IQR: (251–11 509)	NR
	Localized- patients with recurrence		144	Euro	Healthcare costs for full disease course		Mean (SD): 20,007 (20284) Median (IQR): 14,887 (IQR: 685-130,901)	NR
	Regionally advanced- Patients without recurrence	Regionally advanced melanoma	51	Euro	Healthcare costs for full disease course	Total cost	Mean (SD): 5951(4575) Median (IQR): 4484(1270–25 400)	NR
	Regionally advanced- Patients with recurrence		47	Euro	Healthcare costs for full disease course		Mean (SD): 19519(12947) Median (IQR): 17530(IQR: 2081-52709)	NR
	Localized- patients without recurrence	Localized melanoma	54	Euro	Healthcare costs of the initial treatment episode	Total cost	Mean (SD): 3032(2338) Median (IQR): 2579 (IQR: 251–11 509)	NR
	Localized- patients with recurrence		144	Euro	Healthcare costs of the initial treatment episode		Mean (SD): 3015(2078) Median (IQR): 2392 (IQR: 342-12432)	NR
	Regionally advanced melanoma - patients without recurrence	Regionally advanced melanoma	50	Euro	Healthcare costs of the initial treatment episode	Total cost	Mean (SD): 5951 (4575) Median (IQR): 4484 (1270–25 400)	NR
	Regionally advanced- Patients with recurrence		47	Euro			Mean (SD): 7648 (6975) Median (IQR): 6175(924-40569)	NR
	Patients with recurrence	Local recurrence	13	Euro	Healthcare costs of subsequent treatment episodes	Total cost	Mean (SD): 4414 (3868) Median (IQR): 3241(747-11794)	NR
	Patients with recurrence	Intratympanic metastases	40	Euro			Mean (SD): 4604 (11181) Median (IQR): 1696(189-86785)	NR
	Patients with recurrence	Regional lymph node metastasis	73	Euro			Mean (SD): 8129 (5926) Median (IQR): 7027(95-40520)	NR
	Patients with recurrence	Distant metastases	128	Euro			Mean (SD): 10393 (14345) Median (IQR): 6133(95-105483)	NR
Jang 2020 (52)	Patients with recurrence	Stage IIB/C	NR	USD	Healthcare cost at 1 year	Total cost	Mean (SD): 31870 (49,147)	NR
	Patients with recurrence	Stage IIIA	NR	USD			Mean (SD): 29224 (48,837)	NR
Serra-Arbeola 2017 (61)	Patients with recurrence	Phase 3: Nodal Recurrence;	NR	Euro	Costs of Diagnostic and Therapeutic Processes	Ultrasound-guided fine-needle aspiration	Cost per Unit: 495.47;	NR
			NR	Euro		Histology	Cost per Unit: 179.12;	NR
			NR	Euro		Anesthesia consultation	Cost per Unit: 188.70;	NR
			NR	Euro		Preanesthetic assessment (x-ray, ECG, blood tests)	Cost per Unit: 326.66;	NR
			NR	Euro		Intervention (lymph node dissection)	Cost per Unit: 4902.57;	NR
			NR	Euro		Histology	Cost per Unit: 298.54;	NR
			NR	Euro		Assessment of tumor extension	Cost per Unit: 661.11;	NR
			NR	Euro		2nd visit (and subsequent)	Cost per Unit: 156.50;	NR
			NR	Euro		Oncology consultation	Cost per Unit: 255.04;	NR
			NR	Euro		Follow-up oncology consultation	Cost per Unit: 156.50;	NR
			NR	Euro		Total cost, without adjuvant treatment	Total Cost: 7620.21	NR
			NR	Euro		INF treatment4 (5 d × 4 wk)	Cost per Unit: 4809.67;	NR
			NR	Euro		Oncology day hospital (5 d × 4 wk)	Cost per Unit: 8432.33;	NR
			NR	Euro		Total cost, with INF adjuvant treatment	Cost per Unit: €20 862.21;	NR
			NR	Euro		Radiotherapy	Cost per Unit: 3872.52;	NR
			NR	Euro		Total cost with radiotherapy	Total Cost: 11492.73	NR
	Patients with recurrence	Distant metastasis detected on recurrence	NR	Euro	Biopsy results	SLNB- positive (total cost)	Total Cost: 108587	NR
			NR	Euro		SLNB- negative (total cost)	Total Cost: 91741	NR
Tarhini 2018 (58)	Non-Metastatic melanoma patients	NR	6400	USD	All cause healthcare cost	Total healthcare cost	Mean (SD): 1225 (2636)	NR
	Locoregional recurrence cohort	NR	950	USD	All cause healthcare cost	Total healthcare cost	Mean (SD): 1989 (4202)	NR
	Matched recurrence free cohorts	NR	950	USD	All cause healthcare cost	Total healthcare cost	Mean (SD): 1720 (2779)	NR
	Distant recurrence cohort	NR	87	USD	All cause healthcare cost	Total healthcare cost	Mean (SD): 2210 (2740)	NR
	Matched recurrence free cohorts	NR	87	USD	All cause healthcare cost	Total healthcare cost	Mean (SD): 2357 (6439)	NR
	During episodes of locoregional recurrence (N=1116 patients) (N=1524 episodes)	NR	1116	USD	All-cause healthcare costs, $US 2017 (PPPM)	Total healthcare cost	Mean (SD): 2645 (6638)	NR
	During episodes of distant recurrence	NR	102	USD	All-cause healthcare costs, $US 2017 (PPPM)	Total healthcare cost	Mean (SD): 12940 (16341)	NR
	During the recurrence-free period	NR	6400	USD	All-cause healthcare costs, $US 2017 (PPPM)	Total healthcare cost	Mean (SD): 1076 (3661)	NR
	Locoregional recurrence cohort	NR	950	USD	Healthcare cost	Total healthcare cost	Mean (SD): 1647 (NR)	<0.001 vs. MRF cohort
	Matched recurrence free cohorts	NR	950	USD	Healthcare cost	Total healthcare cost	Mean (SD): 805 (NR)	NR
	Distant recurrence cohort	NR	87	USD	Healthcare cost	Total healthcare cost	Mean (SD): 15937 (NR)	<0.001 vs. MRF cohort
	Matched recurrence free cohorts	NR	87	USD	Healthcare cost	Total healthcare cost	Mean (SD): 984 (NR)	NR
TNBC								
Haiderali 2021 (72)	Locoregional recurrence in early-stage TNBC patients	Stage II–IIIB	21	USD	Mean monthly cost per patient	NR	Mean (SD): 7820 (14914)	NR
Başer 2012 (70)	Recurrence patients with TNBC	Stage I-III	87	USD	Adjusted Annual Post-Index Total and Health Plan-paid Costs for Recurrent Patients with TNBC (all cause inpatient costs)	All cause impatient cost	Total cost: 28105	NR
	Recurrence patients without TNBC		202		Total cost: 13505		NR

Euro, Euros; HNC, Head and neck cancer; IQR, Interquartile range; MRF, Matched recurrence free; NR, Not reported; SD, Standard deviation; TNBC, Triple negative breast cancer; USD, United States dollar.

### 3.7 Risk of bias

According to the NOS, of the 77 included cohort studies, the majority of studies (n=69/77) had a total score of 4-6 (out of a maximum score of nine; and 7–9 for high quality, 4–6 for medium quality, 0–3 for low quality studies), meaning they were of medium quality and had a high risk of bias. Only one study (49) had a total score of seven, implying that it was of high quality, and no studies had scores greater than 7. Most studies (71/77) lacked comparability between cohorts in design or analysis. The majority of studies did not adequately describe follow-up of cohorts; 64 studies (n=61/77) did not include any statements regarding follow-up of cohorts and one study (n=1/77) did not provide a description of patients that were lost to follow up (**Supplementary Appendix S8**).

Furthermore, four cross-sectional studies were assessed using the NOS. Three out of four studies were of medium quality with high risk of bias (scores of 4-6). One study reported high quality and low risk of bias (scores of 7-9). The main weaknesses of the studies were related to the selection of the control group and the comparability of the subjects in different outcome groups based on the study design or analysis, where none of the two studies scored any points. (**Supplementary Appendix S8**). A single cost of illness study was identified in this SLR, with quality assessment conducted according to Larg-Moss quality assessment (**Supplementary Appendix S8**). According to the assessment checklist, plausibility of occurrence of counterfactual population was the study weakness, while strengths included epidemiologic approach, quantification methods, cost components, and uncertainty analysis, with detailed documentation and discussion of limitations.

## 4 Discussion

This SLR aimed to assess recurrence rates and their clinical, humanistic, and economic burden in early-stage cancers. To the best of our knowledge, this study represents the first attempt to systematically review this type of evidence across a broad range of early-stage tumor types, including bladder, gastric, HNC, melanoma, NSCLC, RCC, and TNBC. Across most cancer types, recurrences occurred frequently, with later stages at diagnosis being associated with higher recurrence rates and shorter time to recurrence compared to earlier stage at diagnosis. The findings of this study also describe the multifaceted impact of recurrence among patients with cancer. From a clinical perspective, experiencing a recurrence was found to have a considerable impact on survival outcomes, regardless of tumor type. Patients diagnosed at earlier stages generally experienced lower recurrence rates and improved post-recurrence OS, while patients diagnosed and treated at later stages had an increased risk of recurrence and reduced OS expectations. Furthermore, the type of recurrence was also identified as an important contributing factor for post-recurrence OS, which was generally improved among patients with locoregional recurrences compared to those experiencing distant metastases. Cancer recurrence was also linked to higher costs and increased HCRU when compared to non-recurrent patients, with hospital admissions representing the main cost driver. In addition, due to the prognostic implications associated with disease recurrence, the humanistic burden among patients was largely characterized by fear of cancer recurrence, which in turn was associated with lower quality of life, and higher levels of both anxiety and depression.

### 4.1 Recurrence rates and post-recurrence survival

Evidence from the SLR revealed that even after treatment with standard treatment options, many patients diagnosed with early-stage cancers are still at high risk of recurrence, resulting in shorter survival times. Additionally, cancer staging at initial diagnosis was found to have a profound impact on the rate of disease recurrence, time to recurrence and post-recurrence OS. For most cancer types, including bladder cancer, NSCLC, HNC, RCC, melanoma and TNBC, diagnosis at a later, more advanced stage was associated with higher recurrence rates when compared with early diagnosis (46, 57, 63, 71, 73, 77, 86, 91, 94, 96, 102, 107, 114). This finding was consistent with the existing literature not part of this SLR and that has focused on specific cancer types. For example, in previous studies evaluating patients diagnosed with melanoma, the overall recurrence rate ranged from 9% to 79% (69, 122). Previous studies also found an increase in recurrence rate with more advanced stages of disease; that is in stage I, the recurrence rate was 19%, whereas in stages IIIB/IIIC the recurrence rate was 79% (122). Recurrence also increased over time as previous reports showed that patients diagnosed with stage I and II melanoma were estimated to have 1-year and 10-year recurrence rates of 9% and 23%, respectively (25, 123–125). The recurrence rates identified in this review were comparable (1-year recurrence rate in stage IIA: 12.50%, stage IIC: 21.8% and stage IIIA: 28.2%; 10-year recurrence rate for stages IA-IIC: 10.8% (52, 59)). More advanced melanoma stage at diagnosis was consistently related to higher recurrence rates, aligned to the increased risk of recurrence at five years reported by a large retrospective study conducted at Memorial Sloan Kettering Cancer Center (stage IIA: 21.6%, stage IIB: 35.1%, stage IIC: 45.3%) and another retrospective analysis (stage IIIA: 48%, stage IIIB: 71%, stage IIIC: 85%) (25, 52, 59, 124, 126). In our review, identified studies reported notably higher 5-year recurrence rates ranging from 6.40-13.13% in stage I (46, 56, 57), 13.6-100% in stage II (46, 47, 56, 57), and 44.30-100% in stage III (40, 46, 56, 57).

Higher recurrence rates were identified in our SLR among patients with more advanced HNC (with the 10-year recurrence rate increasing by stage, from 13.38 for stage I, 32.42% for stage II and 34.23% for stage III, and then decreasing to 23.73% for stage IV (102)). This finding was generally consistent with those reported in previous studies. For example, the prevalence for locoregional recurrences in African patients with HNC has previously reported as 19.0% for stages I and II, and 81.0% for stages III and IV HNC (26).

Median time to recurrence was influenced by the type of recurrences experienced by patients, as highlighted by the longer time associated with the development of distant metastases, compared to locoregional and regional recurrences, among patients with early-stage melanoma (56). Results from a previous literature review in early-stage cutaneous melanoma were consistent with these findings, as recurrences were reported to be more common in patients with more advanced disease, and that, for the majority of cases, metastases occurred within two to three years of diagnosis in early-stage melanoma (stages I-III) (25). Moreover, the median or mean time to recurrence was generally prolonged among patients initially diagnosed at early stages, compared to those diagnosed at later stages, although identified data were limited to patients with bladder cancer, melanoma, TNBC, HNC and NSCLC (49, 56, 69, 75, 91, 100, 104, 109). The above findings emphasize the clinical burden that a diagnosis of early-stage cancer has on patients, due to the high likelihood of developing recurrences, particularly in the first few years after diagnosis.

Our study also showed the impact of recurrence on OS. Although the specific patient populations varied across studies, our findings concluded that a recurrence among patients with more advanced melanoma was related to lower post-recurrence OS, with a median survival of 3.9 years among patients who developed regional lymph node metastases, 2.8 years for intralymphatic metastases, and 0.5 years for distant metastases, respectively (56). Median survival reported in other studies has also differed depending on melanoma stage at diagnosis. For example, among patients with melanoma and recurrences in the local and in-transit (LCIT)-lymph node, median survival has been reported as 22 months, compared to seven months for patients with systemic recurrence (25, 122).

### 4.2 Humanistic burden of cancer recurrence

FCR among patients and their caregivers is one of the most common unmet needs reported by patients with cancer, associated with impaired QoL and psychosocial adjustment, elevated emotional distress, and a range of physical symptoms (1, 9). While FCR, reduced QoL and uncertainty of health status following cancer treatment are hallmarks of the humanistic burden among oncology patients, limited evidence was identified on the humanistic burden associated with cancer recurrence, highlighting the need for further studies in this area. Among the identified evidence, patients with recurrent cancer were generally reported to experience lower QoL, including higher levels of anxiety and depression, and worsening cancer-related symptoms such as fatigue, nausea/vomiting and pain, compared to those without recurrence (38, 93, 117, 119, 120).

As highlighted in a previous study, which reported that FCR was associated with worse psychological distress among minority or indigenous populations when compared with dominant populations, the humanistic burden may be exacerbated by a diverse range of patient-related factors (1, 9, 127, 128). This study indicated the need for future research to be cognizant of such disparities, and for FCR to be considered through a population-specific lens (129).

### 4.3 Economic burden of cancer recurrence

From an economic perspective, the findings of our SLR indicated that patients with recurrence of melanoma, TNBC, and HNC incurred substantial cost burden. Across the studies, the direct costs in recurrent patients (predominantly in the form of inpatient and outpatient costs) were the main drivers for the total healthcare costs incurred, irrespective of the cancer types and stages.

This was aligned to findings from a previous study where cancer management (primarily driven by outpatient, ED visits and hospitalization) was reported to represent the majority of total direct costs among patients with TNBC (14). Our findings also demonstrate that overall costs were driven by hospitalizations, pharmacotherapy and surgical interventions (64).

When considering the impact of disease stage, advanced cancer stages at the time of diagnosis were associated with higher overall costs and direct medical costs in recurrent patients. The primary driver for this additional cost burden was increased need for medical management for recurrent disease, in the form of higher inpatient visits, ER visits, supportive care visits, and other medical services. Stratification according to the type of recurrence suggested that among patients with melanoma, those who experienced distant recurrence incurred higher costs (€10,393) when compared to those with locoregional recurrence (€4,414) (42). This trend was also reported previously, where monthly healthcare costs for patients with TNBC and metastatic recurrence and patients with TNBC and locoregional recurrence were $8,575 and $3,609 higher, respectively, when compared with patients without recurrence (130).

While evidence was sparse and there was lack of evidence identified for other tumor types, three studies investigating the economic burden of recurrence among patients with early-stage NSCLC were published in 2023, which revealed extensive recurrence associated costs across Italy, Spain and the US (131–133). Similar studies across other geographies and other tumor types should be encouraged.

### 4.4 Implications to clinical practice and research

By providing a broad perspective across tumors diagnosed in early stages, this study offers a consolidated overview of the current understanding with regards to recurrence patterns across several different cancer types. The findings from this study emphasize the extent of clinical burden that a diagnosis of early-stage cancer has on patients due to the high likelihood of developing recurrences, particularly in the first few years after diagnosis. Additionally, as the stage of disease severity at the time of initial diagnosis serves as a pivotal predictor for both recurrence probability and long-term survival, these findings highlight the importance of timely diagnosis in cancer management. In turn, this may help healthcare providers and decision makers to make informed decisions on the levels of unmet need among patients with early stage cancers, particularly when considering new treatment strategies. Furthermore, it was identified that lung, brain tissue and bones were the predominant sites for cancer recurrence across multiple tumor types, which underscores the importance of prioritizing these areas in surveillance protocols when recurrence is suspected (37, 47, 48, 54, 68, 74, 76, 82, 100, 110, 114). Adopting a proactive approach may help improve detection of recurrence among patients, implement specialized interventions and more effective allocation of resources, ultimately improving patient outcomes.

This narrative SLR provides critical insights into the impact of cancer recurrence and highlights the need for tailored cancer screening policies, targeted research into predictive models and novel surveillance techniques, and potential economic benefits through improved early detection. The findings underscore the importance of multidisciplinary collaboration in follow-up care, the adoption of personalized medicine approaches, and the integration of emerging technologies in surveillance protocols. Furthermore, this study emphasizes the global health implications of cancer recurrence and the necessity for holistic survivorship care addressing both physical and psychological well-being. By identifying key knowledge gaps, particularly in certain cancer types and patient-reported outcomes, this review lays the foundation for future research, including comparative effectiveness studies of follow-up and treatment strategies. These insights can inform policy decisions, optimize resource allocation, and ultimately enhance patient outcomes across diverse healthcare systems.

Notably, this review identified significant gaps in the current evidence for outcomes related to cancer recurrence, across various tumor types and stages. Crucially, there was limited data for gastric cancer, NSCLC or RCC which hinders the evaluation of recurrence patterns for these cancers. Furthermore, evidence on post-recurrence OS was not identified for RCC and NSCLC, while for gastric cancer only one study (reporting DFS rather than OS) was identified. In addition to clinical outcomes, PRO outcomes represent a key aspect of patient care and should factor into decisions regarding treatment and disease management. However, very limited evidence on the humanistic burden associated with recurrence was identified. Future research to assess the humanistic burden associated with recurrence is required to better inform interventions to reduce anxiety, improve coping mechanisms, patient well-being and potentially improve long-term outcomes. Efforts to measure the impact of FCR across tumor types beyond those identified in this review should also be encouraged, along with additional studies to evaluate potential interventions to reduce FCR, and determine feasibility of their implementation across various healthcare systems (9). Early recognition, support, and validation of feelings associated with FCR or recurrence, and appropriate referrals to psychosocial oncology, could be beneficial for many patients, especially considering the impact on their QoL (1).

### 4.5 Strengths and limitations

To our knowledge, this review was the first to broadly evaluate recurrence rates using real world data, as well as their associated clinical, humanistic and economic outcomes across patients diagnosed with a broad range of early-stage tumor types. In order to assess the robustness of this review, a critical appraisal of the included studies was conducted using the NOS and the adapted checklist by Larg and Moss. The majority of the identified studies were assessed to be of moderate quality, which indicated potentially high risk of bias and that some caution should be exercised when considering their findings for clinical application or for decision-making purposes. Potential biases and methodological inconsistencies across included studies may limit reliability and generalizability of the findings. In terms of the economic impact of recurrences, this could lead to over- or underestimation of the financial burden associated with cancer recurrence. In addition, variations in study methodologies across studies may hinder cross-study comparisons limiting the extent of conclusions regarding the true economic and humanistic burden of cancer recurrence. Moreover, the included studies were heterogeneous in terms of variations in patient characteristics, treatment modalities, and outcome definitions, and this may influence the consistency and comparability of the findings. This heterogeneity was anticipated and was the reason why the study was originally designed as a narrative systematic review. One significant challenge related to the variability in the definitions of recurrence as reported across different studies. For example, while some studies differentiated between local and distant recurrence, others employed varying time-based categorizations, and a subset of studies failed to provide any clear definition of recurrence.

This review also highlights a substantial gap in research on the humanistic impact of cancer recurrence, with only 5 studies included that covered melanoma, gastric, and bladder cancers. This gap may limit the provision of comprehensive patient-centered care, the facilitation of shared decision-making, and the ability to tailor care to individual patient needs, potentially underestimating the full impact of recurrence on patients’ lives. Future research should prioritize longitudinal studies of fear of recurrence and quality of life across multiple cancer types to enhance patient care through a more holistic, personalized approach. A recently published study examined patients with early-stage cancer experiencing recurrence, and in caregivers of such patients, across various cancer types in the US. The findings of this study indicated that both patients and caregivers experienced decreased QoL following recurrence, particularly among those with distant/metastatic recurrence compared to locoregional recurrence (134). As part of the gaps in the existing literature identified by this study, the need for further investigation into the humanistic burden of recurrence across different patient populations was emphasized.

The available evidence on the economic burden of cancer recurrence was similarly limited, with data primarily focused on HNC, melanoma, and TNBC; therefore, restricting the generalizability of findings across cancer types. While costs from the studies were extracted according to the currencies and price years as reported in the included studies, no attempt was made to convert all costs to a common currency and price year, to prevent the introduction of distortions that could potentially misrepresent the economic reality of different settings. A more comprehensive understanding of the economic impact of cancer recurrence is necessary to inform healthcare decision-making. Future research should expand to cover additional cancer types using standardized methodologies and leveraging real-world data would enhance the accuracy of economic burden assessments. Results from a recently published study on the economic burden of recurrences for patients with early stage cancers and caregivers of such patients (including bladder cancer, gastric cancer, HNC, melanoma, NSCLC, RCC and TNBC) in the US (135) demonstrated significant negative impacts on work productivity, employment, finances, and HCRU, with variations across cancers and recurrence types. The substantial financial burden for patients, caregivers, and healthcare systems identified by this study suggests that strategies to prevent recurrences could mitigate these economic challenges. Future research should be expanded to evaluate these aspects across a wider range of cancer types and geographies.

There is a potential for publication bias, where studies with statistically significant or positive results are more likely to be published, while studies with negative results may not be published. In addition, the inclusion criteria identified English language studies only, and therefore relevant studies published in other languages may have been missed.

A further limitation relates to the fact that approximately 40% of the included studies reporting clinical outcomes utilized data collected before the year 2000. Given the significant advances in cancer treatments over the past two decades, reliance on older data may bias the reported recurrence rates and survival outcomes.

Some limitations in the interpretation of the results may be related to ecological fallacy, in particular when comparing results according to patients’ disease stage at the time of diagnosis or treatment. It needs to be emphasized that some other factors may impact the outcomes of recurrences, including the cancer type, health status, and treatment response. Thus, it will be important to interpret the population-level data with caution.

A significant limitation in interpreting the results stems from the inherent challenges in tracking disease recurrence using certain databases. The stage of the disease at diagnosis may not accurately reflect the patient’s condition at the initiation of treatment. This discrepancy arises due to the potential time lag between diagnosis and treatment commencement, during which the disease may have progressed. Consequently, this limitation introduces a degree of uncertainty in associating treatment outcomes with specific disease stages, potentially impacting the accuracy of analyses and subsequent conclusions drawn from the data.

### 4.6 Conclusion

This study highlights the high recurrence rates experienced by patients diagnosed with early-stage cancer, particularly if diagnosed at later stages, and their clinical, humanistic and economic impact. Cancer stage at the time of diagnosis was a key indicator of recurrence risk and post-recurrence outcomes, emphasizing the high unmet need among patients with early-stage cancer that experience recurrences. This highlights the importance of earlier diagnosis and the need for therapies that prevent recurrences to better mitigate their clinical, humanistic and economic burden. Recurrence patterns observed across multiple tumor types indicate that the lung, brain tissue, and bones are predominant recurrence sites, highlighting the need for targeted surveillance strategies. The study is instrumental in helping identify specific early-stage sub-populations where unmet need exists, which, in turn, can inform targeted and efficient implementation of health policies, improving the allocation of already stretched healthcare resources. This research underscores the necessity for tailored cancer screening policies, predictive models, and novel surveillance techniques. It also emphasizes the importance of multidisciplinary collaboration, personalized medicine approaches, and holistic care addressing both physical and psychological aspects of cancer survivorship. Despite the insights gained, this review also identifies key knowledge gaps, particularly in certain cancer types and patient-reported outcomes; therefore, this review sets the stage for future research directions.

Substantial heterogeneity across studies and limited data on humanistic and economic burdens for several cancer types remain significant challenges. Addressing these gaps through standardized methodologies, broader cancer type coverage, and longitudinal studies on fear of recurrence and quality of life is essential to advancing patient-centered care. While recent studies have begun to explore these areas, further comprehensive research is needed to fully understand the multifaceted impact of cancer recurrence and guide more effective clinical and policy interventions to inform patient-centered care strategies.

## Data Availability

The original contributions presented in the study are included in the article/**Supplementary Material**. Further inquiries can be directed to the corresponding author.
