# Supplementary material for: Clinical, humanistic and economic burden associated with recurrence among patients with early-stage cancers: a systematic literature review
Source: Front Oncol. 2025 May 26;15:1575813. doi: 10.3389/fonc.2025.1575813 (PMC12146636; doi:10.3389/fonc.2025.1575813)
Supplement: Supplementary file 1 [file DataSheet1.pdf]

Supplementary Material

Appendix S1. Search strategy

Table 1. Embase search results for HNC, gastric cancer, and RCC (conducted on 13th July 2022)

No.	Query	Results
**1**	'early stage' OR 'early-stage' OR 'early cancer' OR 'early diagnosis'/exp OR 'preliminary stage' OR 'first stage' OR 'primary stage' OR 'initial stage' OR 'earliest stage' OR 'earlier stage' OR 'early presentation' OR (('early' OR 'earlier' OR 'earliest' OR 'primary' OR 'initial' OR 'preliminary' OR 'first') NEAR/3 ('stage' OR 'staging' OR 'cancer' OR 'disease' OR 'detect*' OR 'diagnos*')) OR 'early':ab,ti OR 'early detection' OR 'early detection of cancer' OR 'stage 1*' OR 'stage i*' OR 'stage one' OR 'stage-1*' OR 'stage-i*' OR 'stage-one' OR 'stage 2*' OR 'stage ii*' OR 'stage two' OR 'stage-2*' OR 'stage-ii*' OR 'stage-two' OR 'stage 3*' OR 'stage iii*' OR 'stage three' OR 'stage-3*' OR 'stage-iii*' OR 'stage-three' OR 'stage 4*' OR 'stage iv*' OR 'stage four' OR 'stage-4*' OR 'stage-iv*' OR 'stage-four' OR 'stage 1-2' OR 'stage 1,2' OR 'stage i-ii' OR 'stage i,ii' OR 'stage 1-3' OR 'stage 1,3' OR 'stage i-iii' OR 'stage i,iii' OR 'stage 2-3' OR 'stage 2,3' OR 'stage ii-iii' OR 'stage ii,iii' OR 'stage 1-4' OR 'stage 1,4' OR 'stage i-iv' OR 'stage i,iv' OR 'stage 2-4' OR 'stage 2,4' OR 'stage ii-iv' OR 'stage ii,iv' OR 'stage 3-4' OR 'stage 3,4' OR 'stage iii-iv' OR 'stage iii,iv' OR ('stage' NEAR/2 ('1*' OR 'one' OR '2*' OR 'two' OR '3*' OR 'three' OR '4*' OR 'four')) OR 'non-advanced' OR 'non advanced' OR 'not advanced' OR 'non-metastatic' OR 'non metastatic' OR 'not metastatic' OR 'advanced' OR 'advanc*' OR 'locally advanced' OR ('local*' NEAR/3 'advance*')	4743402
**2**	'recurrence' OR 'recurrent disease'/syn OR 'disease recurrence' OR 'periodic disease' OR 'recurrent' OR 'recurrences' OR 'recurred' OR 'recur*' OR 'recurrent*' OR 'cancer recurrence'/syn OR 'recurrent cancer' OR 'recurrent carcinoma' OR 'recurrent tumor' OR 'recurrent tumour' OR 'tumor recurrence'/syn OR 'tumour recurrence' OR 'neoplasm recurrence' OR (('cancer' OR 'tumor' OR 'tumour' OR 'neoplasm') NEAR/3 ('return*' OR 'com* back' OR 'back' OR 'relapse*' OR 'recur*' OR 'residual' OR 'residue' OR 'reoccur*' OR 'reappear' OR 'repeat' OR 'reappearance')) OR 'relapse*'	1477303
**3**	#1 AND #2	390118
**4**	'value' NEAR/3 'money'	2935
**5**	'deductibles' AND 'coinsurance'	167
**6**	'societal cost' OR 'social cost' OR 'social care cost' OR 'out of pocket' OR 'out-of-pocket' OR 'patient cost' OR 'co-payment' OR 'co?payment' OR 'private expenditure' OR 'patient time' OR 'carer cost' OR 'carer expenditure' OR 'carer time' OR	79981

No.	Query	Results
	'caregiver cost' OR 'caregiver expenditure' OR 'caregiver time' OR 'economic burden' OR 'cost burden' OR 'resource burden' OR 'financial burden' OR 'economic consequences' OR 'cost of illness' OR 'healthcare cost' OR 'cost of disease'	
**7**	'costly' OR 'costing' OR pharmacoeconomic* OR 'pharmaco economic*' OR financ* OR 'finances' OR 'financed' OR 'cost analysis' OR 'cost assessment' OR 'cost study'	482783
**8**	('value' NEAR/2 ('money' OR 'monetary')) OR (cost* NEAR/3 (treat* OR therap*))	71356
**9**	('out' NEAR/2 'pocket') OR (patient* NEAR/2 cost*) OR copay* OR (privat* NEAR/2 expendit*) OR ((carer* OR caregiv*) NEAR/2 (cost* OR expendit* OR time))	50571
**10**	(econ* NEAR/2 ('burden' OR 'disease' OR 'assessment')) OR (cost* NEAR/2 (illness* OR health* OR 'burden' OR 'disease' OR 'assessment' OR estimate OR variable OR saving* OR sharing OR direct OR indirect OR employer OR drug OR hospital))	422489
**11**	'economics' OR 'cost' OR 'health care cost' OR 'drug cost' OR 'hospital cost' OR 'socioeconomics' OR 'health economics' OR 'health economics'/exp OR 'fee' OR 'charge*' OR 'budget' OR 'hospital finance' OR 'health care financing' OR 'low cost' OR 'high cost' OR (health*care NEXT/1 cost*) OR ('health care' NEXT/1 cost*) OR 'fiscal' OR 'funding' OR 'financial' OR 'finance' OR ('unit' NEXT/1 cost*) OR price* OR 'pricing' OR expenditure* OR expense* OR 'cost control' OR 'cost allocation' OR 'economic aspect' OR 'financial management'	2529724
**12**	(health*care NEAR/2 (utilisation OR utilization)) OR ('health care' NEAR/2 (utilisation OR utilization)) OR ('resource' NEAR/2 (utilisation OR utilization OR use))	128194
**13**	'resource use' OR 'healthcare resources' OR 'resource utilization' OR 'resource' OR 'health resource' OR 'healthcare resource'	373435
**14**	'patient readmission' OR 'patient admission' OR 'length of stay' OR readmi* OR rehosp* OR 'hospital readmission' OR 'reoperation' OR 'emergency room'	478028
**15**	'stay' NEAR/2 ('length' OR 'duration')	261527
**16**	(outpatient* OR 'clinic' OR physician* OR 'office' OR specialist* OR 'professional' OR 'practitioner') NEAR/2 (visit* OR 'care')	195800
**17**	('resource' OR 'staff') NEAR/4 (utilis* OR 'allocation')	34921
**18**	('high dependency' OR 'intensive') NEAR/2 ('unit' OR 'care')	591015
**19**	'resource allocation'/exp OR 'healthcare utilization'	34200

No.	Query	Results
**20**	('healthcare' OR 'health care' OR service* OR resource* OR hospital* OR 'clinic' OR 'clinics') NEAR/3 ('visits' OR 'utilisation' OR 'utilization' OR 'frequency' OR 'number' OR 'access') NEAR/3 ('patient' OR 'patients' OR 'parents' OR 'subjects' OR 'elderly' OR 'adults')	16256
**21**	'health care rationing' OR 'medical savings accounts' OR 'resource allocation' OR 'hospital stay' OR 'hospitalization' OR 'hospitalisation' OR 'hospitalised' OR 'hospitalized'	798391
**22**	'caregiver burden'/syn	12494
**23**	('caregiver' OR 'caregivers' OR 'family' OR 'adult children' OR 'spouses' OR 'family caregiver' OR 'carers' OR spous* OR senior* OR 'adult' OR 'adults' OR 'elderly' OR 'frail' OR pensioner* OR 'middle-aged' OR 'middle aged') NEAR/4 'burden'	20942
**24**	carer*:ab,ti OR caretaker*:ab,ti OR caregiver*:ab,ti OR 'care giver*':ab,ti OR 'caregiver time'	146361
**25**	'family caregiver'	3039
**26**	'family':ab,ti,kw OR 'families':ab,ti,kw OR 'kid':ab,ti,kw OR 'kids':ab,ti,kw OR child*:ab,ti,kw OR 'son':ab,ti,kw OR 'sons':ab,ti,kw OR daughter*:ab,ti,kw OR 'offspring':ab,ti,kw OR spouse*:ab,ti,kw OR 'partner':ab,ti,kw OR husband*:ab,ti,kw OR 'wife':ab,ti,kw OR 'wives':ab,ti,kw OR sibling*:ab,ti,kw OR grandchild*:ab,ti,kw OR parent*:ab,ti,kw OR sister*:ab,ti,kw OR brother*:ab,ti,kw OR uncle*:ab,ti,kw OR aunt*:ab,ti,kw OR niece*:ab,ti,kw OR nephew*:ab,ti,kw OR cousin*:ab,ti,kw	4385840
**27**	'adult children' OR 'spouses' OR 'siblings' OR 'only child' OR 'nuclear family' OR 'parents' OR 'family'	1803557
**28**	#26 OR #27	4723060
**29**	'care':ab,ti,kw OR 'caring':ab,ti,kw OR 'caregiving':ab,ti,kw OR carer*:ab,ti,kw OR caregiver*:ab,ti,kw OR 'cares':ab,ti,kw OR caretaker*:ab,ti,kw	2440024
**30**	'caregivers'	92629
**31**	#29 OR #30	2440047
**32**	#28 AND #31	595649
**33**	#22 OR #23 OR #24 OR #25 OR #32	660972
**34**	#4 OR #5 OR #6 OR #7 OR #8 OR #9 OR #10 OR #11 OR #12 OR #13 OR #14 OR #15 OR #16 OR #17 OR #18 OR #19 OR #20 OR #21 OR #33	4730827
**35**	'quality of life'/syn OR 'health related quality of life'/exp OR 'health-related quality of life'/exp	728634
**36**	'hrqol' OR 'hrql'/exp OR 'qol' OR 'quality life' OR 'life quality' OR 'health-related qol'	609304
**37**	'quality adjusted life' OR 'quality-adjust-life' OR 'qaly' OR 'qald' OR 'qale' OR 'qtime' OR 'quality adjusted life year'/syn OR 'life year*':ab,ti OR 'hql':ab,ti OR 'hqol':ab,ti OR 'h qol':ab,ti OR 'hr qol':ab,ti	49514

No.	Query	Results
**38**	'quality' NEAR/3 'life'	735493
**39**	'qols' OR 'quality of life scale' OR (('instrument' OR 'instruments') NEAR/3 ('quality of life' OR 'qol'))	12995
**40**	'quality of well-being' OR 'qwb'	499
**41**	'unmet need' OR 'burden' OR 'fear' OR 'phobia' OR 'anxiety' OR 'distress' OR 'stress' OR 'phobic' OR 'anxious' OR 'afraid' OR 'nervous' OR 'worry' OR 'humanistic' OR 'concern'	3881987
**42**	#35 OR #36 OR #37 OR #38 OR #39 OR #40 OR #41	4458881
**43**	'cohort study':ab,ti OR cohort*:ab,ti OR ((('follow up' OR 'followup') NEXT/1 ('study' OR 'studies')):ab,ti) OR 'retrospective study':ab,ti OR 'cohort analysis':ab,ti OR 'longitudinal study':ab,ti OR 'prospective study':ab,ti OR 'observational study':ab,ti OR ((cohort NEXT/1 stud*):ab,ti) OR ((cohort NEXT/1 analy*):ab,ti) OR 'register':ab,ti OR 'registry':ab,ti OR (('database' NEAR/2 'study'):ab,ti) OR (('real' NEXT/1 'world'):ab,ti) OR (('healthcare' NEXT/1 'record'):ab,ti) OR 'pragmatic trial':ab,ti OR 'real-world clinical trial':ab,ti OR 'pragmatic clinical trial':ab,ti OR 'real-world':ab,ti OR 'real world':ab,ti OR 'database':ab,ti OR 'real-life':ab,ti OR 'real life':ab,ti	2787150
**44**	#34 OR #42 OR #43	10198268
**45**	#3 AND #44	157215
**46**	'head and neck cancer'/exp OR 'hnscc':ab,ti OR 'hn scc':ab,ti OR 'scchn':ab,ti OR 'scc hn':ab,ti OR 'hnsc':ab,ti OR 'hnc':ab,ti	223288
**47**	'stomach cancer'/exp OR 'gastric cancer'	163407
**48**	'renal cell carcinoma'/exp OR (renal*:ab,ti AND (carcinoma*:ab,ti OR cancer*:ab,ti OR neoplasm*:ab,ti OR adeno*:ab,ti OR pyelocarcinoma*:ab,ti OR oncocytoma:ab,ti)) OR 'rcc':ab,ti	154832
**49**	#46 OR #47 OR #48	525902
**50**	#45 AND #49	11294
**51**	#45 AND #49 AND ([conference abstract]/lim OR [conference paper]/lim OR [conference review]/lim OR [editorial]/lim OR [letter]/lim OR [note]/lim OR [review]/lim)	5599
**52**	#45 AND #49 AND [animals]/lim NOT ([humans]/lim AND [animals]/lim)	65
**53**	#51 OR #52	5616
**54**	#50 NOT #53	5678
**55**	#50 NOT #53 AND [2012-2022]/py	3841

Table 2. Embase search results for Melanoma, bladder cancer, TNBC, and NSCLC (conducted on 13th July 2022)

No.	Query	Results
1	'early stage' OR 'early-stage' OR 'early cancer' OR 'early diagnosis'/exp OR 'preliminary stage' OR 'first stage' OR 'primary stage' OR 'initial stage' OR 'earliest stage' OR 'earlier stage' OR 'early presentation' OR (('early' OR 'earlier' OR 'earliest' OR 'primary' OR 'initial' OR 'preliminary' OR 'first') NEAR/3 ('stage' OR 'staging' OR 'cancer' OR 'disease' OR 'detect*' OR 'diagnos*')) OR 'early':ab,ti OR 'early detection' OR 'early detection of cancer' OR 'stage 1*' OR 'stage i*' OR 'stage one' OR 'stage-1*' OR 'stage-i*' OR 'stage-one' OR 'stage 2*' OR 'stage ii*' OR 'stage two' OR 'stage-2*' OR 'stage-ii*' OR 'stage-two' OR 'stage 3*' OR 'stage iii*' OR 'stage three' OR 'stage-3*' OR 'stage-iii*' OR 'stage-three' OR 'stage 1-2' OR 'stage 1,2' OR 'stage i-ii' OR 'stage i,ii' OR 'stage 1-3' OR 'stage 1,3' OR 'stage i-iii' OR 'stage i,iii' OR 'stage 2-3' OR 'stage 2,3' OR 'stage ii-iii' OR 'stage ii,iii' OR ('stage' NEAR/2 ('1*' OR 'one' OR '2*' OR 'two' OR '3*' OR 'three')) OR 'non-advanced' OR 'non advanced' OR 'not advanced' OR 'non-metastatic' OR 'non metastatic' OR 'not metastatic'	3046263
2	'recurrence' OR 'recurrent disease'/syn OR 'disease recurrence' OR 'periodic disease' OR 'recurrent' OR 'recurrences' OR 'recurred' OR 'recur*' OR 'recurren*' OR 'cancer recurrence'/syn OR 'recurrent cancer' OR 'recurrent carcinoma' OR 'recurrent tumor' OR 'recurrent tumour' OR 'tumor recurrence'/syn OR 'tumour recurrence' OR 'neoplasm recurrence' OR (('cancer' OR 'tumor' OR 'tumour' OR 'neoplasm') NEAR/3 ('return*' OR 'com* back' OR 'back' OR 'relapse*' OR 'recur*' OR 'residual' OR 'residue' OR 'reoccur*' OR 'reappear' OR 'repeat' OR 'reappearance')) OR 'relapse*'	1477303
3	#1 AND #2	289558
4	'value' NEAR/3 'money'	2935
5	'deductibles' AND 'coinsurance'	167
6	'societal cost' OR 'social cost' OR 'social care cost' OR 'out of pocket' OR 'out-of-pocket' OR 'patient cost' OR 'co-payment' OR 'co?payment' OR 'private expenditure' OR 'patient time' OR 'carer cost' OR 'carer expenditure' OR 'carer time' OR 'caregiver cost' OR 'caregiver expenditure' OR 'caregiver time' OR 'economic burden' OR 'cost burden' OR 'resource burden' OR 'financial burden' OR 'economic consequences' OR 'cost of illness' OR 'healthcare cost' OR 'cost of disease'	79981
7	'costly' OR 'costing' OR pharmacoeconomic* OR 'pharmaco economic*' OR financ* OR 'finances' OR 'financed' OR 'cost analysis' OR 'cost assessment' OR 'cost study'	482783
8	('value' NEAR/2 ('money' OR 'monetary')) OR (cost* NEAR/3 (treat* OR therap*))	71356
9	('out' NEAR/2 'pocket') OR (patient* NEAR/2 cost*) OR copay* OR (privat* NEAR/2 expendit*) OR ((carer* OR caregiv*) NEAR/2 (cost* OR expendit* OR time))	50571

No.	Query	Results
**10**	(econ* NEAR/2 ('burden' OR 'disease' OR 'assessment')) OR (cost* NEAR/2 (illness* OR health* OR 'burden' OR 'disease' OR 'assessment' OR estimate OR variable OR saving* OR sharing OR direct OR indirect OR employer OR drug OR hospital))	422489
**11**	'economics' OR 'cost' OR 'health care cost' OR 'drug cost' OR 'hospital cost' OR 'socioeconomics' OR 'health economics' OR 'health economics'/exp OR 'fee' OR 'charge*' OR 'budget' OR 'hospital finance' OR 'health care financing' OR 'low cost' OR 'high cost' OR (health*care NEXT/1 cost*) OR ('health care' NEXT/1 cost*) OR 'fiscal' OR 'funding' OR 'financial' OR 'finance' OR ('unit' NEXT/1 cost*) OR price* OR 'pricing' OR expenditure* OR expense* OR 'cost control' OR 'cost allocation' OR 'economic aspect' OR 'financial management'	2529724
**12**	(health*care NEAR/2 (utilisation OR utilization)) OR ('health care' NEAR/2 (utilisation OR utilization)) OR ('resource' NEAR/2 (utilisation OR utilization OR use))	128194
**13**	'resource use' OR 'healthcare resources' OR 'resource utilization' OR 'resource' OR 'health resource' OR 'healthcare resource'	373435
**14**	'patient readmission' OR 'patient admission' OR 'length of stay' OR readmi* OR rehosp* OR 'hospital readmission' OR 'reoperation' OR 'emergency room'	478028
**15**	'stay' NEAR/2 ('length' OR 'duration')	261527
**16**	(outpatient* OR 'clinic' OR physician* OR 'office' OR specialist* OR 'professional' OR 'practitioner') NEAR/2 (visit* OR 'care')	195800
**17**	('resource' OR 'staff') NEAR/4 (utilis* OR 'allocation')	34921
**18**	('high dependency' OR 'intensive') NEAR/2 ('unit' OR 'care')	591015
**19**	'resource allocation'/exp OR 'healthcare utilization'	34200
**20**	('healthcare' OR 'health care' OR service* OR resource* OR hospital* OR 'clinic' OR 'clinics') NEAR/3 ('visits' OR 'utilisation' OR 'utilization' OR 'frequency' OR 'number' OR 'access') NEAR/3 ('patient' OR 'patients' OR 'parents' OR 'subjects' OR 'elderly' OR 'adults')	16256
**21**	'health care rationing' OR 'medical savings accounts' OR 'resource allocation' OR 'hospital stay' OR 'hospitalization' OR 'hospitalisation' OR 'hospitalised' OR 'hospitalized'	798391
**22**	'caregiver burden'/syn	12494
**23**	('caregiver' OR 'caregivers' OR 'family' OR 'adult children' OR 'spouses' OR 'family caregiver' OR 'carers' OR spous* OR senior* OR 'adult' OR 'adults' OR 'elderly' OR 'frail' OR pensioner* OR 'middle-aged' OR 'middle aged') NEAR/4 'burden'	20942
**24**	carer*:ab,ti OR caretaker*:ab,ti OR caregiver*:ab,ti OR 'care giver*':ab,ti OR 'caregiver time'	146361
**25**	'family caregiver'	3039

No.	Query	Results
**26**	'family':ab,ti,kw OR 'families':ab,ti,kw OR 'kid':ab,ti,kw OR 'kids':ab,ti,kw OR child*:ab,ti,kw OR 'son':ab,ti,kw OR 'sons':ab,ti,kw OR daughter*:ab,ti,kw OR 'offspring':ab,ti,kw OR spouse*:ab,ti,kw OR 'partner':ab,ti,kw OR husband*:ab,ti,kw OR 'wife':ab,ti,kw OR 'wives':ab,ti,kw OR sibling*:ab,ti,kw OR grandchild*:ab,ti,kw OR parent*:ab,ti,kw OR sister*:ab,ti,kw OR brother*:ab,ti,kw OR uncle*:ab,ti,kw OR aunt*:ab,ti,kw OR niece*:ab,ti,kw OR nephew*:ab,ti,kw OR cousin*:ab,ti,kw	4385840
**27**	'adult children' OR 'spouses' OR 'siblings' OR 'only child' OR 'nuclear family' OR 'parents' OR 'family'	1803557
**28**	#26 OR #27	4723060
**29**	'care':ab,ti,kw OR 'caring':ab,ti,kw OR 'caregiving':ab,ti,kw OR carer*:ab,ti,kw OR caregiver*:ab,ti,kw OR 'cares':ab,ti,kw OR caretaker*:ab,ti,kw	2440024
**30**	'caregivers'	92629
**31**	#29 OR #30	2440047
**32**	#28 AND #31	595649
**33**	#22 OR #23 OR #24 OR #25 OR #32	660972
**34**	#4 OR #5 OR #6 OR #7 OR #8 OR #9 OR #10 OR #11 OR #12 OR #13 OR #14 OR #15 OR #16 OR #17 OR #18 OR #19 OR #20 OR #21 OR #33	4730827
**35**	'quality of life'/syn OR 'health related quality of life'/exp OR 'health-related quality of life'/exp	728634
**36**	'hrqol' OR 'hrql'/exp OR 'qol' OR 'quality life' OR 'life quality' OR 'health-related qol'	609304
**37**	'quality adjusted life' OR 'quality-adjust-life' OR 'qaly' OR 'qald' OR 'qale' OR 'qtime' OR 'quality adjusted life year'/syn OR 'life year*':ab,ti OR 'hql':ab,ti OR 'hqol':ab,ti OR 'h qol':ab,ti OR 'hr qol':ab,ti	49514
**38**	'quality' NEAR/3 'life'	735493
**39**	'qols' OR 'quality of life scale' OR (('instrument' OR 'instruments') NEAR/3 ('quality of life' OR 'qol'))	12995
**40**	'quality of well-being' OR 'qwb'	499
**41**	'unmet need' OR 'burden' OR 'fear' OR 'phobia' OR 'anxiety' OR 'distress' OR 'stress' OR 'phobic' OR 'anxious' OR 'afraid' OR 'nervous' OR 'worry' OR 'humanistic' OR 'concern'	3881987
**42**	#35 OR #36 OR #37 OR #38 OR #39 OR #40 OR #41	4458881
**43**	'cohort study':ab,ti OR cohort*:ab,ti OR ((('follow up' OR 'followup') NEXT/1 ('study' OR 'studies')):ab,ti) OR 'retrospective study':ab,ti OR 'cohort analysis':ab,ti OR 'longitudinal study':ab,ti OR 'prospective study':ab,ti OR 'observational study':ab,ti OR ((cohort NEXT/1 stud*):ab,ti) OR ((cohort NEXT/1 analy*):ab,ti) OR 'register':ab,ti OR 'registry':ab,ti OR (('database' NEAR/2 'study'):ab,ti) OR (('real' NEXT/1 'world'):ab,ti) OR (('healthcare' NEXT/1 'record'):ab,ti) OR 'pragmatic trial':ab,ti	2787150

No.	Query	Results
	OR 'real-world clinical trial':ab,ti OR 'pragmatic clinical trial':ab,ti OR 'real-world':ab,ti OR 'real world':ab,ti OR 'database':ab,ti OR 'real-life':ab,ti OR 'real life':ab,ti	
**44**	#34 OR #42 OR #43	1019826 8
**45**	#3 AND #44	119988
**46**	'melanoma'/exp OR 'melano*'	349567
**47**	'triple negative breast cancer'/exp OR (('breast cancer' OR 'breast tumor' OR 'breast tumour' OR 'breast neoplasm' OR 'breast carcinoma') NEAR/3 ('triple negative' OR 'triple-negative'))	33342
**48**	'bladder cancer'/exp OR (((bladder OR urothelial OR 'transitional cell') NEAR/3 (cancer OR cancers OR tumor OR tumors OR tumour OR tumours OR carcinoma OR carcinomas OR neoplasm OR neoplasms)):ab,ti) OR 'transitional cell carcinoma'/exp OR 'bladder tumor'/exp OR 'urinary bladder neoplasms'	135571
**49**	'non small cell lung cancer'/exp OR 'nsclc' OR 'non-small cell lung cancer' OR 'non-small-cell lung cancer' OR 'non-small':ab,ti OR 'non small':ab,ti OR 'nonsmall':ab,ti OR 'non-small cell':ab,ti OR 'non small cell':ab,ti OR 'nonsmallcell':ab,ti OR 'non-small-cell':ab,ti	210923
**50**	#46 OR #47 OR #48 OR #49	702719
**51**	#45 AND #50	9584
**52**	#45 AND #50 AND ([conference abstract]/lim OR [conference paper]/lim OR [conference review]/lim OR [editorial]/lim OR [letter]/lim OR [note]/lim OR [review]/lim)	5637
**53**	#45 AND #50 AND [animals]/lim NOT ([humans]/lim AND [animals]/lim)	91
**54**	#52 OR #53	5662
**55**	#51 NOT #54	3922
**56**	#51 NOT #54 AND [2012-2022]/py	2859

Table 3. Medline-In Process search for gastric cancer, RCC, and head and neck cancer (conducted on 21st July 2022)

No.	Query	Results
**1**	"early stage" OR "early-stage" OR "early cancer" OR "early diagnosis" [MeSH Terms] OR "Early Detection of Cancer" [MeSH Terms] OR "preliminary stage" OR "first stage" OR "primary stage" OR "initial stage" OR "earliest stage" OR "earlier stage" OR "early presentation" OR (("early" OR "earlier" OR "earliest" OR "primary" OR "initial" OR	5958413

No.	Query	Results
	"preliminary" OR "first") AND ("stage" OR "staging" OR "cancer" OR "disease" OR "detect*" OR "diagnos*")) OR "early" [Title/Abstract] OR "early detection" OR "early detection of cancer" OR "stage 1*" OR "stage i*" OR "stage one" OR "stage-1*" OR "stage-i*" OR "stage-one" OR "stage 2*" OR "stage ii*" OR "stage two" OR "stage-2*" OR "stage-ii*" OR "stage-two" OR "stage 3*" OR "stage iii*" OR "stage three" OR "stage-3*" OR "stage-iii*" OR "stage-three" OR "stage 4*" OR "stage iv*" OR "stage four" OR "stage-4*" OR "stage-iv*" OR "stage-four" OR "stage 1-2" OR "stage 1,2" OR "stage i-ii" OR "stage i,ii" OR "stage 1-3" OR "stage 1,3" OR "stage i-iii" OR "stage i,iii" OR "stage 2-3" OR "stage 2,3" OR "stage ii-iii" OR "stage ii,iii" OR "stage 1-4" OR "stage 1,4" OR "stage i-iv" OR "stage i,iv" OR "stage 2-4" OR "stage 2,4" OR "stage ii-iv" OR "stage ii,iv" OR "stage 3-4" OR "stage 3,4" OR "stage iii-iv" OR "stage iii,iv" OR ("stage" AND ("1" OR "one" OR "2" OR "two" OR "3" OR "three" OR "4" OR "four")) OR "non-advanced" OR "non advanced" OR "not advanced" OR "non-metastatic" OR "non metastatic" OR "not metastatic" OR "advanced" OR "advanc*" OR "locally advanced" OR ("local*" AND "advanc*")	
**2**	"recurrence" OR "recurrence" [MeSH Terms] OR "Neoplasm Recurrence, Local" [MeSH Terms] OR "recurrent disease" OR "disease recurrence" OR "periodic disease" OR "recurrent" OR "recurrences" OR "recurred" OR "recur*" OR "recurren*" OR "cancer recurrence" OR "recurrent cancer" OR "recurrent carcinoma" OR "recurrent tumor" OR "recurrent tumour" OR "tumor recurrence" OR "tumour recurrence" OR "neoplasm recurrence" OR (("cancer" OR "tumor" OR "tumour" OR "neoplasm") AND ("return*" OR "comes back" OR "coming back" OR "come back" OR "back" OR "relapse*" OR "recur*" OR "residual" OR "residue" OR "reoccur*" OR "reappear" OR "repeat" OR "reappearance")) OR "relapse*"	1070644
**3**	#1 AND #2	452539
**4**	"value" AND "money"	3957
**5**	"deductibles" AND "coinsurance"	1926
**6**	"societal cost" OR "social cost" OR "social care cost" OR "out of pocket" OR "out-of-pocket" OR "patient cost" OR "co-payment" OR "co?payment" OR "private expenditure" OR "patient time" OR "carer cost" OR "carer expenditure" OR "carer time" OR "caregiver cost" OR "caregiver expenditure" OR "caregiver time" OR "economic burden" OR "cost burden" OR "resource burden" OR "financial burden" OR "economic consequences" OR "cost of illness" OR "healthcare cost" OR "cost of disease"	75049
**7**	"costly" OR "costing" OR pharmacoeconomic* OR "pharmaco economic*" OR financ* OR "finances" OR "financed" OR "cost analysis" OR "cost assessment" OR "cost study"	879009
**8**	("value" AND ("money" OR "monetary")) OR (cost* AND (treat* OR therap*))	385978

No.	Query	Results
**9**	("out" AND "pocket") OR (patient* AND cost*) OR copay* OR (privat* AND expendit*) OR ((carer* OR caregiv*) AND (cost* OR expendit* OR time))	395852
**10**	(econ* AND ("burden" OR "disease" OR "assessment")) OR (cost* AND (illness* OR health* OR "burden" OR "disease" OR "assessment" OR estimate OR variable OR saving* OR sharing OR direct OR indirect OR employer OR drug OR hospital))	883998
**11**	"economics" OR "cost" OR "health care cost" OR "drug cost" OR "hospital cost" OR "socioeconomics" OR "health economics" OR "Health Care Economics and Organizations" [MeSH Terms] OR "fee" OR "charge*" OR "budget" OR "hospital finance" OR "health care financing" OR "low cost" OR "high cost" OR (health*care AND cost*) OR ("health care" AND cost*) OR "fiscal" OR "funding" OR "financial" OR "finance" OR ("unit" AND cost*) OR price* OR "pricing" OR expenditure* OR expense* OR "cost control" OR "cost allocation" OR "economic aspect" OR "financial management"	3333121
**12**	(health*care AND (utilisation OR utilization)) OR ("health care" AND (utilisation OR utilization)) OR ("resource" AND (utilisation OR utilization OR use))	466208
**13**	"resource use" OR "healthcare resources" OR "resource utilization" OR "resource" OR "health resource" OR "healthcare resource"	278509
**14**	"patient readmission" OR "patient admission" OR "length of stay" OR readmi* OR rehosp* OR "hospital readmission" OR "reoperation" OR "emergency room"	338714
**15**	"stay" AND ("length" OR "duration")	179561
**16**	(outpatient* OR "clinic" OR physician* OR "office" OR specialist* OR "professional" OR "practitioner") AND (visit* OR "care")	750271
**17**	("resource" OR "staff") AND (utilis* OR "allocation")	30465
**18**	("high dependency" OR "intensive") AND ("unit" OR "care")	360323
**19**	"resource allocation" [MeSH Terms] OR "healthcare utilization"	25071
**20**	("healthcare" OR "health care" OR service* OR resource* OR hospital* OR "clinic" OR "clinics") AND ("visits" OR "utilisation" OR "utilization" OR "frequency" OR "number" OR "access") AND ("patient" OR "patients" OR "parents" OR "subjects" OR "elderly" OR "adults")	895072
**21**	"health care rationing" OR "medical savings accounts" OR "resource allocation" OR "hospital stay" OR "hospitalization" OR "hospitalisation" OR "hospitalised" OR "hospitalized"	470345
**22**	"caregiver burden" [MeSH Terms]	502

No.	Query	Results
**23**	("caregiver" OR "caregivers" OR "family" OR "adult children" OR "spouses" OR "family caregiver" OR "carers" OR spous* OR senior* OR "adult" OR "adults" OR "elderly" OR "frail" OR pensioner* OR "middle-aged" OR "middle aged") AND "burden"	136794
**24**	carer* [Title/Abstract] OR caretaker* [Title/Abstract] OR caregiver* [Title/Abstract] OR "care giver*" [Title/Abstract] OR "caregiver time"	110110
**25**	"family caregiver"	2370
**26**	"family" [Title/Abstract] OR "families" [Title/Abstract] OR "kid" [Title/Abstract] OR "kids" [Title/Abstract] OR child* [Title/Abstract] OR "son" [Title/Abstract] OR "sons" [Title/Abstract] OR daughter* [Title/Abstract] OR "offspring" [Title/Abstract] OR spouse* [Title/Abstract] OR "partner" [Title/Abstract] OR husband* [Title/Abstract] OR "wife" [Title/Abstract] OR "wives" [Title/Abstract] OR sibling* [Title/Abstract] OR grandchild* [Title/Abstract] OR parent* [Title/Abstract] OR sister* [Title/Abstract] OR brother* [Title/Abstract] OR uncle* [Title/Abstract] OR aunt* [Title/Abstract] OR niece* [Title/Abstract] OR nephew* [Title/Abstract] OR cousin* [Title/Abstract]	3444361
**27**	"adult children" OR "spouses" OR "siblings" OR "only child" OR "nuclear family" OR "parents" OR "family"	1489309
**28**	#26 OR #27	3733498
**29**	"care" [Title/Abstract] OR "caring" [Title/Abstract] OR "caregiving" [Title/Abstract] OR carer* [Title/Abstract] OR caregiver* [Title/Abstract] OR "cares" [Title/Abstract] OR caretaker* [Title/Abstract]	1829611
**30**	"caregivers"	88865
**31**	#29 OR #30	1834326
**32**	#28 AND #31	453408
**33**	#22 OR #23 OR #24 OR #25 OR #32	609243
**34**	#4 OR #5 OR #6 OR #7 OR #8 OR #9 OR #10 OR #11 OR #12 OR #13 OR #14 OR #15 OR #16 OR #17 OR #18 OR #19 OR #20 OR #21 OR #33	5763952
**35**	"quality of life" [MeSH Terms] OR "health related quality of life" OR "health-related quality of life"	268895
**36**	"hrqol" OR "hrql" OR "qol" OR "quality life" OR "life quality" OR "health-related qol"	83076
**37**	"quality adjusted life" OR "quality-adjust-life" OR "qaly" OR "qald" OR "qale" OR "qtime" OR "quality adjusted life year"/syn OR "life year*" [Title/Abstract] OR "hql" [Title/Abstract] OR "hqol" [Title/Abstract] OR "h qol" [Title/Abstract] OR "hr qol" [Title/Abstract]	25801
**38**	"quality" AND "life"	509118
**39**	"qols" OR "quality of life scale" OR (("instrument" OR "instruments") AND ("quality of life" OR "qol"))	27044

No.	Query	Results
**40**	"quality of well-being" OR "qwb"	393
**41**	"unmet need" OR "burden" OR "fear" OR "phobia" OR "anxiety" OR "distress" OR "stress" OR "phobic" OR "anxious" OR "afraid" OR "nervous" OR "worry" OR "humanistic" OR "concern"	2576297
**42**	#35 OR #36 OR #37 OR #38 OR #39 OR #40 OR #41	2984174
**43**	"cohort study" [Title/Abstract] OR cohort* [Title/Abstract] OR ((("follow up" OR "followup") AND ("study" OR "studies")) [Title/Abstract]) OR "retrospective study" [Title/Abstract] OR "cohort analysis" [Title/Abstract] OR "longitudinal study" [Title/Abstract] OR "prospective study" [Title/Abstract] OR "observational study" [Title/Abstract] OR ((cohort AND stud*) [Title/Abstract]) OR ((cohort AND analy*) [Title/Abstract]) OR "register" [Title/Abstract] OR "registry" [Title/Abstract] OR (("database" AND "study") [Title/Abstract]) OR (("real" AND "world") [Title/Abstract]) OR (("healthcare" AND "record") [Title/Abstract]) OR "pragmatic trial" [Title/Abstract] OR "real-world clinical trial" [Title/Abstract] OR "pragmatic clinical trial" [Title/Abstract] OR "real-world" [Title/Abstract] OR "real world" [Title/Abstract] OR "database" [Title/Abstract] OR "real-life" [Title/Abstract] OR "real life" [Title/Abstract]	2896817
**44**	#34 OR #42 OR #43	9651572
**45**	#3 AND #44	242130
**46**	"Head and Neck Neoplasms" [MeSH Terms] OR "hnscc" [Title/Abstract] OR "hn scc" [Title/Abstract] OR "scchn" [Title/Abstract] OR "scc hn" [Title/Abstract] OR "hnsc" [Title/Abstract] OR "hnc" [Title/Abstract]	348083
**47**	"Stomach Neoplasms" [MeSH Terms] OR "gastric cancer"	130136
**48**	"Carcinoma, Renal Cell" [MeSH Terms] OR (renal* [Title/Abstract] AND (carcinoma* [Title/Abstract] OR cancer* [Title/Abstract] OR neoplasm* [Title/Abstract] OR adeno* [Title/Abstract] OR pyelocarcinoma* [Title/Abstract] OR oncocytoma [Title/Abstract])) OR "rcc" [Title/Abstract]	102720
**49**	#46 OR #47 OR #48	567696
**50**	#45 AND #49	24141
**51**	#45 AND #49 AND ‘conference review’ [Publication Type] OR editorial [Publication Type] OR letter [Publication Type] OR review [Publication Type]	4884733
**52**	#50 NOT #51	21929
**53**	#50 NOT #51	11232
**54**	#53 AND (inprocess[sb] OR pubstatusaheadofprint)	71

Table 4. Medline-In Process search for melanoma, TNBC, bladder cancer, and NSCLC (conducted on 21st July 2022)

No.	Query	Results
1	"early stage" OR "early-stage" OR "early cancer" OR "early diagnosis" [MeSH Terms] OR "Early Detection of Cancer" [MeSH Terms] OR "preliminary stage" OR "first stage" OR "primary stage" OR "initial stage" OR "earliest stage" OR "earlier stage" OR "early presentation" OR (("early" OR "earlier" OR "earliest" OR "primary" OR "initial" OR "preliminary" OR "first") AND ("stage" OR "staging" OR "cancer" OR "disease" OR "detect*" OR "diagnos*")) OR "early" [Title/Abstract] OR "early detection" OR "early detection of cancer" OR "stage 1*" OR "stage i*" OR "stage one" OR "stage-1*" OR "stage-i*" OR "stage-one" OR "stage 2*" OR "stage ii*" OR "stage two" OR "stage-2*" OR "stage-ii*" OR "stage-two" OR "stage 3*" OR "stage iii*" OR "stage three" OR "stage-3*" OR "stage-iii*" OR "stage-three" OR "stage 1-2" OR "stage 1,2" OR "stage i-ii" OR "stage i,ii" OR "stage 1-3" OR "stage 1,3" OR "stage i-iii" OR "stage i,iii" OR "stage 2-3" OR "stage 2,3" OR "stage ii-iii" OR "stage ii,iii" OR ("stage" AND ("1" OR "one" OR "2" OR "two" OR "3" OR "three")) OR "non-advanced" OR "non advanced" OR "not advanced" OR "non-metastatic" OR "non metastatic" OR "not metastatic"	4877925
2	"recurrence" OR "recurrence" [MeSH Terms] OR "Neoplasm Recurrence, Local" [MeSH Terms] OR "recurrent disease" OR "disease recurrence" OR "periodic disease" OR "recurrent" OR "recurrences" OR "recurred" OR "recur*" OR "recurren*" OR "cancer recurrence" OR "recurrent cancer" OR "recurrent carcinoma" OR "recurrent tumor" OR "recurrent tumour" OR "tumor recurrence" OR "tumour recurrence" OR "neoplasm recurrence" OR (("cancer" OR "tumor" OR "tumour" OR "neoplasm") AND ("return*" OR "comes back" OR "coming back" OR "come back" OR "back" OR "relapse*" OR "recur*" OR "residual" OR "residue" OR "reoccur*" OR "reappear" OR "repeat" OR "reappearance")) OR "relapse*"	1070644
3	#1 AND #2	414263
4	"value" AND "money"	3957
5	"deductibles" AND "coinsurance"	1926
6	"societal cost" OR "social cost" OR "social care cost" OR "out of pocket" OR "out-of-pocket" OR "patient cost" OR "co-payment" OR "co?payment" OR "private expenditure" OR "patient time" OR "carer cost" OR "carer expenditure" OR "carer time" OR "caregiver cost" OR "caregiver expenditure" OR "caregiver time" OR "economic burden" OR "cost burden" OR "resource burden" OR "financial burden" OR "economic consequences" OR "cost of illness" OR "healthcare cost" OR "cost of disease"	75049
7	"costly" OR "costing" OR pharmacoeconomic* OR "pharmaco economic*" OR financ* OR "finances" OR "financed" OR "cost analysis" OR "cost assessment" OR "cost study"	879009
8	("value" AND ("money" OR "monetary")) OR (cost* AND (treat* OR therap*))	385978

No.	Query	Results
**9**	("out" AND "pocket") OR (patient* AND cost*) OR copay* OR (privat* AND expendit*) OR ((carer* OR caregiv*) AND (cost* OR expendit* OR time))	395852
**10**	(econ* AND ("burden" OR "disease" OR "assessment")) OR (cost* AND (illness* OR health* OR "burden" OR "disease" OR "assessment" OR estimate OR variable OR saving* OR sharing OR direct OR indirect OR employer OR drug OR hospital))	883998
**11**	"economics" OR "cost" OR "health care cost" OR "drug cost" OR "hospital cost" OR "socioeconomics" OR "health economics" OR "Health Care Economics and Organizations" [MeSH Terms] OR "fee" OR "charge*" OR "budget" OR "hospital finance" OR "health care financing" OR "low cost" OR "high cost" OR (healthcare AND cost*) OR ("health-care" AND cost*) OR ("health care" AND cost*) OR "fiscal" OR "funding" OR "financial" OR "finance" OR ("unit" AND cost*) OR price* OR "pricing" OR expenditure* OR expense* OR "cost control" OR "cost allocation" OR "economic aspect" OR "financial management"	3345104
**12**	(healthcare AND (utilisation OR utilization)) OR (health-care AND (utilisation OR utilization)) OR ("health care" AND (utilisation OR utilization)) OR ("resource" AND (utilisation OR utilization OR use))	943296
**13**	"resource use" OR "healthcare resources" OR "resource utilization" OR "resource" OR "health resource" OR "healthcare resource"	278509
**14**	"patient readmission" OR "patient admission" OR "length of stay" OR readmi* OR rehosp* OR "hospital readmission" OR "reoperation" OR "emergency room"	338714
**15**	"stay" AND ("length" OR "duration")	179561
**16**	(outpatient* OR "clinic" OR physician* OR "office" OR specialist* OR "professional" OR "practitioner") AND (visit* OR "care")	750271
**17**	("resource" OR "staff") AND (utilis* OR "allocation")	30465
**18**	("high dependency" OR "intensive") AND ("unit" OR "care")	360323
**19**	"resource allocation" [MeSH Terms] OR "healthcare utilization"	25071
**20**	("healthcare" OR "health care" OR service* OR resource* OR hospital* OR "clinic" OR "clinics") AND ("visits" OR "utilisation" OR "utilization" OR "frequency" OR "number" OR "access") AND ("patient" OR "patients" OR "parents" OR "subjects" OR "elderly" OR "adults")	895072
**21**	"health care rationing" OR "medical savings accounts" OR "resource allocation" OR "hospital stay" OR "hospitalization" OR "hospitalisation" OR "hospitalised" OR "hospitalized"	470345

No.	Query	Results
**22**	"caregiver burden" [MeSH Terms]	502
**23**	("caregiver" OR "caregivers" OR "family" OR "adult children" OR "spouses" OR "family caregiver" OR "carers" OR spous* OR senior* OR "adult" OR "adults" OR "elderly" OR "frail" OR pensioner* OR "middle-aged" OR "middle aged") AND "burden"	136794
**24**	carer* [Title/Abstract] OR caretaker* [Title/Abstract] OR caregiver* [Title/Abstract] OR "care giver*" [Title/Abstract] OR "caregiver time"	110110
**25**	"family caregiver"	2370
**26**	"family" [Title/Abstract] OR "families" [Title/Abstract] OR "kid" [Title/Abstract] OR "kids" [Title/Abstract] OR child* [Title/Abstract] OR "son" [Title/Abstract] OR "sons" [Title/Abstract] OR daughter* [Title/Abstract] OR "offspring" [Title/Abstract] OR spouse* [Title/Abstract] OR "partner" [Title/Abstract] OR husband* [Title/Abstract] OR "wife" [Title/Abstract] OR "wives" [Title/Abstract] OR sibling* [Title/Abstract] OR grandchild* [Title/Abstract] OR parent* [Title/Abstract] OR sister* [Title/Abstract] OR brother* [Title/Abstract] OR uncle* [Title/Abstract] OR aunt* [Title/Abstract] OR niece* [Title/Abstract] OR nephew* [Title/Abstract] OR cousin* [Title/Abstract]	3444361
**27**	"adult children" OR "spouses" OR "siblings" OR "only child" OR "nuclear family" OR "parents" OR "family"	1489309
**28**	#26 OR #27	3733498
**29**	"care" [Title/Abstract] OR "caring" [Title/Abstract] OR "caregiving" [Title/Abstract] OR carer* [Title/Abstract] OR caregiver* [Title/Abstract] OR "cares" [Title/Abstract] OR caretaker* [Title/Abstract]	1829611
**30**	"caregivers"	88865
**31**	#29 OR #30	1834326
**32**	#28 AND #31	453408
**33**	#22 OR #23 OR #24 OR #25 OR #32	609243
**34**	#4 OR #5 OR #6 OR #7 OR #8 OR #9 OR #10 OR #11 OR #12 OR #13 OR #14 OR #15 OR #16 OR #17 OR #18 OR #19 OR #20 OR #21 OR #33	5935131
**35**	"quality of life" [MeSH Terms] OR "health related quality of life" OR "health-related quality of life"	268895
**36**	"hrqol" OR "hrql" OR "qol" OR "quality life" OR "life quality" OR "health-related qol"	83076
**37**	"quality adjusted life" OR "quality-adjust-life" OR "qaly" OR "qald" OR "qale" OR "qtime" OR "quality-adjusted life years"[MeSH Terms] OR "life year*" [Title/Abstract] OR "hql" [Title/Abstract] OR "hqol" [Title/Abstract] OR "h qol" [Title/Abstract] OR "hr qol" [Title/Abstract]	31460

No.	Query	Results
**38**	"quality" AND "life"	509118
**39**	"qols" OR "quality of life scale" OR (("instrument" OR "instruments") AND ("quality of life" OR "qol"))	27044
**40**	"quality of well-being" OR "qwb"	393
**41**	"unmet need" OR "burden" OR "fear" OR "phobia" OR "anxiety" OR "distress" OR "stress" OR "phobic" OR "anxious" OR "afraid" OR "nervous" OR "worry" OR "humanistic" OR "concern"	2576297
**42**	#35 OR #36 OR #37 OR #38 OR #39 OR #40 OR #41	2987080
**43**	"cohort study"[Title/Abstract] OR cohort*[Title/Abstract] OR (("follow up" OR "followup") AND ("study"[Title/Abstract] OR "studies"[Title/Abstract])) OR "retrospective study"[Title/Abstract] OR "cohort analysis"[Title/Abstract] OR "longitudinal study"[Title/Abstract] OR "prospective study"[Title/Abstract] OR "observational study"[Title/Abstract] OR (cohort [Title/Abstract] AND stud*[Title/Abstract]) OR (cohort[Title/Abstract] AND analy*[Title/Abstract]) OR "register"[Title/Abstract] OR "registry"[Title/Abstract] OR ("database"[Title/Abstract] AND "study"[Title/Abstract]) OR ("real" [Title/Abstract] AND "world" [Title/Abstract]) OR ("healthcare" [Title/Abstract] AND "record" [Title/Abstract]) OR "pragmatic trial"[Title/Abstract] OR "real-world clinical trial"[Title/Abstract] OR "pragmatic clinical trial"[Title/Abstract] OR "real-world"[Title/Abstract] OR "real world"[Title/Abstract] OR "database"[Title/Abstract] OR "real-life"[Title/Abstract] OR "real life"[Title/Abstract]	2440884
**44**	#34 OR #42 OR #43	9430998
**45**	#3 AND #44	204037
**46**	"melanoma" [MeSH Terms] OR "melano*"	257421
**47**	"triple negative breast neoplasms" [MeSH Terms] OR (("breast cancer" OR "breast tumor" OR "breast tumour" OR "breast neoplasm" OR "breast carcinoma") AND ("triple negative" OR "triple-negative"))	20197
**48**	"urinary bladder neoplasms" [MeSH Terms] OR "urinary bladder neoplasms" OR ((bladder [Title/Abstract] OR urothelial [Title/Abstract] OR "transitional cell" [Title/Abstract]) AND (cancer [Title/Abstract] OR cancers [Title/Abstract] OR tumor [Title/Abstract] OR tumors [Title/Abstract] OR tumour [Title/Abstract] OR tumours [Title/Abstract] OR carcinoma [Title/Abstract] OR carcinomas [Title/Abstract] OR neoplasm [Title/Abstract] OR neoplasms [Title/Abstract])) OR "carcinoma, transitional cell" [MeSH Terms] OR "bladder tumor"	104236
**49**	"Carcinoma, Non-Small-Cell Lung" [MeSH Terms] OR "nsclc" OR "non-small cell lung cancer" OR "non-small-cell lung cancer" OR "non-small" [Title/Abstract] OR "non small" [Title/Abstract] OR "nonsmall" [Title/Abstract] OR "non-small	98787

No.	Query	Results
	cell" [Title/Abstract] OR "non small cell" [Title/Abstract] OR "nonsmallcell" [Title/Abstract] OR "non-small-cell" [Title/Abstract]	
**50**	#46 OR #47 OR #48 OR #49	474376
**51**	#45 AND #50	14387
**52**	#45 AND #50 AND ‘conference review’ [Publication Type] OR editorial [Publication Type] OR letter [Publication Type] OR review [Publication Type]	4884733
**53**	#51 NOT #52	12758
**54**	#51 NOT #52	8040
**55**	#54 AND (inprocess[sb] OR pubstatusaheadofprint)	61

Appendix S2. Definitions of recurrence overview

Table 5: Definitions of recurrences provided in the included studies (n=22)

Study name	Disease	Outcome	Definition
Rasmussen 2019 (1)	Bladder cancer	Recurrence/ Remission	The patients were considered in complete cancer remission/cancer-free period if there was no evidence of active disease in the DNPR, that is no registrations of malignant pathology, no new malignant diagnoses and no cancer-related procedural codes.
Canter 2014 (2)		Recurrence	Recurrence was defined as any tumor present after initial complete resection at any surveillance point. Stage progression was defined as muscle invasive pathology at any surveillance point.
Chamie 2013 (2)		Recurrence	Recurrence as an inclusive state that includes: 1) recurrence without progression (receipt of a transurethral resection of a bladder tumor); 2) progression (receipt of radical cystectomy, radiotherapy, or systemic chemotherapy); or 3) bladder cancer-related death occurring > 90 days after diagnosis.
Olsson 2013 (3)		Recurrence	Tumour at re-resection was considered as a recurrence.
Chaux 2012 (4)		Recurrence	Recurrence was defined as the histopathologically proven reappearance of a pTa urothelial carcinoma during follow-up.
Thomas 2013 (5)		Recurrence	patients with one or more previous high-risk tumours.
Simon 2019 (6)		Recurrence	The time to first recurrence was defined as a new TaG1 or Ta low grade/ G2 tumor in the same way
Kolla 2021 (7)	Melanoma	Recurrence	Detection of disease in patients who had no residual disease following intervention.
Loidi 2021 (8)		Relapse	Relapses were divided into two categories: locoregional (including intralymphatic metastasis and regional nodes) and haemato-visceral (including distant nodes and visceral ones). Haemato-visceral metastasis was considered as multiple dissemination if more than 2 organs were affected. In case of synchronous locoregional and distant relapse, it was considered as haemato-visceral recurrence.
Sarac 2020 (9)		Late recurrence	Late recurrence defined as recurrence diagnosed after 10 years.

Study name	Disease	Outcome	Definition
		Early recurrence	Early recurrence defined as recurrence within 10 years.
Feigelson 2019 (10)		Recurrence	Defined as a detected melanoma that occurs after a patient is declared disease free after completion of definitive therapy (e.g., excision, radiation, and/or chemotherapy).
Von Schuckman 2019 (11)		Recurrence	Histologic or radiologic evidence of a metastatic melanoma deposit diagnosed at least 1 month after diagnosis of the primary tumor
Osella-Abate 2015 (12)		Late recurrence	Melanoma patients who recur 10 years after the diagnosis
Jang 2020 (13)		Recurrence	Identified by metastases, end-of-life care, death, or cancer treatment (chemotherapy, radiotherapy, or surgery) post-index, following a 3-month treatment-free interval after the primary treatment.
Karacz 2020 (14)	NSCLC	Recurrence	Local: recurrence in initial primary organ<br>Regional: recurrence in adjacent organ or lymph nodes draining the organ<br>Distant: recurrence in a location beyond regional.
Martin 2022 (15)		Relapse	Relapses was defined as disease progression or death.
Haiderali 2021b (16)	TNBC	Recurrence	Locoregional recurrence to metastatic diagnosis, death or end of record
Ignatov 2018 (17)		Recurrence	Loco-regional relapse included the recurrence in ipsilateral breast, chest wall or regional lymph nodes.<br>Distant recurrences consist of distant lymph node metastases (beyond the ipsilateral axillary, infra- and/or supraclavicular, internal mammary area), bone (including bone marrow), brain, liver, lung (including pleura and lymphangitic carcinomatosis), other (including peritoneal, other organs not elsewhere classified and skin not in the breast and chest wall).
Van Roozendaal 2016 (18)		Recurrence	A local recurrence was registered as an event in the ipsilateral breast (or overlying skin) with similar morphologic characteristics as the primary tumor. Events located elsewhere in the body were registered as distant recurrence.
Matro 2015 (19)		Recurrence	Diagnosis of first site of recurrence was identified based on the first date of diagnosis of recurrent disease.

Study name	Disease	Outcome	Definition
Eralp 2014 (20)		Recurrence	Locoregional recurrence was defined as involvement of the ipsilateral axillary, internal mammarian or supraclavicular lymph nodes and/or skin or subcutaneous tissue with/without ipsilateral breast parenchyma involvement.
Min Sun Bae 2021 (21)		Recurrence	Recurrence was defined as local-regional recurrence (including patients with in-breast recurrence and regional recurrences in the axilla, chest wall, internal mammary, or supraclavicular fossa lymph nodes), contralateral cancer, or distant metastasis.

Abbreviations: DNPR: Danish National Patient Registry; NSCLC: Non-Small Cell Lung Cancer; TNBC: Triple Negative Breast Cancer

Appendix S3. Clinical review findings overview

Bladder cancer

Table 6: Recurrence rates in patients with bladder cancer (n = 14 studies)

Study name	Country	Patient population	Type of subgroup	N	Timepoint	Follow-up	n (%) patients with recurrence	*p*-value
Garg 2021 (22)	USA	NMIBC	Ta/T1 low and high grade; Tis or Ta with CIS	2956	Mean follow-up time for each stage/ Grade: 51.1 (53.8)	Median: 29.4 months	1062 (35.9)	<0.05
		NMIBC	PUNLMP	327	Mean (SD) follow-up time for each stage/ Grade: 54.5 (42.8) months		78 (23.9)	
		NMIBC	Ta Low grade	1420	Mean (SD) follow-up time for each stage/ Grade: 59.5 (59.7) months		509 (35.9)	
		NMIBC	Ta high grade	371	Mean (SD) follow-up time for each stage/ Grade: 35.4 (36.8) months		133 (35.9)	
		NMIBC	Tis or Ta with CIS	211	Mean (SD) follow-up time for each stage/ Grade: 43.6 (51.5) months		110 (52.1)	

**Study name**	**Country**	**Patient population**	**Type of subgroup**	**N**	**Timepoint**	**Follow-up**	**n (%) patients with recurrence**	***p*-value**
		NMIBC	T1 Low grade	126	Mean (SD) follow-up time for each stage/ Grade: 59.1 (63.3) months		59 (46.8)	
		NMIBC	T1 high grade	501	Mean (SD) follow-up time for each stage/ Grade: 37.7 (45.4) months		173 (34.5)	
		NMIBC	PUNLMP-Low grade (<T2)	327	1 year		10.33 %	NR
				327	3 years		22.95 %	NR
				327	5 years		25.79 %	NR
		NMIBC	Ta Low grade (<2)	1420	1 year		17.19 %	NR
				1420	3 years		30.28 %	NR
				1420	5 years		36.75 %	NR
		NMIBC:	Ta High grade (<2)	371	1 year		24.29 %	NR
				371	3 years		38.64 %	NR
				371	5 years		42.19 %	NR
		NMIBC	T1 High grade (<2)	501	1 year		29.5 %	NR
				501	3 years		36.28 %	NR
				501	5 years		39.2 %	NR
		NMIBC	T1 Low grade (<2)	126	1 year		29.5 %	NR
				126	3 years		37.38 %	NR
				126	5 years		43.77 %	NR
		NMIBC:	Ta/Tis Carcinoma low/high grade (<2)	211	1 year		35.65 %	NR
				211	3 years		49.84 %	NR
				211	5 years		52.29 %	NR
	UK	NMIBC	NR	75			29 (38.7)	NR

Study name	Country	Patient population	Type of subgroup	N	Timepoint	Follow-up	n (%) patients with recurrence	*p*-value
Parsons 2020 (23)		Low risk (NMIBC)	NR	17	Maximum follow up 59.4 months	Mean (range): 23.4 (0.6–59.4) months	4 (23.5)	NR
		Intermediate risk (NMIBC)	NR	13			3 (23.1)	NR
		High risk (NMIBC)	NR	45			22 (48.9)	NR
Rasmussen 2019 (1)	Denmark	Bladder cancer	Female: T1-T4, N0-N4	417	1 year	NR	15.51%	NR
					2 years		19.66%	NR
					3 years		21.14%	NR
					5 years		22.90%	NR
			Male: T1-T4, N0-N4	1525	1 year		15.70%	NR
					2 years		19.72%	NR
					3 years		21.38%	NR
					5 years		23.82%	NR
			Female: T1-T4, N0-N4	417	NR	Median 40 months (IQR: 18–69 months)	117 (28.1%)	NR
			Male: T1-T4, N0-N4	1525	NR	Median 29 months (IQR: 11–58)	290 (19%)	NR
Ratanapornsompon 2019 (24)	Thailand	NMIBC	T1 High-Grade	70	Maximum follow up 252.8 months	Median (range): 40.1 (2.0 to 252.8)	39 (55.7)	NR
	NR		Overall	165	1 year		14.00%	NR

Study name	Country	Patient population	Type of subgroup	N	Timepoint	Follow-up	n (%) patients with recurrence	*p*-value
Lee 2019 (25)		Primary, solitary, Ta, low-grade tumors <3 cm		165	5 years	Median (IQR): 79 (47–118)	37.00%	NR
			Tumor ≤1.0 cm	75	1 year		8.00%	NR
			Tumor >1.0 cm	90			19.00%	
			Tumor ≤1.0 cm	75	5 years		23.00%	
			Tumor >1.0 cm	90			49.00%	
		Low-risk NMIBC	Tumour ≤1.0 cm	75	2 years		9 (12)	
		Low-risk NMIBC	Tumour >1.0 cm	90			27 (30)	
		Overall: Low-risk NMIBC	Overall	165	NR	Median: 79 months (IQR: 47-118)	66 (40%)	
			Tumor ≤1.0 cm	75	NR		20 (27%)	
			Tumor >1.0 cm	90	NR		46 (51%)	
		NIMBC-Recurrence patients	Female	66	NR		11 (17%)	
			Male	66	NR		55 (83%)	
Simon 2019 (6)	France	NMIBC (Patients free of recurrence at year 2), followed from 2-6 years*	Low grade (TaG1)	NR	4 years	Median (IQR): 7.2 (4.2–10.9) Years	30%; 95% CI: 23-37	NR

Study name	Country	Patient population	Type of subgroup	N	Timepoint	Follow-up	n (%) patients with recurrence	*p*-value
		NMIBC (Patients free of recurrence at year 3), followed from 3-7 years*	Low grade (TaG1)	NR	4 years		24%; 95% CI: 17-32	NR
		NMIBC (Patients free of recurrence at year 4), followed from 4-8 years*	Low grade (TaG1)	NR	4 years		20%; 95% CI: 14-28	NR
		NMIBC (Patients free of recurrence at year 5), followed from 5-9 years*	Low grade (TaG1)	NR	4 years		15%; 95% CI: 11-25	NR
		Primary low grade (TaG1) NMIBC	Primary low grade (TaG1)	NR	1 year		29%	NR
				NR	2 years		43%	NR
				NR	4 years		53%	NR
				NR	6 years		60%	NR
				NR	10 years		61%	NR
			Overall	470	NR		251 (53.4%)	NR
Comperat 2015 (26)	France	UBC patients	NR	142	Maximum follow-up: 207.5 months	Mean: 87 months	36 (25)	NR
		NMIBC	Low grade pTa	55			16 (29)	NR

Study name	Country	Patient population	Type of subgroup	N	Timepoint	Follow-up	n (%) patients with recurrence	*p*-value
			High grade pTa	45		Median: 49.5 months	11 (24.4)	
		PUNLMP	NR	42			7 (16.67)	
		UBC: Recurrent population	Male	106			26 (24.53)	NR
			Female	36			10 (27.78)	NR
			NMIBC	122			32 (26.23)	NR
			Stage: Tis	1			1 (100)	NR
			Stage: Ta	103			28 (27.18)	NR
			Stage: T1	19			3 (15)	NR
			MIBC	20			4 (20)	NR
			Stage: T2	10			4 (40)	NR
			Stage: T3	6			0 (0)	NR
			Stage: T4	4			0 (0)	NR
Canter 2014 (2)	USA	BC	High-Grade T1	222	Maximum follow up 261.2 months	Mean: 50.8; Median (Range): 32.5 (2.2-261.2) months	112 (50.5)	NR
					1 year		43.69%	NR
					3 years		54.71%	NR
					5 years		54.68%	NR
Chamie 2013 (27)	USA	Non-metastatic, high-grade (poorly or undifferentiated), urothelial, NMIBC	Overall	7410	2 years	5 years	NR (61.1)	NR
			Overall		5 years		NR (69.5)	NR
			Overall		10 years		NR (74.3)	NR
			Age: 66-69	985	2 years		NR (61.7)	NR
			Age: 70-74	1731	2 years		NR (61.7)	NR
			Age: 75-79	1825	2 years		NR (62.7)	NR
			Age: >/=80	2869	2 years		NR (59.4)	NR
			Male	5597	2 years		NR (61)	NR

Study name	Country	Patient population	Type of subgroup	N	Timepoint	Follow-up	n (%) patients with recurrence	*p*-value
			Female	1813	2 years		NR (61.3)	NR
			White	6742	2 years		NR (61.1)	NR
			Black	235	2 years		NR (62.8)	NR
			Hispanic	188	2 years		NR (66)	NR
			Others	245	2 years		NR (55.9)	NR
			Not married	2804	2 years		NR (61.1)	NR
			Married	4606	2 years		NR (61.1)	NR
			Charlson score: 0	4868	2 years		NR (60.8)	NR
			Charlson score: 1	1635	2 years		NR (63)	NR
			Charlson score: 2	587	2 years		NR (57.3)	NR
			Charlson score: >=3	320	2 years		NR (62)	NR
			% bachelor's degree: <15%	1627	2 years		NR (59.6)	NR
			% bachelor's degree: 15%-25%	1906	2 years		NR (62.4)	NR
			% bachelor's degree: 25%-35%	1608	2 years		NR (61.3)	NR
			% bachelor's degree: >35%	2269	2 years		NR (60.9)	NR

Study name	Country	Patient population	Type of subgroup	N	Timepoint	Follow-up	n (%) patients with recurrence	*p*-value
			Household income: <$35,000	1303	2 years		NR (62.1)	NR
			Household income: $35,000-$45,000	1770	2 years		NR (60.6)	NR
			Household income: $45,001-$55,000	1893	2 years		NR (60.8)	NR
			Household income: >$55,000	2444	2 years		NR (61.1)	NR
			Grade: Poorly differentiated	5785	2 years		NR (61)	NR
			Grade: Undifferentiated	1625	2 years		NR (61.3)	NR
			Stage: Stage, Ta	2398	2 years		NR (56.8)	NR
			Stage: Stage, Tis	754	2 years		NR (57.3)	NR
			Stage: Stage, T1	4258	2 years		NR (64.2)	NR
			Age: 66-69	985	5 years		NR (70.1)	NR
			Age: 70-74	1731	5 years		NR (70.1)	NR

Study name	Country	Patient population	Type of subgroup	N	Timepoint	Follow-up	n (%) patients with recurrence	*p*-value
			Age: 75-79	1825	5 years		NR (71.1)	NR
			Age: >/=80	2869	5 years		NR (67.8)	NR
			Male	5597	5 years		NR (69.4)	NR
			Female	1813	5 years		NR (69.7)	NR
			White	6742	5 years		NR (69.5)	NR
			Black	235	5 years		NR (71.2)	NR
			Hispanic	188	5 years		NR (74.2)	NR
			Others	245	5 years		NR (64.3)	NR
			Not married	2804	5 years		NR (69.5)	NR
			Married	4606	5 years		NR (69.5)	NR
			Charlson score: 0	4868	5 years		NR (69.2)	NR
			Charlson score: 1	1635	5 years		NR (71.4)	NR
			Charlson score: 2	587	5 years		NR (65.7)	NR
			Charlson score: 3	320	5 years		NR (70.4)	NR
			% bachelor's degree: <15%	1627	5 years		NR (68)	NR
			% bachelor's degree: 15%-25%	1906	5 years		NR (70.8)	NR
			% bachelor's degree: 25%-35%	1608	5 years		NR (69.7)	NR

Study name	Country	Patient population	Type of subgroup	N	Timepoint	Follow-up	n (%) patients with recurrence	*p*-value
			% bachelor's degree: >35%	2269	5 years		NR (69.3)	NR
			Household income: <$35,000	1303	5 years		NR (70.5)	NR
			Household income: $35,000-$45,000	1770	5 years		NR (69)	NR
			Household income: $45,001-$55,000	1893	5 years		NR (69.2)	NR
			Household income: >$55,000	2444	5 years		NR (69.5)	NR
			Grade: Poorly differentiated	5785	5 years		NR (69.4)	NR
			Grade: Undifferentiated	1625	5 years		NR (69.7)	NR
			Stage: Ta	2398	5 years		NR (65.2)	NR
			Stage: Tis	754	5 years		NR (65.7)	NR
			Stage: T1	4258	5 years		NR (72.5)	NR
			Age: 66-69	985	10 years		NR (74.9)	NR

Study name	Country	Patient population	Type of subgroup	N	Timepoint	Follow-up	n (%) patients with recurrence	*p*-value
			Age: 70-74	1731	10 years		NR (74.9)	0.98 vs 66-69 years
			Age: 75-79	1825	10 years		NR (75.8)	0.55 vs 66-69 years
			Age: >/=80	2869	10 years		NR (72.7)	0.16 vs 66-69 years
			Male	5597	10 years		NR (74.2)	NR
			Female	1813	10 years		NR (74.5)	0.81 vs Male
			White	6742	10 years		NR (74.3)	NR
			Black	235	10 years		NR (75.9)	0.56 vs White
			Hispanic	188	10 years		NR (78.8)	0.11 vs White
			Others	245	10 years		NR (69.2)	0.08 vs White
			Not married	2804	10 years		NR (74.2)	NR
			Married	4606	10 years		NR (74.3)	0.96 vs Not married
			Charlson score: 0	4868	10 years		NR (74)	NR
			Charlson score: 1	1635	10 years		NR (76.1)	0.08 vs score 0
			Charlson score: 2	587	10 years		NR (70.6)	0.08 vs score 0
			Charlson score: >=3	320	10 years		NR (75.1)	0.65 vs score 0
			% bachelor's degree: <15%	1627	10 years		NR (72.9)	NR

Study name	Country	Patient population	Type of subgroup	N	Timepoint	Follow-up	n (%) patients with recurrence	*p*-value
			% bachelor's degree: 15%-25%	1906	10 years		NR (75.5)	0.07 vs <15%
			% bachelor's degree: 25%-35%	1608	10 years		NR (74.5)	0.34 vs <15%
			% bachelor's degree: >35%	2269	10 years		NR (74.1)	0.49 vs <15%
			Household income: <$35,000	1303	10 years		NR (75.2)	NR
			Household income: $35,000-$45,000	1770	10 years		NR (73.8)	0.38 vs <$35,000
			Household income: $45,001-$55,000	1893	10 years		NR (74)	0.48 vs <$35,000
			Household income: >$55,000	2444	10 years		NR (74.3)	0.62 vs <$35,000
			Grade: Poorly differentiated	5785	10 years		NR (74.2)	NR
			Grade: Undifferentiated	1625	10 years		NR (74.5)	0.79 vs Poorly differentiated

Study name	Country	Patient population	Type of subgroup	N	Timepoint	Follow-up	n (%) patients with recurrence	*p*-value
			Stage: Ta	2398	10 years		NR (70.1)	NR
			Stage: Tis	754	10 years		NR (70.6)	0.79 vs Stage, Ta
			Stage: T1	4258	10 years		NR (77.2)	<.01 vs Stage, Ta
Olsson 2013 (3)	Sweden	Primary T1 UCB (recurrence patients)	Stage: T1	211	192 months	Median (Range): 60 (3-192) months	168 (80)	
			Female	36			30 (14.2)	NR
			Male	175			139 (65.9)	NR
			Age: <73 years	103			86 (40.8)	NR
			Age: >73 years	108			83 (39.3)	NR
			WHO grade 99: II	36			30 (14.2)	NR
			WHO grade 99: III	175			139 (65.9)	NR
			LVI: No	150			117 (55.5)	NR
			LVI: Suspected	45			38 (18.0)	NR
			LVI: Yes	16			14 (6.6)	NR
			Tumour size: <30 mm	102			77 (36.5)	NR
			Tumour size: >30 mm	109			92 (43.6)	NR
			Multiplicity: No	146			112 (53.1)	NR
			Multiplicity: Yes	65			57 (27.0)	NR

Study name	Country	Patient population	Type of subgroup	N	Timepoint	Follow-up	n (%) patients with recurrence	*p*-value
			Tumour volume: <Median	113			91 (43.1)	NR
			Tumour volume: >Median	98			78 (37.0)	NR
			Tumour volume proportion: <0.65 (median)	106			86 (40.8)	NR
			Tumour volume proportion: >0.65 (median)	105			83 (39.3)	NR
			Substage pT1a	75			57 (27.0)	NR
			Substage pT1b	81			71 (33.6)	NR
			Substage pT1c	55			41 (19.4)	NR
Thomas 2013 (5)	UK	NMIBC-High risk NMIBC	Overall NMIBC-high risk population	809	NR		318 (39.31%)	NR
			pTis- high risk population				130 (26%)	NR

Study name	Country	Patient population	Type of subgroup	N	Timepoint	Follow-up	n (%) patients with recurrence	*p*-value
			pTa- high risk population			Recurrent high-risk tumours: Mean follow–up: 59.4 months [IQR: 6–188])	139 (28%)	NR
			pT1- high risk population				221 (45%)	NR
			Grade 1- high risk population				4 (1%)	NR
			Grade 2- high risk population				66 (13%)	NR
			Grade 3- high risk population				424 (86%)	NR
Chaux 2012 (4)	USA	High-grade papillary urothelial carcinoma	Overall	85	Maximum follow-up: 240 months	24 months	31 (36.5)	NR
Yu 2021 (28)	South Korea	Urachal carcinoma of bladder	Stage I	48	3 years	Mean: 65 months	11.40%	NR
			Stage II	108			30%	NR
			Stage III	23			63.90%	NR
			Stage I	48	5 years		19.10%	NR
			Stage II	108			33.40%	NR
			Stage III	23			63.90%	NR
Sultana 2021 (29)	Europe	NMIBC; G1pTa Urothelial BC	Ta Low-risk NMIBC	180	1 years	NR	12 (6.7)	NR
			NMIBC	129	5 years		28 (21.7)	NR

Study name	Country	Patient population	Type of subgroup	N	Timepoint	Follow-up	n (%) patients with recurrence	*p*-value
			NMIBC	24	10 years		3 (12.5)	NR
		NMIBC; G2pTa Urothelial BC	Ta Low-risk NMIBC	243	1 years		27 (11.1)	NR
			NMIBC	150	5 years		50 (33.3)	NR
			NMIBC	43	10 years		16 (37.2)	NR

* Cumulative incidence of first recurrence over the 4 years in patients free of recurrence at increasing timepoints

Abbreviations: BC: bladder cancer; IQR: interquartile range; LVI: lymph vascular invasion; NMIBC: non-muscle invasive bladder cancer; NR: not reported; PUNLMP: papillary urothelial neoplasm of low malignant potential; SD: standard deviation; UCB: Urothelial carcinoma of the urinary bladder; UK: United Kingdom; USA: United States of America; WHO: The World Health Organization.

Table 7: Site of cancer recurrence in patients with BC (n = 2 studies)

Study name	Country	Patient population	N	Follow-up	Site	Outcome subtypes/parameter	n (%) patients	*p*-value
Chaux 2012 (4)	USA	High-grade papillary UC in Urinary bladder and upper urinary tract	Total: 85 Number of patients with distant metastasis: 17	24 months	Visceral metastasis	Bones	2 (11.8%)	NR
						Lungs and bones	1 (5.9%)	
						Liver	1 (5.9%)	
						Lungs	1 (5.9%)	
						Liver and lungs	1 (5.9%)	
					Multiple systemic sites	Multiple systemic sites	11 (64.7%)	
Garg 2021 (22)	USA	NMIBC	2956	29.4 months	Site of recurrence	Muscle invasion (≥cT2)	111 (10.45)	NR

Abbreviations: BC: bladder cancer; NMIBC: non-muscle invasive bladder cancer; UC: urothelial carcinoma; USA: United States of America.

Table 8: Time to cancer recurrence in patients with BC (n = 5 studies)

Study name	Country	Patient population	Type of subgroup	N	Follow-up	Time to recurrence	*p*-value
Parsons 2020 (23)	UK	NMIBC	NR	75	Mean (range): 23.4 (0.6–59.4) months	Mean (Range): 0.80 (0.25-2.67) years [293 (91–973) days]	NR
		Low risk NMIBC		17		Mean (Range): 1.07 (0.44-1.64) [392 (162–600) days]	NR
		Intermediate risk NMIBC		13		Mean (Range): 1.28 (0.31-2.67) years [469 (114–973) days]	NR
		High risk NMIBC		45		Mean (Range): 0.70 (0.25-2.19) [256 (91–798) days]	NR
Ratanapornsompon 2019 (30)	Thailand	NMIBC; T1 High-Grade	NR	70	Median (range): 40.1 (2.0 to 252.8) months	Median (Range): 1.03 (0.14-11.78) years [12.3 (1.7-141.3) months]	NR
Canter 2014 (31)	USA	NMIBC; T1 High-Grade	NR	222	Mean: 50.8; Median (Range): 32.5 (2.2-261.2) months	Mean: 2.4 years; Median: 0.92 years [28.8 months; Median: 12.9 months]	NR
Olsson 2013 (32)	Sweden	Primary T1 UCB	NR	211	Median (Range): 60 (3-192) months	Mean (Range): 0.96 (0.08-7.92) years [11 (1-95) months]	NR
Sultana 2021 (29)	Europe	NMIBC Low risk G1pTa UCB	NR	180	NR	Mean (SD): 2.29 (1.79) years [27.5 (21.5) months]	NR
		NMIBC Low risk G2pTa UCB	NR	243	NR	Mean (SD): 0.19 (0.15) years [2.3 (1.8) months]	NR

Note: The actual estimates provided in the study were provided in square brackets. Data were converted to years to enable comparison with other studies.

Abbreviations: NR: not reported; NMIBC: non-muscle invasive bladder cancer; SD: standard deviation; UCB: urothelial carcinoma of bladder; USA: united state of America.

Table 9: Overall survival outcomes in patients with BC (n = 1 study)

Study name	Country	Patient population	Type of subgroup	N	Timepoint	Follow-up	Results	*p*-value
Ratanapornsompon 2019 (24)	Thailand	NMIBC; T1 High-Grade	Recurrent population	39	NR	Median (range): 40.1 (2.0 to 252.8)	Median: 55.13 (range: 8.66-252) months	0.765
			Non-recurrent population	31	NR		Median: 35.13 (range: 2.03-95.86) months	
			Progression population	10	NR		Median: 18.2 months	0.002 vs non-progressive group

Abbreviations: BC: bladder cancer; NMIBC: non-muscle invasive bladder cancer; NR: not reported.

Gastric cancer

Table 10. Recurrence rates in patients with gastric cancer (n = 1 study)

Study name	Country	Patient population	Type of subgroup	N	Timepoint	Follow-up	n (%) patients with recurrence	*p*-value
Kraja 2021 (33)	Albania	Gastric cancer	NR	180	NR	Median: 36± 13 months	6.7%	NR

Abbreviations: NR: Not reported.

Head and neck cancer

Table 11: Recurrence rates in patients with HNC (n = 2 studies)

Study name	Country	Patient population	Type of subgroup	N	Time point	Follow-up	n (%) patients with recurrence	*p*-value
Brandstorp-Boesen 2016 (34)	Norway	Laryngeal squamous cell carcinoma; stage I-IV without metastasis	Overall	1,615	At 120 months	Median (range): 38.4 (0–339.6) months	368 (22.8)	NR
			Male	1,403			322 (22.9)	
			Female	212			46 (21.7)	
			Smoking: Ever	1434			326 (22.7)	
			Smoking: Never	97			30 (30.9)	
			Smoking: Unknown	84			12 (14.3)	
			Alcohol: Ever	223			61 (27.4)	
			Alcohol: Never	928			213 (22.96)	
			Alcohol: Unknown	464			94 (20.26)	
			Age: 0–59	458			130 (28.38)	
			Age: 60–69	542			131 (24.17)	
			Age: ≥70	615			107 (17.38)	
			Subsite: Glottic	1127			243 (21.56)	
			Subsite:	437			113 (25.86)	

Study name	Country	Patient population	Type of subgroup	N	Time point	Follow-up	n (%) patients with recurrence	*p*-value
			Supraglottic					
			Subsite: Subglottic	51			12 (23.53)	
			T-status: T1	667			90 (13.49)	
			T-status: T2	387			120 (31.01)	
			T-status: T3	231			85 (36.79)	
			T-status: T4	330			73 (22.12)	
			N-status: N0	1343			300 (22.34)	
			N-status: N1	99			23 (23.23)	
			N-status: N2+	173			45 (26.01)	
			M-status: M0	1601			368 (22.98)	
			Stage: Early stage	983			194 (19.74)	
			Stage: Advanced stage	632			174 (27.53)	
			Stage: I	650			87 (13.38)	
			Stage: II	330			107 (32.42)	
			Stage: III	222			76 (34.23)	
			Stage: IV	413			98 (23.73)	
			Overall Cumulative	1615	12 months		Risk: 11.3%	

Study name	Country	Patient population	Type of subgroup	N	Time point	Follow-up	n (%) patients with recurrence	*p*-value
			risk of recurrence					
			Overall Cumulative risk of recurrence	1615	36 months		Risk: 20.5%	
			Overall Cumulative risk of recurrence	1615	60 months		Risk: 22.5%	
			Overall Cumulative risk of recurrence	1615	120 months		Risk: 23.6%	
		Laryngeal squamous cell carcinoma: Stagfe I-IV without metastasis	Overall Cumulative risk of recurrence	1,615	36 months		Risk (95%CI): 20.5 (18.6-22.5)	
			Sex: Male	1,403			Risk (95%CI): 20.5 (18.4-22.6)	
			Sex: Female	212			Risk (95%CI): 20.8 (15.5-26.5)	
			Smoking: ever	1434			Risk (95%CI): 21.1 (19-23.3)	
			Smoking: Never	97			Risk (95%CI): 24.3 (16.2-33.4)	
			Smoking: Unknown	84			Risk (95%CI): 12 (6.2-20)	

Study name	Country	Patient population	Type of subgroup	N	Time point	Follow-up	n (%) patients with recurrence	*p*-value
			Alcohol: Ever	223			Risk (95%CI): 26.3 (20.7-32.2)	
			Alcohol: Never	928			Risk (95%CI): 17.1 (13.8-20.7)	
			Alcohol: Unknown	464			NR	
			Age (years): 0–59	458			Risk (95%CI): 25.2 (21.3-29.3)	
			Age (years): 60–69	542			Risk (95%CI): 22.7 (19.2-26.3)	
			Age (years): ≥70	615			Risk (95%CI): 15.1 (12.3-18.1)	
			Subsite: Glottic	1127			Risk (95%CI): 18.7 (16.5-21.1)	
			Subsite: Supraglottic	437			Risk (95%CI): 25.1 (21-29.3)	
			Subsite: Subglottic	51			Risk (95%CI): 21.7 (11.6-33.8)	
			T-status, T1	667			Risk (95%CI): 11 (8.8-13.6)	
			T-status: T2	387			Risk (95%CI): 27.3 (22.9-31.9)	
			T-status: T3	231			Risk (95%CI): 35.8 (29.6-42.1)	
			T-status: T4	330			Risk (95%CI): 21.1 (16.9-25.7)	
			N-status: N0	1343			Risk (95%CI): 19.7 (17.6-21.9)	
			N-status: N1	99			Risk (95%CI): 23.4 (15.6-32.1)	

Study name	Country	Patient population	Type of subgroup	N	Time point	Follow-up	n (%) patients with recurrence	*p*-value
			N-status: N2+	173			Risk (95%CI): 25.5 (19.2-32.3)	
			M-status: M0	1601			NR	
			Stage: Early stage	983			Risk (95%CI): 16.7 (14.4-19.1)	
			Stage: I	650			Risk (95%CI): 10.9 (8.6-13.4)	
			Stage: II	330			Risk (95%CI): 28.2 (23.4-33.1)	
			Stage: III	222			Risk (95%CI): 33.6 (27.4-39.9)	
			Stage: IV	413			Risk (95%CI): 22.6 (18.7-26.8)	
Park 2017 (35)	Korea	Salivary gland cancer Stage I-IV; without distant metastases	Salivary duct carcinoma	240	36 months	Median (range): 160 months (121.5-282.2)	72.89%	NR
			Adenoid cystic carcinoma				39.6%	
			Salivary duct carcinoma		60 months		78.39%	
			Adenoid cystic carcinoma				52.35%	
			Salivary duct carcinoma		120 months		78.39%	

Study name	Country	Patient population	Type of subgroup	N	Time point	Follow-up	n (%) patients with recurrence	*p*-value
			Adenoid cystic carcinoma				63.49%	
		Salivary gland cance	Stage T1, Total	61	Maximum follow-up 282.2 months	Median (range): 160 months (121.5-282.2)	19 (31.15)	
			Stage T1, Early	61			17 (27.87)	
			Stage T1, Late	61			2 (3.28)	
			Stage T2, Total	70			26 (37.14)	
			Stage T2, Early	70			19 (27.14)	
			Stage T2, Late	70			7 (10)	
			Stage T3, Total	79			54 (68.35)	
			Stage T3, Early	79			49 (62.02)	
			Stage T3, Late	79			5 (6.33)	
			Stage T4, Total	30			25 (83.33)	
			Stage T4, Early	30			23 (76.67)	
			Stage T4, Late	30			2 (6.66)	

Study name	Country	Patient population	Type of subgroup	N	Time point	Follow-up	n (%) patients with recurrence	*p*-value
		Salivary gland cancer	Stage N0, Total	179			72 (40.22)	
			Stage N0, Early	179			58 (32.4)	
			Stage N0, Late	179			14 (7.82)	
			Stage N1, Total	7			4 (57.14)	
			Stage N1, Early	7			3 (42.86)	
			Stage N1, Late	7			1 (14.29)	
			Stage N2, Total	54			48 (88.89)	
			Stage N2, Early	54			47 (87.04)	
			Stage N2, Late	54			1 (1.85)	
		Salivary gland cancer (Sta	Stage I, Total	50			10 (20)	
			Stage I, Early	50			8 (16)	
			Stage I, Late	50			2 (4)	
			Stage II, Total	58			20 (34.48)	
			Stage II, Early	58			13 (22.41)	

Study name	Country	Patient population	Type of subgroup	N	Time point	Follow-up	n (%) patients with recurrence	*p*-value
			Stage II, Late	58			7 (12.07)	
			Stage III, Total	64			36 (56.25)	
			Stage III, Early	64			32 (50)	
			Stage III, Late	64			4 (6.25)	
			Stage IV, Total	68			58 (85.29)	
			Stage IV, Early	68			55 (80.88)	
			Stage IV, Late	68			3 (4.41)	
Wilson 2021 (36)	USA	Oral tongue squamous cell carcinoma	Early stage (pT1N0-pT3N0	96	NR	Median : 60 months	20 (20.8)	NR
Kanatas 2014 (37)	UK	Primary oral squamous cell carcinoma	Clinical stage 0-1	98	NR	60 months	13 (13)	NR
			Clinical stage 2	73			16 (22)	
			Clinical stage 3	28			7 (25)	
Park 2017 **(35)**	Korea	Salivary gland cancer	Stage I-IV; without	240	Overall	Median (range): 160	124 (51.7)	NR
					<24 months		84 (35)	

Study name	Country	Patient population	Type of subgroup	N	Time point	Follow-up	n (%) patients with recurrence	*p*-value
			distant metastases		24 – 60 months	(121.5-282.2) months	24 (10)	
					>60 months		16 (6.7)	
Jung 2014 **(38)**	Korea	Head and neck cancer	Stage: T1	218	NR	Mean (SD): 34.7 (22.8) months	30 (13.8)	NR
			Stage: T2	154			35 (22.7)	
			Stage: T3	73			18 (24.7)	
			Stage: T4	59			17 (28.8)	

Abbreviations: CI: confidence interval; HNC: head and neck cancer; HNSCC: head and neck squamous cell carcinoma; NR: not reported; SD: standard deviation; USA: United States of America; UK: United Kingdom.

Table 12: Site of recurrence in patients with HNC (n = 2 studies)

Study name	Country	Patient population	N	Follow-up	Timepoint	Site	Results n (%)	p-value
Kim 2012 (39)	USA	Recurrent locally advanced head and neck cancer; Locally advanced	324	60 months	NR	Tongue	67 (20.7)	NR
						Gum	4 (1.2)	
						Floor of mouth	10 (3.1)	
						Other and unspecified parts of mouth	45 (13.9)	
						Oropharynx	40 (12.3)	
						Hypopharynx	4 (1.2)	
						Other and ill-defined sites within the lip oral cavity and pharynx	14 (4.3)	
						Larynx	140 (43.2)	
Park 2017 (35)	Korea	Salivary gland cancer with recurrence; Stage: I-IV without distant metastasis	9	Median (range): 160 months (121.5-282.2)	NR	Lung	8 (88.9)	NR
						Cervical spine	1 (11.1)	

Abbreviations: HNC: head and neck cancer; NR: not reported; USA: United States of America.

Table 13: Time to recurrence in patients with HNC (n = 2 studies)

Study name	Country	Patient population	Type of subgroup	N	Follow-up	Results	*p*-value
Kim 2012 (39)	USA	Recurrent locally advanced head and neck cancer	Locally advanced	324	NR	Mean (SD): 18.96 (11.24) months after diagnosis	NR
Park 2017 (35)	Korea	Salivary gland cancer	NR	448	Median (range): 160 months (121.5-282.2)	Median (Range): 92.5 (60.2–138.3) months among patients with late recurrence (> 5 years after initial treatment)	NR

Abbreviations: HNC: head and neck cancer; NR: not reported; USA: United States of America.

Table 14: Type of recurrence in patients with HNC (n = 3 studies)

Study name	Country	Patient population	Type of subgroup	N	Timepoint	Follow-up	Type	Results, n (%)	p-value
Wilson 2021 (36)	USA	Oral tongue SCC	Early stage (pT1N0-pT3N0)	20	NR	Median: 48 months	Local	15 (75%)	NR
							Regional	12 (60%)	
							Distant	6 (30%)	
Park 2017 (35)	Korea	Salivary gland cancer with recurrence	Stage I-IV without distant metastasis	124	NR	Median (range): 160 (121.5-282.2) months	Overall: Local	48 (38.7)	NR
							Overall: Regional	20 (16.1)	
							Distant	84 (67.7)	
				16	NR		Late recurrence: Local	7 (43.8)	
							Late recurrence: Regional	5 (31.3)	

Study name	Country	Patient population	Type of subgroup	N	Timepoint	Follow-up	Type	Results, n (%)	p-value
							Late recurrence: Distant	9 (56.3)	
				108	5 years		Early recurrence: Local	41 (38.0)	0.657vs. late recurrence
							Early recurrence: Regional	15 (13.9)	0.136 vs. late recurrence
							Early recurrence: Distant	75 (69.4)	0.292 vs. late recurrence
Brandstorp-Boesen 2016 (34)	Norway	Laryngeal SCC; Overall	(T1-T4, N0-N2+, Stage I-IV Non - metastatic)	368	10 years	Median (range): 3.2 years (0–28.3)	Overall: Local recurrence	261 (70.9%)	NR
							Regional lymph node metastasis	50 (13.6%)	
							Loco-regional	13 (3.5%)	
							Local stoma	13 (3.5%)	
							Distant pulmonary	22 (6%)	
							Loco-regional+ distant	9 (2.5%)	
				243			Glottic subsite: Local recurrence	190 (78.2%)	

Study name	Country	Patient population	Type of subgroup	N	Timepoint	Follow-up	Type	Results, n (%)	p-value
							Regional lymph node metastasis	25 (10.3%)	
							Loco-regional	7 (2.9%)	
							Local stoma	9 (3.7%)	
							Distant pulmonary	8 (3.3%)	
							Loco-regional+ distant	4 (1.6%)	
				113			Supraglottic subsite: Local recurrence	64 (56.7%)	
							Regional lymph node metastasis	24 (21.2%)	
							Loco-regional	6 (5.3%)	
							Local stoma	3 (2.7%)	
							Distant pulmonary	12 (10.6%)	
							Loco-regional+ distant	4 (3.5%)	
				12			Subglottic subsite: Local recurrence	7 (58.4%)	
							Regional lymph node metastasis	1 (8.3%)	
							Loco-regional	0 (0%)	
							Local stoma	1 (8.3%)	

Study name	Country	Patient population	Type of subgroup	N	Timepoint	Follow-up	Type	Results, n (%)	p-value
							Distant pulmonary	2 (16.7%)	
							Loco-regional+ distant	1 (8.3%)	

Abbreviations: HNC: head and neck cancer; NR: not reported; USA: United States of America, SCC: Squamous cell carcinoma.

Table 15: Overall survival in patients with HNC (n = 1 study)

Study name	Country	Patient population	Type of subgroup	N	Timepoint	Follow-up	Results	*p*-value
Park 2017 (35)	Korea	Salivary gland cancer with recurrence	NR	140	NR	Median (range): 160 months (121.5-282.2)	Patients with early recurrence (recurrence ≤5 years after initial treatment): median (range): 19.7 (0.2–172.2) months	NR
							Patients with late recurrence (recurrence >5 years after initial treatment): median OS (range): 79.7 (0.2–163.4) months	
						NR	Salivary duct carcinoma patients with recurrence: median OS (range): 8.4 (1.7–102.9)	
							Adenoid cystic carcinoma patients with recurrence: median OS (range): 46.9 (0.2–172.2) months	

Abbreviations: HNC: head and neck cancer; NR: not reported; OS: overall survival.

Melanoma

Table 16: Recurrence rates in patients with melanoma (n = 21 studies)

Study name	Country	Patient population	Type of subgroup	N	Timepoint	Follow-up	n (%) patients with recurrence	*p*-value
Bleicher 2020 (40)	USA	Cutaneous melanoma	Stage IIA	280	5 years	Median (IQR): 4.9 (2.5-7.9) years	38 (13.6)	NR
			Stage IIB	220			62 (28.2)	
			Stage IIC	80			36 (45)	
			Stage IIA	280	3 years		12%	
			Stage IIA	280	5 years		21%	
			Stage IIB	220	3 years		19%	
			Stage IIB	220	5 years		35%	
			Stage IIC	80	3 years		37%	
			Stage IIC	80	5 years		48%	
Chakera 2019 (41)	Australia	Subungual melanoma	Stage IA-IIIB/C	44	5 year	Median: 105 months	48%	NR
				44	10 years		54%	
				103	NR		44 (42.7%)	
Quhill 2021 (42)	Ireland	Uveal melanoma	Non-Metastatic	182	NR	Median: 79.5 months	7.6%	NR
Tarhini 2018 (43)	NR	Non-Metastatic melanoma	Non-Metastatic	6400	NR	Median: 23.1 months	1191 (18.6%)	NR
Chen 2021 (44)	Taiwan	Conjunctival melanoma	Stages T1-T3	20	181 months	Mean (SD): 68.7 (55.8)	18 (90)	NR
					2 years		45%	

Study name	Country	Patient population	Type of subgroup	N	Timepoint	Follow-up	n (%) patients with recurrence	*p*-value
					3 years		50%	
					5 years		65%	
					10 years		81%	
		Conjunctival melanoma with local recurrence; Stage T1-T3	Sex: Male	12	181 months		3 (25)	
			Sex: Female	8			3 (38)	
			Subgroup: T3	9			3 (33)	
			Subgroup: T2	7			2 (289)	
			Subgroup: T1	4			1 (25)	
			Subgroup: Tumor thickness >2mm	16			6 (40)	
			Subgroup: Tumor thickness <2mm	4			0 (0)	
		Conjunctival melanoma with distant recurrence	Subgroup: T3	9			7 (77.8)	
			Subgroup: T2	7			2 (28.6)	
			Subgroup: T1	4			3 (75)	

Study name	Country	Patient population	Type of subgroup	N	Timepoint	Follow-up	n (%) patients with recurrence	*p*-value
Jang 2020 (13)	USA	Melanoma	Stage IIB	NR	1-year post index (resection)	NR	12.5%	NR
			Stage IIC				21.8%	
			Stage IIIA				28.2%	
			Stage IIB	NR	2-year post index (resection)		29.3%	
			Stage IIC				43.5%	
			Stage IIIA				46.5%	
Kolla 2021 (7)	USA	Acral lentiginous melanoma	Stage IIB	47	Within 5 years	Median (range): 32 (0–259) months	94.7%	NR
			Stage IIC	26			100%	
			Stage IIIA	14			83.3%	
			Stage IIIB	17			88.9%	
			Stage IIIC	61			97.1%	
			Stage 0	NR	3 years		0%	
			Stage 0	NR	5 years		0%	
			Stage I	NR	3 years		5.56%	
			Stage I	NR	5 years		9.59%	
			Stage II	NR	3 years		32.31%	
			Stage II	NR	5 years		49.78%	
			Stage III	NR	3 years		55.53%	
			Stage III	NR	5 years		61.77%	
			Stage IIA	NR	3 years		12.27%	
			Stage IIA	NR	5 years		32.06%	
			Stage IIB	NR	3 years		36.79%	

Study name	Country	Patient population	Type of subgroup	N	Timepoint	Follow-up	n (%) patients with recurrence	*p*-value
			Stage IIB	NR	5 years		54.92%	
			Stage IIC	NR	3 years		48.79%	
			Stage IIC	NR	5 years		61.69%	
			Stage IIIA	NR	3 years		44.32%	
			Stage IIIA	NR	5 years		44.32%	
			Stage IIIB	NR	3 years		49.94%	
			Stage IIIB	NR	5 years		49.94%	
			Stage IIIC	NR	3 years		54.03%	
			Stage IIIC	NR	5 years		66.67%	
			Acral Lentiginous Melanoma	384	5 years		NR (37.2)	
			Stage IA	89	5 years		NR (9.3)	
			Stage IB	58	5 years		NR (10.3)	
			Stage IIA	41	5 years		NR (32.2)	
			Stage IIB	47	5 years		NR (55)	
			Stage IIC - Total	26	5 years		NR (61.7)	
			Stage IIIA - Total	14	5 years		NR (44.3)	
			Stage IIIB - Total	17	5 years		NR (49.8)	

Study name	Country	Patient population	Type of subgroup	N	Timepoint	Follow-up	n (%) patients with recurrence	*p*-value
			Stage IIIC - Total	61	5 years		NR (66.6)	
			Stage IIID - Total	8	5 years		NR (100)	
			Stage 0	NR	5 years		NR (0)	
			Stage I	NR	5 years		NR (9.7)	
			Stage II	NR	5 years		NR (49.9)	
			Stage III	NR	5 years		NR (61.8)	
Leeneman 2019 (45)	Netherlands	Localized and regionally advanced cutaneous melanoma	Stage IB	755	5 years	Median: 5.4 years	57 (8%)	NR
			Stage II	471			137 (29%)	
			Stage III	171			81 (47%)	
			Stage IB	755	3 years		4.2%	
			Stage IB	755	5 years		6.4%	
			Stage II	471	3 years		24.9%	
			Stage II	471	5 years		30.3%	
			Stage III	171	3 years		39.3%	
			Stage III	171	5 years		45.9%	
Loidi 2021 (8)	Spain	Cutaneous melanoma	stage I-III	308	5 years	Mean: 68.63 months	78 (25.3)	NR
					10 years		92 (29.9)	
					NR		94 (30.52)	
Ogata 2021 (46)	Japan	Sentinel node-positive melanoma	Pre-June 2017- Stage III	NR	3 years	Median	61.1%	NR
					5 years		61.23%	

Study name	Country	Patient population	Type of subgroup	N	Timepoint	Follow-up	n (%) patients with recurrence	*p*-value
			Post-July 2017- Stage III		3 years	Pre-June 2017: 1107 days<br>Post-July 2017: 587.5 days	59.4%	
		Overall	Overalll (stage II)	119	NR		52 (43.7%)	
		Completion lymph node dissection (CLND) group		94	NR		45 (47.9%)	
		Observation		25	NR		7 (28%)	
		Cohort 1: Sentinel node-positive melanoma (Stage III), pre-June 2017 group		63	NR	Median<br>Pre-June 2017: 1107 days	36 (57.14%)	
		Cohort 2: Sentinel node-positive melanoma (Stage III), post-July 2017 group		56	NR	Post-July 2017: 587.5 days	16 (28.57%)	

Study name	Country	Patient population	Type of subgroup	N	Timepoint	Follow-up	n (%) patients with recurrence	*p*-value
Osella-Abate 2015 (12)	Italy	Melanoma	Stage I/II	1,372	30.1 years (maximum follow up); late recurrence	Median (range): 5.4 (0.1-30.1) years	77 (5.6%)	NR
Feigelson 2019 (10)	USA	Melanoma	Stage I-III	1,877	NR	Median: 4.1 years	8.8%	NR
Varey 2017 (47)	Australia	Neurotropic cutaneous melanoma	Stage I-III	1389	NR	Median time: Case (neurotropic melanomas): 3.5 years; Control (non-neurotropic melanomas): 3.6 years	370 (26.6%)	
		Non-neurotropic					157 (22%)	
		Neurotropic					213 (32%)	
		Neurotropic subtype- non-desmoplastic					73 (38%)	
		Neurotropic subtype-Desmoplastic					140 (29%)	
Rasmussen 2019 (1)	Denmark	Non-metastatic malignant melanoma	Sex: Women	7,328	3 years	NR	7.9%	NR
			Sex: Women	7,328	5 years		10%	
Rockberg 2016 (48)	Sweden		Stage I	2,523	Before 180 days		127 (5.0)	NR

Study name	Country	Patient population	Type of subgroup	N	Timepoint	Follow-up	n (%) patients with recurrence	*p*-value
		Cutaneous malignant melanoma			After 180 days	Median (range): 4.43 (0-9.8) years	242 (9.6)	
			Stage II	746	Before 180 days		108 (14.5)	
					After 180 days		190 (25.5)	
			Stage III	239	Before 180 days		144 (60.3)	
					After 180 days		53 (22.2)	
			Stage I	2,523	3 years		10.55%	
					5 years		13.13%	
			Stage II	746	3 years		34.04%	
					5 years		37.93%	
			Stage III	239	3 years		80.16%	
					5 years		82.75	
			Overall - stage I-III	3508	9.8 years	4.43 years (0-9.8)	846 (24.1)	
			Stage I	2523			369 (14.6)	
			Stage II	746			298 (39.9)	
			Stage III	239			197 (82.4)	

Study name	Country	Patient population	Type of subgroup	N	Timepoint	Follow-up	n (%) patients with recurrence	*p*-value
Sarac 2020 (9)	Germany	Superficially spreading melanoma, nodular melanoma, lentigo malignant melanoma, acrolentiginous melanoma, and others who had metastasized locoregionally or distantly	Stage IA-IIC	1,537	10 years (early recurrence)	3 months (minimum)	1,438 (10.8%)	NR
					More than 10 years (late recurrence)		99 (0.73%)	
Tas 2019 (49)	Turkey	Local and regional cutaneous melanoma	Overall Stage I-III	1087	271.8 months (maximum follow up)	Median (range): 73.6 (36.2–271.8) months	365 (33.6)	NR
			Stage I-II	457			196 (18)	NR
			Stage III	250			169 (15.5)	NR
		Locoregional alone	Stage I-II	196			111 (56.6)	NR
		Locoregional and distant	Stage I-II	196			43 (21.9)	NR
		Distant alone	Stage I-II	196			42 (21.4)	NR

Study name	Country	Patient population	Type of subgroup	N	Timepoint	Follow-up	n (%) patients with recurrence	*p*-value
		Distant metastases-pulmonary (lung and pleura)	Stage I-II	196			62 (31.8)	NR
		Distant metastases-Bone	Stage I-II	196			29 (14.8)	NR
		Distant metastases-Liver	Stage I-II	196			29 (14.8)	NR
		Distant metastases-brain	Stage I-II	196			18 (9.2)	NR
		Locoregional alone	Stage III	169			71 (42)	NR
		Locoregional and distant	Stage III	169			43 (25.4)	NR
		Distant alone	Stage III	169			55 (32.5)	NR
		Distant metastases-pulmonary (lung and pleura)	Stage III	169			55 (32.7)	NR

Study name	Country	Patient population	Type of subgroup	N	Timepoint	Follow-up	n (%) patients with recurrence	*p*-value
		Distant metastases-Bone	Stage III	169			28 (16.7)	NR
		Distant metastases-Liver	Stage III	169			27 (16.1)	NR
		Distant metastases-brain	Stage III	169			23 (13.6)	NR
		Age < 50	Stage I-II	218			92 (42.2)	NR
		Age ≥ 50	Stage I-II	239			104 (43.5)	NR
		Female	Stage I-II	230			71 (30.9)	NR
		Male	Stage I-II	227			125 (55.1)	NR
		Site of lesion: Axial	Stage I-II	265			121 (45.7)	NR
		Site of lesion: Extremity	Stage I-II	192			75 (39.1)	NR
		Histopathology: Non-nodular	Stage I-II	262			78 (29.8)	NR
		Histopathology: Nodular	Stage I-II	90			49 (54.4)	NR
		Clark level: I–III	Stage I-II	157			39 (24.8)	NR
		Clark level: IV–V	Stage I-II	232			102 (44)	NR

Study name	Country	Patient population	Type of subgroup	N	Timepoint	Follow-up	n (%) patients with recurrence	*p*-value
		Thickness equals depth, mm: ≤ 1	Stage I-II	81			15 (18.5)	NR
		Thickness equals depth, mm: 1.01–2	Stage I-II	99			26 (26.3)	NR
		Thickness equals depth, mm: 2.01–4	Stage I-II	122			53 (43.4)	NR
		Thickness equals depth, mm: > 4	Stage I-II	80			49 (61.3)	NR
		TIL: No	Stage I-II	143			54 (37.8)	NR
		TIL: Yes	Stage I-II	180			55 (30.6)	NR
		Mitotic rate, /mm2: 0–1	Stage I-II	118			19 (16.1)	NR
		Mitotic rate, /mm2: 1.1–4.9	Stage I-II	109			43 (39.4)	NR
		Mitotic rate, /mm2: 5–9.9	Stage I-II	57			26 (45.6)	NR
		Mitotic rate, /mm2: ≥ 10	Stage I-II	34			20 (58.8)	NR
		Ulceration: No	Stage I-II	182			37 (20.3)	NR
		Ulceration: Yes	Stage I-II	161			85 (52.8)	NR

Study name	Country	Patient population	Type of subgroup	N	Timepoint	Follow-up	n (%) patients with recurrence	*p*-value
		Regression: No	Stage I-II	210			71 (33.8)	NR
		Regression: Yes	Stage I-II	69			16 (23.2)	NR
		LVI: No	Stage I-II	291			92 (31.6)	NR
		LVI: Yes	Stage I-II	21			11 (52.4)	NR
		BRAFV600E mutation: Negative	Stage I-II	18			18 (100)	NR
		BRAFV600E mutation: Positive	Stage I-II	14			14 (100)	NR
		Age < 50	Stage III	119			75 (63)	NR
		Age ≥ 50	Stage III	131			94 (71.8)	NR
		Female	Stage III	102			66 (64.7)	NR
		Male	Stage III	148			103 (69.6)	NR
		Site of lesion: Axial	Stage III	125			83 (66.4)	NR
		Site of lesion: Extremity	Stage III	112			78 (69.6)	NR
		Histopathology: Non-nodular	Stage III	112			66 (58.9)	NR
		Histopathology: Nodular	Stage III	66			48 (72.7)	NR

Study name	Country	Patient population	Type of subgroup	N	Timepoint	Follow-up	n (%) patients with recurrence	*p*-value
		Clark level: I–III	Stage III	25			13 (52)	NR
		Clark level: IV–V	Stage III	167			113 (67.7)	NR
		Thickness equals depth, mm: ≤ 1	Stage III	4			1 (25)	NR
		Thickness equals depth, mm: 1.01–2	Stage III	29			17 (58.6)	NR
		Thickness equals depth, mm: 2.01–4	Stage III	62			37 (59.7)	NR
		Thickness equals depth, mm: > 4	Stage III	93			69 (74.2)	NR
		TIL: No	Stage III	109			71 (65.1)	NR
		TIL: Yes	Stage III	56			39 (69.6)	NR
		Mitotic rate, /mm2: 0–1	Stage III	20			10 (50)	NR
		Mitotic rate, /mm2: 1.1–4.9	Stage III	74			47 (63.5)	NR
		Mitotic rate, /mm2: 5–9.9	Stage III	45			33 (73.3)	NR

Study name	Country	Patient population	Type of subgroup	N	Timepoint	Follow-up	n (%) patients with recurrence	*p*-value
		Mitotic rate, /mm2: ≥ 10	Stage III	32			24 (75)	NR
		Ulceration: No	Stage III	57			31 (54.4)	NR
		Ulceration: Yes	Stage III	118			85 (72)	NR
		Regression: No	Stage III	117			76 (65)	NR
		Regression: Yes	Stage III	36			22 (61.1)	NR
		LVI: No	Stage III	121			79 (65.3)	NR
		LVI: Yes	Stage III	29			20 (69)	NR
		BRAFV600E mutation: Negative	Stage III	28			28 (100)	NR
		BRAFV600E mutation: Positive	Stage III	20			20 (100)	NR
Von Schuckman 2019 (11)	Australia	Newly diagnosed Melanoma patients; Stage IB-IIC	AJCC 8 stages IB-IIC	700	2 years	NR	94 (13.4)	
			AJCC 7 stages IB-IIC	789			99 (12.5)	
			Male- AJCC 8	410			65 (15.8)	
			Female- AJCC 8	290			29 (10)	

Study name	Country	Patient population	Type of subgroup	N	Timepoint	Follow-up	n (%) patients with recurrence	*p*-value
			Age <55 years	189			19 (10.1)	
			Age 55-70 years	309			39 (12.6)	
			Age>70 years	202			36 (17.8)	
			No previous melanoma	563			75 (13.3)	
			With previous melanoma	137			19 (13.9)	
			Sentinel lymph node biopsy - no	442			65 (14.7)	
			Sentinel lymph node biopsy (SLNB)- yes	258			30 (11.6)	
			Sentinel lymph node biopsy (SLNB) positive- No	220			12 (5.4)	

Study name	Country	Patient population	Type of subgroup	N	Timepoint	Follow-up	n (%) patients with recurrence	*p*-value
			Sentinel lymph node biopsy (SLNB) positive- yes	38			18 (47.4)	
			AJCC 8: body site trunk	247			30 (12.14)	
			AJCC 8: body site head/neck	154			38 (24.6)	
			AJCC 8: body site upper limbs	143			7 (4.9)	
			AJCC 8: body site lower limbs	156			19 (12.17)	
			Thickness, mm: ≤1	121			6 (4.95)	
			Thickness, mm: >1-2	312			31 (9.93)	
			Thickness, mm: >2-4	178			33 (18.5)	
			Thickness, mm: >4	89			24 (26.9)	

Study name	Country	Patient population	Type of subgroup	N	Timepoint	Follow-up	n (%) patients with recurrence	*p*-value
			Ulceration: No	504			50 (9.9)	
			Ulceration: Yes	196			44 (22.4)	
			Mitotic rated: <1	109			10 (9.17)	
			Mitotic rated: 44564	300			20 (6.66)	
			Mitotic rated: >3	273			61 (22.3)	
			Regression: No	457			68 (14.9)	
			Regression: Yes	243			26 (10.7)	
			Subtype: SSM	278			25 (8.99)	
			Subtype: Nodular	172			27 (15.7)	
			Subtype: Other	118			15 (12.71)	
			Subtype: Not classified	132			27 (20.5)	
			T stage: T1b:	121			6 (5)	
			T stage: T2a	242			17 (7)	

Study name	Country	Patient population	Type of subgroup	N	Timepoint	Follow-up	n (%) patients with recurrence	*p*-value
			T stage: T2b	70			14 (20)	
			T stage: T3a	121			21 (17.4)	
			T stage: T3b	57			12 (21.1)	
			T stage: T4a	39			8 (20)	
			T stage: T4b	50			16 (32.7)	
			Clinical stage: IB	352			20 (5.7)	
			Clinical stage: IIA	176			29 (16.5)	
			Clinical stage: IIB	93			18 (19.4)	
			Clinical stage: IIC	41			10 (24.4)	
			T stage: T1b:	195			10 (5.1)	
			T stage: T2a	253			17 (6.7)	
			T stage: T2b	71			14 (17.7)	
			T stage: T3a	122			22 (18.0)	
			T stage: T3b	57			12 (21.1)	
			T stage: T4a	40			8 (20.0)	
			T stage: T4b	50			16 (32.7)	
			Clinical stage: IB	437			24 (5.5)	

Study name	Country	Patient population	Type of subgroup	N	Timepoint	Follow-up	n (%) patients with recurrence	*p*-value
			Clinical stage: IIA	178			30 (16.9)	
			Clinical stage: IIB	93			18 (19.1)	
			Clinical stage: IIC	42			10 (24.4)	

Abbreviations: AJCC: American Joint Committee on Cancer; LVI: lymphovascular invasion; NR: not reported, IQR: inter-quartile range, USA: United States of America.

Table 17. Site of recurrences in patients with melanoma (n = 6 studies)

Study name	Country	Patient population	Type of subgroup	N	Time point	Follow-up	Outcome	Site	Results	*p*-value
Bleicher 2020 (40)	USA	Cutaneous melanoma with distant recurrence	Stage II	80	NR	Median (IQR): 4.9 (2.5-7.9) years	Site of recurrence (metastases)	Pulmonary metastases	n (%): 29 (36.3)	NR
								Brain metastases	n (%): 17 (21.3)	
								Intraabdominal metastases	n (%): 9 (11.3)	
								Bone metastases	n (%): 6 (7.5)	
								Multiple sites of primary recurrence	n (%): 7 (8.8)	
								Distant metastasis at another location.	n (%): 12 (14.8)	
Chen 2021 (44)	Taiwan	Stage T1-T3 melanoma with distant metastasis	Stage T1-T3	12	NR	Mean: 68.7± 55.8 months	Site of distant metastasis	Lung metastasis	4 (33.3%)	
								Central nervous system	3 (25%)	

Study name	Country	Patient population	Type of subgroup	N	Time point	Follow-up	Outcome	Site	Results	*p*-value
						(12 to 181 months)		Regional lymph node	2 (16.7%)	
								Liver	1 (8.3%)	
								Bone	1 (8.3%)	
Osella-Abate 2015 (12)	Italy	Melanoma (stage I/II) with regional recurrence	Stage I/II	52	NR	Median (range): 5.4 (0.1-30.1) years	Site of recurrences	In transit metastases	n (%): 19 (36.5)	
								Lymph nodes	n (%): 31 (59.6)	
								Skin + lymph nodes	n (%): 2 (3.8)	
		Melanoma with distant recurrence		25				Visceral involvement	n (%): 21 (84)	
								Cutaneous metastases	n (%): 3 (12)	
								Lymph node metastases	n (%): 1 (4)	
								Brain	n (%): 6 (24)	
								Lung	n (%): 6 (24)	

Study name	Country	Patient population	Type of subgroup	N	Time point	Follow-up	Outcome	Site	Results	*p*-value
								Liver	n (%): 1 (4)	
								Skin	n (%): 3 (12)	
								Multiple organs	n (%): 8 (32)	
Von Schuckman 2019 (11)	Australia	Newly diagnosed localized melanoma at a high risk of metastasis, with recurrence according to AJCC 8 or AJCC 7 (stage IB-IIC)	AJCC 8 or AJCC 7 (stage IB-IIC)	94	2 years	NR	Affected sites overall	Regional lymph nodes	n (%): 95 (38.0%)	
								Lung	n (%): 35 (14.0%)	
								Local sites	n (%):23 (9.2%)	
								Distant lymph nodes	n (%): 23 (9.2%)	
								Central nervous system	n (%): 23 (9.2%)	
					NR		Site of first recurrence	Lungs	n (%): 9 (47.4)	
								CNS	n (%): 6 (31.6)	

Study name	Country	Patient population	Type of subgroup	N	Time point	Follow-up	Outcome	Site	Results	*p*-value
								Multiple organs	n (%): 3 (15.8%)	
							Second recurrence	Bone	n (%): 10 (12.2)	
								Abdominal organs	n (%): 18 (24.4)	
								Thyroid	n (%): 2 (2.7)	
								Parotoid glands	n (%): 2 (2.7)	
Tas 2017 (50)	Turkey	Early-stage cutaneous melanoma with recurrence (stage I-III)	Early-stage I-III	332<br>Number of events: 294	NR	NR	Site of recurrence, total metastasis	Lung metastasis	n (%): 102 (30.7)	NR
								Bone metastasis	n (%): 52 (15.7)	
								Liver metastasis	n (%): 46 (13.9)	
								Brain	n (%): 36 (10.8)	
								Pleural effusion	n (%): 19 (5.7)	

Study name	Country	Patient population	Type of subgroup	N	Time point	Follow-up	Outcome	Site	Results	*p*-value
								Adrenal gland	n (%): 13 (3.9)	
								Spleen	n (%): 11 (3.3)	
								Peritoneum	n (%): 8 (2.4)	
								Others	n (%): 7 (2.1)	
		Lung	NR	332				Single organ metastasis	n (%): 15 (4.5)	
								Associated with another organ metastasis	n (%): 87 (26.2)	
								Total	n (%): 102 (30.7)	
		Bone						Single organ metastasis	n (%): 3 (0.9)	
								Associated with another organ metastasis	n (%): 49 (14.8)	

Study name	Country	Patient population	Type of subgroup	N	Time point	Follow-up	Outcome	Site	Results	*p*-value
								Total	n (%): 52 (15.7)	
		Liver						Single organ metastasis	n (%): 4 (1.2)	
								Associated with another organ metastasis	n (%): 42 (12.7)	
								Total	n (%): 46 (13.9)	
		Brain						Single organ metastasis	n (%): 14 (4.2)	
								Associated with another organ metastasis	n (%): 22 (6.6)	
								Total	n (%): 36 (10.8)	
		Pleural effusion		NR				Single organ metastasis	n (%): 2 (0.6)	

Study name	Country	Patient population	Type of subgroup	N	Time point	Follow-up	Outcome	Site	Results	*p*-value
								Associated with another organ metastasis	n (%): 17 (5.1)	
								Total	n (%): 19 (5.7)	
		Adrenal gland						Single organ metastasis	n (%): 0 (0)	
								Associated with another organ metastasis	n (%): 13 (3.9)	
								Total	n (%): 13 (3.9)	
		Spleen						Single organ metastasis	n (%): 0 (0)	
								Associated with another organ metastasis	n (%): 11 (3.3)	
								Total	n (%): 11 (3.3)	

Study name	Country	Patient population	Type of subgroup	N	Time point	Follow-up	Outcome	Site	Results	*p*-value
		Peritoneum						Single organ metastasis	n (%): 0 (0)	
								Associated with another organ metastasis	n (%): 8 (2.4)	
								Total	n (%): 8 (2.4)	
		Other sites						Single organ metastasis	n (%): 2 (0.6)	
								Associated with another organ metastasis	n (%): 5 (1.5)	
								Total	n (%): 7 (2.1)	
Tas 2019 (49)	Turkey	Patients with Stage I-II having distant recurrence	Stage I-II	42 (Number of events: 138)	NR	Median: 73.6 months (range 36.2–271.8 months)	Site of distant metastasis	Pulmonary	n (%): 62 (44.9)	NR
								Bone	n (%): 29 (21)	
								Liver	n (%): 29 (21)	

Study name	Country	Patient population	Type of subgroup	N	Time point	Follow-up	Outcome	Site	Results	*p*-value
								Brain	n (%): 18 (13)	
		Patients with Stage III having distant recurrence	Stage III	55 (Number of events: 133)				Pulmonary	n (%): 55 (41.4)	
								Bone	n (%): 28 (21.1)	
								Liver	n (%): 27 (20.3)	
								Brain	n (%): 23 (17.3)	

Abbreviations: AJCC: American Joint Committee on Cancer; CNS: central nervous system; IQR: interquartile range; NR: not reported.

Table 18. Time to recurrence in patients with melanoma (n = 7 studies)

Study name	Country	Patient population	Type of subgroup	N	Follow-up	Results	*p*-value
Ertekin 2021 (51)	Spain	Invasive cutaneous melanoma	Stage IA–IIID	784	NR	Time in years to first recurrence after the diagnosis of primary melanoma: 1.77 years (IQR 0.88–3.46)	<0.001
			Stage I	171		Median time to recurrence: 3.32 years (IQR 1.72–6.14)	<0.001
			Stage II	260		Median time to recurrence: 1.85 years (IQR 0.99–3.38)	<0.001

Study name	Country	Patient population	Type of subgroup	N	Follow-up	Results	*p*-value
			Stage III	353		Median time to recurrence: 1.19 years (IQR 0.70–2.42)	<0.001
Leeneman 2019 (45)	Netherlands	Cutaneous melanoma	Stage IB	755	5.4 years	Median time to first recurrence: 2.8 years	NR
			Stage II	471		Median time to first recurrence: 1.5 years	
			Stage III	171		Median time to first recurrence: 1.0 years	
		Cutaneous melanoma; Intralymphatic metastasis	Stage IB	10		Median time to recurrence: 2.1 years	
		Cutaneous melanoma; Regional lymph node metastases		24		Median time to recurrence:3.0 years	
		Cutaneous melanoma; Distant metastases		20		Median time to recurrence: 3.1 years	
		Cutaneous melanoma; Intralymphatic metastasis	Stage II	25		Median time to recurrence: 2.1 years	
		Cutaneous melanoma; Regional lymph node metastases		51		Median time to recurrence: 0.80 years	
		Cutaneous melanoma; Distant metastases		57		Median time to recurrence: 2.2 years	
		Cutaneous melanoma; Intralymphatic metastasis	Stage III	15		Median time to recurrence: 1.3 years	

Study name	Country	Patient population	Type of subgroup	N	Follow-up	Results	*p*-value
		Cutaneous melanoma; Regional lymph node metastases		25		Median time to recurrence: 0.5 years	
		Cutaneous melanoma; Distant metastases		39		Median time to recurrence: 1.1 years	
Tas 2019 (49)	Turkey	Cutaneous melanoma	Stage I-III	365	Median: 73.6 months (range 36.2–271.8 months)	Median time to relapse: 1.43 years 17.1 months (range 0.8–240.5)	NR
			Stage I-II	196		Median times to relapse: 1.86 years 22.3 months (range 0.8–240.5)	NR
			Stage III	169		Median times to relapse: 1.12 years 13.4 months (range 1.0–124.6)	NR
Tas 2017 (50)	Turkey	Cutaneous melanoma	Non-metastatic	332	NR	Median time to recurrence: 1.37 years; 16.5 months (range 1-24.5)	NR
				168		Median time to recurrence (locoregional): 1.36 years; 16.45 months (range 1-24.5)	NR
				86		Median time to recurrence (distant metastases): 1.35 years; 16.25 months (range 3.6-172.0)	NR
						Median time to recurrence (mixed locoregional and distant): 1.38 years; 16.5 months (range 1-87.8)	NR

Study name	Country	Patient population	Type of subgroup	N	Follow-up	Results	*p*-value
Loidi 2021 (8)	Spain	Cutaneous melanoma	NR	308	Mean: 68.63 months	Mean time to recurrence: 2.7 years (32.97 ± 30.99 months)	NR
		Recurrent cutaneous melanoma		94		Median time to recurrence (locoregional): 1.67 years	NR
						Median time to recurrence (Haemato-visceral): 1.67 years	NR
Von Schuckman 2019 (11)	Australia	Cutaneous melanoma	Stage IB-IIC	700	NR	Median time to first recurrence: 0.77 years	NR
		Cutaneous melanoma		700		Median time to second recurrence: 1.09 years	NR
Kolla 2021 (7)	USA	Primary acral lentiginous melanoma; Transit/satellite recurrence	Stage IIB/IIC	40	32 months (range 0–259 months)	Median time to recurrence: 1.79 years	NR
		Primary acral lentiginous melanoma; Regional lymph nodes recurrence		29		Median time to recurrence: 1.92 years	NR
		Primary acral lentiginous melanoma; Nodal and distant recurrence		9		Median time to recurrence: 1.58 years	NR
		Primary acral lentiginous melanoma; Distant metastases		25		Median time to recurrence: 2.0 years	NR

Abbreviations: IQR: inter-quartile range; NR: not reported; USA: United States of America.

Table 19. Type of recurrences in patients with melanoma (n=16 studies)

Study name	Country	Patient population	Type of subgroup	N	Timepoint	Type	Definition	Results	*p*-value
Kolla 2021 (7)	USA	Acral lentiginous melanoma	Overall stage IA to IIIA	103	NR	Regional nodes	NR	n (%): 29 (28.2%)	NR
						Distant metastasis		n (%): 25 (24.2%)	
						In transit		n (%): 40 (38.8%)	
						Nodal + distant		n (%): 9 (8.7%)	
			Stage IA	89	Median (range): 32 (0–259) months	In transit/satellite		n (%): 4 (4.5)	
						Nodal		n (%): 2 (2.2)	
						Distant		n (%): 0	
						Nodal + distant		n (%): 0	
						Total		n (%): 6 (6.7)	
			stage IB	58		In transit/satellite		n (%): 0	

Study name	Country	Patient population	Type of subgroup	N	Timepoint	Type	Definition	Results	*p*-value
						Nodal		n (%): 1 (1.7)	
						Distant		n (%): 2 (3.4)	
						Nodal + distant		n (%): 0	
						Total		n (%): 3 (5.2)	
			stage IIA	41		In transit/satellite		n (%): 4 (9.8)	
						Nodal		n (%): 1 (2.4)	
						Distant		n (%): 0	
						Nodal + distant		n (%): 2 (4.9)	
						Total		n (%): 7 (17.1)	
			stage IIB	47		In transit/satellite		n (%): 4 (8.5)	

Study name	Country	Patient population	Type of subgroup	N	Timepoint	Type	Definition	Results	*p*-value
						Nodal		n (%): 10 (21.3)	
						Distant		n (%): 2 (4.3)	
						Nodal + distant		n (%): 2 (4.3)	
						Total		n (%): 19 (40.4)	
			stage IIC	26		In transit/satellite		n (%): 5 (19.2)	
						Nodal		n (%): 3 (11.5)	
						Distant		n (%): 3 (11.5)	
						Nodal + distant		n (%): 0	
						Total		n (%): 11 (42.3)	
			stage IIIA	14		In transit/satellite		n (%): 2 (14.3)	

Study name	Country	Patient population	Type of subgroup	N	Timepoint	Type	Definition	Results	*p*-value
						Nodal		n (%): 1 (7.1)	
						Distant		n (%): 3 (21.4)	
						Nodal + distant		n (%): 0	
						Total		n (%): 6 (42.9)	
			stage IIIB	17		In transit/satellite		n (%): 7 (41.2)	
						Nodal		n (%): 1 (5.9)	
						Distant		n (%): 0	
						Nodal + distant		n (%): 1 (5.9)	
						Total		n (%): 9 (52.9)	
			stage IIIC	61		In transit/satellite		n (%): 11 (18)	

Study name	Country	Patient population	Type of subgroup	N	Timepoint	Type	Definition	Results	*p*-value
						Nodal		n (%): 8 (13.1)	
						Distant		n (%): 11 (18)	
						Nodal + distant		n (%): 4 (6.6)	
						Total		n (%):35 (57.4)	
			stage IIID	8		In transit/satellite		n (%): 2 (25)	
						Nodal		n (%): 2 (25)	
						Distant		n (%): 4 (50)	
						Nodal + distant		n (%): 0	
						Total		n (%): 8 (100)	
Bleicher 2020 (40)	USA	Cutaneous melanoma with recurrence	stage II	158	Median (IQR): 4.9	Local /in-transit recurrence	NR	n (%):31 (19.6%)	NR

Study name	Country	Patient population	Type of subgroup	N	Timepoint	Type	Definition	Results	*p*-value
					(2.5-7.9) years	Regional nodal		n (%): 47 (28.8%)	
						Distant		n (%): 80 (50.6%)	
Feigelson 2019 (10)	USA	Invasive melanoma with recurrence	stage I-IV	NR	Median: 4.1 years	Distant recurrence	NR	20%	NR
						Local recurrence	NR	18%	
Tarhini 2018 (43)	USA	Non metastatic melanoma	NR	6,400	Median: 23.1 months	Locoregional	NR	n (%): 1,116 (17.4)	NR
						Distant		n (%): 102 (1.6)	
						Both locoregional and distant		n (%): 27 (0.4)	
Ertekin 2021 (51)	Spain		stage IA-IIID	784	Median (IQR):	Locoregional	NR	n (%): 393 (50.1)	NR

Study name	Country	Patient population	Type of subgroup	N	Timepoint	Type	Definition	Results	*p*-value
					6.89 (2.91-12.16) years	Mixed	Detection of both locoregional and distant metastases synchronously within a 3-month interval from the first recurrence	n (%): 98 (12.5)	
						Distant	NR	n (%): 293 (37.4)	
		Cutaneous melanoma with recurrence	stage II	171		Locoregional	NR	n (%): 102 (59.6)	
						Mixed	Detection of both locoregional and distant metastases synchronously within a 3-month interval from the first recurrence	n (%): 19 (11.1)	
						Distant	NR	n (%):50 (29.2)	
			stage II	260		Locoregional	NR	n (%):137 (52.7)	

Study name	Country	Patient population	Type of subgroup	N	Timepoint	Type	Definition	Results	*p*-value
						Mixed	Detection of both locoregional and distant metastases synchronously within a 3-month interval from the first recurrence	n (%): 32 (12.3)	
						Distant	NR	n (%): 91 (35.0)	
			stage III	353		Locoregional	NR	n (%): 154 (43.6)	
						Mixed	Detection of both locoregional and distant metastases synchronously within a 3-month interval from the first recurrence	n (%): 47 (13.3)	
						Distant	NR	n (%): 152 (43.1)	
Leeneman 2019 (45)	Netherlands	Localized and regionally	Stage IB	57		Local recurrence	NR	n (%):3 (5)	

Study name	Country	Patient population	Type of subgroup	N	Timepoint	Type	Definition	Results	*p*-value
		advanced cutaneous melanoma				Intralymphatic metastasis		n (%):10 (18)	
						Regional lymph node metastasis		n (%):24 (42)	
						Distant metastasis		n (%):20 (35)	
			Stage II	137		Local recurrence		n (%): 4 (3)	
						Intralymphatic metastasis		n (%): 25 (18)	
						Regional lymph node metastasis		n (%): 51 (37)	
						Distant metastasis		n (%):57 (42)	
			Stage III	81		Local recurrence		n (%): 2 (2)	
						Intralymphatic metastasis		n (%): 15 (19)	

Study name	Country	Patient population	Type of subgroup	N	Timepoint	Type	Definition	Results	*p*-value
						Regional lymph node metastasis		n (%): 25 (31)	
						Distant metastasis		n (%): 39 (48)	
			Stage IB	57		Distant metastases		n (%): 36 (63)	
						First recurrence		n (%): 20 (56)	
						Second recurrence or higher		n (%): 16 (44)	
			Stage II	137		Distant metastases		n (%): 91 (66)	
						First recurrence		n (%): 57 (63)	
						Second recurrence or higher		n (%): 34 (37)	
			Stage III	81		Distant metastases		n (%): 54 (67)	

Study name	Country	Patient population	Type of subgroup	N	Timepoint	Type	Definition	Results	*p*-value
						First recurrence		n (%): 39 (72)	
						Second recurrence or higher		n (%): 15 (28)	
Sarac 2020 (9)	Germany	Superficially spreading melanoma, nodular melanoma, lentigo malignant melanoma, acrolentiginous melanoma, and others	Stage IA-IIC)	1,537	3 months (minimum)	Locoregional	NR	n (%): 1,080 (70.3)	NR
						Distant		n (%): 457 (29.7)	
			Stage IA-IIC-LR>10 years	99		Locoregional		n (%): 54 (54.5)	
						Distant		n (%): 45 (45.5)	
			Stage IA-IIC- ER ≤10 years	1,438		Locoregional		n (%): 1,026 (71.3)	
						Distant		n (%): 412 (28.7)	
	Italy	Melanoma with late recurrence	Stage I/II	1372	Median (range):	Regional	NR	n (%): 52 (4)	NR
						Distant		n (%): 25 (2)	

Study name	Country	Patient population	Type of subgroup	N	Timepoint	Type	Definition	Results	*p*-value
Osella-Abate 2015 (12)					5.4 (0.1-30.1) years	Nodal and transit metastasis		N (%): 2 (0.1)	
Loidi 2021 (8)	Spain	Cutaneous melanoma with relapse	Stage I-III	308	Mean: 68.63 months	Locoregional	Locoregional: including intralymphatic metastasis and regional nodes<br>Haemato-visceral: including distant nodes and visceral ones	n (%): 49 (16)	NR
						Haemato-visceral	NR	n (%): 46 (15)	
Ogata 2021 (46)	Japan	Sentinel node-positive melanoma, who underwent CLND	Stage III	45	Pre-June 2017 group (median): 1107 days	Transit metastases	NA	n (%): 19 (42.2)	NR

Study name	Country	Patient population	Type of subgroup	N	Timepoint	Type	Definition	Results	*p*-value
		Sentinel node-positive melanoma who underwent course observation		7	Post-July 2017 group (median): 587.5 days	Regional lymph node metastasis		n (%): 6 (86.7)	
		Overall		119		Regional lymph node		n (%): 11 (9.2)	
						Distant metastasis		n (%): 21 (17.6)	
						Transit metastases		n (%): 20 (16.8)	
Chen 2021 (44)	Taiwan	Conjunctival melanoma with recurrence	Stage T1-T3	20	Mean (SD): 68.7 (55.8)	Local recurrence	NR	n (%): 6 (30)	NR
						Distant metastasis		n (%): 12 (60)	
	Australia			700	NR	Distant	NR	n (%): 28 (4)	

Study name	Country	Patient population	Type of subgroup	N	Timepoint	Type	Definition	Results	*p*-value
Von Schuckman 2019 (11)		Newly diagnosed localized melanoma at a high risk of metastasis, with recurrence according to AJCC 8 or AJCC 7	Stage IB-IIC			Locoregional		n (%): 66 (9.4)	
Chakera 2019 (41)	Australia	Subungual melanoma of the hand with recurrence	Stage IA-IIIB/C	103	Median: 105 months	Local recurrence	NR	n (%): 5 (4.9)	
						In transit recurrence		n (%): 8 (7.8)	
						Regional recurrence		n (%): 17 (16.5)	
						Distant recurrence		n (%): 14 (13.6)	
		Recurrent population (Stage I-III)		44		Local recurrence		n (%): 5 (11)	
						In transit recurrence		n (%): 17 (39)	

Study name	Country	Patient population	Type of subgroup	N	Timepoint	Type	Definition	Results	*p*-value
						Regional recurrence		n (%): 14 (32)	
						Distant recurrence		n (%): 8 (18)	
Tas 2019 (49)	Turkey	Local and regional cutaneous melanoma,	Stage I-II	457	Median (range): 733.6 (36.2–271.8) months	Distant	NR	n (%): 42 (9.2)	
						Locoregional		n (%):111 (24.2)	
						Simultaneous locoregional and distant		n (%):43 (9.4)	
			Stage III	250		Distant		n (%): 55 (22)	
						Locoregional		n (%):71 (28.4)	
						Simultaneous locoregional and distant		n (%):43 (17.2)	
Tas 2017 (50)	Turkey	Early-stage cutaneous	Stage I-III	332	3-years	Locoregional metastasis	NR	45.69%	NR

Study name	Country	Patient population	Type of subgroup	N	Timepoint	Type	Definition	Results	*p*-value
		melanoma with recurrence				Distant metastasis alone		9.55%	
					5-years	Locoregional metastasis		35.24%	
						Distant metastasis alone		6.45%	
Burns 2019 (52)	NR	melanoma	Stage III	370	Median: 5.3 years	Local/regional	NR	n (%): 21 (6)	NR
						Distant		n (%): 60 (16)	

Abbreviations: NR: not reported; USA: United States of America.

Table 20. Survival outcomes in patients with melanoma (n = 3 studies)

Study name	Country	Patient population	Type of subgroup	N	Time-point	Follow-up	Type of survival	Definition	Results	*p*-value
Leeneman 2019 (45)	Netherlands	Localized and regionally advanced cutaneous melanoma	Stage IB	57	NR	Median: 5.4 years	Post-recurrence OS	NR	Median (95% CI): 1.9 (0.8-3.2) years	NR
					2 years				41%	
					3 years				38.1%	
					5 years				27.9%	
			Stage II	137	NR				Median (95% CI): 1.5 (1.1-2.1) years	

Study name	Country	Patient population	Type of subgroup	N	Time-point	Follow-up	Type of survival	Definition	Results	*p*-value
					2 years				42%	
					3 years				35.2%	
					5 years				24.1%	
			Stage III	81	NR				Median (95% CI): 1.1 (0.6-2.2) years	
					2 years				43%	
					3 years				35.2%	
					5 years				25.7%	
			Regional lymph node metastases	100	NR				Median (95% CI): 3.9 (2.5-NR) years	
					2 years				65%	
					3 years				57.6%	
					5 years				58%	
			Intratympanic metastases	116	NR				Median (95% CI): 2.8 (1.9-4.6) years	
					2 years				57%	
					3 years				45.3%	
					5 years				31.3%	
			Distant metastases	NR	NR				Median (95% CI): 0.5 (0.3-0.6) years	
					2 years				12%	
					3 years				6.8%	
					5 years				5.7%	
			Stage II	137	3 years				35.2%	
			Stage III	81					35.2%	
			Stage IB	57					38.1%	
			Stage II	137	5 years				24.1%	
			Stage III	81					25.7%	

Study name	Country	Patient population	Type of subgroup	N	Time-point	Follow-up	Type of survival	Definition	Results	*p*-value
			Stage IB	57					27.9%	
Loidi 2021 (8)	Spain	Cutaneous melanoma	Stage I-III	308	NR	Mean: 68.63 months	OS	Cumulative survival after diagnosis	Mean (95% CI): 56.94 (42.16–71.72) months<br>Median: 27 months	NR
Rockberg 2016 (48)	Sweden	Cutaneous malignant melanoma	Stage I	2,523	5 years	Median (range): 4.43 (0-9.8) years	OS after recurrence / progression	NR	88.2% (95%CI: 65.1-96.4)	NR
			Stage II	746					75.1% (95%CI: 62.3-84.1)	
			Stage III	239					42.9% (95%CI: 33.2-52.2)	

Abbreviations: CI: confidence interval; survival; NR: not reported; OS: overall survival; SD: standard deviation.

Non-small cell lung cancer (NSCLC)

Table 21: Recurrence rates in patients with NSCLC (n = 5 studies)

Study name	Country	Patient population	Type of subgroup	N	Timepoint	Follow-up	n (%) patients with recurrence	*p*-value
Chouaid 2018 (53)	France	Completely resected stage IB-IIIA NSCLC	stage IB-IIIA	251	3 years	Median: 26 months	49%	NR
	Germany			287			41%	
	UK			293			41%	
	France			251	NR	30 months	43	
	Germany			287		24 months.	31	
	UK			293		25 months	25.6	
Slim 2021 (54)	Tunisia	Patients operated for NSCLC, stage pT IB-IIIA	pT IA	10	6.7 years	Mean (SD): 770 (79) days	1 (10)	NR
			pT IB	4			0 (0)	
			pT IIA	6			3 (50)	
			pT IIB	18			7 (39)	
			pT IIIA	25			13 (52)	
			pT IIIB	1			1 (100)	
Li 2019 (55)	NR	Resectable NSCLC; Primary lung adenocarcinoma, GEO	Stage I-IIIA, High neutrophil group	NR	1 year	NR	19.5%	NR
			Stage I-IIIA, Low neutrophil group				6.1%	
		Resectable NSCLC; Primary lung adenocarcinoma, TCGA	Stage I-IIIA, High neutrophil group				27.3%	

Study name	Country	Patient population	Type of subgroup	N	Timepoint	Follow-up	n (%) patients with recurrence	*p*-value
			Stage I-IIIA, Low neutrophil group				19%	
Martin 2022 (15)	Argentina, Chile, Colombia, Dominican Republic, Mexico, Peru and Uruguay	NSCLC	Stage III	203	6.6 years	Median (range): 660 (7–2404) days	136 (67)	NR
			Stage IIIA	89			63 (70.8)	
			Stage IIIB	80			59 (73.8)	
		Resectable NSCLC	Stage III	59			29 (49.2)	
		Unresectable NSCLC		116			87 (75)	
Kumar 2019 (56)	NR	NSCLC (Surgery group)	Stage I	22	NR	Median follow-up 12.8 months;	5 (22%)	NR
		NSCLC (Radiation group)		11	NR	Median follow-up 11.4 months	4 (36%)	NR

Abbreviations: NR: not reported; NSCLC: non-small cell lung cancer; SD: standard deviation; TCGA: The Cancer Genome Atlas; UK: United Kingdom.

Table 22: Type of recurrence in patients with NSCLC (n = 3 studies)

Study name	Country	Patient population	Type of subgroup	N	Timepoint	Follow-up	Type	Results	*p*-value
Karacz 2020 (14)	USA	NSCLC	Stage I-III	NR	NR	NR	Distant	NR (54)	NR
	USA			148	NR	NR	Distant	87 (58.7)	NR

Study name	Country	Patient population	Type of subgroup	N	Timepoint	Follow-up	Type	Results	*p*-value
Buck 2015 (57)		NSCLC with recurrence	Stage IB to IIIA				Local or regional	61 (41.2)	
Chouaid 2018 (53)	France, Germany, UK	Recurrent completely resected NSCLC, overall	Stage IB-IIIA	272	NR	Median: 26 months	Local only	86 (31.6)	NR
		Recurrent Completely resected NSCLC, France		108				38 (35.2)	
		Recurrent completely resected NSCLC, Germany		89				26 (29.2)	
		Recurrent completely resected NSCLC, UK		75				22 (29.3)	
		Recurrent completely resected NSCLC, overall		272			Distant metastases with local or regional recurrence	60 (22.1)	

Study name	Country	Patient population	Type of subgroup	N	Timepoint	Follow-up	Type	Results	*p*-value
		Recurrent Completely resected NSCLC, France		108				21 (19.4)	
		Recurrent completely resected NSCLC, Germany		89				21 (23.6)	
		Recurrent completely resected NSCLC, UK		75				18 (24)	
		Recurrent completely resected NSCLC, overall		272			Further progression to distant metastases (for patients with local/regional recurrence only)	14 (16.3)	
		Recurrent Completely resected NSCLC, France		108				6 (15.8)	
		Recurrent completely resected NSCLC, Germany		89				3 (11.5)	

Study name	Country	Patient population	Type of subgroup	N	Timepoint	Follow-up	Type	Results	*p*-value
		Recurrent completely resected NSCLC, UK		75				5 (22.7)	

Abbreviations: NR: not reported; NSCLC: non-small cell lung cancer; UK: United Kingdom; USA: United States of America.

Table 23: Site of recurrence in patients with NSCLC (n = 1 study)

Study name	Country	Patient population	Type of subgroup	N	Timepoint	Follow-up	Site	Results	*p*-value
Chouaid 2018 (53)	France, Germany, UK	NSCLC patients with metastasis	Stage IB-IIIA	200	NR	Median: 26 months	Brain	82 patients (41%)	NR
							Lung	65 patients (33%)	
							Bone	47 patients (24%)	
							Liber	26 patients (13%)	

Abbreviations: NR: not reported; NSCLC: non-small cell lung cancer; UK: United Kingdom.

Table 24: Time to recurrence in patients with NSCLC (n = 1 study)

Study name	Country	Patient population	Type of subgroup	N	Follow-up	Results	*p*-value
Buck 2015 (57)	USA	NSCLC patients	Stage IB-IIIA	609	NR	Median (range): 12.56 (0.82-71.54) months	0.2208

Study name	Country	Patient population	Type of subgroup	N	Follow-up	Results	*p*-value
		NSCLC patients	Stage IB	215		Median (range): 14.33 (3.78-61.64) months	
		NSCLC patients	Stage IIA/II	130		Median (range): 12.82 (1.61-62.40) months	
		NSCLC patients	Stage IIB	110		Median (range): 11.31 (1.94-65.16) months	
		NSCLC patients	Stage IIIA	154		Median (range): 10.68 (0.82-71.54) months	

Abbreviations: NR: not reported; NSCLC: non-small cell lung cancer; USA: United States of America.

Renal cell carcinoma (RCC)

Table 25. Recurrence rates in patients with RCC (n = 3 studies)

Study name	Country	Patient population	Type of subgroup	N	Time point	Follow-up	n (%) patients with recurrence	*p*-value
Dabestani 2016 (58)	Sweden	RCC (M0, and M1)	Overall	623	60 months	60 months	623 (20.1)	NR
			Males				384 (20.7)	
			Females				239 (19.1)	
			Stage T1a				54 (5.4)	
			Stage T1b				126 (15.3)	
			Stage T2				153 (25.7)	
			Stage T3				263 (42.1)	
			Stage T4				21 (60)	
			Stage Tx				6 (25)	
			Fuhrman grade, G1				23 (6.3)	

Study name	Country	Patient population	Type of subgroup	N	Time point	Follow-up	n (%) patients with recurrence	*p*-value
			Fuhrman grade, G2				196 (13.3)	
			Fuhrman grade, G3				239 (31.7)	
			Fuhrman grade, G4				107 (55.2)	
			Fuhrman grade, Gx				36 (23.5)	
			N stage at the time of diagnosis, N0				415 (19.4)	
			N stage at the time of diagnosis, N1-2				73 (59.3)	
			N stage at the time of diagnosis, NX				135 (15.9)	
			Histopathology, Clear cell				529 (22)	
			Histopathology, Papillary				47 (13.1)	
			Histopathology, Chromophobe				13 (8.6)	
			Histopathology, Other kidney cancer				13 (22)	
Thorstenson 2015 (59)	Sweden	RCC	Stages T0-T4	8,556	60 months	60 months	1,711 (20)	NR

Study name	Country	Patient population	Type of subgroup	N	Time point	Follow-up	n (%) patients with recurrence	*p*-value
Alvarado 2019 (60)	NR	Tubulocystic RCC with disease progression	pT2-pT4	25	NR	12 – 72 months	23 (92)	NR

Abbreviations: NR: not reported; RCC: renal cell carcinoma.

Table 26: Type of RCC cancer recurrence (n = 2 studies)

Study name	Country	Patient population	Type of subgroup	N	Timepoint	Follow-up	Type	Results, n (%)	p-value
Dabestani 2016 (58)	Sweden	RCC	T1a	1003	60 months	60 months	Metastases/ local recurrence	5.4%	NR
			T1b	826				15.3%	
			T2	595				25.7%	
			T3	624				42.1%	
			T4	35				60.0%	
Alvarado 2019 (60)	NR	Tubulocystic RCC with disease progression	pT2-pT4	23	NR	12 – 72 months	Local	11 (47.8)	NR
							Distant	12 (52.2)	

Abbreviations: NR: not reported; RCC: renal cell carcinoma.

Table 27: Site of RCC recurrence (n = 3 studies)

Study name	Country	Patient population	Typo of subgroup	N	Timepoint	Follow-up	Site	Results	p-value
Dabestani 2016 (58)	Sweden	RCC with recurrence	M0, and M1	623	NR	60 months	Lung	54%	NR
							Lymph nodes	22%	
							Bone	20%	
							Liver	15%	

<table>
<tr><th>Study name</th><th>Country</th><th>Patient population</th><th>Typo of subgroup</th><th>N</th><th>Timepoint</th><th>Follow-up</th><th>Site</th><th>Results</th><th>p-value</th></tr>
<tr><td rowspan="2">Alvarado 2019 (60)</td><td rowspan="2">NR</td><td rowspan="2">Tubulocystic RCC with disease progression</td><td rowspan="2">pT2-pT4</td><td rowspan="2">23</td><td rowspan="2">NR</td><td rowspan="2">12 – 72 months</td><td>Para-aortic lymph nodes, bone and liver</td><td>12 (52%)</td><td rowspan="2">NR</td></tr>
<tr><td>Intraabdominal metastases</td><td>2 (8.69%)</td></tr>
<tr><td>Thorstenson 2015</td><td>Sweden</td><td>Newly diagnosed RCC</td><td>T0-T4</td><td>8,556</td><td>NR</td><td>5 years</td><td>The most frequent sites of recurrence were found to be the lung, followed by the bone, lymph nodes, and liver</td><td>NR</td><td>NR</td></tr>
</table>

Abbreviations: NR: not reported; RCC: renal cell carcinoma.

Table 28. Time to recurrence in patients with RCC (n = 1 study)

Study name	Country	Patient population	Type of subgroup	N	Time-\point	Follow-up	Results	p-value
Dabestani 2016 (58)	Sweden	RCC	M0, and M1	623	NR	60 months	Mean (SD): 24 months (± 20 months).	NR

Abbreviations: NR: not reported; RCC: renal cell carcinoma; SD: standard deviation.

Triple negative breast cancer (TNBC)

Table 29. Recurrence rates in TNBC (n = 13 studies)

Study name	Country	Patient population	Type of subgroup	N	Time point	Follow-up	n (%) patients with recurrence	*p*-value
Kaplan 2017 (61)	USA	TNBC; Stage I-III	Age: 25-64	612	23 years	Median (range): 6.23 (0.4-23) years<br>Mean: 7.34 years	126 (21)	NR
			Age: 65-74	100			12 (12)	
			Age: 75+	59			12 (20)	
			Age: 25-64	612	2 years		123 (22)	
			Age: 65-74	100			12 (14)	
			Age: 75+	59			12 (23)	
		TNBC; Stage I	Age: 25-64	NR	5 years		NR (18)	
			Age: 65-74				NR (11)	
			Age: 75+				NR (19)	
James 2019 (62)	New Zealand	TNBC	T1-T4, N0-N3, Non-metastatic	1390	NR	Median: 3.5 years	343 (25%)	NR
	USA	TNB SIII	Overall	414	NR		110 (26.6)	NR

Study name	Country	Patient population	Type of subgroup	N	Time point	Follow-up	n (%) patients with recurrence	*p*-value
Steward 2014 (63)			3-year recurrence rate	110	3 years	Median: 29 months	18.6%	
			5-year recurrence rate	110	5 years		22.6%	
			Locoregional recurrence	19	140.9 months		19 (17.27)	
			Distant recurrence	70	140.9 months		70 (63.64)	
			Locoregional + Distant recurrence	21	140.9 months		21 (19.09)	
			Locoregional recurrence	19	3 years		NR (3.2)	
			Locoregional + Distant recurrence	21			NR (4.42)	

Study name	Country	Patient population	Type of subgroup	N	Time point	Follow-up	n (%) patients with recurrence	*p*-value
			Distant recurrence	70			NR (11.26)	
			Locoregional recurrence	19	5 years		NR (3.65)	
			Locoregional + Distant recurrence	21			NR (4.73)	
			Distant recurrence	70			NR (14.68)	
		TNBC	Clinical stage I	19	140.9 months		Locoregional: 6 (31.57)	
			Clinical stage II	49			Locoregional: 7 (14.28)	
			Clinical stage III	29			Locoregional: 2 (6.89)	
		TNBC	Clinical stage I	19			Distant: 12 (63.15)	
			Clinical stage II	49			Distant: 33 (67.34)	
			Clinical stage III	29			Distant: 18 (62.06)	

Study name	Country	Patient population	Type of subgroup	N	Time point	Follow-up	n (%) patients with recurrence	*p*-value
		TNBC	Clinical stage I	19			Both: 1 (5.26)	
			Clinical stage II	49			Both: 9 (18.36)	
			Clinical stage III	29			Both: 9 (31.03)	
Dawood 2012 **(64)**	USA	TNBC; Stage I-III	Overall patients with distant recurrence	2448	233 months	Median (Range): 39 months (1-233 month)	805 (32.9)	NR
			Overall patients with distant recurrence	2448	2 years		NR (24.9)	NR
			Overall patients with distant recurrence	2448	5 years		NR (38.5)	NR

Study name	Country	Patient population	Type of subgroup	N	Time point	Follow-up	n (%) patients with recurrence	*p*-value
			Patients with brain metastasis	115	2 years		NR (3.7); 95% CI: 2.9-4.5	NR
			Patients with brain metastasis	115	3 years		NR (48.2)	NR
			Patients with brain metastasis	115	5 years		NR (5.4); 95% CI: 4.4-6.5	NR
			Subgroup: Stage I patients with brain metastasis	616	5 year		NR (2.8)	NR

Study name	Country	Patient population	Type of subgroup	N	Time point	Follow-up	n (%) patients with recurrence	*p*-value
			Subgroup: Stage II patients with brain metastasis	1256	5 year		NR (4.6)	NR
			Subgroup: Stage III patients with brain metastasis	576	5 year		NR (9.6)	<0.0001
			Age: <50 years	1189	2 years		65 (4); 95% CI: 2.9-5.3	NR
			Age: ≥50 years	1259	2 years		50 (3.4); 95% CI: 2.3-4.4	NR
			Pre-Menopausal status	1023	2 years		53 (4); 95% CI: 2.7-5.3	NR
			Post Menopausal status	1410	2 years		62 (3.6); 95% CI: 2.5-4.6	NR

Study name	Country	Patient population	Type of subgroup	N	Time point	Follow-up	n (%) patients with recurrence	*p*-value
			Race: White	1596	2 years		82 (3.9); 95% CI: 2.9-4.9	NR
			Race: Black	437	2 years		15 (2.6); 95% CI: 1-4.3	NR
			Race: Other	415	2 years		18 (3.9); 95% CI: 1.8-6	NR
			Stage I	616	2 years		16 (0.008)	NR
			Stage II	1256	2 years		51 (0.031)	NR
			Stage III	576	2 years		48 (0.08)	p<0.0001
			Stage I	616	3 years		NR (0.0188)	NR
			Stage II	1256	3 years		NR (0.0436)	NR
			Stage III	576	3 years		NR (0.0925)	NR
			Nuclear grade: I/II	212	2 years		6 (0.005)	NR
			Nuclear grade: III	2163	2 years		107 (4.1); 95% CI: 3.2-5	NR
			Lympho vascular invasion: Negative	1708	2 years		66 (3.1); 95% CI: 2.2-4	NR
			Lympho vascular invasion: Positive	683	2 years		46 (5.3); 95% CI: 3.5-7	NR
			Age: <50 years	1189	5 years		NR (6); 95% CI: 4.5-7.5	NR

Study name	Country	Patient population	Type of subgroup	N	Time point	Follow-up	n (%) patients with recurrence	*p*-value
			Age: ≥50 years	1259	5 years		NR (4.7); 95% CI: 3.4-6	0.11
			Pre-Menopausal status	1023	5 years		NR (5.8); 95% CI: 4.2-7.5	NR
			Post Menopausal status	1410	5 years		NR (5); 95% CI: 3.7-6.3	0.4
			Race: White	15	5 years		NR (5.8); 95% CI: 4.5-7.1	NR
			Race: Black	437	5 years		NR (3.3); 95% CI: 1.4-5.1	NR
			Race: Other	415	5 years		NR (4.6); 95% CI: 2.3-7	0.38
			Stage I	616	5 years		NR (2.8); 95% CI: 1.2-4.4	NR
			Stage II	1256	5 years		NR (4.6); 95% CI: 3.3-5.9	NR
			Stage III	576	5 years		NR (9.6); 95% CI: 6.9-12.3	p<0.0001
			Nuclear grade: I/II	212	5 years		NR (1.7); 95% CI: 0-3.6	NR
			Nuclear grade: III	2163	5 years		NR (5.7); 95% CI: 4.6-6.8	0.07

Study name	Country	Patient population	Type of subgroup	N	Time point	Follow-up	n (%) patients with recurrence	*p*-value
			Lympho vascular invasion: Negative	1708	5 years		NR (4.3); 95% CI: 3.2-5.3	NR
			Lympho vascular invasion: Positive	683	5 years		NR (7.6); 95% CI: 5.4-9.8	0.009
			Overall	2311	233 months		874 (37.8)	NR
			Patients with BMI <25 kg/m2	794	3 years		NR (32.33)	NR
			Patients with BMI 25–29 kg/m2	692	3 years		NR (32.33)	NR
			Patients with BMI 30+ kg/m2	825	3 years		NR (32.33)	NR
			Patients with BMI <25 kg/m2	794	3 year		NR (37.87)	NR

Study name	Country	Patient population	Type of subgroup	N	Time point	Follow-up	n (%) patients with recurrence	*p*-value
			Patients with BMI 25–29 kg/m2	692	3 year		NR (37.87)	NR
			Patients with BMI 30+ kg/m2	825	3 year		NR (35.78)	NR
			Overall	2311	5 years		NR (43)	NR
			<25 years	794	5 years		NR (44)	NR
			25-29.9 years	692	5 years		NR (44)	NR
			>30 years	825	5 years		NR (42)	NR
			<50 years	428	5 years		NR (49)	NR
			>50 years	366	5 years		NR (38)	NR
			Menopausal status, Pre	389	5 years		NR (49)	NR
			Menopausal status, Post	402	5 years		NR (38)	NR

Study name	Country	Patient population	Type of subgroup	N	Time point	Follow-up	n (%) patients with recurrence	*p*-value
			Race, White	593	5 years		NR (44)	NR
			Race, Black	66	5 years		NR (42)	NR
			Race, Others	135	5 years		NR (39)	NR
			Stage, I	224	5 years		NR (22)	NR
			Stage, II	395	5 years		NR (45)	NR
			Stage, III	161	5 years		NR (59)	NR
			Grade, I	5	5 years		NR (08)	NR
			Grade, II	69	5 years		NR (34)	NR
			Grade, III	702	5 years		NR (44)	NR
			Lymph vascular invasion: Negative	534	5 years		NR (32)	NR
			Lympho vascular invasion: Positive	245	5 years		NR (68)	NR
			pCR: No	191	5 years		NR (52)	NR
			pCR: Yes	71	5 years		NR (09)	NR
			Brain Metastasis: No	757	5 years		NR (4)	NR

Study name	Country	Patient population	Type of subgroup	N	Time point	Follow-up	n (%) patients with recurrence	*p*-value
			Brain Metastasis: Yes	37	5 years		NR (95)	NR
			Visceral Metastasis: No	626	5 years		NR (27)	NR
			Visceral Metastasis: Yes	168	5 years		NR (95)	NR
			Bone Metastasis: No	705	5 years		NR (35)	NR
			Bone Metastasis: Yes	89	5 years		NR (94)	NR
			Local Metastasis: No	627	5 years		NR (27)	NR
			Local Metastasis: Yes	167	5 years		NR (95)	NR
			Patients with BMI <25 kg/m2	794	5 years		NR (43.66)	NR

Study name	Country	Patient population	Type of subgroup	N	Time point	Follow-up	n (%) patients with recurrence	*p*-value
			Patients with BMI 25–29 kg/m2	692	5 years		NR (43.66)	NR
			Patients with BMI 30+ kg/m2	825	5 years		NR (417)	NR
			Overall	2311	5 years		NR (38)	NR
			Patients with BMI <25 kg/m2	794	5 years		NR (39)	NR
			Patients with BMI 25–29 kg/m2	692	5 years		NR (38)	NR
			Patients with BMI 30+ kg/m2	825	5years		NR (38)	NR
			<50 years	428	5 years		NR (44)	NR
			>50 years	366	5 years		NR (32)	NR
			Stage, I	224	5 years		NR (18)	NR
			Stage, II	395	5 years		NR (39)	NR

Study name	Country	Patient population	Type of subgroup	N	Time point	Follow-up	n (%) patients with recurrence	*p*-value
			Stage, III	161	5 years		NR (57)	NR
Tecic 2020 (65)	Croatia	TNBC	T1-T3, N0-N3; early stage	151	5 years	5-Years	50 (32.9)	NR
Ignatov 2018 (17)	Germany	TNBC	Primary, non-metastatic	1,374	During the first 5 years of follow-up	Median (range): 64 (1–209)	NR (70.2)	NR
					First 3 years of follow-up		NR (51.30)	
					3 years		NR (16.40)	
					5 years		NR (19.60)	
					10 year		NR (19.10)	
					209 months		NR (19.2)	
Van 2016 (18)	Netherlands	Overall women with clinically T1-2N0 TNBC; Solitary recurrence	Stage T1-2N0	2,548	5 years	Median: 5-years	328 (12.9)	NR

**Study name**	**Country**	**Patient population**	**Type of subgroup**	**N**	**Time point**	**Follow-up**	**n (%) patients with recurrence**	***p*-value**
		Overall women with clinically T1-2N0 TNBC; Concomitant disease					75 (2.98)	
		Total women with recurrent TNBC					NR (21.30)	
Gonçalves 2018 (66)	Brazil	TNBC	Stage I-III	87	5-years	5-years	37 (42.5)	NR
					5-years	3-years	NR (36%)	
Parshad 2017 (67)	India	TNBC	Stage I-III	98	15-years	Mean: 4.1 years	28 (28.6)	NR
			With recurrence	28	3-years		NR (28.90)	
				28	5-years		NR (44.40)	
Gal 2018 (68)	Israel	TNBC	Non-metastatic; T1-T4; N0-N4	34	3-years	Median (range): 10.6 (8–12) years	NR (24.28)	NR
					5-year		NR (32.59)	
					3 years		NR (12.93)	
					5-year		NR (17.78)	

Study name	Country	Patient population	Type of subgroup	N	Time point	Follow-up	n (%) patients with recurrence	*p*-value
Eralp 2014 (20)	Turkey	TNBC; Stage I-III	Patients with recurrence	75	5-year recurrence rate	Median: 52.2 months	NR (28.4)	NR
			Age: <50	28			NR (33.6)	
			Age: >50	288			NR (28)	
			Family history: (+)	34			NR (20.6)	
			Family history: (-)	282			NR (29.4)	
			Menopause status: premenopausal	154			NR (30.9)	
			Menopause status: postmenopausal	162			NR (25.7)	
			Pathological stage: Stage 1	84			NR (19.6)	

Study name	Country	Patient population	Type of subgroup	N	Time point	Follow-up	n (%) patients with recurrence	*p*-value
			Pathological stage: Stage 2&3	232			NR (31.6)	
			Histological grade: Grade 1&2	76			NR (31.6)	
			Histological grade: Grade 3	200			NR (29.1)	
			Tumor stage: T1&T2	280			NR (23.7)	
			Tumor stage: T3&T4	36			NR (60.6)	
			Nodal positivity: node (-)	163			NR (20.6)	
			Nodal positivity: node (+)	153			NR (35.6)	

Study name	Country	Patient population	Type of subgroup	N	Time point	Follow-up	n (%) patients with recurrence	*p*-value
			Extensive nodal involvement: positive node≤ 10	293			NR (25.1)	
			Extensive nodal involvement: positive node>10	23			NR (59)	
			Tumor size: ≤2 cm	128			NR (22.7)	
			Tumor size: >2 cm	188			NR (31.6)	
			Locally advanced disease: (-)	177			NR (21.3)	
			Locally advanced disease: (+)	139			NR (36.9)	

Study name	Country	Patient population	Type of subgroup	N	Time point	Follow-up	n (%) patients with recurrence	*p*-value
			Site of progression: Locoregional	22			NR (90.9)	
			Site of progression: Distant metastases	55			NR (94.3)	
			Type of operation: Mastectomy	178			NR (32.2)	
			Type of operation: Breast conserving	138			NR (22.9)	
Metzger-Filho 2013 (69)	Multiple countries	TNBC; early stage	Local recurrence	310	3 years	Median: 12.5 years	NR (4.02)	NR
					5 years		NR (4.32)	
					3 years		NR (0.83)	

Study name	Country	Patient population	Type of subgroup	N	Time point	Follow-up	n (%) patients with recurrence	*p*-value
			Contralateral breast recurrence		5 years		NR (1.49)	
			Bone recurrence		3 years		NR (0.72)	
					5 years		NR (1.36)	
Min Sun Bae 2015 (21)	NR	Triple-negative breast cancer	Overall	398	10.4 years	Median (Range): 6.1 years (0.3–10.4 years)	63 (15.83)	NR
			Overall	398	5 year		48 (12.1)	NR
			Non–MR imaging group	53	10.4 years		47 (13.6)	NR
			MR imaging group	345	10.4 years		16 (30.2)	NR
			Non–MR imaging group	53	3 years		NR (15.44)	NR
			MR imaging group	345	3 years		NR (6.96)	NR
			Non–MR imaging group	53	5 years		NR (19.58)	NR

Study name	Country	Patient population	Type of subgroup	N	Time point	Follow-up	n (%) patients with recurrence	*p*-value
			MR imaging group	345	5 years		NR (9.63)	NR
			Age: ≤40 Years	91	10.4 years		19 (30.2)	NR
			Age: 41–49 Years	141	10.4 years		23 (36.5)	NR
			Age: ≥50 Years	166	10.4 years		21 (33.3)	NR
			Symptoms: Absent	53	10.4 years		8 (12.7)	NR
			Symptoms: Present	345	10.4 years		55 (87.3)	NR
			Family history of breast cancer: Absent	374	10.4years		55 (87.3)	NR
			Family history of breast cancer: Present	24	10.4 years		8 (12.7)	NR

Study name	Country	Patient population	Type of subgroup	N	Time point	Follow-up	n (%) patients with recurrence	*p*-value
			Multifocal or multicentric disease: No	359	10.4 years		55 (87.3)	NR
			Multifocal or multicentric disease: Yes	39	10.4 years		8 (12.7)	NR
			Contralateral disease: No	387	10.4 years		63 (100)	NR
			Contralateral disease: Yes	11	10.4 years		0 (0)	NR
			Ductal, NOS	364	10.4 years		56 (88.9)	NR
			Ductal, special type	29	10.4 years		7 (11.1)	NR
			Lobular	5	10.4 years		0 (0)	NR

Study name	Country	Patient population	Type of subgroup	N	Time point	Follow-up	n (%) patients with recurrence	*p*-value
			Tumor grade: Low	4	10.4 years		0 (0)	NR
			Tumor grade: Intermediate	48	10.4 years		5 (7.9)	NR
			Tumor grade: High	346	10.4 years		58 (92.1)	NR
			Tumor size (cm): Tumor size (cm)	2.29	10.4 years		2.27 (6 0.91)	NR
			No. of metastatic lymph nodes: Stage 0	313	10.4 years		44 (69.8)	NR
			No. of metastatic lymph nodes: Stage 1–3	84	10.4 years		18 (28.6)	NR

Study name	Country	Patient population	Type of subgroup	N	Time point	Follow-up	n (%) patients with recurrence	*p*-value
			No. of metastatic lymph nodes: Stage ≥4	1	10.4 years		1 (1.6)	NR
			Stage I	151	10.4 years		19 (30.2)	NR
			Stage II	247	10.4 years		44 (69.8)	NR
			Surgery type: Breast conservation	292	10.4 years		43 (68.3)	NR
			Surgery type: Mastectomy	106	10.4 years		20 (31.7)	NR
			Margin status: Clear	387	10.4 years		61 (96.8)	NR
			Margin status: Close (,2 mm)	11	10.4 years		2 (3.2)	NR
			Lymphovascular invasion: Absent	279	10.4 years		37 (58.7)	NR

Study name	Country	Patient population	Type of subgroup	N	Time point	Follow-up	n (%) patients with recurrence	*p*-value
			Lymphovascular invasion: Present	119	10.4 years		26 (41.3)	NR
			Ki-67 index: <10	204	10.4 years		27 (42.9)	NR
			Ki-67 index: ≥10	194	10.4 years		36 (57.1)	NR
			Mammographic density: Nondense	123	10.4 years		10 (15.9)	NR
			Mammographic density: Dense	275	10.4 years		53 (84.1)	NR
			Preoperative MR imaging: No	53	10.4 years		16 (25.4)	NR
			Preoperative MR imaging: Yes	345	10.4 years		47 (74.6)	NR

Study name	Country	Patient population	Type of subgroup	N	Time point	Follow-up	n (%) patients with recurrence	*p*-value
			Dense breast tissue	NR	3 years		NR (10.95)	NR
			Non-dense breast tissue	NR	3 years		NR (2.72)	NR
			Dense breast tissue	NR	5 years		NR (14.15)	NR
			Non-dense breast tissue	NR	5 years		NR (4.9)	NR
			Family history of breast cancer: Absent	NR	3 years		NR (10.29)	NR
			Family history of breast cancer: Present	NR	3 years		NR (7.86)	NR

Study name	Country	Patient population	Type of subgroup	N	Time point	Follow-up	n (%) patients with recurrence	*p*-value
			Family history of breast cancer: Absent	NR	5 years		NR (10.65)	NR
			Family history of breast cancer: Present	NR	5 years		NR (13.98)	NR
			Subgroup: Lymphovascular invasion: No lymphovascular invasion	NR	3 years		NR (6.5)	NR
			Subgroup: Lymphovascular invasion: Lymphovascular invasion	NR	3 years		NR (11.1)	NR

Study name	Country	Patient population	Type of subgroup	N	Time point	Follow-up	n (%) patients with recurrence	*p*-value
			Subgroup: Lymphovascular invasion: No lymphovascular invasion	NR	5 years		NR (3.53)	NR
			Subgroup: Lymphovascular invasion: Lymphovascular invasion	NR	5 years		NR (15.94)	NR

Abbreviations: BMI: Body Mass Index; CI: confidence interval; NR: not reported, TNBC: triple negative breast cancer, pCR: polymerase chain reaction.

Table 30: Type of recurrence in patients with TNBC (n = 11 studies)

Study name	Country	Patient population	Type of subgroup	N	Timepoint	Follow-up	Type	Results, n (%)	*p*-value
Kaplan 2017 (61)	USA	TNBC, Age: 25-64	NR	612	23 years		Local/regional	22 (4)	0.028
							Distant	104 (17)	
		TNBC, Age: 65-74		100			Local/regional	0 (0)	
							Distant	12 (12)	

Study name	Country	Patient population	Type of subgroup	N	Timepoint	Follow-up	Type	Results, n (%)	*p*-value
		TNBC, Age: 75+		59		Median (range): 6.23 (0.4-23) years Mean: 7.34 years	Local/regional	5 (9)	
							Distant	7 (12)	
		TNBC, Age: 25-64		612	2 years		Local/regional	21 (3.7)	NR
							Distant	102 (18)	
		TNBC, Age: 65-74		100			Local/regional	0 (0)	
							Distant	12 (14)	
		TNBC, Age: 75+		59			Local/regional	5 (9.6)	
							Distant	7 (13.5)	
Steward 2014 (63)	USA	TNBC with recurrence	Stage I-III	414	NR	Mean (SD): 68.2 (36.4) months	Locoregional	19 (17.3)	NR
							Distant	70 (63.6)	
							Both locoregional and distant recurrence	21 (19.1)	
Dawood 2012 (70)	USA	TNBC	Stage I-III	2,311	NR	Median (range): 39 (1-233) months	Distant metastasis	771 (33.4)	NR
Haiderali 2021b (16)	USA	TNBC	Stage II-IIIB	308	Time 3 (Locoregional recurrence to metastatic diagnosis, death or end of record.	Median: 46.1 months	Locoregional	21 (6.8)	NR
	Germany	TNBC		1,374	NR		Local	105 (7.6)	NR

Study name	Country	Patient population	Type of subgroup	N	Timepoint	Follow-up	Type	Results, n (%)	*p*-value
Ignatov 2018 (17)			Primary, non-metastatic			Median (range): 64 (1–209)	Regional (lymph nodes)	45 (3.3)	
							Distant	99 (7.2)	
		Recurrence patients in TNBC	NR	264	NR	Median (range): 64 (1–209)	Local	105 (39.77)	
							Regional (lymph nodes)	45 (17.04)	
							Distant	99 (37.5)	
James 2019 (62)	New Zealand	Non-metastatic TNBC with recurrence	Non-metastatic	343	NR	Median: 3.5 years	Locoregional	88 (25.6)	NR
							Concurrent locoregional and systemic	49 (14.2)	
							Systemic only	206 (60.2)	
							Distant	NR (74)	
		Non-metastatic recurrent TNBC, Mastectomy		229	NR		Local and regional or regional only	16 (7)	
							Local and regional or regional alone with systemic	34 (15)	
							Systemic only	152 (66)	
							Local only	27 (12)	
				27			Systemic disease after local recurrence	13 (48)	

Study name	Country	Patient population	Type of subgroup	N	Timepoint	Follow-up	Type	Results, n (%)	*p*-value
				43			Systemic disease after locoregional recurrence	22 (51)	
		Non-metastatic recurrent TNBC, Wide local excision		105			Local and regional or regional only	8 (8)	
							Local and regional or regional alone with systemic	15 (14)	
							Systemic only	46 (44)	
							Local only	36 (34)	
				36			Systemic disease after local recurrence	12 (33)	
				44			Systemic disease after locoregional recurrence	17 (40)	
Van 2016 (18)	Netherlands	TNBC	Stage T1-2, N0	2,548	During follow-up of 5 years	Median: 5 years	Regional	25 (1)	NR
							Local	60 (2.4)	
							Distant	243 (9.5)	
		TNBC with concomitant disease	NR	403			Local and regional	10 (0.4)	
							Local and distant	25 (1)	
							Regional and distant	28 (1.1)	

Study name	Country	Patient population	Type of subgroup	N	Timepoint	Follow-up	Type	Results, n (%)	*p*-value
							Local, regional, and distant	12 (0.5)	
		TNBC with regional recurrence	NR	403			Local	60 (14.85)	
							Regional lymph node	25 (6.20)	
							Distant metastatic	243 (60.30)	
							Local and regional	10 (2.5)	
							Local and distant	25 (6.2)	
							Regional and distant	28 (6.9)	
							Local, regional, and distant	12 (3)	
Parshad 2017 (67)	India	TNBC with recurrence	Stage I-III	28	NR	Mean: 4.1 years	Distant	18 (64.2)	NR
Eralp 2014 (20)	Turkey	TNBC	Stage I-III	316	NR	Median: 52.2 months	Locoregional	16 (5.1)	NR
							Locoregional+ distant	2 (0.6)	
							Distant	51 (16.1)	
		TNBC Patients with recurrence	Stage I-III	75	NR		Locoregional	51 (68)	
							Locoregional+ distant	16 (21.3)	
							Distant	2 (2.7)	
Metzger-Filho 2013 (69)	Multiple countries	TNBC	Early stage	310	10 years	Median: 12.5 years	Local	22 (6.60) (SE): 6.60 (0.01)	NR

Study name	Country	Patient population	Type of subgroup	N	Timepoint	Follow-up	Type	Results, n (%)	*p*-value
							Nodal (no chemotherapy)	10 (7.71), (SE: 0.02)	
							Nodal (chemotherapy)	5 (2.79), (SE: 0.01)	
							Contralateral breast	16 (3.95) (SE: 0.01)	
							Nodal (no CT)	11 (7.71) (SE: 0.02)	
							Nodal (CT)	5 (2.71) (SE: 0.01)	
							Bone	6 (2.01) (SE: 0.01)	
							Visceral (no CT)	21 (16.26), (SE: 0.03)	
							Visceral (CT)	16 (7.82) (SE: 0.02)	
Min Sun Bae 2015 (21)	NR	TNBC with recurrence	Stage I-II	63	NR	Median (range): 6.1 (0.3–10.4) years	Local-regional	20 (31.7)	NR
							Distant	20 (31.7)	
							Both local-regional and distant recurrences	10 (15.8)	
				53	10.4-years		Non–MR imaging group Locoregional	9 (3.8)	
				345	10.4-years		MR imaging group Locoregional	7 (2)	

Abbreviations: CT: chemotherapy; NR: not reported; SD: standard deviation; SE: standard error; TNBC: triple-negative breast cancer; USA: United States of America.

Table 31: Site of recurrences in patients with TNBC (n = 8 studies)

Study name	Country	Patient population	Type of subgroup	N	Timepoint	Follow-up	Site	Definition	Results, n (%)	*p*-value
Eralp 2014 (20)	Turkey	TNBC	Stage I-III	316	NR	Median: 52.2 months	Brain	Number of events per total of 55 events	7 (12.7)	NR
							Liver		13 (23.6)	
							Lung		24 (43.6)	
							Bone		18 (32.7)	
Matro 2015 (19)	USA	TNBC with distant recurrence	Stage III	72	NR	Median: 29 months	Bone/Bone marrow	Diagnosis of first site of recurrence was identified based. on the first date of diagnosis of recurrent disease	17 (24)	NR
							Brain/CNS/Meninges		12 (17)	
							Lung/Pleural Effusion		18 (25)	
							Liver		17 (24)	
							Chest wall		15 (21)	
							Regional lymph nodes		10 (14)	
							Contralateral Locoregional Lymph Nodes		5 (7)	
							Skin		4 (6)	
							Ipsilateral Breast		1 (1)	
							Other		18 (25)	
Dawood 2012 (70)	USA	TNBC	Stage I-III	2,448	NR	Median (range): 39 (1-233) months	Brain	First site of distant recurrence	115 (4.7)	NR
			Stage I	616					16 (13.9)	
			Stage I	1,256					51 (44.3)	
			Stage I	576					48 (41.7)	
		TNBC (stage I-III)	Stage I-III	NR			Brain metastasis: BMI <25 kg/m2		37 (4.7)	NR

Study name	Country	Patient population	Type of subgroup	N	Timepoint	Follow-up	Site	Definition	Results, n (%)	*p*-value
							Brain metastasis: BMI25–29kg/m2		35(5.1)	
							Brain metastases: BMI30+kg/m2		33(4.0)	
							Visceral metastasis: BMI<25kg/m2		168(21.2)	
							Visceral metastasis: BMI25–29kg/m2		139(20.1)	
							Visceral metastasis: BMI 30+kg/m2		149(18.1)	
							Bone metastasis: BMI<25kg/m2		89(11.2)	
							Bone metastasis: BMI25–29kg/m2		74(10.7)	
							Bone metastasis: BMI30+kg/m2		83(10.1)	
							Local metastasis: BMI<25kg/m2		167(21.0)	
							Local metastasis: BMI25–29kg/m2		131(18.9)	
							Local metastasis: BMI30+kg/m2		144(17.5)	
Ignatov 2018 (17)	Germany	TNBC	Primary, non-metastatic	1,374	NR	Median (range): 64 (1–209)	>1 metastasis	NR	99 (7.2)	NR
							Distant nodal		52 (3.8)	
							Bone		76 (5.5)	
							Liver		47 (3.4)	

Study name	Country	Patient population	Type of subgroup	N	Timepoint	Follow-up	Site	Definition	Results, n (%)	*p*-value
				(455 with distant metastasis)			Lung		96 (7)	
							Brain		67 (4.9)	
							Other		18 (1.3)	
		TNBC patients with recurrence	NR	455			>1 metastasis		99 (37.5)	
							Distant nodal		52 (19.7)	
							Bone		76 (28.8)	
							Liver		47 (17.8)	
							Lung		96 (36.4)	
							Brain		67 (25.4)	
							Other		18 (6.8)	
James 2019 (62)	New Zealand	TNBC with distant recurrence	Non-metastatic	1,029	NR	Median: 3.5 years	Lung	Occurrence of each metastasis site, the sum is >100% due to multiple metastasis	NR (55.9)	NR
							Bone		NR (44.4)	
							Liver		NR (30.9)	
							Brain		NR (21.9)	
							Multiple metastasis		NR (51)	
Suhani 2017 (67)	India	TNBC with any recurrence	Stage I-III	18 distant metastases	NR	Mean: 4.1 years	CNS	NR	5 (28)	NR
							Lung		NR (11)	
							Liver		NR (6)	
							Soft tissue		NR (33)	
							Multiple sites		NR (22)	
	USA			91	NR		Brian	NR	32 (35.16)	NR

Study name	Country	Patient population	Type of subgroup	N	Timepoint	Follow-up	Site	Definition	Results, n (%)	*p*-value
Steward 2014 (63)		TNBC with distant recurrence	Stage I-III			Mean (SD): 68.2 (36.4) months	Bone		31 (34)	
							Liver		29 (31.87)	
							Lung		38 (41.76)	
Metzger-Filho 2013 (69)	Multiple countries	TNBC	Early stage	310	10-year	Median: 12.5 years	Contralateral breast	First site of breast cancer-free interval	16 (NR)<br>10-year % (SE): 3.95 (0.01)	NR
							Nodal (no chemotherapy)		11 (NR)<br>10-year % (SE): 7.71 (0.02)	
							Nodal (chemotherapy)		5 (NR)<br>10-year % (SE): 2.79 (0.01)	
							Bone		6 (NR)<br>10-year % (SE): 2.01 (0.01)	
							Visceral (no chemotherapy)		21 (NR)<br>10-year % (SE): 16.26 (0.03)	
							Lung		13 (NR)	
							Liver		2 (NR)	
							CNS		4 (NR)	
							Other		3 (NR)	
							Visceral (chemotherapy)		16 (NR)<br>10-year % (SE): 7.82 (0.02)	

Study name	Country	Patient population	Type of subgroup	N	Timepoint	Follow-up	Site	Definition	Results, n (%)	*p*-value
							Lung		6 (NR)	
							Liver		6 (NR)	
							CNS		4 (NR)	
							Other		2 (NR)	

Abbreviations: BMI: body mass index, CNS: central nervous system; NR: not reported; SD: standard deviation; TNBC: triple-negative breast cancer; USA: United States of America.

Table 32: Time to recurrence in patients with TNBC (n = 8 studies)

Study name	Country	Patient population	Type of subgroup	N	Follow-up	Results	*p*-value
Dawood 2012 (70)	USA	TNBC	Stage I-III	2,448	Median (range): 39 (1-233) months	Median (range): 31 (0-214) months	NR
Eralp 2014 (20)	Turkey	TNBC	Stage I-III	316	Median: 52.2 months	Median (SD): 24.4 (1.8)	NR
Haiderali 2021a (16)	USA	TNBC, Neoadjuvant treatment + adjuvant treatment	Stage II-IIIB	72	Median: 46.1 months	Median: 53.4 months	NR
James 2019 (62)	New Zealand	TNBC with recurrence	Non-metastatic	343	Median: 3.5 years	Median: 19.2 months (1.6 years)	NR
Metzger-Filho 2013 (69)	Multiple countries	TNBC	Early stage	310	Median: 12.5 years	BCFI*: 71%	NR
Min Sun Bae 2015 (21)	NR	TNBC	Stage I-II	398	Median (range): 6.1 (0.3–10.4) years	Median (range): 32.4 (4.8-92.4) months	NR
Steward 2014 (63)	USA	TNBC	Stage I-III	414	Mean (SD): 68.2 (36.4) months	Mean (range): 18.8 (3.9-112) months	NR
Van de Wal 2016 (18)	Netherlands	TNBC	Stage T1-2N0	2,548	Median: 5 years	Median (range): 20.4 (1.2-60) months	NR
		TNBC with regional recurrence	NR	75		Median (range): 19.2 (1.2-58.8) months	NR

Note: The actual estimates provided in the study were provided in square brackets. Data were converted to months to enable comparison with other studies.

*BCFI was defined as the length of time from the date of random assignment to any invasive BC recurrence (including ipsilateral or contralateral breast recurrence) and was censored at date of last follow-up or at date of death without recurrence

Abbreviations: BCFI: BC-free interval; NR: not reported; SD: standard deviation; TNBC: triple-negative breast cancer; USA: United States of America.

Table 33: Overall survival outcomes in patients with TNBC (n = 2 studies)

Study name	Country	Patient population	Stage of the disease	N	Time-point	Follow-up	Outcome subtype/parameter	Definition	Results	*p*-value
Steward 2014 (63)	USA	TNBC patients with locoregional recurrence	Stage I-III	19	NR	Mean (SD): 68.2 (36.4) months	1-year OS	NR	80.3%	NR
							2-year OS		73.1%	
							3-year OS		65.8%	
							5-year OS		65.9%	
		TNBC patients with distant recurrence		70			1-year OS		47.8%	
							2-year OS		28.2%	
							3-year OS		23.4%	
							5-year OS		20.3%	
		TNBC patients with both locoregional and distant recurrence		21			1-year OS		38.1%	
							2-year OS		27.8%	
							3-year OS		22.2%	
							5-year OS		16.8%	
Van de Wal 2016 (18)	Netherlands	TNBC with regional recurrence	NR	75	5 years		5-year OS	NR	28.4%	NR

Abbreviations: NR: not reported; SD: standard deviation; TNBC: triple-negative breast cancer; OS: overall survival; USA: United States of America.

All indications

Table 34. Recurrence rates by tumor type according to stage at diagnosis

Tumor type and early-stage criteria*	Stage at diagnosis	1-year recurrence rate (%)	3-year recurrence rate (%)	5-year recurrence rate (%)	10-year recurrence rate (%)
**Bladder cancer**					
**Stages I to IV, excluding unresectable stage III (muscle invasive and non-muscle invasive), stages Ta-T1 (low grade and high grade) and PUNLMP**	T1-T4	15.51-15.70 (1)	21.14-21.38 (1)	22.90-23.82 (1)	
	Stage I		11.40 (28)	19.10 (28)	
	Stage II		30.00 (28)	33.40 (28)	
	Stage III		63.90 (28)	63.90 (28)	
	High grade Ta-T1			64.30-74.20 (27)	69.20-78.80 (27)
	Ta low grade	6.70-29.00 (2, 6, 71)	30.28 (22)	21.70-49.00 (2, 71, 72)	12.50-61.00 (2, 6)
	Ta high grade	24.29 (22)	38.64 (22)	42.19 (22)	70.1 (22)
	Tis	35.65 (22)	49.84 (22)	52.29 (22)	70.6 (22)
	T1 low grade	29.50 (22)	37.38 (22)	43.77 (22)	
	T1 high grade	29.50-43.69 (2, 22)	36.28-54.71 (2, 29, 71)	39.2 -54.68 (2, 22)	77.2 (22)
	G2pTa			33.3 (2)	37.20 (2)
	PUNLMP - low	10.33 (22)	22.95 (22)	25.79 (22)	
**HNC**					
	Stage I				13.38 (34)

**Stage I-IV, stage T1-T4**	Stage II				32.42 (34)
	Stage III				34.23 (34)
	Stage IV				23.73 (34)
	Stage I-IV		39.60-72.89 (35)	52.35-78.29 (35)	63.49-78.39 (35)
	Stage T1-T4				1.44-45.19 (35)
Melanoma					
**Stages IA to IIID, excluding unresectable stage III, stage T1-T4**	Stage I-III (overall)		9.55 - 50.00 (44, 50)	6.45 – 65.00 (7, 8, 41, 44, 50)	29.90 - 81.00 (8, 41, 44)
	Stage T1-T4		7.90 (1)	10.00 (1)	
	Stage I		5.56-10.55 (7, 48)	9.59-13.13 (7, 48)	
	Stage IA			9.30 (7)	
	Stage IA-stage IIC				10.80 (9)
	Stage IB		4.20 (45)	6.40-10.30 (7, 45)	
	Stage II		24.90-34.04 (7, 45, 48)	29.10-49.90 (7, 45, 48)	
	Stage IIA		12.00-12.27 (7, 40)	13.60-32.20 (7, 40)	
	Stage IIB	12.50 (13)	19.00-36.79 (7, 40)	28.2-94.70 (7, 40)	
	Stage IIC	21.80 (13)	37.00-48.79 (7, 40)	45.00-100.00 (7, 40)	
	Stage III		39.30-80.16 (7, 45, 46, 48)	45.90-82.75 (7, 45, 46, 48)	
	Stage IIIA	28.20 (13)	44.32 (7)	44.30-83.30 (7)	
	Stage IIIB		49.94 (7)	49.80-88.90 (7)	
	Stage IIIC		54.03 (7)	66.60-97.10 (7)	
	Stage IIID			100.00 (7)	
**Non-small cell lung cancer (NSCLC)**					
**Stages I-IIIA**	Stage I–IIIA	6.10-27.30 (55)			
	Stage IB-IIIA		41.00-49.00 (53)		
**Renal cell carcinoma**					

**Stages T1-T4, N1-N2**	Overall T1a-Tx, N0-NX			6.30 – 55.20 (58)	
	N0			19.40 (58)	
	N1-2			59.30 (58)	
	NX			15.90 (58)	
	T1a			5.40 (58)	
	T1b			15.30 (58)	
	T2			25.70 (58)	
	T3			42.10 (58)	
	T4			60.00 (58)	
	Tx			25.00 (58)	
**Triple negative breast cancer (TNBC)**					
**Stages I to III, excluding unresectable stage III, stage T1-2N0**	Stage I		1.90 (64)	2.80-22.00 (61, 63, 64, 70)	30.20 (21)
	Stage II		4.40 (64)	4.60-45.0 (63, 64, 70)	69.80** (21)
	Stage I-II		2.72-15.44 (21)	3.50-19.58 (21)	1.60-96.80 (21)
	Stage II - III			31.60 (20)	
	Stage III		9.30 (64)	9.60-59.00 (63, 64, 70)	
	Stage I-III		0.72-51.30 (17, 19, 63, 64, 67, 68, 69)	1.36-94.30 (17, 19, 20, 63, 64, 65, 67, 68, 69)	19.10-28.60** (17, 21)
	Stage T1-2N0			2.94-21.30 (18)	

*Early-stage criteria used is aligned to that which is defined in the inclusion criteria of the SLR

** 10.4 years of follow up.

Abbreviations: HNC: Head and neck cancer; NMIBC: Non-muscle invasive bladder cancer; NSCLC: Non-small cell lung cancer; PUNLMP: Papillary urothelial neoplasm of low malignant potential; RCC: Renal cell carcinoma; SLR: Systematic literature review; TNBC: Triple negative breast cancer; T: tumor; N0: No cancer near the lymph-nodes; NX: Cancer in nearby lymph nodes cannot be measured; TX: Main tumor cannot be measured.

Appendix S4. Humanistic review findings

Bladder cancer

Table 35. Fear of recurrence outcomes in patients with bladder cancer (n = 1 study)

Study name	Patient population	Time-point	N	QoL scale	QoL domain	Score	Proportion of patients, n (%)	*p*-value
Leclair 2019 (73)	Bladder cancer, stage I-III	NR	89	Cancer Problems in Living Scales (CIPLS) FCR subscale, Fear of Cancer Recurrence Inventory-Severity subscale (FCRI-s)	Low- Fear of cancer recurrence	NR	24 (27%)	NR
					Moderate- Fear of cancer recurrence	NR	55 (61.8%)	NR
					High- Fear of cancer recurrence	NR	10 (11.2%)	NR

Abbreviations: CIPLS: Cancer Problems in Living Scales; FCR: Fear of Cancer Recurrence; FCRI-s: Fear of Cancer Recurrence Inventory-Severity subscale; NR: not reported; QoL: quality of life.

Table 36. HRQoL outcomes in recurrent patients with bladder cancer (n = 1 study)

Study name	Patient population	Timepoint	N	QoL scale	QoL domain	Score Mean (SD) [median]
Smith 2022 (74)	Non-recurrent NMIBC	NR	306	EORTC QLQ-C30	General, mean (SD) [median]	87.2 (11.3) [89.7]
					Global health status/QOL	75.1 (18.5) [83.3]
					Physical functioning	84.2 (20.9) [100.0]
					Role functioning	88.5 (21.2) [100.0]
					Emotional functioning	85.3 (16.4) [83.3]
					Cognitive functioning	85.3 (16.4) [83.3]
					Social functioning	84.4 (20.3) [100.0]
					Fatigue	20.3 (19.9) [11.1]
					Nausea and vomiting	2.8 (7.9) [0.0]

Study name	Patient population	Timepoint	N	QoL scale	QoL domain	Score Mean (SD) [median]
					Pain	14.0 (21.2) [0]
					Dyspnea	12.5 (23.0) [0]
					Insomnia	24.7 (27.5) [33.3]
					Appetite loss	6.4 (16.6) [0]
					Constipation	12.2 (21.9) [0]
					Diarrhea	7.3 (17.3) [0]
					Financial difficulties	11.4 (22.9) [0]
	Recurrent NMIBC	NR	272	EORTC QLQ-C30	General, mean (SD) [median]	85.7 (11.1) [88.0]
					Global health status/QOL	72.8 (20.0) [75.0]
					Physical functioning	83.7 (21.7) [100.0]
					Role functioning	85.5 (24.1) [100.0]
					Emotional functioning	83.3 (19.2) [83.3]
					Cognitive functioning	83.3 (19.2) [83.3]
					Social functioning	78.6 (24.0) [83.3]
					Fatigue	23.9 (21.6) [22.2]
					Nausea and vomiting	3.5 (9.8) [0.0]
					Pain	18.4 (24.1) [0.0]
					Dyspnea	9.9 (20.5) [0]
					Insomnia	30.3 (29.0) [33.3]
					Appetite loss	6.2 (16.7) [0]
					Constipation	14.3 (24.1) [0]
					Diarrhea	9.4 (20.1) [0]
					Financial difficulties	16.0 (27.4) [0]
	MIBC	NR	270	EORTC QLQ-C30	General, mean (SD) [median]	83.3 (12.9) [85.0]
					Global health status/QOL	72.8 (19.4) [75.0]
					Physical functioning	80.5 (21.0) [80.0]
					Role functioning	85.2 (20.3) [100.0]
					Emotional functioning	82.6 (17.3) [83.3]

**Study name**	**Patient population**	**Timepoint**	**N**	**QoL scale**	**QoL domain**	**Score Mean (SD) [median]**
					Cognitive functioning	82.6 (17.3) [83.3]
					Social functioning	76.1 (23.6) [83.3]
					Fatigue	26.8 (20.9) [22.2]
					Nausea and vomiting	5.9 (14.2) [0.0]
					Pain	16.3 (22.4) [0]
					Dyspnea	14.0 (22.6) [0]
					Insomnia	29.5 (30.1) [33.3]
					Appetite loss	11.3 (20.7) [0]
					Constipation	17.4 (25.1) [0]
					Diarrhea	11.6 (21.0) [0]
					Financial difficulties	17.7 (25.6) [0]
	Non-recurrent NMIBC	NR	306	BCI scale mean (SD) [median]	Urinary Summary Score (n = 823)	89.0 (13.8) [93.8]
					Function	84.1 (22.3) [100.0]
					Bother	91.4 (12.7) [96.9]
					Bowel Summary Score (n = 833)	61.8 (10.5) [65.5]
					Function	44.7 (9.3) [50.5]
					Bother	73.4 (12.8) [79.3]
					Sexual Summary Score (n = 756)	53.3 (24.8) [54.5]
					Function	41.8 (28.0) [42.3]
					Bother	69.3 (27.7) [75]
	Recurrent NMIBC	NR	272	BCI scale mean (SD) [median]*	Urinary Summary Score (n = 823)	86.7 (14.8) [91.7]
					Function	82.2 (24.0) [95.9]
					Bother	88.8 (13.8) [93.7]
					Bowel Summary Score (n = 833)	59.7 (11.4) [63]
					Function	43.1 (11.4) [50.5]

Study name	Patient population	Timepoint	N	QoL scale	QoL domain	Score Mean (SD) [median]
					Bother	70.8 (13.8) [75.2]
					Sexual Summary Score (n = 756)	49.9 (26.3) [48.6]
					Function	41.0 (28.0) [40.4]
					Bother	62.4 (29.7) [65]
	MIBC	NR	270	BCI scale mean (SD) [median]	Urinary Summary Score (n = 823)	81.9 (17.3) [87.5]
					Function	76.1 (27.5) [83.5]
					Bother	84.9 (15.4) [90.6]
					Bowel Summary Score (n = 833)	58 (12.3) [60.6]
					Function	41.8 (11.6) [44.7]
					Bother	68.8 (14.8) [71.2]
					Sexual Summary Score (n = 756)	41.3 (23.8) [40]
					Function	31.6 (25.8) [26.1]
					Bother	54.9 (28.7) [50]
	Non-recurrent NMIBC	NR	306	Comprehensive score for Financial toxicity*, Mean (SD) [Median]	Cost	13.1 (9.7) [12]
	Recurrent NMIBC	NR	272			13.6 (10.5) [12]
	MIBC	NR	270			14.0 (9.9) [12]
	Non-recurrent NMIBC	NR	306	Work productivity and activity impairment*, %, mean (SD) [median]	Absenteeism	3.1 (8.3) [0]
					Presenteesim	10.8 (17.2) [0]
					Total work impairment	13.4 (19.4) [8.75]
					Total activity impairment	11.6 (20.7) [0]
		NR	272		Absenteeism	5.1 (14.4) [0

Study name	Patient population	Timepoint	N	QoL scale	QoL domain	Score Mean (SD) [median]
	Recurrent NMIBC			Work productivity and activity impairment*, %, mean (SD) [median]n]	Presenteesim	11.5 (18.0) [0]
					Total work impairment	15.7 (23.0) [0]
					Total activity impairment	16.4 (24.2) [0]
	MIBC	NR	270	Work productivity and activity impairment*, %, mean (SD) [median]	Absenteeism	11.1 (25.5) [0]
					Presenteesim	16.4 (21.6) [10]
					Total work impairment	19.6 (26.2) [10]
					Total activity impairment	20.1 (26.5) [10]

Note: none of the studies reported results for proportion of patients, difference or p-values. * Comprehensive score for financial toxicity and Work productivity and activity impairment reported as outcomes of questionnaires Abbreviations: BCI: Bacillus Calmette Guerin; EORTC: European Organisation for Research and Treatment of Cancer; MIBC: muscle invasive bladder cancer; NMIBC: non-muscle invasive bladder cancer; NR: not reported; QoL: quality of life; SD: standard deviation.

Gastric cancer

Table 37. Fear of recurrence outcomes in patients with gastric cancer (n = 1 study)

Study name	Patient population	Timepoint	N	QoL scale	QoL domain	Score	*p*-value
Shin 2022 (75)	Gastric cancer survivors, overall	NR	363	FCRI	Total score	Mean (SD): 58.3 (24.3)	<0.001
				FCRI subscale	Triggers	Mean (SD): 13.1 (7.1)	
					Severity	Mean (SD): 11.5 (7.2)	
					Psychological distress	Mean (SD): 4.2 (4)	
					Functioning impairments	Mean (SD): 4.8 (5.4)	
					Insight	Mean (SD): 1.3 (2.1)	
					Reassurance	Mean (SD): 4.8 (3.3)	0.008
					Coping strategies	Mean (SD): 18.6 (7.6)	0.508
	Gastric cancer survivors, non-FCRI group		221	FCRI	Total score	Mean (SD): 45.5 (17.2)	<0.001
				FCRI subscale	Triggers	Mean (SD): 9.8 (5.7)	
					Severity	Mean (SD): 6.7 (3.6)	
					Psychological distress	Mean (SD): 2.4 (2.9)	
					Functioning impairments	Mean (SD): 3.4 (4.6)	
					Insight	Mean (SD): 0.5 (1)	
					Reassurance	Mean (SD): 4.4 (3.4)	0.008
					Coping strategies	Mean (SD): 18.4 (8.1)	0.508
	Gastric cancer survivors, FCRI group		142	FCRI	Total score	Mean (SD): 78.1 (20)	<0.001
				FCRI subscale	Triggers	Mean (SD): 18.2 (6)	
					Severity	Mean (SD): 18.9 (4.6)	
					Psychological distress	Mean (SD): 7.1 (3.8)	
					Functioning impairments	Mean (SD): 7 (5.7)	
					Insight	Mean (SD): 2.7 (2.5)	

Study name	Patient population	Timepoint	N	QoL scale	QoL domain	Score	*p*-value
					Reassurance	Mean (SD): 5.4 (3.1)	0.008
					Coping strategies	Mean (SD): 18.9 (6.9)	0.508
	Gastric cancer survivors, overall	NR	363	EQ VAS	Overall	Mean (SD): 68.9 (16.4)	NR
				EORTC QLQ-C30	Global health status	Mean (SD): 66.1 (17.3)	
					Functional scales (Physical functioning)	Mean (SD): 81.7 (15.3)	
					Functional scales (Role functioning)	Mean (SD): 86.9 (18.7)	
					Functional scales (Emotional functioning)	Mean (SD): 79.4 (18.4)	
					Functional scales (Cognitive functioning)	Mean (SD): 76.8 (17.8)	
					Functional scales (Social functioning)	Mean (SD): 80.2 (22.8)	
					Symptom scales (Fatigue)	Mean (SD): 34.9 (23.4)	
					Symptom scales (Nausea and vomiting)	Mean (SD): 12.8 (17)	
					Symptom scales (Pain)	Mean (SD): 14.2 (18.7)	
					Symptom scales (Dyspnea)	Mean (SD): 16 (21.7)	
					Symptom scales (Insomnia)	Mean (SD): 27.3 (31.6)	
					Symptom scales (Appetite loss)	Mean (SD): 13.9 (21.5)	
					Symptom scales (Constipation)	Mean (SD): 17.5 (23.1)	
					Symptom scales (Diarrhea)	Mean (SD): 31.8 (26.4)	
					Symptom scales (Financial difficulties)	Mean (SD): 18.4 (24.7)	

Study name	Patient population	Timepoint	N	QoL scale	QoL domain	Score	*p*-value
				Fatigue severity scale	NR	Mean (SD): 2.8 (1.7)	
				Self-administered questionnaire	Sleep problems	Mean (SD): 2 (1.1)	
				Hospital Anxiety and Depression Scale (HADS)	Anxiety	Mean (SD): 4.7 (3.3)	
					Depression	Mean (SD): 8 (3.6)	
				NR	Communication unsatisfaction	Mean (SD): 4.3 (1.8)	
					Care coordination	Mean (SD): 1.7 (0.9)	
					Functional social support questionnaire (Functional social support)	Mean (SD): 3.0 (1.0)	
					Confidant support	Mean (SD): 15.0 (3.9)	
					Affective support	Mean (SD): 11.2 (2.9)	
	Gastric cancer survivors, non-clinical FCRI		221	EQ VAS	Overall	Mean (SD): 70.8 (15.7)	
				EORTC QLQ-C30	Global health status	Mean (SD): 67.9 (16.7)	
					Functional scales (Physical functioning)	Mean (SD): 84.1 (14.4)	
					Functional scales (Role functioning)	Mean (SD): 89.5 (16.7)	
					Functional scales (Emotional functioning)	Mean (SD): 84.5 (15.7)	
					Functional scales (Cognitive functioning)	Mean (SD): 79.4 (16.4)	

Study name	Patient population	Timepoint	N	QoL scale	QoL domain	Score	*p*-value
					Functional scales (Social functioning)	Mean (SD): 84.7 (18.4)	
					Symptom scales (Fatigue)	Mean (SD): 29.9 (21.9)	
					Symptom scales (Nausea and vomiting)	Mean (SD): 10.6 (17.1)	
					Symptom scales (Pain)	Mean (SD): 11.4 (17.9)	
					Symptom scales (Dyspnea)	Mean (SD): 13 (19.7)	
					Symptom scales (Insomnia)	Mean (SD): 23.9 (31.4)	
					Symptom scales (Appetite loss)	Mean (SD): 11.9 (21.2)	
					Symptom scales (Constipation)	Mean (SD): 16.3 (22.2)	
					Symptom scales (Diarrhea)	Mean (SD): 28.7 (25.1)	
					Symptom scales (Financial difficulties)	Mean (SD): 15.5 (22.4)	
				Fatigue severity scale	NR	Mean (SD): 2.4 (1.5)	
				Self-administered questionnaire	Sleep problems	Mean (SD): 1.9 (1.1)	
				Hospital Anxiety and Depression Scale (HADS)	Anxiety	Mean (SD): 3.5 (2.5)	
					Depression	Mean (SD): 7.4 (3.5)	
				NR	Communication unsatisfaction	Mean (SD): 4.1 (1.8)	

Study name	Patient population	Timepoint	N	QoL scale	QoL domain	Score	*p*-value
					Care coordination	Mean (SD): 1.6 (0.8)	
					Functional social support questionnaire (Functional social support)	Mean (SD): 3.2 (1.7)	
					Confidant support	Mean (SD): 16.0 (3.6)	
					Affective support	Mean (SD): 12.3 (2.5)	
	Gastric cancer survivors, clinical FCRI		142	EQ VAS	Overall	Mean (SD): 66.6 (16.6)	
				EORTC QLQ-C30	Global health status	Mean (SD): 63.6 (17.4)	
					Functional scales (Physical functioning)	Mean (SD): 78 (15.2)	
					Functional scales (Role functioning)	Mean (SD): 83.2 (21)	
					Functional scales (Emotional functioning)	Mean (SD): 70.9 (19.2)	
					Functional scales (Cognitive functioning)	Mean (SD): 72.1 (18.3)	
					Functional scales (Social functioning)	Mean (SD): 72.7 (26.1)	
					Symptom scales (Fatigue)	Mean (SD): 43 (23.4)	
					Symptom scales (Nausea and vomiting)	Mean (SD): 16.8 (17.8)	
					Symptom scales (Pain)	Mean (SD): 18.6 (19.4)	
					Symptom scales (Dyspnea)	Mean (SD): 20.4 (23.1)	
					Symptom scales (Insomnia)	Mean (SD): 32.2 (30.6)	
					Symptom scales (Appetite loss)	Mean (SD): 17.1 (22.4)	

Study name	Patient population	Timep oint	N	QoL scale	QoL domain	Score	*p*-value
					Symptom scales (Constipation)	Mean (SD): 19.7 (24.8)	
					Symptom scales (Diarrhea)	Mean (SD): 36.9 (27.7)	
					Symptom scales (Financial difficulties)	Mean (SD): 23 (27.6)	
				Fatigue severity scale	NR	Mean (SD): 3.4 (1.8)	
				Self-administered questionnaire	Sleep problems	Mean (SD): 2.1 (1.1)	
				Hospital Anxiety and Depression Scale (HADS)	Anxiety	Mean (SD): 6.5 (3.5)	
					Depression	Mean (SD): 8.9 (3.6)	
				NR	Communication unsatisfaction	Mean (SD): 4.6 (1.8)	NR
					Care coordination	Mean (SD): 1.9 (0.9)	
					Functional social support questionnaire (Functional social support)	Mean (SD): 2.5 (1.5)	
					Confidant support	Mean (SD): 13.4 (3.8)	
					Affective support	Mean (SD): 9.8 (2.7)	

Abbreviations: EQ-VAS: European questionnaire-vertical visual analogue; EORTC: European Organisation for Research and Treatment of Cancer; FCRI: Fear of Cancer Recurrence Inventory; NR: not reported SD: standard deviation.

Melanoma

Table 38. Fear of recurrence in patient with melanoma (n = 3 studies)

Study name	Patient population	Timepoint	N	QoL scale	QoL domain	Score	Proportion of patients, n (%)	*p*-value
Leclair 2019 (73)	Melanoma survivors (stage I-III)	NR	139	–	Low FCR	NR	46 (33.1)	NR
					Moderate FCR		87 (62.6)	
					High FCR		6 (4.3)	
Van de Wal 2016 (76)	Early stage or locally advanced disease (stage I/II) melanoma	NR	469	Impact of Cancer scale- Health Worries subscale (IOC-HWS)	Fear of cancer recurrence	Mean (SD): 2.69 (0.96)	NR	NR
Atkinson 2013 (77)	Melanoma, Women within ten days to two years of completing surgical treatment for primary cutaneous melanoma	NR	100	Concerns about recurrence scale	Fear of cancer recurrence	Mean (SE): 3.16 (0.13)	NR	NR

Abbreviations: FCR: fear of cancer recurrence; IOC-HWS: Impact of Cancer scale- Health Worries subscale; NR: not reported; QoL: quality of life; SD: Standard deviation; SE: Standard error

Appendix S5. Economic review findings overview

Table 39: Economic outcomes in patients included in HNC studies (n = 1 study)

Study name	Patient population	Stage at diagnosis	N	Currency	Description of cost	Cost item	Cost	*p*-value
Kim 2012 (39)	Recurrent locally advanced head and neck cancer	Locally advanced	324	USD	Direct healthcare costs per patient for 6-months	Total cost*	25837	<0.0001
	Control (Cancer free patients)						2752	
	Adjusted difference between head and neck cancer and controls						Difference: 21,141	
	Recurrent locally advanced head and neck cancer					Pharmacy costs	3503	<0.0001
	Control (Cancer free patients)						968	
	Adjusted difference between head and neck cancer and controls						Difference: 2,309	
	Recurrent locally advanced head and neck cancer					Medical costs**	22334	<0.0001
	Control (Cancer free patients)						1784	
	Adjusted difference between head and neck cancer and controls						Difference: 19,199	
	Recurrent locally advanced head and neck cancer					Inpatient costs	6986	<0.0001
	Control (Cancer free patients)						702	
	Adjusted difference between head and neck cancer and controls						Difference: 5,682	
	Recurrent locally advanced head and neck cancer					Outpatient costs	13902	<0.0001
	Control (Cancer free patients)						954	

Study name	Patient population	Stage at diagnosis	N	Currency	Description of cost	Cost item	Cost	*p*-value
	Adjusted difference between head and neck cancer and controls						Difference: 12,323	
	Recurrent locally advanced head and neck cancer					Emergency room costs	601	<0.0001
	Control (Cancer free patients)						33	
	Adjusted difference between head and neck cancer and controls						Difference: 575	
	Recurrent locally advanced head and neck cancer					Supportive care costs	342	<0.0001
	Control (Cancer free patients)						24	
	Adjusted difference between head and neck cancer and controls						Difference: 328	
	Recurrent locally advanced head and neck cancer					Other medical costs***	502	<0.0001
	Control (Cancer free patients)						71	
	Adjusted difference between head and neck cancer and controls						Difference: 389	

*Total cost is the sum of medical cost and pharmacy cost

**Medical cost is the sum of inpatient, outpatient, emergency room costs, supportive costs and other medical costs

***other medical cost consisted of expenses for medical services that are not included in inpatient, outpatient, emergency room, or supportive care services. E.g., home visits, skilled nursing facility services, etc.

Abbreviations: HNC: head and neck cancer; TNM: tumour, nodes, and metastases; NR: not reported; USD: united states dollar.

Table 40: HCRU outcomes in patients included in HNC studies (n = 1 study)

Study name	Patient population	Stage at diagnosis	N	HCRU item	Resource use	*p*-value
Kim 2012 (39)	Recurrent locally advanced head and neck cancer	Locally advanced	324	Inpatient visits, rate of utilization	22.8%	<0.0001
	Control (Cancer free patients)		324	Inpatient visits, rate of utilization	6.5%	
	Recurrent locally advanced head and neck cancer		324	Outpatient visits, rate of utilization	97.8%	NR
	Control (Cancer free patients)		324	Outpatient visits, rate of utilization	74.1%	NR
	Recurrent locally advanced head and neck cancer		324	Emergency room visits, rate of utilization	17%	<0.0001
	Control (Cancer free patients)		324	Emergency room visits, rate of utilization	4.6%	
	Recurrent locally advanced head and neck cancer		324	Supportive care, rate of utilization	9.3%	<0.0001
	Control (Cancer free patients)		324	Supportive care, rate of utilization	1.9%	
	Recurrent locally advanced head and neck cancer		324	Others, rate of utilization	45.7%	<0.0001
	Control (Cancer free patients)		324	Others, rate of utilization	18.8%	

	Recurrent locally advanced head and neck cancer		324	Inpatient visits	Mean visits: 0.3	NR
	Control (Cancer free patients)		324	Inpatient visits	Mean visits: 0.1	NR
	Recurrent locally advanced head and neck cancer		324	Outpatient visits	Mean visits: 17.5	NR
	Control (Cancer free patients)		324	Outpatient visits	Mean visits: 4.4	NR
	Recurrent locally advanced head and neck cancer		324	Emergency room visits	Mean visits: 0.3	NR
	Control (Cancer free patients)		324	Emergency room visits	Mean visits: 0.1	NR
	Recurrent locally advanced head and neck cancer		324	Supportive care, visits	Mean visits: 0.8	NR
	Control (Cancer free patients)		324	Supportive care, visits	Mean visits: 0.1	NR
	Recurrent locally advanced head and neck cancer		324	Others, visits	Mean visits: 1.9	NR
	Control (Cancer free patients)		324	Others, visits	Mean visits: 0.5	NR

Abbreviations: HNC: head and neck cancer; NR: not reported, HRCU: healthcare resource utilization

Table 41: HCRU of recurrent locally advanced HNC patients and their controls during the 6-month study period (n = 1 study)

Study name	Patient population	Stage at diagnosis	N	Resource use	Estimate (95% CI)	*p*-value
Kim 2012 (39)	Recurrent locally advanced head and neck cancer vs. Controls	Locally advanced	324	Inpatient visit rate of utilization in recurrent locally advanced patients vs. controls	OR: 4.7 (2.6-8.7)	<0.0001
				Emergency room visits, rate of utilization in recurrent locally advanced patients vs. controls	OR: 4.9 (2.3-10.1)	<0.0001
				Supportive care, rate of utilization in recurrent locally advanced patients vs. controls	OR: 4.7 (1.8-11.7)	<0.0001
				Others, rate of utilization in recurrent locally advanced patients vs. controls	OR: 3.7 (2.4-5.6)	<0.0001
				Inpatient visits in recurrent locally advanced patients vs. controls	IRR: 4.3 (2.6-7)	<0.0001
				Outpatient visits in recurrent locally advanced patients vs. controls	IRR: 3.9 (3.3-4.6)	<0.0001
				Emergency room visits in recurrent locally advanced patients vs. controls	IRR: 4.7 (2.6-8.7)	<0.0001
				Supportive care, visits in recurrent locally advanced patients vs. controls	IRR: 7.8 (2.3-26.5)	<0.0001
				Others, visits in recurrent locally advanced patients vs. controls	IRR: 3.1 (2.2-4.5)	<0.0001

Abbreviations: HNC: head and neck cancer; IRR: incident rate ratios; OR: odds ratios, CI: confidence intervals.

Table 42: Economic outcomes in patients included in melanoma studies (n = 4 studies)

Study name	Patient population	Stage of disease	N	Currency	Description of cost	Oncology specific cost	Cost item	Unit	Cost	*p*-value
Leeneman 2021 (78)	Healthcare cost for full disease course-Localized-patients without recurrence	Localized melanoma	54	Euro	Healthcare costs	NR	Total Cost	NR	Mean (SD): 3032 (2338) Median (IQR): 2579 (IQR: (€251–€11 509)	NR
			54	Euro	Healthcare costs	NR	Medical imaging	NR	Mean (SD): 105 (250)	NR
			54	Euro	Healthcare costs	NR	Pathology	NR	Mean (SD): 191 (108)	NR
			54	Euro	Healthcare costs	NR	Hospital visits	NR	Mean (SD): 1578 (998)	NR
			54	Euro	Healthcare costs	NR	Hospital admissions	NR	Mean (SD): 642 (1012)	NR
			54	Euro	Healthcare costs	NR	Surgery	NR	Mean (SD): 374 (266)	NR
			54	Euro	Healthcare costs	NR	Radiotherapy	NR	Mean (SD): 142 (1043)	NR
			54	Euro	Healthcare costs	NR	Systemic therapy	NR	Mean (SD): 0 (0)	NR
	Healthcare cost for full disease course-Localized-patients with recurrence	Localized melanoma	144	Euro	Healthcare costs	NR	Total Cost	NR	Mean (SD): 20007 (20284) Median (IQR): 14887 (IQR: €685-€130901)	NR
			144	Euro	Healthcare costs	NR	Medical imaging	NR	Mean (SD): 1382 (1218)	NR

Study name	Patient population	Stage of disease	N	Currency	Description of cost	Oncology specific cost	Cost item	Unit	Cost	*p*-value
			144	Euro	Healthcare costs	NR	Pathology	NR	Mean (SD): 385(221)	NR
			144	Euro	Healthcare costs	NR	Hospital visits	NR	Mean (SD): 3974(€ 2456)	NR
			144	Euro	Healthcare costs	NR	Hospital admissions	NR	Mean (SD): 6029(€ 7005)	NR
			144	Euro	Healthcare costs	NR	Surgery	NR	Mean (SD): 2175(€ 2169)	NR
			144	Euro	Healthcare costs	NR	Radiotherapy	NR	Mean (SD): 3283(€ 5791)	NR
			144	Euro	Healthcare costs	NR	Systemic therapy	NR	Mean (SD): 2778(13608)	NR
	Healthcare cost for full disease course-Regionally advanced-Patients without recurrence	Regionally advanced melanoma	51	Euro	Healthcare costs	NR	Total cost	NR	Mean (SD): 5951(4575) Median (IQR): 4484(€1270–€25 400)	NR
			51	Euro	Healthcare costs	NR	Medical imaging	NR	Mean (SD): 232(382)	NR
			51	Euro	Healthcare costs	NR	Pathology	NR	Mean (SD): 204(131)	NR
			51	Euro	Healthcare costs	NR	Hospital visits	NR	Mean (SD): 1438(1369)	NR
			51	Euro	Healthcare costs	NR	Hospital admissions	NR	Mean (SD): 1971(3154)	NR
			51	Euro	Healthcare costs	NR	Surgery	NR	Mean (SD): 1956(837)	NR

Study name	Patient population	Stage of disease	N	Currency	Description of cost	Oncology specific cost	Cost item	Unit	Cost	*p*-value
			51	Euro	Healthcare costs	NR	Radiotherapy	NR	Mean (SD): 150(1074)	NR
			51	Euro	Healthcare costs	NR	Systemic therapy	NR	Mean (SD): 0(0)	NR
	Healthcare cost for full disease course-Regionally advanced-Patients with recurrence	Regionally advanced melanoma	47	Euro	Healthcare costs	NR	Total cost	NR	Mean (SD): 19519(12947) Median (IQR): 17530(IQR: €2081-€52709)	NR
			47	Euro	Healthcare costs	NR	Medical imaging	NR	Mean (SD): 1477(2085)	NR
			47	Euro	Healthcare costs	NR	Pathology	NR	Mean (SD): 404(283)	NR
			47	Euro	Healthcare costs	NR	Hospital visits	NR	Mean (SD): 3542(3286)	NR
			47	Euro	Healthcare costs	NR	Hospital admissions	NR	Mean (SD): 8009(8318)	NR
			47	Euro	Healthcare costs	NR	Surgery	NR	Mean (SD): 3191(2706)	NR
			47	Euro	Healthcare costs	NR	Radiotherapy	NR	Mean (SD): 2489(4532)	NR
			47	Euro	Healthcare costs	NR	Systemic therapy	NR	Mean (SD): 407(2423)	NR
		Localized melanoma	54	Euro	Healthcare costs	NR	Total cost	NR	Mean (SD): 3032(2338) Median (IQR): 2579(IQR: €251–€11 509)	NR

Study name	Patient population	Stage of disease	N	Currenc y	Descripti on of cost	Oncology specific cost	Cost item	Unit	Cost	*p*-value
	Healthcare costs of the initial treatment episode- Localized- patients without recurrence		54	Euro	Medical imaging	NR	Medical imaging	NR	Mean (SD): 105(250)	NR
			54	Euro	Medical imaging	NR	X-ray	NR	Mean (SD): 15(32)	NR
			54	Euro	Medical imaging	NR	Ultrasound	NR	Mean (SD): 45(82)	NR
			54	Euro	Medical imaging	NR	CT scan	NR	Mean (SD): 34(154)	NR
			54	Euro	Medical imaging	NR	MRI scan	NR	Mean (SD): 10(76)	NR
			54	Euro	Medical imaging	NR	PET/CT scan	NR	Mean (SD): 0(0)	NR
			54	Euro	Pathology	NR	Pathology	NR	Mean (SD): 191(108)	NR
			54	Euro	Pathology	NR	Cytology/hist ology	NR	Mean (SD): 191(108)	NR
			54	Euro	Hospital visits	NR	Hospital visits	NR	Mean (SD): 1578(998)	NR
			54	Euro	Hospital visits	NR	Consultation by telephone	NR	Mean (SD): 18(28)	NR
			54	Euro	Hospital visits	NR	Emergency room visit	NR	Mean (SD): 10(51)	NR
			54	Euro	Hospital visits	NR	Outpatient visit	NR	Mean (SD): 1524(987)	NR
			54	Euro	Hospital visits	NR	Daycare treatment	NR	Mean (SD): 27(101)	NR

Study name	Patient population	Stage of disease	N	Currency	Description of cost	Oncology specific cost	Cost item	Unit	Cost	*p*-value
			54	Euro	Hospital admissions	NR	Hospital admissions	NR	Mean (SD): 642(1012)	NR
			54	Euro	Hospital admissions	NR	Inpatient hospital day	NR	Mean (SD): 642(1012)	NR
			54	Euro	Hospital admissions	NR	ICU day	NR	Mean (SD): 0(0)	NR
			54	Euro	Surgery	NR	Surgery	NR	Mean (SD): 374(266)	NR
			54	Euro	Surgery	NR	Biopsy	NR	Mean (SD): 16(58)	NR
			54	Euro	Surgery	NR	Excision	NR	Mean (SD): 264(139)	NR
			54	Euro	Surgery	NR	Amputation	NR	Mean (SD): 0(0)	NR
			54	Euro	Surgery	NR	Sentinel lymph node biopsy	NR	Mean (SD): 94(213)	NR
			54	Euro	Surgery	NR	Lymph node dissection	NR	Mean (SD): 0(0)	NR
			54	Euro	Surgery	NR	Isolated limb perfusion	NR	Mean (SD): 0(0)	NR
			54	Euro	Radiotherapy	NR	Radiotherapy	NR	Mean (SD): 142(1043)	NR
			54	Euro	Radiotherapy	NR	Short course (≤6 sessions)	NR	Mean (SD): 0(0)	NR

Study name	Patient population	Stage of disease	N	Currency	Description of cost	Oncology specific cost	Cost item	Unit	Cost	*p*-value
			54	Euro	Radiotherapy	NR	Standard course (>6 sessions)	NR	Mean (SD): 142(1043)	NR
	Healthcare costs of the initial treatment episode-Localized-patients with recurrence	Localized melanoma	144	Euro	Healthcare costs	NR	Total cost	NR	Mean (SD): 3015(2078) Median (IQR): 2392(IQR: €342-€12432)	NR
			144	Euro	Medical imaging	NR	Overall	NR	Mean (SD): 148(285)	NR
			144	Euro	Medical imaging	NR	X-ray	NR	Mean (SD): € 20(€ 47)	NR
			144	Euro	Medical imaging	NR	Ultrasound	NR	Mean (SD): € 60(€ 101)	NR
			144	Euro	Medical imaging	NR	CT scan	NR	Mean (SD): € 29(€ 81)	NR
			144	Euro	Medical imaging	NR	MRI scan	NR	Mean (SD): € 10(€ 61)	NR
			144	Euro	Medical imaging	NR	PET/CT scan	NR	Mean (SD): € 30(€ 176)	NR
			144	Euro	Pathology	NR	Cytology/histology	NR	Mean (SD): € 182(€ 90)	NR
			144	Euro	Hospital visits	NR	Overall	NR	Mean (SD): € 1447(€ 934)	NR
			144	Euro	Hospital visits	NR	Consultation by telephone	NR	Mean (SD): € 7(€ 16)	NR

Study name	Patient population	Stage of disease	N	Currency	Description of cost	Oncology specific cost	Cost item	Unit	Cost	*p*-value
			144	Euro	Hospital visits	NR	Emergency room visit	NR	Mean (SD): € 26(€ 112)	NR
			144	Euro	Hospital visits	NR	Outpatient visit	NR	Mean (SD): € 1328(€ 846)	NR
			144	Euro	Hospital visits	NR	Daycare treatment	NR	Mean (SD): € 86(€ 192)	NR
			144	Euro	Hospital admissions	NR	Overall	NR	Mean (SD): € 709(€ 1261)	NR
			144	Euro	Hospital admissions	NR	Inpatient hospital day	NR	Mean (SD): € 709(€ 1261)	NR
			144	Euro	Hospital admissions	NR	ICU day	NR	Mean (SD): 0(0)	NR
			144	Euro	Surgery	NR	Overall	NR	Mean (SD): 456(479)	NR
			144	Euro	Surgery	NR	Biopsy	NR	Mean (SD): € 12(€ 44)	NR
			144	Euro	Surgery	NR	Excision	NR	Mean (SD): € 242(€ 121)	NR
			144	Euro	Surgery	NR	Amputation	NR	Mean (SD): € 60(€ 359)	NR
			144	Euro	Surgery	NR	Sentinel lymph node biopsy	NR	Mean (SD): € 141(€ 246)	NR
			144	Euro	Surgery	NR	Lymph node dissection	NR	Mean (SD): € 0(€ 0)	NR

Study name	Patient population	Stage of disease	N	Currency	Description of cost	Oncology specific cost	Cost item	Unit	Cost	*p*-value
			144	Euro	Surgery	NR	Isolated limb perfusion	NR	Mean (SD): € 0(€ 0)	NR
			144	Euro	Radiotherapy	NR	Overall	NR	Mean (SD): € 73(€ 680)	NR
			144	Euro	Radiotherapy	NR	Short course (≤6 sessions)	NR	Mean (SD): € 20(€ 238)	NR
			144	Euro	Radiotherapy	NR	Standard course (>6 sessions)	NR	Mean (SD): € 53(€ 639)	NR
	Healthcare costs of the initial treatment episode- Regionally advanced melanoma - patients without recurrence	Regionally advanced melanoma	50	Euro	Healthcare costs	NR	Total cost	NR	Mean (SD): €5951 (4575) Median (IQR): €4484 (€1270–€25 400)	NR
			50	Euro	Medical imaging	NR	Medical imaging	NR	Mean (SD): 232(382)	NR
			50	Euro	Medical imaging	NR	X-ray	NR	Mean (SD): 34(95)	NR
			50	Euro	Medical imaging	NR	Ultrasound	NR	Mean (SD): 64(105)	NR
			50	Euro	Medical imaging	NR	CT scan	NR	Mean (SD): 39(80)	NR
			50	Euro	Medical imaging	NR	MRI scan	NR	Mean (SD): 11(55)	NR
			50	Euro	Medical imaging	NR	PET/CT scan	NR	Mean (SD): 84(290)	NR
			50	Euro	Pathology	NR	Pathology	NR	Mean (SD): 204(131)	NR

Study name	Patient population	Stage of disease	N	Currency	Description of cost	Oncology specific cost	Cost item	Unit	Cost	*p*-value
			50	Euro	Pathology	NR	Cytology/histology	NR	Mean (SD): 204(131)	NR
			50	Euro	Hospital visits	NR	Hospital visits	NR	Mean (SD): 1438(1369)	NR
			50	Euro	Hospital visits	NR	Consultation by telephone	NR	Mean (SD): 6(16)	NR
			50	Euro	Hospital visits	NR	Emergency room visit	NR	Mean (SD): 32(158)	NR
			50	Euro	Hospital visits	NR	Outpatient visit	NR	Mean (SD): 1372(1333)	NR
			50	Euro	Hospital visits	NR	Daycare treatment	NR	Mean (SD): 28(104)	NR
			50	Euro	Hospital admissions	NR	Hospital admissions	NR	Mean (SD): 1971(3154)	NR
			50	Euro	Hospital admissions	NR	Inpatient hospital day	NR	Mean (SD): 1971(3154)	NR
			50	Euro	Hospital admissions	NR	ICU day	NR	Mean (SD): 0(0)	NR
			50	Euro	Surgery	NR	Surgery	NR	Mean (SD): 1956(837)	NR
			50	Euro	Surgery	NR	Biopsy	NR	Mean (SD): 38(81)	NR
			50	Euro	Surgery	NR	Excision	NR	Mean (SD): 236(144)	NR
			50	Euro	Surgery	NR	Amputation	NR	Mean (SD): 0(0)	NR

Study name	Patient population	Stage of disease	N	Currency	Description of cost	Oncology specific cost	Cost item	Unit	Cost	*p*-value
			50	Euro	Surgery	NR	Sentinel lymph node biopsy	NR	Mean (SD): 322(283)	NR
			50	Euro	Surgery	NR	Lymph node dissection	NR	Mean (SD): 1360(721)	NR
			50	Euro	Surgery	NR	Isolated limb perfusion	NR	Mean (SD): 0(0)	NR
			50	Euro	Radiotherapy	NR	Radiotherapy	NR	Mean (SD): 150(1074)	NR
			50	Euro	Radiotherapy	NR	Short course (≤6 sessions)	NR	Mean (SD): 0(0)	NR
			50	Euro	Radiotherapy	NR	Standard course (>6 sessions)	NR	Mean (SD): 150(1074)	NR
	Healthcare costs of the initial treatment episode-Regionally advanced-Patients with recurrence	Regionally advanced melanoma	47	Euro	Total cost	NR	Total cost	NR	Mean (SD): 7648(6975) Median (IQR): 6175(€924-€40569)	NR
			47	Euro	Medical imaging	NR	Medical imaging	NR	Mean (SD): 330(377)	NR
			47	Euro	Medical imaging	NR	X-ray	NR	Mean (SD): € 55(€ 105)	NR
			47	Euro	Medical imaging	NR	Ultrasound	NR	Mean (SD): € 91(€ 118)	NR
			47	Euro	Medical imaging	NR	CT scan	NR	Mean (SD): € 98(€ 158)	NR

Study name	Patient population	Stage of disease	N	Currency	Description of cost	Oncology specific cost	Cost item	Unit	Cost	*p*-value
			47	Euro	Medical imaging	NR	MRI scan	NR	Mean (SD): € 42(€ 117)	NR
			47	Euro	Medical imaging	NR	PET/CT scan	NR	Mean (SD): € 46(€ 218)	NR
			47	Euro	Pathology	NR	Cytology/histology	NR	Mean (SD): € 200(€ 102)	NR
			47	Euro	Hospital visits	NR	Hospital visits	NR	Mean (SD): € 1417(€ 1260)	NR
			47	Euro	Hospital visits	NR	Consultation by telephone	NR	Mean (SD): € 12(€ 28)	NR
			47	Euro	Hospital visits	NR	Emergency room visit	NR	Mean (SD): € 29(€ 116)	NR
			47	Euro	Hospital visits	NR	Outpatient visit	NR	Mean (SD): € 1291(€ 1147)	NR
			47	Euro	Hospital visits	NR	Daycare treatment	NR	Mean (SD): € 86(€ 179)	NR
			47	Euro	Hospital admissions	NR	Hospital admissions	NR	Mean (SD): € 2988(€ 5555)	NR
			47	Euro	Hospital admissions	NR	Inpatient hospital day	NR	Mean (SD): € 2961(€ 5538)	NR
			47	Euro	Hospital admissions	NR	ICU day	NR	Mean (SD): € 26(€ 180)	NR
			47	Euro	Surgery	NR	Surgery	NR	Mean (SD): € 2102(€ 1234)	NR

Study name	Patient population	Stage of disease	N	Currency	Description of cost	Oncology specific cost	Cost item	Unit	Cost	*p*-value
			47	Euro	Surgery	NR	Biopsy	NR	Mean (SD): € 33(€ 78)	NR
			47	Euro	Surgery	NR	Excision	NR	Mean (SD): € 185(€ 70)	NR
			47	Euro	Surgery	NR	Amputation	NR	Mean (SD): € 46(€ 317)	NR
			47	Euro	Surgery	NR	Sentinel lymph node biopsy	NR	Mean (SD): 337(281)	NR
			47	Euro	Surgery	NR	Lymph node dissection	NR	Mean (SD): € 1329(€ 902)	NR
			47	Euro	Surgery	NR	Isolated limb perfusion	NR	Mean (SD): € 172(€ 826)	NR
			47	Euro	Radiotherapy	NR	Radiotherapy	NR	Mean (SD): € 611(€ 2704)	NR
			47	Euro	Radiotherapy	NR	Short course (≤6 sessions)	NR	Mean (SD): € 121(€ 582)	NR
			47	Euro	Radiotherapy	NR	Standard course (>6 sessions)	NR	Mean (SD): € 489(€ 2479)	NR
	Healthcare costs of subsequent treatment episodes- Local recurrence	Local recurrence	13	Euro	Total cost	NR	Total cost	NR	Mean (SD): 4414(3868) Median (IQR): 3241(€747-€11794)	NR
			13	Euro	Medical imaging	NR	Overall	NR	Mean (SD): 230(391)	NR

Study name	Patient population	Stage of disease	N	Currency	Description of cost	Oncology specific cost	Cost item	Unit	Cost	*p*-value
			13	Euro	Medical imaging	NR	X-ray	NR	Mean (SD): € 14(€ 22)	NR
			13	Euro	Medical imaging	NR	Ultrasound	NR	Mean (SD): € 77(€ 110)	NR
			13	Euro	Medical imaging	NR	CT scan	NR	Mean (SD): € 35(€ 67)	NR
			13	Euro	Medical imaging	NR	MRI scan	NR	Mean (SD): € 22(€ 78)	NR
			13	Euro	Medical imaging	NR	PET/CT scan	NR	Mean (SD): € 82(€ 297)	NR
			13	Euro	Pathology	NR	Cytology/histology	NR	Mean (SD): € 183(€ 71)	NR
			13	Euro	Hospital visits	NR	Overall	NR	Mean (SD): € 1653(€ 887)	NR
			13	Euro	Hospital visits	NR	Consultation by telephone	NR	Mean (SD): € 27(€ 37)	NR
			13	Euro	Hospital visits	NR	Emergency room visit	NR	Mean (SD): € 21(€ 75)	NR
			13	Euro	Hospital visits	NR	Outpatient visit	NR	Mean (SD): € 1406(€ 705)	NR
			13	Euro	Hospital visits	NR	Daycare treatment	NR	Mean (SD): € 199(€ 396)	NR
			13	Euro	Hospital admissions	NR	Overall	NR	Mean (SD): € 1771(€ 2961)	NR
			13	Euro	Hospital admissions	NR	Inpatient hospital day	NR	Mean (SD): € 1676(€ 2842)	NR

Study name	Patient population	Stage of disease	N	Currency	Description of cost	Oncology specific cost	Cost item	Unit	Cost	*p*-value
			13	Euro	Hospital admissions	NR	ICU day	NR	Mean (SD): € 95(€ 342)	NR
			13	Euro	Surgery	NR	Overall	NR	Mean (SD): € 576(€ 1078)	NR
			13	Euro	Surgery	NR	Biopsy	NR	Mean (SD): € 22(€ 57)	NR
			13	Euro	Surgery	NR	Excision	NR	Mean (SD): € 243(€ 133)	NR
			13	Euro	Surgery	NR	Lymph node dissection	NR	Mean (SD): 0(0)	NR
			13	Euro	Surgery	NR	Isolated limb perfusion	NR	Mean (SD): € 311(1123)	NR
			13	Euro	Surgery	NR	Metastasectomy	NR	Mean (SD): € 0(0)	NR
			13	Euro	Radiotherapy	NR	Overall	NR	Mean (SD): € 0(0)	NR
			13	Euro	Radiotherapy	NR	Short course (≤6 sessions)	NR	Mean (SD): € 0(0)	NR
			13	Euro	Radiotherapy	NR	Standard course (>6 sessions)	NR	Mean (SD): € 0(0)	NR
			13	Euro	Radiotherapy	NR	Hyperthermia	NR	Mean (SD): € 0(0)	NR
			13	Euro	Systemic therapy	NR	Overall	NR	Mean (SD): € 0(0)	NR

Study name	Patient population	Stage of disease	N	Currency	Description of cost	Oncology specific cost	Cost item	Unit	Cost	*p*-value
			13	Euro	Systemic therapy	NR	Dacarbazine	Vial 200 mg	Mean (SD): € 0(0)	NR
			13	Euro	Systemic therapy	NR	Ipilimumab	Vial 50 mg; Vial 200 mg	Mean (SD): € 0(0)	NR
			13	Euro	Systemic therapy	NR	Vemurafenib	Tablet 240 mg	Mean (SD): € 0(0)	NR
	Healthcare costs of subsequent treatment episodes-Intralymphatic metastases	Intratympanic metastases	40	Euro	Total cost	NR	Total cost	NR	Mean (SD): 4604(11181) Median (IQR): 1696(€189-€86785)	NR
			40	Euro	Medical imaging	NR	Overall	NR	Mean (SD): 297(510)	NR
			40	Euro	Medical imaging	NR	X-ray	NR	Mean (SD): € 15(€ 34)	NR
			40	Euro	Medical imaging	NR	Ultrasound	NR	Mean (SD): € 57(€ 86)	NR
			40	Euro	Medical imaging	NR	CT scan	NR	Mean (SD): € 53(€ 105)	NR
			40	Euro	Medical imaging	NR	MRI scan	NR	Mean (SD): € 29(€ 99)	NR

Study name	Patient population	Stage of disease	N	Currency	Description of cost	Oncology specific cost	Cost item	Unit	Cost	*p*-value
			40	Euro	Medical imaging	NR	PET/CT scan	NR	Mean (SD): € 144(€ 452)	NR
			40	Euro	Pathology	NR	Cytology/histology	NR	Mean (SD): € 114(€ 79)	NR
			40	Euro	Hospital visits	NR	Overall	NR	Mean (SD): € 946(€ 729)	NR
			40	Euro	Hospital visits	NR	Consultation by telephone	NR	Mean (SD): € 18(€ 70)	NR
			40	Euro	Hospital visits	NR	Emergency room visit	NR	Mean (SD): € 4(€ 33)	NR
			40	Euro	Hospital visits	NR	Outpatient visit	NR	Mean (SD): € 907(€ 679)	NR
			40	Euro	Hospital visits	NR	Daycare treatment	NR	Mean (SD): € 17(€ 69)	NR
			40	Euro	Hospital admissions	NR	Overall	NR	Mean (SD): € 625(€ 1164)	NR
			40	Euro	Hospital admissions	NR	Inpatient hospital day	NR	Mean (SD): € 606(€ 1090)	NR
			40	Euro	Hospital admissions	NR	ICU day	NR	Mean (SD): € 18(€ 151)	NR
			40	Euro	Surgery	NR	Overall	NR	Mean (SD): € 367(€ 874)	NR
			40	Euro	Surgery	NR	Biopsy	NR	Mean (SD): € 21(€ 57)	NR

Study name	Patient population	Stage of disease	N	Currency	Description of cost	Oncology specific cost	Cost item	Unit	Cost	*p*-value
			40	Euro	Surgery	NR	Excision	NR	Mean (SD): € 113(€ 113)	NR
			40	Euro	Surgery	NR	Lymph node dissection	NR	Mean (SD): € 26(€ 212)	NR
			40	Euro	Surgery	NR	Isolated limb perfusion	NR	Mean (SD): 181(843)	NR
			40	Euro	Surgery	NR	Metastasectomy	NR	Mean (SD): € 26(€ 52)	NR
			40	Euro	Radiotherapy	NR	Overall	NR	Mean (SD): € 1247(€ 4059)	NR
			40	Euro	Radiotherapy	NR	Short course (≤6 sessions)	NR	Mean (SD): € 170(€ 843)	NR
			40	Euro	Radiotherapy	NR	Standard course (>6 sessions)	NR	Mean (SD): € 229(€ 1314)	NR
			40	Euro	Radiotherapy	NR	Hyperthermia	NR	Mean (SD): € 848(€ 3391)	NR
			40	Euro	Systemic therapy	NR	Overall	NR	Mean (SD): € 1007(€ 8244)	NR
			40	Euro	Systemic therapy	NR	Dacarbazine	Vial 200 mg; Vial 500 mg; Vial 1000 mg	Mean (SD): € 0(€ 0)	NR

Study name	Patient population	Stage of disease	N	Currency	Description of cost	Oncology specific cost	Cost item	Unit	Cost	*p*-value
			40	Euro	Systemic therapy	NR	Ipilimumab	Vial 50 mg; Vial 200 mg	Mean (SD): € 1007(€ 8244)	NR
			40	Euro	Systemic therapy	NR	Vemurafenib	Tablet 240 mg	Mean (SD): € 0(€ 0)	NR
	Healthcare costs of subsequent treatment episodes- Regional lymph node metastases	Regional lymph node metastasis	73	Euro	Total cost	NR	Total cost	NR	Mean (SD): 8129(5926) Median (IQR): 7027(€95-€40520)	NR
			73	Euro	Medical imaging	NR	Overall	NR	Mean (SD): 722(649)	NR
			73	Euro	Medical imaging	NR	X-ray	NR	Mean (SD): € 29(€ 52)	NR
			73	Euro	Medical imaging	NR	Ultrasound	NR	Mean (SD): € 160(€ 178)	NR
			73	Euro	Medical imaging	NR	CT scan	NR	Mean (SD): € 209(€ 333)	NR
			73	Euro	Medical imaging	NR	MRI scan	NR	Mean (SD): € 54(€ 155)	NR
			73	Euro	Medical imaging	NR	PET/CT scan	NR	Mean (SD): € 271(€ 468)	NR
			73	Euro	Pathology	NR	Cytology/histology	NR	Mean (SD): € 174(€ 128)	NR

Study name	Patient population	Stage of disease	N	Currency	Description of cost	Oncology specific cost	Cost item	Unit	Cost	*p*-value
			73	Euro	Hospital visits	NR	Overall	NR	Mean (SD): € 1781(€ 1424)	NR
			73	Euro	Hospital visits	NR	Consultation by telephone	NR	Mean (SD): € 24(€ 44)	NR
			73	Euro	Hospital visits	NR	Emergency room visit	NR	Mean (SD): € 65(€ 155)	NR
			73	Euro	Hospital visits	NR	Outpatient visit	NR	Mean (SD): € 1595(€ 1215)	NR
			73	Euro	Hospital visits	NR	Daycare treatment	NR	Mean (SD): € 97(€ 371)	NR
			73	Euro	Hospital admissions	NR	Overall	NR	Mean (SD): € 2599(€ 3881)	NR
			73	Euro	Hospital admissions	NR	Inpatient hospital day	NR	Mean (SD): € 2584(€ 3872)	NR
			73	Euro	Hospital admissions	NR	ICU day	NR	Mean (SD): € 15(€ 135)	NR
			73	Euro	Surgery	NR	Overall	NR	Mean (SD): € 1620(€ 931)	NR
			73	Euro	Surgery	NR	Biopsy	NR	Mean (SD): € 20(€ 59)	NR
			73	Euro	Surgery	NR	Excision	NR	Mean (SD): € 68(€ 110)	NR
			73	Euro	Surgery	NR	Lymph node dissection	NR	Mean (SD): 1442(849)	NR

Study name	Patient population	Stage of disease	N	Currency	Description of cost	Oncology specific cost	Cost item	Unit	Cost	*p*-value
			73	Euro	Surgery	NR	Isolated limb perfusion	NR	Mean (SD): 0(€ 0)	NR
			73	Euro	Surgery	NR	Metastasectomy	NR	Mean (SD): 91(€ 384)	NR
			73	Euro	Radiotherapy	NR	Overall	NR	Mean (SD): 1232(€ 3091)	NR
			73	Euro	Radiotherapy	NR	Short course (≤6 sessions)	NR	Mean (SD): 138(€ 615)	NR
			73	Euro	Radiotherapy	NR	Standard course (>6 sessions)	NR	Mean (SD): 924(€ 2511)	NR
			73	Euro	Radiotherapy	NR	Hyperthermia	NR	Mean (SD): 171(€ 1559)	NR
			73	Euro	Systemic therapy	NR	Overall	NR	Mean (SD): 0(€ 0)	NR
			73	Euro	Systemic therapy	NR	Dacarbazine	Vial 200/ 500/ 1000 mg	Mean (SD): 0(€ 0)	NR
			73	Euro	Systemic therapy	NR	Ipilimumab	Vial 50/200 mg	Mean (SD): 0(€ 0)	NR
			73	Euro	Systemic therapy	NR	Vemurafenib	Tablet 240 mg	Mean (SD): 0(€ 0)	NR

Study name	Patient population	Stage of disease	N	Currency	Description of cost	Oncology specific cost	Cost item	Unit	Cost	*p*-value
	Healthcare costs of subsequent treatment episodes-Distant metastases	Distant metastases	128	Euro	Total cost	NR	Total cost	NR	Mean (SD): 10393(14345) Median (IQR): 6133(€95-€105483)	NR
			128	Euro	Medical imaging	NR	Overall	NR	Mean (SD): 786(954)	NR
			128	Euro	Medical imaging	NR	X-ray	NR	Mean (SD): € 64(€ 105)	NR
			128	Euro	Medical imaging	NR	Ultrasound	NR	Mean (SD): € 67(€ 109)	NR
			128	Euro	Medical imaging	NR	CT scan	NR	Mean (SD): € 251(€ 307)	NR
			128	Euro	Medical imaging	NR	MRI scan	NR	Mean (SD): € 162(€ 298)	NR
			128	Euro	Medical imaging	NR	PET/CT scan	NR	Mean (SD): € 243(€ 694)	NR
			128	Euro	Pathology	NR	Cytology/histology	NR	Mean (SD): € 76(€ 127)	NR
			128	Euro	Hospital visits	NR	Overall	NR	Mean (SD): € 1223(€ 1278)	NR
			128	Euro	Hospital visits	NR	Consultation by telephone	NR	Mean (SD): € 23(€ 46)	NR
			128	Euro	Hospital visits	NR	Emergency room visit	NR	Mean (SD): € 113(€ 222)	NR
			128	Euro	Hospital visits	NR	Outpatient visit	NR	Mean (SD): € 934(€ 1122)	NR

Study name	Patient population	Stage of disease	N	Currency	Description of cost	Oncology specific cost	Cost item	Unit	Cost	*p*-value
			128	Euro	Hospital visits	NR	Daycare treatment	NR	Mean (SD): € 153(€ 372)	NR
			128	Euro	Hospital admissions	NR	Overall	NR	Mean (SD): € 3818(€ 5258)	NR
			128	Euro	Hospital admissions	NR	Inpatient hospital day	NR	Mean (SD): € 3766(€ 5241)	NR
			128	Euro	Hospital admissions	NR	ICU day	NR	Mean (SD): € 52(€ 419)	NR
			128	Euro	Surgery	NR	Overall	NR	Mean (SD): € 700(€ 1724)	NR
			128	Euro	Surgery	NR	Biopsy	NR	Mean (SD): € 4(€ 31)	NR
			128	Euro	Surgery	NR	Excision	NR	Mean (SD): 25(78)	NR
			128	Euro	Surgery	NR	Lymph node dissection	NR	Mean (SD): € 184(€ 535)	NR
			128	Euro	Surgery	NR	Isolated limb perfusion	NR	Mean (SD): € 0(€ 0)	NR
			128	Euro	Surgery	NR	Metastasectomy	NR	Mean (SD): € 487(€ 1572)	NR
			128	Euro	Radiotherapy	NR	Overall	NR	Mean (SD): € 1929(€ 4267)	NR
			128	Euro	Radiotherapy	NR	Short course (≤6 sessions)	NR	Mean (SD): € 951(€ 1862)	NR

Study name	Patient population	Stage of disease	N	Currency	Description of cost	Oncology specific cost	Cost item	Unit	Cost	*p*-value
			128	Euro	Radiotherapy	NR	Standard course (>6 sessions)	NR	Mean (SD): € 527(€ 2507)	NR
			128	Euro	Radiotherapy	NR	Hyperthermia	NR	Mean (SD): € 451(€ 2497)	NR
			128	Euro	Systemic therapy	NR	Overall	Vial 200/ 500/ 1000 mg	Mean (SD): € 1861(10953)	NR
			128	Euro	Systemic therapy	NR	Dacarbazine	Vial 50/200 mg	Mean (SD): € 58(€ 186)	NR
			128	Euro	Systemic therapy	NR	Ipilimumab	Tablet 240 mg	Mean (SD): € 469(€ 6442)	NR
			128	Euro	Systemic therapy	NR	Vemurafenib	NR	Mean (SD): € 1335(€ 8940)	NR
Serra 2017 (79)	Nodal recurrence	NR	NR	Euro		NR	Ultrasound-guided fine-needle aspiration	NR	Cost per Unit: €495.47;	NR
		NR	NR	Euro		NR	Histology	NR	Cost per Unit: €179.12;	NR
		NR	NR	Euro		NR	Anesthesia consultation	NR	Cost per Unit: €188.70;	NR

Study name	Patient population	Stage of disease	N	Currency	Description of cost	Oncology specific cost	Cost item	Unit	Cost	*p*-value
		NR	NR	Euro	Phase 3: Nodal Recurrence; Costs of Diagnostic and Therapeutic Processes	NR	Preanesthetic assessment (x-ray, ECG, blood tests)	NR	Cost per Unit: €326.66;	NR
		NR	NR	Euro		NR	Intervention (lymph node dissection)	NR	Cost per Unit: €4902.57;	NR
		NR	NR	Euro		NR	Histology	NR	Cost per Unit: €298.54;	NR
		NR	NR	Euro		NR	Assessment of tumor extension	NR	Cost per Unit: €661.11;	NR
		NR	NR	Euro		NR	2nd visit (and subsequent)	NR	Cost per Unit: €156.50;	NR
		NR	NR	Euro		NR	Oncology consultation	NR	Cost per Unit: €255.04;	NR
		NR	NR	Euro		NR	Follow-up oncology consultation	NR	Cost per Unit: €156.50;	NR
		NR	NR	Euro		NR	Total cost, without adjuvant treatment	NR	Total Cost: €7620.21	NR
		NR	NR	Euro		NR	INF treatment4 (5 days × 4 weeks)	NR	Cost per Unit: €4809.67;	NR

Study name	Patient population	Stage of disease	N	Currency	Description of cost	Oncology specific cost	Cost item	Unit	Cost	*p*-value
		NR	NR	Euro		NR	Oncology day hospital (5 d × 4 weeks)	NR	Cost per Unit: €8432.33;	NR
		NR	NR	Euro		NR	Total cost, with INF adjuvant treatment	NR	Cost per Unit: €20 862.21;	NR
		NR	NR	Euro		NR	Radiotherapy	NR	Cost per Unit: €3872.52;	NR
		NR	NR	Euro		NR	Total cost with radiotherapy	NR	Total Cost: 11492.73	NR
	Distant metastasis detected on recurrence	NR	NR	Euro	Biopsy results	NR	SLNB-positive (total cost)	NR	Total Cost: 108,587	NR
		NR	NR	Euro	Biopsy results	NR	SLNB-negative (total cost)	NR	Total Cost: 91741	NR
Jang 2020 (13)	Stage IIB/C	Stage IIB/C	NR	USD	Total Healthcare cost at 1 year	NR	Stage IIB/C	1-Year	Mean (SD): $31,870($49,147)	NR
	Stage IIIA	Stage IIIA	NR	USD	Total Healthcare cost at 1 year	NR	Stage IIIA	1-Year	Mean (SD): $29,224($48,837)	NR

Study name	Patient population	Stage of disease	N	Currency	Description of cost	Oncology specific cost	Cost item	Unit	Cost	*p*-value
Tarhini 2018 (43)	Non-Metastatic melanoma patients	NR	6400	USD	All cause healthcare cost	NR	Total healthcare cost	PPPM	Mean (SD): 1225(2636)	NR
		NR	6400	USD		NR	Total medical	PPPM	Mean (SD): 1103(2547)	NR
		NR	6400	USD		NR	Inpatient admission	PPPM	Mean (SD): 193(1731)	NR
		NR	6400	USD		NR	Emergency department	PPPM	Mean (SD): 39(272)	NR
		NR	6400	USD		NR	Outpatient visits, including drug administration	PPPM	Mean (SD): 864(1668)	NR
		NR	6400	USD		NR	Other	PPPM	Mean (SD): 7(78)	NR
		NR	6400	USD		NR	Pharmacy prescriptions	PPPM	Mean (SD): 122(540)	NR
	Locoregional recurrence cohort,	NR	950	USD		NR	Total healthcare cost	PPPM	Mean (SD): 1989(4202)	NR
		NR	950	USD		NR	Total medical	PPPM	Mean (SD): 1768(4035)	NR
		NR	950	USD		NR	Inpatient admission	PPPM	Mean (SD): 244(1354)	NR
		NR	950	USD		NR	Emergency department	PPPM	Mean (SD): 37(304)	NR

Study name	Patient population	Stage of disease	N	Currency	Description of cost	Oncology specific cost	Cost item	Unit	Cost	*p*-value
		NR	950	USD		NR	Outpatient visits, including drug administration	PPPM	Mean (SD): 1479(3699)	NR
		NR	950	USD		NR	Other	PPPM	Mean (SD): 8(65)	NR
		NR	950	USD		NR	Pharmacy prescriptions	PPPM	Mean (SD): 221(676)	NR
	Matched recurrence free cohorts,	NR	950	USD		NR	Total healthcare cost	PPPM	Mean (SD): 1720(2779)	NR
		NR	950	USD		NR	Total medical	PPPM	Mean (SD): 1513(2612)	NR
		NR	950	USD		NR	Inpatient admission	PPPM	Mean (SD): 225(1385)	NR
		NR	950	USD		NR	Emergency department	PPPM	Mean (SD): 44(231)	NR
		NR	950	USD		NR	Outpatient visits, including drug administration	PPPM	Mean (SD): 1233(2073)	NR
		NR	950	USD		NR	Other	PPPM	Mean (SD): 11(67)	NR
		NR	950	USD		NR	Pharmacy prescriptions	PPPM	Mean (SD): 207(734)	NR

Study name	Patient population	Stage of disease	N	Currency	Description of cost	Oncology specific cost	Cost item	Unit	Cost	*p*-value
	Distant recurrence cohort,	NR	87	USD		NR	Total healthcare cost	PPPM	Mean (SD): 2210(2740)	NR
		NR	87	USD		NR	Total medical	PPPM	Mean (SD): 1979(2624)	NR
		NR	87	USD		NR	Inpatient admission	PPPM	Mean (SD): 369(1316)	NR
		NR	87	USD		NR	Emergency department	PPPM	Mean (SD): 71(265)	NR
		NR	87	USD		NR	Outpatient visits, including drug administration	PPPM	Mean (SD): 1506(1941)	NR
		NR	87	USD		NR	Other	PPPM	Mean (SD): 34(211)	NR
		NR	87	USD		NR	Pharmacy prescriptions	PPPM	Mean (SD): 230(848)	NR
	Matched recurrence free cohorts,	NR	87	USD		NR	Total healthcare cost	PPPM	Mean (SD): 2357(6439)	NR
		NR	87	USD		NR	Total medical	PPPM	Mean (SD): 2087(6250)	NR
		NR	87	USD		NR	Inpatient admission	PPPM	Mean (SD): 239(894)	NR
		NR	87	USD		NR	Emergency department	PPPM	Mean (SD): 55(248)	NR

Study name	Patient population	Stage of disease	N	Currency	Description of cost	Oncology specific cost	Cost item	Unit	Cost	*p*-value
		NR	87	USD		NR	Outpatient visits, including drug administration	PPPM	Mean (SD): 1781(6105)	NR
		NR	87	USD		NR	Other	PPPM	Mean (SD): 12(96)	NR
		NR	87	USD		NR	Pharmacy prescriptions	PPPM	Mean (SD): 270(613)	NR
	During episodes of locoregional recurrence (N=1116 patients) (N=1524 episodes)	NR	1116	USD	All-cause healthcare costs, $US 2017 (PPPM)	NR	Total	PPPM	Mean (SD): 2645(6638)	NR
		NR	1116	USD		NR	Medicaid	PPPM	Mean (SD): 2340(6538)	NR
		NR	1116	USD		NR	IP	PPPM	Mean (SD): 341(2021)	NR
		NR	1116	USD		NR	ED	PPPM	Mean (SD): 70(447)	NR
		NR	1116	USD		NR	OP, including drug administration	PPPM	Mean (SD): 1942(5914)	NR
		NR	1116	USD		NR	Other	PPPM	Mean (SD): 11(101)	NR
		NR	1116	USD		NR	Pharmacy prescriptions	PPPM	Mean (SD): 306(1047)	NR
		NR	102	USD		NR	Total	PPPM	Mean (SD): $12 940(16 341)	NR

Study name	Patient population	Stage of disease	N	Currency	Description of cost	Oncology specific cost	Cost item	Unit	Cost	*p*-value
	During episodes of distant recurrence	NR	102	USD		NR	Medicaid	PPPM	Mean (SD): $11 549(16 118)	NR
		NR	102	USD		NR	IP	PPPM	Mean (SD): 3446(8902)	NR
		NR	102	USD		NR	ED	PPPM	Mean (SD): 492(2553)	NR
		NR	102	USD		NR	OP, including drug administration	PPPM	Mean (SD): 7603(12 621)	NR
		NR	102	USD		NR	Other	PPPM	Mean (SD): 7(28)	NR
		NR	102	USD		NR	Pharmacy prescriptions	PPPM	Mean (SD): 1392(3519)	NR
	During the recurrence-free period	NR	6400	USD		NR	Total	PPPM	Mean (SD): 1076(3661)	NR
		NR	6400	USD		NR	Medicaid	PPPM	Mean (SD): 893(3502)	NR
		NR	6400	USD		NR	IP	PPPM	Mean (SD): 177(1126)	NR
		NR	6400	USD		NR	ED	PPPM	Mean (SD): 40(198)	NR
		NR	6400	USD		NR	OP, including drug administration	PPPM	Mean (SD): 665(3163)	NR

Study name	Patient population	Stage of disease	N	Currency	Description of cost	Oncology specific cost	Cost item	Unit	Cost	*p*-value
		NR	6400	USD		NR	Other	PPPM	Mean (SD): 11(81)	NR
		NR	6400	USD		NR	Pharmacy prescriptions	PPPM	Mean (SD): 183(659)	NR
	Locoregional recurrence cohort	NR	950	USD	Melanoma specific cost during episodes of recurrence	NR	Total surgery and Radiotherapy	PPPM	Total cost: 730	NR
	Locoregional recurrence cohort	NR	950	USD		NR	Total monitoring	PPPM	Total cost: 232	NR
	Locoregional recurrence cohort	NR	950	USD		NR	Total pharma	PPPM	Total cost: 575	NR
	Distant recurrence	NR	87	USD		NR	Total surgery and Radiotherapy	PPPM	Total cost: 1564	NR
	Distant recurrence	NR	87	USD		NR	Total monitoring	PPPM	Total cost: 1086	NR
	Distant recurrence	NR	87	USD		NR	Total pharma	PPPM	Total cost: 5195	NR
	Locoregional recurrence cohort	NR	950	USD	Healthcare cost	NR	Total healthcare	PPPM	Mean (SD): 1647(NR)	<0.001 vs. MRF cohort
		NR	950	USD	Healthcare cost	NR	Total medical	PPPM	Mean (SD): 1345(NR)	<0.001 vs. MRF cohort

Study name	Patient population	Stage of disease	N	Currency	Description of cost	Oncology specific cost	Cost item	Unit	Cost	*p*-value
		NR	950	USD	Healthcare cost	NR	IPA	PPPM	Mean (SD): 210(NR)	0.144 vs. MRF cohort
		NR	950	USD	Healthcare cost	NR	EDA	PPPM	Mean (SD): 44(NR)	0.072 vs. MRF cohort
		NR	950	USD	Health care cost	NR	OP visits	PPPM	Mean (SD): 1083(NR)	<0.001 vs. MRF cohort
		NR	950	USD	Healthcare cost	NR	Other	PPPM	Mean (SD): 8(NR)	0.685 vs. MRF cohort
		NR	950	USD	Healthcare cost	NR	Pharmacy	PPPM	Mean (SD): 302(NR)	<0.001 vs. MRF cohort
	Matched recurrence free cohorts,	NR	950	USD	Healthcare cost	NR	Total healthcare	PPPM	Mean (SD): 805(NR)	NR
		NR	950	USD	Healthcare cost	NR	Total medical	PPPM	Mean (SD): 620(NR)	NR
		NR	950	USD	Healthcare cost	NR	IPA	PPPM	Mean (SD): 141(NR)	NR
		NR	950	USD	Healthcare cost	NR	EDA	PPPM	Mean (SD): 30(NR)	NR

Study name	Patient population	Stage of disease	N	Currency	Description of cost	Oncology specific cost	Cost item	Unit	Cost	*p*-value
		NR	950	USD	Healthcare cost	NR	OP visits	PPPM	Mean (SD): 441(NR)	NR
		NR	950	USD	Healthcare cost	NR	Other	PPPM	Mean (SD): 7(NR)	NR
		NR	950	USD	Healthcare cost	NR	Pharmacy	PPPM	Mean (SD): 186(NR)	NR
	Distant recurrence cohort,	NR	87	USD	Healthcare cost	NR	Total healthcare	PPPM	Mean (SD): $15 937(NR)	<0.001 vs. MRF cohort
		NR	87	USD	Healthcare cost	NR	Total medical	PPPM	Mean (SD): $14 379(NR)	<0.001 vs. MRF cohort
		NR	87	USD	Healthcare cost	NR	IPA	PPPM	Mean (SD): 3662(NR)	<0.001 vs. MRF cohort
		NR	87	USD	Healthcare cost	NR	EDA	PPPM	Mean (SD): 643(NR)	<0.001 vs. MRF cohort
		NR	87	USD	Healthcare cost	NR	OP visits	PPPM	Mean (SD): $10 063(NR)	<0.001 vs. MRF cohort

**Study name**	**Patient population**	**Stage of disease**	**N**	**Currency**	**Description of cost**	**Oncology specific cost**	**Cost item**	**Unit**	**Cost**	***p*-value**
		NR	87	USD	Healthcare cost	NR	Other	PPPM	Mean (SD): 11(NR)	0.236 vs. MRF cohort
		NR	87	USD	Healthcare cost	NR	Pharmacy	PPPM	Mean (SD): 1558(NR)	<0.001 vs. MRF cohort
	Matched recurrence free cohorts,	NR	87	USD	Healthcare cost	NR	Total healthcare	PPPM	Mean (SD): 984(NR)	NR
		NR	87	USD	Healthcare cost	NR	Total medical	PPPM	Mean (SD): 667(NR)	NR
		NR	87	USD	Healthcare cost	NR	IPA	PPPM	Mean (SD): 194(NR)	NR
		NR	87	USD	Healthcare cost	NR	EDA	PPPM	Mean (SD): 35(NR)	NR
		NR	87	USD	Healthcare cost	NR	OP visits	PPPM	Mean (SD): 435(NR)	NR
		NR	87	USD	Healthcare cost	NR	Other	PPPM	Mean (SD): 4(NR)	NR
	Matched recurrence free cohorts,	NR	87	USD	Healthcare cost	NR	Pharmacy	PPPM	Mean (SD): 317(NR)	NR
	Locoregional recurrence cohort	NR	6	USD	Healthcare cost	NR	Pharma-Ipilimumab	PPPM	Total cost: NR	NR
		NR	1	USD	Healthcare cost	NR	Pharma-Metastatic	PPPM	Total cost: NR	NR

Study name	Patient population	Stage of disease	N	Currency	Description of cost	Oncology specific cost	Cost item	Unit	Cost	*p*-value
		NR	43	USD	Healthcare cost	NR	Pharma-Adjuvant	PPPM	Total cost: NR	NR
		NR	265	USD	Healthcare cost	NR	Pharma-Other	PPPM	Mean (SD): 511(NR)	NR
		NR	446	USD	Healthcare cost	NR	Pharma-Lab	PPPM	Mean (SD): 86(NR)	NR
		NR	197	USD	Healthcare cost	NR	Pharma-BRAF	PPPM	Mean (SD): 131(NR)	NR
		NR	316	USD	Healthcare cost	NR	Pharma-Imaging	PPPM	Mean (SD): 616(NR)	NR
		NR	85	USD	Healthcare cost	NR	Radiotherapy	PPPM	Mean (SD): 659(NR)	NR
		NR	48	USD	Healthcare cost	NR	Surgery-Other	PPPM	Mean (SD): 531(NR)	NR
		NR	102	USD	Healthcare cost	NR	Surgery-Lymph	PPPM	Mean (SD): 1476(NR)	NR
		NR	94	USD	Healthcare cost	NR	Surgery-Skin	PPPM	Mean (SD): 617(NR)	NR
	Distant recurrence	NR	9	USD	Healthcare cost	NR	Pharma-Ipilimumab	PPPM	Mean (SD): (NR)	NR
		NR	41	USD	Healthcare cost	NR	Pharma-Metastatic	PPPM	Mean (SD): 7432(NR)	NR
		NR	8	USD	Healthcare cost	NR	Pharma-Adjuvant	PPPM	Mean (SD): 4552(NR)	NR
		NR	75	USD	Healthcare cost	NR	Pharma-Other	PPPM	Mean (SD): 408(NR)	NR
		NR	88	USD	Healthcare cost	NR	Pharma-Lab	PPPM	Mean (SD): 75(NR)	NR

Study name	Patient population	Stage of disease	N	Currency	Description of cost	Oncology specific cost	Cost item	Unit	Cost	*p*-value
		NR	58	USD	Healthcare cost	NR	Pharma-BRAF	PPPM	Mean (SD): 74(NR)	NR
		NR	95	USD	Healthcare cost	NR	Pharma-Imaging	PPPM	Mean (SD): 1052(NR)	NR
		NR	38	USD	Healthcare cost	NR	Radiotherapy	PPPM	Mean (SD): 1423(NR)	NR
		NR	37	USD	Healthcare cost	NR	Surgery-Other	PPPM	Mean (SD): 2296(NR)	NR
		NR	22	USD	Healthcare cost	NR	Surgery-Lymph	PPPM	Mean (SD): 696(NR)	NR
		NR	44	USD	Healthcare cost	NR	Surgery-Skin	PPPM	Mean (SD): 118(NR)	NR

Abbreviations: CT: computed tomography; ECG: Electrocardiogram; EDA: emergency department administration; ICU: intensive care Unit; IP: in patient; IPA: inpatient Admission; IQR: interquartile range; MRI: magnetic resonance imaging; OP: outpatient; PET: positron emission tomography; PPM: per patient per month; SD: standard deviation; USD: united states dollar.

Table 43: HCRU outcomes in patients with melanoma (n = 2 studies)

Study name	Patient population	Stage at diagnosis	N	HCRU item	Units	Resource use	*p*-value
Leeneman 2021 (78)	Healthcare resource use of initial treatment Episode-Patients without disease recurrence	Localized melanoma	54	X-ray	NR	Mean (SD): 0.3(0.7)	NR
			54	Ultrasound	NR	Mean (SD): 0.5(0.9)	NR
			54	CT scan	NR	Mean (SD): 0.2(1)	NR
			54	MRI scan	NR	Mean (SD): 0(0.3)	NR
			54	PET/CT scan	NR	Mean (SD): 0(NA)	NR
			54	Cytology/histology	NR	Mean (SD): 3.1(1.8)	NR

Study name	Patient population	Stage at diagnosis	N	HCRU item	Units	Resource use	*p*-value
			54	Consultation by telephone	NR	Mean (SD): 1(1.6)	NR
			54	Emergency room visit	NR	Mean (SD): 0(0.2)	NR
			54	Outpatient visit	NR	Mean (SD): 16.1(10.4)	NR
			54	Daycare treatment	NR	Mean (SD): 0.1(0.4)	NR
			54	Inpatient hospital day	NR	Mean (SD): 1.3(2)	NR
			54	ICU day	NR	Mean (SD): 0(NA)	NR
			54	Biopsy	NR	Mean (SD): 0.2(0.6)	NR
			54	Excision	NR	Mean (SD): 2.8(1.5)	NR
			54	Amputation	NR	Mean (SD): 0(NA)	NR
			54	Sentinel lymph node biopsy	NR	Mean (SD): 0.2(0.4)	NR
			54	Lymph node dissection	NR	Mean (SD): 0(NA)	NR
			54	Isolated limb perfusion	NR	Mean (SD): 0(NA)	NR
			54	Short course (≤6 sessions)	NR	Mean (SD): 0(NA)	NR
			54	Standard course (>6 sessions)	NR	Mean (SD): <0.1(0.1)	NR
	Healthcare resource use of initial treatment Episode-Patients with disease recurrence	Localized melanoma	144	X-ray	NR	Mean (SD): 0.4(1)	NR
			144	Ultrasound	NR	Mean (SD): 0.7(1.1)	NR
			144	CT scan	NR	Mean (SD): 0.2(0.5)	NR

Study name	Patient population	Stage at diagnosis	N	HCRU item	Units	Resource use	*p*-value
			144	MRI scan	NR	Mean (SD): <0.1(0.2)	NR
			144	PET/CT scan	NR	Mean (SD): <0.1(0.2)	NR
			144	Cytology/histology	NR	Mean (SD): 3(1.5)	NR
			144	Consultation by telephone	NR	Mean (SD): 0.4(0.9)	NR
			144	Emergency room visit	NR	Mean (SD): 0.1(0.4)	NR
			144	Outpatient visit	NR	Mean (SD): 14(8.9)	NR
			144	Daycare treatment	NR	Mean (SD): 0.3(0.7)	NR
			144	Inpatient hospital day	NR	Mean (SD): 1.4(2.5)	NR
			144	ICU day	NR	Mean (SD): 0(NA)	NR
			144	Biopsy	NR	Mean (SD): 0.1(0.5)	NR
			144	Excision	NR	Mean (SD): 2.5(1.3)	NR
			144	Amputation	NR	Mean (SD): <0.1(0.2)	NR
			144	Sentinel lymph node biopsy	NR	Mean (SD): 0.2(0.4)	NR
			144	Lymph node dissection	NR	Mean (SD): 0(NA)	NR
			144	Isolated limb perfusion	NR	Mean (SD): 0(NA)	NR
			144	Short course (≤6 sessions)	NR	Mean (SD): <0.1(0.1)	NR

Study name	Patient population	Stage at diagnosis	N	HCRU item	Units	Resource use	*p*-value
			144	Standard course (>6 sessions)	NR	Mean (SD): <0.1(0.1)	NR
	Healthcare resource use of initial treatment Episode-Regionally advanced melanoma patients without disease recurrence	Regionally advanced melanoma	50	X-ray	NR	Mean (SD): 0.7(2)	NR
			50	Ultrasound	NR	Mean (SD): 0.7 (1.2)	NR
			50	CT scan	NR	Mean (SD): 0.3 (0.5)	NR
			50	MRI scan	NR	Mean (SD): <0.1 (0.2)	NR
			50	PET/CT scan	NR	Mean (SD): 0.1 (0.3)	NR
			50	Cytology/histology	NR	Mean (SD): 3.3 (2.2)	NR
			50	Consultation by telephone	NR	Mean (SD): 0.3 (0.9)	NR
			50	Emergency room visit	NR	Mean (SD): 0.1 (0.6)	NR
			50	Outpatient visit	NR	Mean (SD): 14.5 (14.1)	NR
			50	Daycare treatment	NR	Mean (SD): 0.1 (0.4)	NR
			50	Inpatient hospital day	NR	Mean (SD): 4 (6.4)	NR
			50	ICU day	NR	Mean (SD): 0 (NA)	NR
			50	Biopsy	NR	Mean (SD): 0.4 (0.9)	NR
			50	Excision	NR	Mean (SD): 2.5 (1.5)	NR

Study name	Patient population	Stage at diagnosis	N	HCRU item	Units	Resource use	*p*-value
			50	Amputation	NR	Mean (SD): 0 (NA)	NR
			50	Sentinel lymph node biopsy	NR	Mean (SD): 0.6 (0.5)	NR
			50	Lymph node dissection	NR	Mean (SD): 0.8 (0.4)	NR
			50	Isolated limb perfusion	NR	Mean (SD): 0 (NA)	NR
			50	Short course (≤6 sessions)	NR	Mean (SD): 0 (NA)	NR
			50	Standard course (>6 sessions)	NR	Mean (SD): <0.1 (0.1)	NR
	Healthcare resource use of initial treatment Episode- Patients with disease recurrence	Regionally advanced melanoma	47	X-ray	NR	Mean (SD): 1.2 (2.2)	NR
			47	Ultrasound	NR	Mean (SD): 1 (1.3)	NR
			47	CT scan	NR	Mean (SD): 0.6 (1)	NR
			47	MRI scan	NR	Mean (SD): 0.1 (0.4)	NR
			47	PET/CT scan	NR	Mean (SD): <0.1 (0.2)	NR
			47	Cytology/histology	NR	Mean (SD): 3.3 (1.7)	NR
			47	Consultation by telephone	NR	Mean (SD): 0.7 (1.6)	NR
			47	Emergency room visit	NR	Mean (SD): 0.1 (0.4)	NR
			47	Outpatient visit	NR	Mean (SD): 13.6 (12.1)	NR

Study name	Patient population	Stage at diagnosis	N	HCRU item	Units	Resource use	*p*-value
			47	Daycare treatment	NR	Mean (SD): 0.3 (0.6)	NR
			47	Inpatient hospital day	NR	Mean (SD): 6 (11.2)	NR
			47	ICU day	NR	Mean (SD): <0.1 (0.1)	NR
			47	Biopsy	NR	Mean (SD): 0.3 (0.8)	NR
			47	Excision	NR	Mean (SD): 1.9 (0.7)	NR
			47	Amputation	NR	Mean (SD): <0.1 (0.1)	NR
			47	Sentinel lymph node biopsy	NR	Mean (SD): 0.6 (0.5)	NR
			47	Lymph node dissection	NR	Mean (SD): 0.8 (0.5)	NR
			47	Isolated limb perfusion	NR	Mean (SD): <0.1 (0.2)	NR
			47	Short course (≤6 sessions)	NR	Mean (SD): <0.1 (0.2)	NR
			47	Standard course (>6 sessions)	NR	Mean (SD): 0.1 (0.3)	NR
	Healthcare costs of subsequent treatment episodes- Local recurrence	Local recurrence	13	X-ray	NR	Mean (SD): 0.3 (0.5)	NR
			13	Ultrasound	NR	Mean (SD): 0.8 (1.2)	NR
			13	CT scan	NR	Mean (SD): 0.2 (0.4)	NR

Study name	Patient population	Stage at diagnosis	N	HCRU item	Units	Resource use	*p*-value
			13	MRI scan	NR	Mean (SD): 0.1 (0.3)	NR
			13	PET/CT scan	NR	Mean (SD): 0.1(0.3)	NR
			13	Cytology/histology	NR	Mean (SD): 3(1.2)	NR
			13	Consultation by telephone	NR	Mean (SD): 1.5(2.1)	NR
			13	Emergency room visit	NR	Mean (SD): 0.1(0.3)	NR
			13	Outpatient visit	NR	Mean (SD): 14.8(7.4)	NR
			13	Daycare treatment	NR	Mean (SD): 0.7(1.4)	NR
			13	Inpatient hospital day	NR	Mean (SD): 3.4(5.7)	NR
			13	ICU day	NR	Mean (SD): 0.1(0.3)	NR
			13	Biopsy	NR	Mean (SD): 0.2(0.6)	NR
			13	Excision	NR	Mean (SD): 2.5(1.4)	NR
			13	Lymph node dissection	NR	Mean (SD): 0(NA)	NR
			13	Isolated limb perfusion	NR	Mean (SD): 0.1(0.3)	NR
			13	Metastasectomy	NR	Mean (SD): 0(NA)	NR
			13	Short course (≤6 sessions)	NR	Mean (SD): 0(NA)	NR

Study name	Patient population	Stage at diagnosis	N	HCRU item	Units	Resource use	*p*-value
			13	Standard course (>6 sessions)	NR	Mean (SD): 0(NA)	NR
			13	Hyperthermia	NR	Mean (SD): 0(NA)	NR
			13	Dacarbazine	NR	Mean (SD): 0(NA)	NR
			13	Ipilimumab	NR	Mean (SD): 0(NA)	NR
			13	Vemurafenib	NR	Mean (SD): 0(NA)	NR
	Healthcare costs of subsequent treatment episodes- Intralymphatic metastases	Intralymphatic metastases	40	X-ray	NR	Mean (SD): 0.3(0.7)	NR
			40	Ultrasound	NR	Mean (SD): 0.6(1)	NR
			40	CT scan	NR	Mean (SD): 0.3(0.7)	NR
			40	MRI scan	NR	Mean (SD): 0.1(0.4)	NR
			40	PET/CT scan	NR	Mean (SD): 0.1(0.4)	NR
			40	Cytology/histology	NR	Mean (SD): 1.9(1.3)	NR
			40	Consultation by telephone	NR	Mean (SD): 1(3.9)	NR
			40	Emergency room visit	NR	Mean (SD): <0.1(0.1)	NR
			40	Outpatient visit	NR	Mean (SD): 9.6(7.2)	NR
			40	Daycare treatment	NR	Mean (SD): 0.1(0.2)	NR
			40	Inpatient hospital day	NR	Mean (SD): 1.2(2.2)	NR

Study name	Patient population	Stage at diagnosis	N	HCRU item	Units	Resource use	*p*-value
			40	ICU day	NR	Mean (SD): <0.1(0.1)	NR
			40	Biopsy	NR	Mean (SD): 0.2(0.6)	NR
			40	Excision	NR	Mean (SD): 1.2(1.2)	NR
			40	Lymph node dissection	NR	Mean (SD): 0.1(0.1)	NR
			40	Isolated limb perfusion	NR	Mean (SD): 0.1(0.2)	NR
			40	Metastasectomy	NR	Mean (SD): 0.3(0.5)	NR
			40	Short course (≤6 sessions)	NR	Mean (SD): 0.1(0.3)	NR
			40	Standard course (>6 sessions)	NR	Mean (SD): 0.1(0.2)	NR
			40	Hyperthermia	NR	Mean (SD): 0.1(0.2)	NR
			40	Dacarbazine	NR	Mean (SD): 0(NA)	NR
			40	Ipilimumab	NR	Mean (SD): <0.1(0.1)	NR
			40	Vemurafenib	NR	Mean (SD): 0(NA)	NR
	Healthcare costs of subsequent treatment episodes- Regional lymph node metastases	Regional lymph node metastases	73	X-ray	NR	Mean (SD): 0.6(1.1)	NR
			73	Ultrasound	NR	Mean (SD): 1.8(2)	NR
			73	CT scan	NR	Mean (SD): 1.4(2.2)	NR

Study name	Patient population	Stage at diagnosis	N	HCRU item	Units	Resource use	*p*-value
			73	MRI scan	NR	Mean (SD): 0.2(0.6)	NR
			73	PET/CT scan	NR	Mean (SD): 0.3(0.4)	NR
			73	Cytology/histology	NR	Mean (SD): 2.9(2.1)	NR
			73	Consultation by telephone	NR	Mean (SD): 1.4(2.5)	NR
			73	Emergency room visit	NR	Mean (SD): 0.2(0.6)	NR
			73	Outpatient visit	NR	Mean (SD): 16.8(12.8)	NR
			73	Daycare treatment	NR	Mean (SD): 0.3(1.3)	NR
			73	Inpatient hospital day	NR	Mean (SD): 5.2(7.8)	NR
			73	ICU day	NR	Mean (SD): <0.1(0.1)	NR
			73	Biopsy	NR	Mean (SD): 0.2(0.6)	NR
			73	Excision	NR	Mean (SD): 0.7(1.2)	NR
			73	Lymph node dissection	NR	Mean (SD): 0.8(0.5)	NR
			73	Isolated limb perfusion	NR	Mean (SD): 0(NA)	NR
			73	Metastasectomy	NR	Mean (SD): 0.1(0.3)	NR

Study name	Patient population	Stage at diagnosis	N	HCRU item	Units	Resource use	*p*-value
			73	Short course (≤6 sessions)	NR	Mean (SD): <0.1(0.2)	NR
			73	Standard course (>6 sessions)	NR	Mean (SD): 0.1(0.3)	NR
			73	Hyperthermia	NR	Mean (SD): <0.1(0.1)	NR
			73	Dacarbazine	NR	Mean (SD): 0(NA)	NR
			73	Ipilimumab	NR	Mean (SD): 0(NA)	NR
			73	Vemurafenib	NR	Mean (SD): 0(NA)	NR
	Healthcare costs of subsequent treatment episodes- Regional lymph node metastases	Distant metastases	128	X-ray	NR	Mean (SD): 1.4(2.2)	NR
			128	Ultrasound	NR	Mean (SD): 0.7(1.2)	NR
			128	CT scan	NR	Mean (SD): 1.6(2)	NR
			128	MRI scan	NR	Mean (SD): 0.6(1.1)	NR
			128	PET/CT scan	NR	Mean (SD): 0.2(0.6)	NR
			128	Cytology/histology	NR	Mean (SD): 1.2(2.1)	NR
			128	Consultation by telephone	NR	Mean (SD): 1.3(2.6)	NR
			128	Emergency room visit	NR	Mean (SD): 0.4(0.8)	NR
			128	Outpatient visit	NR	Mean (SD): 9.9(11.9)	NR
			128	Daycare treatment	NR	Mean (SD): 0.5(1.3)	NR

Study name	Patient population	Stage at diagnosis	N	HCRU item	Units	Resource use	*p*-value
			128	Inpatient hospital day	NR	Mean (SD): 7.6(10.6)	NR
			128	ICU day	NR	Mean (SD): <0.1(0.3)	NR
			128	Biopsy	NR	Mean (SD): <0.1(0.3)	NR
			128	Excision	NR	Mean (SD): 0.3(0.8)	NR
			128	Lymph node dissection	NR	Mean (SD): 0.1(0.3)	NR
			128	Isolated limb perfusion	NR	Mean (SD): 0(NA)	NR
			128	Metastasectomy	NR	Mean (SD): 0.2(0.6)	NR
			128	Short course (≤6 sessions)	NR	Mean (SD): 0.3(0.7)	NR
			128	Standard course (>6 sessions)	NR	Mean (SD): 0.1(0.3)	NR
			128	Hyperthermia	NR	Mean (SD): <0.1(0.2)	NR
			128	Dacarbazine	NR	Mean (SD): 0.1(0.3)	NR
			128	Ipilimumab	NR	Mean (SD): <0.1(0.1)	NR
			128	Vemurafenib	NR	Mean (SD): <0.1(0.2)	NR
Tarhini 2018 (43)		NR	6400	Number of inpatient admissions		Mean (SD): 0.7(3.8)	NR

Study name	Patient population	Stage at diagnosis	N	HCRU item	Units	Resource use	*p*-value
	Overall- Non metastatic melanoma population	NR	6400	Number of inpatient days	Per 100 person-months	Mean (SD): 3.2(22.5)	NR
		NR	6400	Number of emergency department admissions		Mean (SD): 3(10.9)	NR
		NR	6400	Number of outpatient visits		Mean (SD): 146.9(111.1)	NR
	Locoregional recurrence cohort	NR	950	Number of inpatient admissions		Mean (SD): 1.2(4.7)	NR
		NR	950	Number of inpatient days		Mean (SD): 4.4(21.9)	NR
		NR	950	Number of emergency department admissions		Mean (SD): 2.8(9.3)	NR
		NR	950	Number of outpatient visits		Mean (SD): 158.2(156.4)	NR
	Matched recurrence free cohorts	NR	950	Number of inpatient admissions		Mean (SD): 1(4.3)	NR
		NR	950	Number of inpatient days		Mean (SD): 3.5(17.4)	NR
		NR	950	Number of emergency department admissions		Mean (SD): 3.1(9.4)	NR
		NR	950	Number of outpatient visits		Mean (SD): 160.5(155.9)	NR
	Distant recurrence cohort	NR	87	Number of inpatient admissions		Mean (SD): 1.7(5.7)	NR
		NR	87	Number of inpatient days		Mean (SD): 6.7(24.8)	NR
		NR	87	Number of emergency department admissions		Mean (SD): 3.4(10.2)	NR

Study name	Patient population	Stage at diagnosis	N	HCRU item	Units	Resource use	*p*-value
		NR	87	Number of outpatient visits		Mean (SD): 185.4(151.9)	NR
	Matched recurrence free cohorts,	NR	87	Number of inpatient admissions		Mean (SD): 1.5(4.8)	NR
		NR	87	Number of inpatient days		Mean (SD): 7.3(24.6)	NR
		NR	87	Number of emergency department admissions		Mean (SD): 3.1(9)	NR
		NR	87	Number of outpatient visits		Mean (SD): 140(123.2)	NR
	During episodes of locoregional recurrence (N=1524 episodes)	NR	1116	Number of IP admissions		Mean (SD): 2.9(14.7)	NR
		NR	1116	Number of IP days		Mean (SD): 13.5(85.7)	NR
		NR	1116	Number of ED admissions		Mean (SD): 4.4(18.6)	NR
		NR	1116	Number of OP visits		Mean (SD): 216.6(182.3)	NR
	During episodes of distant recurrence (N=102 episodes)	NR	102	Number of IP admissions		Mean (SD): 11.4(19.9)	NR
		NR	102	Number of IP days		Mean (SD): 63.7(125.7)	NR
		NR	102	Number of ED admissions		Mean (SD): 11.5(24.4)	NR
		NR	102	Number of OP visits		Mean (SD): 254.1(186.8)	NR
		NR	6400	Number of IP admissions		Mean (SD): 0.8(4.3)	NR

Study name	Patient population	Stage at diagnosis	N	HCRU item	Units	Resource use	*p*-value
	During the recurrence-free period (N=6400 patients)	NR	6400	Number of IP days		Mean (SD): 4.2(28.3)	NR
		NR	6400	Number of ED admissions		Mean (SD): 3.2(11)	NR
		NR	6400	Number of OP visits		Mean (SD): 123.6(135.6)	NR
	Locoregional recurrence cohort	NR	950	Number of IPA		Mean (SD): 1(NR)	0.004
		NR	950	Number of IP days		Mean (SD): 5.48(NR)	0.008
		NR	950	Number of EDA		Mean (SD): 3.35(NR)	0.012
		NR	950	Number of OP visits		Mean (SD): 154(NR)	<0.001
	Matched recurrence free cohorts	NR	950	Number of IPA		Mean (SD): 0.57(NR)	NR
		NR	950	Number of IP days		Mean (SD): 2.88(NR)	NR
		NR	950	Number of EDA		Mean (SD): 2.3(NR)	NR
		NR	950	Number of OP visits		Mean (SD): 120.44(NR)	NR
	Distant recurrence cohort,	NR	87	Number of IPA		Mean (SD): 10.25(NR)	<0.001
	Distant recurrence cohort,	NR	87	Number of IP days		Mean (SD): 59.45(NR)	<0.001
	Distant recurrence cohort,	NR	87	Number of EDA		Mean (SD): 11.25(NR)	<0.001

Study name	Patient population	Stage at diagnosis	N	HCRU item	Units	Resource use	*p*-value
	Distant recurrence cohort,	NR	87	Number of OP visits		Mean (SD): 297.07(NR)	<0.001
	Matched recurrence free cohorts	NR	87	Number of IPA		Mean (SD): 1.05(NR)	NR
		NR	87	Number of IP days		Mean (SD): 5.1(NR)	NR
		NR	87	Number of EDA		Mean (SD): 2.46(NR)	NR
		NR	87	Number of OP visits		Mean (SD): 111.22(NR)	NR

Abbreviations: CT: computed tomography; ECG: Electrocardiogram; ED: emergency department; EDA: emergency department admission; ICU: intensive care Unit; IP: in patient; IQR: interquartile range; MRI: magnetic resonance imaging; OPA: outpatient admission; PET: positron emission tomography; SD: standard deviation; USD: united states dollar.

Table 44: Economic outcomes in patients included in TNBC studies (n = 2 studies)

Study name	Patient population	Stage of disease	N	Currency	Description of cost	Oncology specific cost	Cost item	Cost	*p*-value
Haiderali 2021 (80)	Locoregional recurrence	Stage II–IIIB	21	USD	Mean monthly cost per patient	Yes	Monthly cost	Mean (SD): 7820 (14914)	NR
			21			Yes	Hospitalization cost	Mean (SD): 3878 (8524)	NR
			21			Yes	ED visits	Mean (SD): 2305 (5199)	NR
			21			Yes	Office visits	Mean (SD): 41 (82); Median: 0	NR
			21			Yes	Inpatient's procedure	Mean (SD): 150 (364); Median: 0	NR

Study name	Patient population	Stage of disease	N	Currency	Description of cost	Oncology specific cost	Cost item	Cost	*p*-value
Başer 2012 (81)	Recurrence patients with TNBC	Stage I-III	87	USD	Adjusted Annual Post-Index Total and Health Plan-paid Costs for Recurrent Patients with TNBC	Yes	All cause impatient cost	Total cost: 28105	NR
	Recurrence patients without TNBC		202			Yes	All cause impatient cost	Total cost: 13505	NR
	Recurrence patients with TNBC		87			Yes	Emergency department cost	Total cost: 307	NR
	Recurrence patients without TNBC		202			Yes	Emergency department cost	Total cost: 17	NR

*Medical cost comprised of total inpatient, outpatient, emergency department and other costs

Abbreviations: ED: emergency department; NR: not reported; TNBC: triple negative breast cancer; USD: united states dollar, SD: standard deviation.

Table 45: HCRU outcomes in patients included in TNBC studies (n = 2 studies)

Study name	Patient population	Stage at diagnosis	N	HCRU item	Resource use	*p*-value
Haiderali 2021 (80)	Locoregional recurrence	Stage II-IIIB	21	Hospitalization visits	Median: 0.15; 38.1%	NR
			21	ED visits	Median: 0.16; 23.8%	NR
Başer 2012 (81)	Recurrence patients with TNBC	Stage I-III	87	All cause Hospitalizations	Mean: 1.67	0.019
	Recurrence patients without TNBC	Stage I-III	202	All cause Hospitalizations	Mean: 1.04	NR
	Recurrence patients with TNBC	Stage I-III	87	Cancer related hospitalizations	Mean: 0.81	0.007
	Recurrence patients without TNBC	Stage I-III	202	Cancer related hospitalizations	Mean: 0.38	NR

Abbreviations: ED: emergency department; HCRU: healthcare resource utilization; IRR: incident rate ratios; NR: not reported; TNBC: triple negative breast cancer.

Appendix S6. Additional review findings overview

Bladder cancer

Table 46: Other survival outcomes in patients with bladder cancer (n = 3 studies)

Study name	Country	Patient population	Type of subgroup	N	Timepoint	Follow-up	Outcome	Results	*p*-value
Ratanapornsompon 2019 (24)	Thailand	T1 High-Grade Non-Muscle Invasive Bladder Cancer	Progression population	10	5 years	Median (range): 40.1 (2.0 to 252.8)	Survival	43.75%	NR
Thomas 2013 (5)	UK	Recurrent (previous high-risk disease) Bladder cancer	Tumour Grade 1	494	NR	Recurrent high-risk tumours-Mean (IQR): 59.4 (6–188) months Progressive high-risk tumour-Mean (IQR): 59.2 (7-179) months	Disease-specific mortality	0 (0)	NR
			Tumour Grade 2					16 (24)	NR
			Tumour Grade 3					110 (26)	NR
			Stage pTis					32 (25)	NR
			Stage pTa					25 (18)	NR
			Stage T1					68 (31)	NR
			Male					80 (21)	NR
			Female					46 (42)	NR
			Age: <72.73					37 (16)	NR
			Age: >72.73					89 (35)	NR
			TUR specimen weight, g: <3					100 (28)	NR
			TUR specimen weight, g: >3					22 (26)	NR

Study name	Country	Patient population	Type of subgroup	N	Timepoint	Follow-up	Outcome	Results	*p*-value
			Tumour growth: solid					7 (29)	NR
			Tumour growth: papillary					58 (26)	NR
			Both					23 (27)	NR
			Histology: variant					2 (17)	NR
			Histology: UCC					124 (26)	NR
			Vascular invasion: yes					1 (5)	NR
			Vascular invasion: no					77 (25)	NR
			Background urothelium: normal					19 (22)	NR
			Background urothelium: Cis					58 (23)	NR
			Background urothelium: Absent					49 (34)	NR
			Muscle in TUR samples: yes					74 (27)	NR
			Muscle in TUR samples: no					19 (25)	NR
			Low/intermediate risk recurrence: yes					21 (28)	NR

Study name	Country	Patient population	Type of subgroup	N	Timepoint	Follow-up	Outcome	Results	*p*-value
			Low/intermediate risk recurrence: no					104 (26)	NR
			High-risk recurrence: yes					55 (28)	NR
			High-risk recurrence: no					70 (25)	NR
			Progression: yes					66 (59)	NR
			Progression: no					60 (16)	NR
		Primary tumor patients with stage Pta, Tis, T1	Overall	699			Overall mortality	376 (53.8)	NR
		Progressive tumor patients with stage Pta, Tis, T1	Overall	110				68 (61.8)	NR
		Recurrent tumor patients with stage Pta, Tis, T1	Overall	494				284 (57.5)	NR
Chaux 2012 (4)	USA	Bladder cancer	Recurrence without progression	85	NR	24 months	Mortality	0 (0)	<0.001 vs. recurrence without progression

Abbreviations: NR: not reported; TUR: transurethral Resection; UCB: Urothelial carcinoma of the urinary bladder; UK: United Kingdom; USA: United States of America; WHO: The World Health Organization.

Table 47: Treatment after recurrence in patients with bladder cancer (n = 2 studies)

Study name	Country	Patient population	N	Follow-up	Treatment	Results
Comperat 2015 (26)	France	Patients with recurrence	36	Mean: 87 months (median 49.5 months)	Cystectomy	12 (33.3%)
Olsson 2013 (3)	Sweden	Primary T1 UCB-Recurrent patients (non-muscle invasive category)	168	Median (range): 60 (3-192) months	Intravesical treatment	51 (NR)<br>BCG Induction alone (n=39)<br>Induction BCG and maintenance intravesical BCG (n=12)

Abbreviations: BCG: Bacillus Calmette–Guérin; NR: not reported; UCB: Urothelial carcinoma of the bladder.

Table 48: Prognostic/predictive/risk factors for recurrence and survival in patients with bladder cancer (n = 8 studies)

Study name	Country	Patient population	N	Outcome	Variable	Results	*p*-value
Garg 2021 (22)	USA	NMIBC	1062	Any Recurrence, Including Progression,	Ta low grade	Reference	NR
					PUNLMP,	HR (95% CI): 0.72 (0.57-0.93)	NR
					Tis or Ta with CIS,	HR (95% CI): 1.48 (1.17-1.88)	NR
			546	Intermediate or High-Risk Recurrence or Progression	Ta low grade	Reference	NR
					Ta high grade,	HR (95% CI): 2.59 (1.96-3.42)	NR
					Tis or Ta with CIS,	HR (95% CI): 4.2 (3.08-5.6)	NR
					T1 low grade,	HR (95% CI): 1.68 (1.08-2.62)	NR

Study name	Country	Patient population	N	Outcome	Variable	Results	*p*-value
					T1 high grade,	HR (95% CI): 2.92 (2.26-3.79)	NR
			238	High-Risk Recurrence or Progression	Ta low grade	Reference	NR
					Ta high grade,	HR (95% CI): 2.34 (1.42-3.87)	NR
					Tis or Ta with CIS,	HR (95% CI): 4.7 (2.93-7.7)	NR
					T1 low grade,	HR (95% CI): 3.28 (1.79-6.0)	NR
					T1 high grade,	HR (95% CI): 6.8 (4.6-10.1)	NR
			111	Progression	Ta low grade	Reference	NR
					Ta high grade,	HR (95% CI): 2.66 (1.31-5.4)	NR
					Tis or Ta with CIS,	HR (95% CI): 3.48 (1.72-7.1)	NR
					T1 low grade,	HR (95% CI): 2.55 (1.02-6.4)	NR
					T1 high grade,	HR (95% CI): 5.2 (2.91-9.2)	NR
Ratanaporn sompon 2019 (24)	Thailand	T1 High-Grade Non-Muscle Invasive Bladder Cancer	150	Recurrent risk factor	Previous intravesical BCG: Yes	HR (95% CI): 3.277 (1.21 to 8.87)	0.019 vs. No
					Muscle layer included: No	HR (95% CI): 1.894 (1.00 to 3.59)	0.049 vs. Yes
				Progression risk factor	Dead: Yes	HR (95% CI): 9.192 (2.36 to 35.79)	0.001 vs. No
	NR		90		Tumor ≤1 cm	Reference	Reference

Study name	Country	Patient population	N	Outcome	Variable	Results	*p*-value
Lee 2019 (82)		Low-risk NMIBC		Time to recurrence	Tumor >1.0 cm	HR (95% CI): 2.54 (1.35-4.77)	0.004
Simon 2019 (6)	France	Primary low-grade (TaG1) NMIBC	NR	Recurrence free time – Landmark analysis at 3 years	Number of recurrences: 1	HR (95% CI): 2.11 (1.28–3.49)	NR
			NR		Number of recurrences: 2 to 3	HR (95% CI): 4.72 (2.77–8.05)	NR
			31	Recurrence free time – Frailty models	Localization: Multifocal	HR (95% CI): 1.47 (1.19-1.80)	NR
			333		Smoking: Yes	HR (95% CI): 0.84 (0.71-0.98)	NR
			NR		Time of occurrence: < 6 months	HR (95% CI): 1.33 (1.08–1.63)	NR
			333		Smoking: Yes	HR (95% CI): 0.79 (0.64-0.97)	NR
			NR		Previous treatment: Yes	HR (95% CI): 1.28 (1.05-1.55)	NR
Rasmussen 2019 (1)	Denmark	Non-metastatic bladder cancer-Women	417	Cancer recurrence	T1	Reference	Reference
					T2	HR (95% CI): 2.69 (1.24–5.82)	NR
					T3	HR (95% CI): 5.22 (2.32–11.74)	NR
					T4	HR (95% CI): 9.67 (3.83–24.37)	NR
					N0	Ref.	NR
					N2-3	HR (95% CI): 2.90 (1.34–6.27)	NR
					Adjuvant therapy: No	Reference	Reference

Study name	Country	Patient population	N	Outcome	Variable	Results	*p*-value
					Adjuvant therapy: Yes	HR (95% CI): 1.52 (1.00–2.31)	NR
		Non-metastatic bladder cancer- Men	1525		T1	Reference	Reference
					T2	HR (95% CI): 1.91 (1.28–2.86)	NR
					T3	HR (95% CI): 3.04 (1.93–4.79)	NR
					T4	HR (95% CI): 4.32 (2.38–7.83)	NR
					N0	Reference	Reference
					N1	HR (95% CI): 2.96 (1.93–4.55)	NR
					N2-3	HR (95% CI): 4.59 (2.91–7.24)	NR
Chaime 2013 (27)	USA	NMIBC	7410	Recurrence	Stage Ta	Ref	NR
					Stage T1	HR (95% CI): 1.22 (1.15-1.3)	
Olsson 2013 (3)	Sweden	Bladder cancer (T1)	211	Recurrence	Tumour size, <30 mm	Reference	Reference
					Tumour size, >/=30 mm	HR (95% CI): 1.42 (1.03-1.95)	0.032
					Multiplicity, no	Reference	Reference
					Multiplicity, yes	HR (95% CI): 1.55 (1.11-2.15)	0.010
					Lymphovascular invasion, no	Reference	Reference
					Lymphovascular invasion, yes	HR (95% CI): 2.36 (1.31-4.28)	0.005

Study name	Country	Patient population	N	Outcome	Variable	Results	*p*-value
				Progression	Lymphovascular invasion, no	Reference	Reference
					Lymphovascular invasion, yes	HR (95% CI): 2.92 (1.47-5.81)	0.005
Chaux 2012 (4)	USA	Bladder cancer	85	Progression*	Tumor size	OR (95% CI): 1.4 (95% CI- 1.1-1.8)	0.009 (For each 1 cm of tumor size)

* Reappearance of a neoplasm with a higher pathologic stage (<pTa).

Abbreviations: BCS: Bacillus Calmette-Guerin; CI: confidence interval; CIS: carcinoma in situ; HR: hazard ratio; NMIBC: non-muscle invasive bladder cancer; NR: not reported; OR: odds ratio; PUNLMP: Papillary Urothelial Neoplasm of Low Malignant Potential; USA: United States of America.

Table 49: Prognostic/predictive/risk factors for patients with bladder cancer (n = 3 studies)

Study name	Country	Patient population	N	Outcome	Variable	Results	*p*-value
Chaux 2012 (4)	USA	Recurrent bladder cancer with tumor progression	85	Cancer specific mortality	Tumor size	OR (95% CI): 1.3 (1.1–1.6)	0.004 (For each 1 cm of tumor size)
Thomas 2013 (5)	UK	Bladder cancer patients with recurrent high-risk tumors	318	DSM	Male vs. female	HR (95% CI): 1.34 (1.04–1.72)	0.025
					Age: ≤72.73 years vs. >72.73 years	HR (95% CI): 1.07 (1.05–1.08)	<0.001
					Progression: Yes vs. No	HR (95% CI): 4.36 (3.43–5.54)	<0.001
					Tumor stage	HR (95% CI): 1.30 (1.12-1.51)	<0.001

Study name	Country	Patient population	N	Outcome	Variable	Results	*p*-value
					Low/intermediate risk recurrence: yes vs. no	HR (95% CI): 0.68 (0.50-0.93)	<0.016
Yu 2021 (28)	South Korea	Urachal carcinoma of bladder	108	RFS	Stage II vs I	2.423 (1.005–5.839)	0.049
			23		Stage III vs I	6.012 (2.212–16.341)	<0.001
			8		Stage IIIC vs I	1.067 (1.178–6.393)	0.043
			4		Stage IIID vs I	2.727 (1.247–30.146)	0.013

Abbreviations: DSM: disease specific mortality; HR: hazard ratio; CI: confidence interval; NR: not reported; OR: odds ratio; RFS: Relapse/ Recurrent Free Survival; UK: United Kingdom; USA: United States of America.

Gastric cancer

Table 50. Other survival outcomes in patients with gastric cancer (n = 1 study)

Study name	Country	Patient population	N	Time-point	Follow-up	Results	*p*-value
Kraja 2021 (33)	Albania	Gastric cancer	180	NR	Median: 36± 13 months	Mean (SD): 11.5 (11.9)	NR

Abbreviations: SD: standard deviation.

Table 51: Prognostic/predictive/risk factors for local recurrence in gastric cancer (n = 1 study)

Study name	Country	Patient population	N	Follow-up	Parameter	Results	*p*-value
Kraja 2021 (33)	Albania	Gastric cancer	180	Median: 36 ± 13 months	Vascular invasion	NR	0.04
					N stage	NR	0.046
					Progressive disease	NR	0.003

Abbreviations: NR: not reported.

Head and neck cancer

Table 52: DMFS in patients with HNC (n = 1 study)

Study name	Country	Patient population	N	Time-point	Follow-up	Results (range)	*p*-value
Wilson 2021 **(36)**	USA	Oral tongue squamous cell carcinoma, early stage (pT1N0-pT3N0)	96	NR	Median: 48 months	94.6% (90.1% to 99.3%)	NR

Abbreviations: DMFS: Distant metastasis-free survival; HNC: head and neck cancer; NR: not reported; USA: United States of America.

Table 53: DFS reported in patients with laryngeal cancer (n = 1 study)

Study name	Country	Patient population	N	Time-point	Follow-up	Results	*p*-value
Merja 2019 (83)	NR	Laryngeal cancer with stage T3 and T4	71	NR	6-86 months	NR	0.001 vs. stage T1 and T2

Abbreviations: DFS: Disease-free survival; NR: not reported.

Table 54: Treatment after recurrence in patients with HNC (n = 3 studies)

Study name	Country	Patient population	N	Follow-up	Treatment	Results, n (%)	*p*-value
Wilson 2021 (36)	USA	Oral tongue squamous cell carcinoma with recurrence	20	Median (range): 4 (1.5 months-17.5 years)	Salvage therapy (surgery/radiation therapy)	11 (55)	NR
Kim 2012 (39)	USA	Recurrent locally advanced head and neck cancer	324	60 months	Head and neck cancer-related surgery, n (%)	41 (12.7)	NR
					Radiation therapy, n (%)	220 (67.9)	
					Head and neck cancer-related supportive care, n (%)	230 (71)	
					Chemotherapy stratified by drugs, n (%)	88 (27.2)	
					Cetuximab	3 (0.9)	
					Cisplatin	18 (5.6)	
					5-fluorouracil (5-FU)	11 (3.4)	
					Carboplatin	15 (4.6)	
					Paclitaxel	11 (3.4)	

Study name	Country	Patient population	N	Follow-up	Treatment	Results, n (%)	*p*-value
					Docetaxel	6 (1.9)	
					Methotrexate	19 (5.9)	
					Ifosfamide	0 (0)	
					Bleomycin	2 (0.6)	
					Other chemotherapies	37 (11.4)	
					Targeted chemotherapy	15 (4.6)	
					Platinum-based chemotherapies	31 (9.6)	
Merja 2019 (83)	NR	Laryngeal cancer patients with locoregional failure	NR	NR	Salvage laryngectomy	46.2%	NR
					Tracheostomy after loco-regional failure	30.7%	

Abbreviations: HNC: head and neck cancer; NR: not reported; USA: United States of America.

Table 55: Prognostic/predictive/risk factors for recurrence in patients with HNC (n = 3 studies)

Study name	Country	Patient population	N	Time point	Follow-up	Outcome	Type of analysis	Variable	Results, HR (95% CI)	*p*-value
Leoncini 2015 (84)	Italy	Head and neck squamous cell carcinoma (stage I-III)	586	NR	Median (IQR): 59 months (20-92)	Recurrence	Multivariate analysis	Stage I	Reference	NR
								Stage II	1.02 (0.51–2.05)	
								Stage III	1.27 (0.64–2.52)	
								Stage IV	2.25 (1.26–4.03)	
			1,615					Stage I	Reference	NA

Study name	Country	Patient population	N	Time point	Follow-up	Outcome	Type of analysis	Variable	Results, HR (95% CI)	*p*-value
Brandstorp-Boesen 2016 (34)	Norway	Laryngeal squamous cell carcinoma (glottic) (Stage I-IV without metastasis)		120 months	Median (range): 38.4 (0–339.6) years	Risk of recurrence	Univariate analysis	Stage II	3.06 (2.25±4.18)	<0.001
								Stage III	3.54 (2.46±5.10)	<0.001
								Stage IV	1.81 (1.22±2.69)	<0.01
							Multivariate analysis	Age 0–59 years	Reference	NA
								Age ≥70 years	0.62 (0.45±0.85)	<0.01
								T Stage: T1a	Reference	NA
								T Stage: T1b	3.93 (2.14±7.21)	<0.001
								T Stage: T2	4.04 (2.65±6.17)	<0.001
								T Stage: T3	5.79 (3.55±9.44)	<0.001
								T Stage: T4	2.67 (1.39±5.12)	<0.01
								Period of treatment: 1983–1989	Reference	NA
								Period of treatment: 1997–2003	0.68 (0.47±1.00)	0.05

Study name	Country	Patient population	N	Time point	Follow-up	Outcome	Type of analysis	Variable	Results, HR (95% CI)	*p*-value
								Period of treatment: 2004–2010	0.50 (0.33±0.77)	<0.01
		Laryngeal squamous cell carcinoma (supraglottic) (Stage I-IV without metastasis)					Multivariate analysis	Age 0–59 years	Reference	NA
								Age ≥70 years	0.58 (0.37±0.92)	0.01
Park 2017 (35)	Korea	Salivary gland cancer (stage III-IV without distant metastasis)	240	NR	Median (range): 160 (121.5-282.2) months	RFS	Univariate analysis	Female	Reference	NR
								Male	1.522 (1.064–2.179)	0.021
								Smoking, ≤20 pack-years	Reference	NR
								Smoking, >20 pack-years	1.756 (1.182–2.608)	0.005
								CCI: 0-2	Reference	NR
								CCI: ≥3	2.158 (1.214–3.838)	0.009
								Tumour size, cm, ≤4.0	Reference	NR
								Tumour size, cm, >4.0	1.915 (1.249–2.935)	0.003

Study name	Country	Patient population	N	Time point	Follow-up	Outcome	Type of analysis	Variable	Results, HR (95% CI)	*p*-value
								Pathology, Others	Reference	NR
								Pathology, Salivary duct carcinoma	2.798 (1.837–4.262)	<0.001
								EPE, no	Reference	NR
								EPE, yes	2.505 (1.757–3.573)	<0.001
								LVI, no	Reference	NR
								LVI, yes	3.436 (2.306–5.119)	<0.001
								PNI, no	Reference	NR
								PNI, yes	2.605 (1.819–3.730)	<0.001
								Stage T1–2	Reference	NR
								Stage T3–4	2.370 (1.655–3.394)	<0.001
							Multivariate analysis	Primary site, Parotid	Reference	NR
								Non-parotid	1.862 (1.246–2.783)	0.002
								Histologic grade, Low	Reference	NR

Study name	Country	Patient population	N	Time point	Follow-up	Outcome	Type of analysis	Variable	Results, HR (95% CI)	*p*-value
								Histologic grade, high	2.031 (1.146–3.601)	0.015
								N classification: N0	Reference	NR
								N classification: N2	5.430 (2.667–11.053)	<0.001

Abbreviations: CCI: Charlson Comorbidity Index; EPE: extra parenchymal extension; LVI: lymph vascular invasion; HNC; head and neck cancer; NA: not applicable; NR: not reported; PNI: perineural invasion; RFS: recurrence free survival.

Table 56: Prognostic/predictive/risk factors for OS in patients with HNC (n = 1 study)

Study name	Country	Patient population	N	Time point	Follow-up	Variable	Results, HR (95% CI)	*p*-value
Chang 2017 (85)	Taiwan	Head and Neck cancer (Stages I-IV without metastasis)	4839	NR	Median: 37.8 (interquartile range, 30.6) months	CCI score > 6	Univariate: 1.08 (1.07–1.10)	<0.001 vs <=6
						Clinical stage at first diagnosis	1.31 (1.27–1.36)	<0.001 vs Early stage
						Recurrence-free interval > 1 year	0.65 (0.59–0.71)	<0.001 vs RFI<1 years
						Age ≥ 65 years	Multivariate: 1.13 (1.01–1.26)	0.04 vs <65 years
						CCI score > 6	1.09 (1.07–1.10)	<0.001 vs <=6
						Clinical stage at first diagnosis	1.24 (1.20–1.29)	<0.001 vs Early stage

Study name	Country	Patient population	N	Time point	Follow-up	Variable	Results, HR (95% CI)	*p*-value
						Recurrence-free interval > 1 year	0.69 (0.63–0.76)	<0.001 RFI <1 years

Abbreviations: CCI: Charlson Comorbidity Index; CI; confidence interval; HNC: head and neck cancer; NR: not reported.

Melanoma

Table 57. Summary of RFS/DFS outcomes in patients with melanoma (n = 3 studies)

Study name	Country	Patient population	N	Time-point	Follow-up	Type	Results
Chakera 2019 (41)	Australia	Subungual melanoma (stage IA-IIIB/C)	103	NR	Median: 105 months	DFS	Median (95% CI): 70 (44-435) months<br>Stage I, (range: 58.81, NR)<br>Stage II: 70.14 (range: 35.52, 239.2)<br>Stage III: 20.17 (range: 14.92, 64.59)
Chen 2021 (44)	Taiwan	Conjunctival melanoma T1-T3	20	NR	181 months	DFS	Mean: 59.5 ± 56 months
Ogata 2021 (46)	Japan	Sentinel node-positive melanoma (Stage III), Cohort 1: pre-June 2017 group	63	NR	Cohort 1 (median): 1107 days<br>Cohort 2 (median): 587.5 days	RFS	Median: 24.5 months
		Sentinel node-positive melanoma (Stage III), Cohort 2: post-July 2017 group	56	NR		RFS	Median: 35.2 months

Abbreviations: CI; confidence interval; DFS: disease free survival; NR: not reported; RFS: recurrence free survival.

Table 58. Additional survival outcomes in patients with melanoma (n = 6 studies)

Study name	Country	Patient population	Type of subgroup	N	Time-point	Follow-up	Type of survival	Definition	Results	*p*-value
Ertekin 2021 (51)	Spain	Cutaneous melanoma	Stage I	171	NR	Median (IQR): 6.89 (2.91-12.16) years	Post-recurrence MSS	NA	2.42 years (29.04 months)	0.025
			Stage II	260					2.27 years (27.24 months)	
			Stage III	353					1.70 years (20.4 months)	
			Stage IA-IIID with locoregional recurrence	393					4.48 years (53.76 months)	<0.001
			Stage IA-IIID with mixed recurrence	98					0.98 year (11.76 months)	
			Stage IA-IIID with distant recurrence	293					0.91 year (10.92 months)	
Leung 2022 (86)	NR	Primary cutaneous melanomas with recurrence	Early-stage (stage I-II)	331	NR	NR	Deceased at end of follow-up (mortality)	NR	132 (39.9)	NR
Loidi 2021 (8)	Spain	Cutaneous melanoma	Stage I-III locoregional relapse		5-year		MSS	NR	50%	NR

Study name	Country	Patient population	Type of subgroup	N	Time-point	Follow-up	Type of survival	Definition	Results	*p*-value
			Stage I-III relapse	94	NR	68.63 months	Mortality	Died due to melanoma	66 (70.2%)	NR
Sarac 2020 (9)	Germany	Superficially spreading, nodular, lentigo malignant, acral lentiginous, and other types of melanomas	Stage IA-IIC: late recurrence	99	NR	3 months (minimum)	MSS	NR	Median (range): 31 (22-39) months	NR
					1-year		MSS survival rate		71.9%	
					2-year				44.3%	
					3-year				37.5%	
			Stage III late recurrence	NR	3-year		MSS survival rate		71.00%	
					5-year				59.00%	
			Stage IA-IIC: early recurrence	1,438	NR		MSS		Median (range): 32 (28-35) months	
					1-year		MSS survival rate		72.4%	
					2-year				47.2%	
					3-year				39.3%	
			Stage III early recurrence	NR	3-year		MSS survival rate		59.00%	
					5-year				50.00%	
			Stage IA-IIC: late locoregional recurrence	54	NR		MSS		Mean (SD): 121.7 (12.9) months	
			Stage IA-IIC: early locoregional recurrence	1026	NR		MSS		Mean (SD): 139.7 (6.8) months	

Study name	Country	Patient population	Type of subgroup	N	Time-point	Follow-up	Type of survival	Definition	Results	*p*-value
			Stage IA-IIC: late recurrence	99	NR		Mortality		55 (55.5)	
			Stage IA-IIC: early recurrence	1,438	NR				819 (56.9)	
Tas 2017 (50)	Turkey	Early-stage cutaneous melanoma with recurrence (stage I-III)	Overall	332	NR	NR	Post recurrence survival	Measured from date of relapse to the date of death from any cause or to the last follow-up date	Median (range): 10.5 (0-168) months	NR
			Locoregional alone						Median (range): 16.5 (0-168) months	
			Mixed locoregional and distant						Median (range): 6.0 (1-44) months	
			Distant alone						Median (range): 6.0 (1-72) months	
			Lung						Median (range): 6.0 (1-41)	
			Bone						Median (range): 5.5 (1-72) months	
			Liver						Median (range): 6.0 (1-44) months	
			Brain						Median (range): 5.0 (1.0-61.0) months	
			Others						Median (range): 4.0 (1-28) months	

Study name	Country	Patient population	Type of subgroup	N	Time-point	Follow-up	Type of survival	Definition	Results	*p*-value
Osella-Abate 2015 (12)	Italy	Melanoma patients' recurrence free for first 10 years after diagnosis	(Stage I/II) with late recurrence	77	NR	Median (range): 5.4 (0.1-30.1) years	Mortality	Patients died of melanoma	39 (50.6%)	NR

Abbreviations: CI: confidence interval; IQR: interquartile range; MSS: melanoma specific survival; NA: not applicable; NR: not reported; OS: overall survival; SD: standard deviation.

Table 59: Prognostic/predictive/risk factors for recurrence and survival (n = 15 studies)

Study name	Country	Patient population	N	Follow-up	Outcome	Type of analysis	Outcome subtypes/parameter	Results HR (95% CI)	*p*-value
Feigelson 2019 (10)	USA	Invasive melanoma (stage I-III) with recurrence	1852	Median: 4.1 years	Predictors of recurrence	Adjusted HR	Female	Ref	NR
							Male	1.72 (1.21-2.45)	0.003
							Age in decades (Each decade of increasing age)	1.18 (1.04-1.33)	0.009
							Stage I	Ref	NR
							Stage II	5.54 (3.83-8.03)	<0.0001
							Stage III	18.58 (12.51-27.61)	<0.0001

Study name	Country	Patient population	N	Follow-up	Outcome	Type of analysis	Outcome subtypes/parameter	Results HR (95% CI)	*p*-value
						Adjusted for stage, gender, age as a continuous variable, race/ethnicity, socioeconomic status as measured by percent of college educated households in census tract of residence, and comorbidity index			
						Adjusted HR	Female	Ref	NR
							Male	1.70 (1.19-2.43)	0.003
							Age in decades	1.2 (1.06-1.37)	0.005 (Each decade of increasing age)
							Stage I	Ref	

Study name	Country	Patient population	N	Follow-up	Outcome	Type of analysis	Outcome subtypes/parameter	Results HR (95% CI)	*p*-value
						This model adds dichotomous variables for the type of treatments received in addition to the covariates listed in the prior model. Surgery was not included as all but one case received surgery as part of treatment	Stage II	5.27 (3.63-7.67)	<0.0001
							Stage III	16.23 (10.45-25.19)	<0.0001
Ertekin 2021 (51)	Spain	Cutaneous melanoma (initial stage IA-IIID)	784	Median (IQR): 6.89 (2.91-12.16) years	Post-recurrence MSS	Multivariate Cox regression analysis	Age >72 years (vs. <45 years)	1.49 (1.13–1.97)	0.005
							Distant metastasis (vs. Locoregional)	3.78 (2.86-4.99)	<0.001
							Mixed metastasis (vs. Locoregional)	3.29 (2.65-4.09)	<0.001
Sarac 2020 (9)	Germany		1,537	3 months (minimum)			Male vs Female	1.4 (1.2–1.6)	<0.001

Study name	Country	Patient population	N	Follow-up	Outcome	Type of analysis	Outcome subtypes/parameter	Results HR (95% CI)	*p*-value
		Superficially spreading, nodular, lentigo malignant, acrolentiginous type of melanoma, and others (Stage IA-IIC)			Risk factors for survival (multivariate analysis)	Cox proportional hazards model	Tumour localization, Trunk vs others	7 (1.4–1.9)	<0.001
							Tumour thickness, >2 mm vs ≤2 mm	1.4 (1.0–2.1)	0.043
							Ulceration, Yes vs No	1.3 (1.1–1.7)	0.007
Rockberg 2016 (48)	Sweden	Cutaneous malignant melanoma (Stage I-III)	3,554	Median (range): 4.43 (0-9.8) years	Recurrence/ progression	Cox proportional hazards regression model after covariate-adjustment	Time to recurrence for stage I	Reference	NR
							Time to recurrence for stage II	2.97 (2.54-3.48)	<0.0001
							Time to recurrence for stage III	11.69 (9.79 - 13.96)	<0.0001
Osella-Abate 2015 (12)	Italy	Melanoma (stage I/II) with late recurrence	77	Median (range): 5.4 (0.1-30.1) years	Risk of late recurrence	Multivariable Cox proportional hazards regression model	Age under 40 years vs >40 years	1.65 (1.02–2.65)	0.040
							Clark Level IV vs V	2.09 (1.08–4.02)	0.028
Ogata 2021 (46)	Japan		119		RFS		Adjuvant (performed vs none)	0.37 (0.14–0.99)	0.047

Study name	Country	Patient population	N	Follow-up	Outcome	Type of analysis	Outcome subtypes/parameter	Results HR (95% CI)	*p*-value
		Sentinel node-positive melanoma (Stage III)		Pre-June 2017 group (median): 1107 days Post-July 2017 group (median): 587.5 days		Multivariate analysis: Cox proportional hazards model	Ulceration (absent vs present)	3.23 (1.62–6.44)	<0.001
							SN tumor burden (4 mm>vs 4 mm≤)	2.39 (1.33–4.32)	0.004
Von Schuckman 2019 (11)	Australia	Newly diagnosed Melanoma patients	700	NR	Risk factors for 2-year recurrence	NR	Body site- Head/Neck (vs. trunk)	1.67 (1.01-2.76)	NR
							Body site- Upper limbs (vs. trunk)	0.42 (0.18-0.97)	
							Presence of ulceration (vs. No)	1.55 (1.00-2.41)	
							Mitotic rate/mm2 >3 (vs. <1)	2.40 (1.10 – 5.24)	
							Unclassified histologic subtype (vs. superficial spreading melanoma)	1.84 (1.04-3.25)	

Study name	Country	Patient population	N	Follow-up	Outcome	Type of analysis	Outcome subtypes/parameter	Results HR (95% CI)	*p*-value
							Stage T4b (vs. T1B)	3.86 (1.40-10.64)	
							Disease stage- IIA (vs. IB)	2.19 (1.22-3.93)	
							Disease stage- IIC (vs. IB)	2.52 (1.08-5.87)	
Varey 2017 (47)	Australia	Neurotropic and non-neurotropic cutaneous melanoma (stage I-III)	1,389	Median: 3.5 years	Predictors of recurrence	Multivariate regression analysis with sub-hazard ratios	Breslow thickness (mm), 0.01–1.00	Reference	<0.001
							Breslow thickness (mm), 1.01–2.00	1.75 (1.10–2.79)	
							Breslow thickness (mm), 2.01–4.00	2.63 (1.65–4.20)	
							Breslow thickness (mm), >4	3.32 (1.98–5.57)	
							Stage I/II disease	Reference	<0.001
							Stage III disease	2.22 (1.59–3.12)	
							Ulceration, No	Reference	0.001
							Ulceration, Yes	1.57 (1.21–2.03)	

Study name	Country	Patient population	N	Follow-up	Outcome	Type of analysis	Outcome subtypes/parameter	Results HR (95% CI)	*p*-value
							Excision margin (mm), <2	Reference	<0.001
							Excision margin (mm), 2-<8	0.68 (0.46–1.01)	
							Excision margin (mm), ≥8 vs <2	0.46 (0.31–0.68)	
		Neurotropic and non-neurotropic cutaneous melanoma (stage I-III) with local recurrence	NR		Predictors of local recurrence		Age (per 10-year increase)	1.16 (1.00–1.34)	0.05
							Body site: Head and neck	Reference	0.02
							Body site: Trunk	0.39 (0.20–0.76)	
							Body site: Arms	0.91 (0.49–1.70)	
							Body site: Legs	1.12 (0.61–2.05)	
							Excision margin (mm), <2	Reference	<0.001
							Excision margin (mm), 2-<8	0.56 (0.31–1.00)	

Study name	Country	Patient population	N	Follow-up	Outcome	Type of analysis	Outcome subtypes/parameter	Results HR (95% CI)	*p*-value
							Excision margin (mm), ≥8	0.24 (0.13–0.46)	
		Neurotropic and non-neurotropic cutaneous melanoma (stage I-III) with regional recurrence	NR		Predictors of regional recurrence		Stage I/II disease	Reference	0.001
							Stage III disease	2.13 (1.37–3.30)	
							Ulceration, No	Reference	0.04
							Ulceration, Yes	1.49 (1.03–2.15)	
							Body site: Head and neck	Reference	0.03
							Body site: Trunk	1.11 (0.71–1.73)	
							Body site: Arms	1.01 (0.59–1.73)	
							Body site: Legs	1.85 (1.15–2.99)	
							Excision margin (mm), <2	Reference	0.03
							Excision margin (mm), 2-<8	0.56 (0.33–0.95)	

Study name	Country	Patient population	N	Follow-up	Outcome	Type of analysis	Outcome subtypes/parameter	Results HR (95% CI)	*p*-value
							Excision margin (mm), ≥8	0.49 (0.29–0.83)	
		Neurotropic and non-neurotropic cutaneous melanoma (stage I-III) with distant recurrence	NR		Predictors of distant recurrence		Age (per 10-year increase)	0.86 (0.78–0.94)	<0.001
							Sex: Male	Reference	0.04
							Sex: Female	0.70 (0.51–0.98)	
							Breslow thickness (mm), 0.01–1.00	Reference	<0.001
							Breslow thickness (mm), 1.01–2.00	2.28 (1.15–4.51)	
							Breslow thickness (mm), 2.01–4.00	5.01 (2.57–9.76)	
							Breslow thickness (mm), >4	5.82 (2.83–11.98)	
							Stage I/II disease	Reference	<0.001
							Stage III disease	2.42 (1.58–3.71)	
							Ulceration, No	Reference	0.01

Study name	Country	Patient population	N	Follow-up	Outcome	Type of analysis	Outcome subtypes/parameter	Results HR (95% CI)	*p*-value
							Ulceration, Yes	1.52 (1.10–2.10)	
							Excision margin (mm), <2	Reference	0.03
							Excision margin (mm), 2-<8	0.79 (0.47–1.33)	
							Excision margin (mm), ≥8	0.56 (0.34–0.92)	
Tas 2019 (49)	Turkey	Local and regional cutaneous melanoma, Stage I-II	457	Median (range): 733.6 (36.2–271.8) months	Factors associated with RFS	Multivariate Cox proportional-hazards regression model	Sex, female vs male	2.188 (1.348–3.549)	0.002
							Breslow depth, mm (≤ 2 vs > 2)	2.469 (1.392-4.379)	0.002
							Mitotic rate (≤ 2 vs> 2)	2.643 (1.563–4.470)	0.0001
		Local and regional cutaneous melanoma, Stage III	250				Mitotic rate (≤ 2 vs> 2)	1.642 (1.057–2.549)	0.02
	NR		1,244	NR		NR	Older at diagnosis	HR: 1.02	<0.001

Study name	Country	Patient population	N	Follow-up	Outcome	Type of analysis	Outcome subtypes/parameter	Results HR (95% CI)	*p*-value
Leung 2022 (86)		Early-stage primary cutaneous melanomas			Risk of melanoma recurrence		Second quartile of income range of $77,484-$99,677	HR: 1.36	0.046
							Clark's level above 4, melanoma stage above 2A with the presence of tumor-infiltrating lymphocytes	HR: 1.51	0.02
							Presence of mitoses	HR: 2.09	0.001
Rasmussen 2019 (1)	Denmark	Non-metastatic malignant melanoma, Women	7,328	NR	Prognostic factors for recurrence	Univariate Cox proportional hazard analyses	T stage, 1	Reference	p value <0.001
							T stage, 2	4.47 (3.39–5.90)	
							T stage, 3	14.19 (10.83–18.59)	
							T stage, 4	27.02 (20.47–35.65)	
							N stage, N0	Reference	
							N stage, N1	13.10 (10.36–16.57)	
							N stage, N2	18.55 (13.78–24.96)	

**Study name**	**Country**	**Patient population**	**N**	**Follow-up**	**Outcome**	**Type of analysis**	**Outcome subtypes/parameter**	**Results HR (95% CI)**	***p*-value**
							N stage, N3	26.97 (14.29–50.89)	
		Non-metastatic malignant melanoma, Men	6,181				T stage, 1	Reference	p value <0.001
							T stage, 2	3.32 (2.59–4.25)	
							T stage, 3	10.36 (8.25–13.01)	
							T stage, 4	18.61 (14.73–23.52)	
							N stage, N0	Reference	
							N stage, N1	7.21 (5.90–8.80)	
							N stage, N2	10.57 (8.16–13.71)	
							N stage, N3	28.79 (17.37–47.72)	
Loidi 2021 (8)	Spain	Patients with cutaneous melanoma	308	68.83 months	Prognostic factors for relapse	Logistic regression model	Sentinel node status-Negative	Reference	0.009
							Sentinel node status-Positive	3.548 (1.377-9.140)	

Study name	Country	Patient population	N	Follow-up	Outcome	Type of analysis	Outcome subtypes/parameter	Results HR (95% CI)	*p*-value
Kolla 2021 (7)	USA	Patents with primary acral lentiginous melanoma	433	32.5 months	Predictive factor for recurrence free survival	Multivariable Cox proportional hazards model	Nodal disease status- Absent	Reference	NR
							Nodal disease status- Positive	2.12 (1.38- 3.80)	0.001
							Breslow thickness (No reference)	1.13 (1.06- 1.20)	<0.001
							Ulceration Present (vs. absent)	2.29 (1.63– 3.21)	<0.001
							Ulceration unknown (vs. absent)	0.53 (.30– .916)	
							Mitoses present (vs. absent)	1.79 (1.25– 2.56)	0.003
							Mitoses unknown (vs. absent)	1.56 (1.04– 2.33)	
							Stage II (vs. I)	17.31 (.0– 290)	<0.001
							Stage III (vs. I)	32.44 (.0– 544)	
Bleicher 2020 (40)	USA	Patients with stage II melanoma	580	4.9 years	Predictive factor for recurrence	multivariable Cox regression.	Age	1.01 (1.00- 1.02)	0.03

Study name	Country	Patient population	N	Follow-up	Outcome	Type of analysis	Outcome subtypes/parameter	Results HR (95% CI)	*p*-value
							Stage IIB (vs. IIA)	2.02 (1.38-2.97)	<0.01
							Stage IIC (vs. IIA)	2.09 (1.36-3.23)	<0.01
Chakera 2019 (41)	Australia	Patients with subungual Melanoma	103	105 months	Predictive factor for DFS	Multivariable Cox proportional hazards model	Right primary	Reference	NR
							Left primary	0.42 (0.21-0.85)	0.0163
							Lymph node status-Negative	Reference	NR
							Lymph node status-Micro/macro positive	2.55 (1.20-5.43)	0.0147

Abbreviations: CI: confidence interval; DFS: disease free survival; HR: hazard ratio; MSS: melanoma specific survival; NR: not reported; RFS: recurrence free survival USA: United States of America.

Table 60: Treatments/surgery after recurrence in patients with melanoma (n = 2 studies)

Study name	Country	Patient population	N	Follow-up	Outcome subtypes/parameter	Results n (%)
Kolla 2021 (7)	USA	Acral lentiginous melanoma with recurrence (Stage IIB)	13	Median (range): 32 (0–259) months	Lymph node excision	11 (84.6)
					Systemic therapy (interferon, chemotherapy, or immunotherapy)	9 (69.2)
					Radiotherapy	4 (30)
			26		Lymph node excision	11 (84)

Study name	Country	Patient population	N	Follow-up	Outcome subtypes/parameter	Results n (%)
		Acral lentiginous melanoma with recurrence (Stage IIC)			Systemic therapy (interferon, chemotherapy, or immunotherapy)	9 (69.2)
					Radiotherapy	4 (3.80)
Von Schuckman 2019 (11)	Australia	Newly diagnosed Melanoma patients having first recurrence	94	NR	Surgery	64 (68.1)

Abbreviations: NR: not reported; USA: United States of America

Non-small cell lung cancer (NSCLC)

Table 61. Survival outcomes for recurrent patient with NSCLC (n = 2 studies)

Study name	Country	Patient population	Type of subgroup	N	Time-point	Follow-up	Type of survival	Results	*p*-value
Chouaid 2018 (53)	France, Germany, UK	NSCLC	Stage IB-IIIA	332	NR	Maximum follow-up: 51.4 months	DFS*	Median (range) [95% CI]: 48 (0.1-51.4) [42.3-NE] months<br>25th percentile: 13.2 (95% CI: 11.0, 15.3)	NR
			Stage IB	71				Median (range) [95% CI]: NE (0.2-50.4) [NE-NE]<br>25th percentile: 23.7 (95% CI: 18.6, 33.9)	
			Stage IIA	68				Median (range) [95% CI]: 42.3 (0.1-49.6) [42.3-NE]<br>25th percentile: 11.9 (95% CI: 9.7, 16.8)	
			Stage IIB	73				Median (range) [95% CI]: 38.5 (0.4-51.4) [25.2-NE]<br>25th percentile: 8.7 (95% CI: 6.8, 13.2)	
			Stage IIIA	120				Median (range) [95% CI]: 28.5 (0.4-50.4) [23.4 -NE]<br>25th percentile: 10.2 (95% CI: 8.5, 13.5)	
			Stage IB-IIIA	284			DMFS*	Median (range) [95% CI]: NE (0.1, 51.4) [38-NE]<br>25th percentile: 17.7 (95% CI: 15.2, 19.9)	

Study name	Country	Patient population	Type of subgroup	N	Time-point	Follow-up	Type of survival	Results	*p*-value
			Stage IB	59				Median (range) [95% CI]: NE (0.2, 50.4) [NE-NE]<br>25th percentile: 31.6 (95% CI: 20.0, NE)	
			Stage IIA	63				Median (range) [95% CI]: 42.3 (0.1, 49.6) [41.7-NE]<br>25th percentile: 15.8 (95% CI: 11.3, 23.2)	
			Stage IIB	63				Median (range) [95% CI]: 48 (0.4, 51.4) [35.1-NE]<br>25th percentile: 15.2 (95% CI: 7.7, 21.2)	
			Stage IIIA	99				Median (range) [95% CI]: 47.4 (0.4, 50.4) [30.2-NE]<br>25th percentile: 14.8 (95% CI: 10.6, 17.7)	
Buck 2015 (57)	USA	NSCLC	Stage IB to IIIA	609	NR	Maximum follow-up: 63.12 months	DFS	25th quartile: 23.38 (95% CI: 17.56 to 40.32)	0.0125
			Stage IB	215				25th quartile: 42.06 (95% CI: 19.83 to NE)	
			Stage IIA/II	130				25th quartile: 39.13 (95% CI: 13.65 to NE)	
			Stage IIB	110				25th quartile: 22.72 (95% CI: 13.02 to 63.12)	
			Stage IIIA	154				25th quartile: 12.2 (95% CI: 7.14 to 20.45)	

*DFS and DMFS reported for patients with recurrence or death

Abbreviations: DFS: disease-free survival; DMFS: distant metastasis free survival; NSCLC: non-small cell lung cancer; NE: not estimated; UK: United Kingdom; USA: United States of America.

Table 62: Therapy/treatments after recurrence in patients with NSCLC (n = 3 studies)

Study name	Country	Patient population	Stage	N	Follow-up	Treatments	Results	*p*-value
Buck 2015 (57)	USA	NSCLC patients taking treatment	Stage IB to IIIA	68	NR	Bevacizumab, carboplatin, paclitaxel	4 (5.9)	NR
						Carboplatin	6 (8.8)	
						Carboplatin, paclitaxel	17 (25.0)	
						Carboplatin, pemetrexed	4 (5.9)	
						Cisplatin	5 (7.4)	
						Erlotinib	7 (10.3)	
						Pemetrexed	n (%):7 (10.3)	
						Others	18 (26.5)	
			Stage IB	17		Bevacizumab, carboplatin, paclitaxel	2 (11.8)	
						Carboplatin	1 (5.9)	
						Carboplatin, paclitaxel	4 (23.5)	
						Carboplatin, pemetrexed	1 (5.9)	
						Cisplatin	2 (11.8)	
						Erlotinib	3 (17.6)	
						Pemetrexed	1 (5.9)	
						Others	3 (17.6)	
			Stage IIA/II	15		Bevacizumab, carboplatin, paclitaxel	2 (13.3)	
						Carboplatin	0 (0)	
						Carboplatin, paclitaxel	4 (26.7)	
						Carboplatin, pemetrexed	0 (0)	
						Cisplatin	2 (13.3)	

Study name	Country	Patient population	Stage	N	Follow-up	Treatments	Results	*p*-value
						Erlotinib	1 (6.7)	
						Pemetrexed	2 (13.3)	
						Others	4 (26.7)	
			Stage IIB	13		Bevacizumab, carboplatin, paclitaxel	0 (0)	
						Carboplatin	1 (7.7)	
						Carboplatin, paclitaxel	3 (23.1)	
						Carboplatin, pemetrexed	0 (0)	
						Cisplatin	0 (0)	
						Erlotinib	1 (7.7)	
						Pemetrexed	3 (23.1)	
						Others	n (%):5 (38.5)	
			Stage IIIA	23		Bevacizumab, carboplatin, paclitaxel	0 (0)	
						Carboplatin	4 (17.4)	
						Carboplatin, paclitaxel	6 (26.1)	
						Carboplatin, pemetrexed	3 (13)	
						Cisplatin	1 (4.3)	
						Erlotinib	2 (8.7)	
						Pemetrexed	1 (4.3)	
						Others	6 (26.1)	
Kumar 2019 (56)	USA	Recurrent NSCLC patients who received surgery	Stage I	5	Median: 12.8 months	Lobectomy	1 (20)	NR
						Salvage chemoradiation/ immunotherapy	3 (60)	

Abbreviations: NR: not reported; NSCLC: non-small cell lung cancer; USA: United States of America.

Table 63: Prognostic factors for RFS in patients with early-stage NSCLC (n = 1 study)

Study name	Country	Patient population	N	Parameter	Results HR (95% CI)	*p*-value
Li 2019 (55)	NR	Stage I-IIIA (resectable)	882	High neutrophil group vs. low neutrophil group	1.95 (1.47-2.51)	<0.0001

Abbreviations: NR: not reported, NSCLC: non-small cell lung cancer; HR: Hazard ratio, CI: confidence interval.

Renal cell carcinoma (RCC)

Table 64: Treatments after recurrence in patients with RCC (n = 1 study)

Study name	Country	Patient population	N	Follow-up	Treatments	Results	*p*-value
Dabestani 2016 (58)	Sweden	RCC	623	60 months	Systemic oncological treatment	311 (50)	NR
					Metastasectomy	107 (17)	
					Resection of local recurrences	21 (3.4)	
					Resection of no tumor-specific tumor	167 (27)	

Abbreviations: NR: not reported; RCC: renal cell carcinoma.

Triple negative breast cancer (TNBC)

Table 65: Additional survival outcomes in patients with TNBC (n = 5 studies)

Study name	Country	Patient population	N	Time-point	Follow-up	Outcome subtype/parameter	Definition	Results	*p*-value
Tečić 2020 (65)	Croatia	TNBC (overall early stage)	152	5-year	5 years	5-year DFS	Months without recurrence or death	Mean (95% CI): 48 (45-51) months	NR
Dawood 2012 (70)	USA	TNBC with brain metastasis, overall	115	NR	Median (range): 39 (1-233) months	Survival	NR	Median (95%CI): 7.2 (5.7-9.4) months	NR
		TNBC with brain metastasis, Age <50	65					Median (95%CI): 8.5 (6.6-11.3) months	0.11
		TNBC with brain metastasis, age >=50	50					Median (95%CI): 5.5 (3.5-9.4) months	
		TNBC with brain metastasis, stage I	16					Median (95%CI): 7.7 (5.7-18.4) months	0.61
		TNBC with brain metastasis, stage II	51					Median (95%CI): 9.8 (5.5-11.6) months	
		TNBC with brain metastasis, stage III	48					Median (95%CI): 5.8 (3.4-6.9) months	

Study name	Country	Patient population	N	Time-point	Follow-up	Outcome subtype/parameter	Definition	Results	*p*-value
		TNBC with brain metastasis, overall	115	NR		Mortality	NR	94 (81.7)	NR
		TNBC with distant metastases at sites other than the brain	690	NR		Survival	NR	Median: 11.6 months	0.006
		TNBC with brain metastasis	116					Median: 7.2 months	
James 2019 (62)	New Zealand	Non-metastatic TNBC	343	NR	Median: 3.5 years	Metastatic recurrence-free survival	The time from diagnosis to recurrence at a site more distal than locoregional sites	Median: 13 (95% CI: 12-14) years	NR

Study name	Country	Patient population	N	Time-point	Follow-up	Outcome subtype/parameter	Definition	Results	*p*-value
						Locoregional recurrence-free survival	Time from diagnosis to clinical, histopathological or radiological recurrence in ipsilateral breast/chest wall or regional lymph nodes at axillary, supraclavicular, infraclavicular or internal mammary regions	Median: 15.3 (95%CI: 14.1-16.4)	
Gonçalves 2018 (66)	Brazil	TNBC	87	NR	5 years	DFS	NR	Median (95%CI): 43 (38-48) months	NR
		TNBC Overall			5-years	DFS	NR	57.50%; 95% CI (46.4 to 67.1)	
		TNBC			3-years	DFS	NR	64%	
Eralp 2014 (20)	Turkey	TNBC (stage I-III)	316	NR	Median: 52.2 months	Survival after initial recurrence	NR	Median ± SD (range): 20.6 ± 2.7 (15.3-25.9) months	NR

Abbreviations: BCFI: BC-free interval; DFS: disease free survival; NR: not reported; RFS: Relapse-free survival SD: standard deviation; TNBC: triple-negative breast cancer; USA: United States of America.

Table 66: Therapies after recurrence in patients with TNBC (n = 1 study)

Study name	Country	Patient population	N	Follow-up	Outcome	Outcome subtypes/parameter	Results	*p*-value
Van 2016 (18)	Netherlands	TNBC with regional recurrence	75	Median: 5 years	Therapy after recurrence	Chemotherapy	45 (60)	NR

Abbreviations: NR: not reported; TNBC: triple-negative breast cancer.

Table 67: Prognostic/predictive/risk factors for recurrence and survival in TNBC (n = 8 studies)

Study name	Country	Patient population	N	Follow-up	Outcome	Outcome subtypes/parameter	Results	*p*-value
Dawood 2012 (64)	USA	TNBC (stage I-III)	2,448	Median (range): 39 (1-233) months	Time to Brain Metastases - Overall	Stage: Stage III vs I	HR (95% CI): 3.51 (1.85-6.67)	0.0001
			2,311	Median: 39 months (range: 1-233 months)	Distant-DFS	Age: > 50 vs. < 50	HR (95% CI)0.74 (0.64-0.86)	0.0001
						Stage: II vs. I	HR (95% CI): 2.53 (1.98-3.24)	<0.0001
						Stage: III vs. I	HR (95% CI): 5.35 (4.06-7.04)	<0.0001
						LVI: Positive vs. Negative	HR (95% CI): 2.42 (2.08-2.82)	<0.0001
						Adjuvant Radiation: Yes vs. No	HR (95% CI): 0.56 (0.48-0.66)	<0.0001
						Patients with BMI <25 kg/m2	HR (95% CI): 0.79 (0.65-0.96)	0.019

Study name	Country	Patient population	N	Follow-up	Outcome	Outcome subtypes/parameter	Results	*p*-value
					RFS	Age: > 50 vs. < 50	HR (95% CI): 0.74 (0.65-0.86)	<0.0001
						Race: Other vs. White	HR (95% CI): 0.8 (0.66-0.98)	0.031
						Stage: II vs. I	HR (95% CI): 2.49 (2.00-3.11)	<0.0001
						Stage: III vs. I	HR (95% CI): 4.79 (3.72-6.17)	<0.0001
						LVI: Positive vs. Negative	HR (95% CI): 2.52 (2.19-2.91)	<0.0001
						Adjuvant radiation Yes vs. No	HR (95% CI): 0.53 (0.45-0.61)	<0.0001
						Taxane: Yes vs. No	HR (95% CI): 0.71 (0.6-0.85)	0.0002
James 2019 (62)	New Zealand	Non-metastatic TNBC	1,396	Median: 3.5 years	Prognostic factors Locoregional RFS	Radiotherapy (yes vs. no)	HR (95% CI): 0.40 (0.28-0.58)	<0.001
						Chemotherapy (yes vs. no)	HR (95% CI): 0.47 (0.34-0.64)	<0.001
						Lymphovascular invasion (yes vs. no)	HR (95% CI): 1.56 (1.13-2.15)	0.006
						Node positive (yes vs. no)	HR (95% CI): 2.82 (2.02-3.95)	<0.001
						Breast surgery (yes vs. no)	HR (95% CI): 0.55 (0.37-0.81)	0.002
						T1	Reference	Reference
						T2 compared with T1	HR (95% CI): 1.47 (1.06-2.05)	0.022

Study name	Country	Patient population	N	Follow-up	Outcome	Outcome subtypes/parameter	Results	*p*-value
						T3 compared with T1	HR (95% CI): 2.53 (1.41-4.53)	0.002
						T4 compared with T1	HR (95% CI): 3.60 (1.71-7.57)	0.001
Gonçalves 2018 (66)	Brazil	TNBC	87	5 years	5-Year DFS	Lymph node involvement Positive vs. negative	HR (95% CI): 2.28 (1.12–4.57)	0.02
Ignatov 2018 (17)	Germany	TNBC	1374	78.8 (0–209) months	TNBC (primary, non-metastatic) relapse hazards	Locoregional	OR (95% CI): 5.6 (4.2–7.4)	NR
						Nodal	OR (95% CI): 11 (6.2–19.7)	NR
						Distance metastasis: Bone	OR (95% CI): 2.7 (2-3.7)	NR
						Distance metastasis: Liver	OR (95% CI): 3.4 (2.2-5.1)	NR
						Distance metastasis: Lung	OR (95% CI): 6.8 (4.8-9.7)	NR
						Distance metastasis: Brain	OR (95% CI): 18 (9.7-33.4)	NR
						Distance metastasis: Other	OR (95% CI): 2.5 (1.4-4.7)	NR
Villarreal 2021 (87)	Mexico	TNBC	54	NR	Factors for RFS	TNBC vs. non-TNBC	HR: 2.47	0.035
Eralp 2014 (20)	Turkey	TNBC (stage I-III)	316	Median: 52.2 months	DFS	Tumor stage: T1&T2	Reference	Reference
						Tumor stage: T3&T4	3.03 (1.71-5.35)	<0.001
						Nodal positivity: node (-)	Reference	Reference

Study name	Count ry	Patient populati on	N	Follow-up	Outcome	Outcome subtypes/parameter	Results	*p*-value
						Nodal positivity: node (+)	1.77 (1.05-3.0)	0.03
						Type of operation: Mastectomy	Reference	Reference
Min Sun Bae 2015 (21)	NR	TNBC	398	Median (range): 6.1 (0.3–10.4) years	Recurrence	Preoperative MR imaging use	1.00	NR
						Preoperative MR imaging use	2.66 (1.49, 4.75)	<0.001
						Mammographic density	1.00	NR
						Mammographic density	2.77 (1.39, 5.51)	0.004
						Family history of breast cancer	1.00	NR
						Family history of breast cancer	2.32 (1.10, 4.90)	0.028
						Subgroup: Lymphovascular invasion	1.00	NR
						Subgroup: Lymphovascular invasion	1.83 (1.11, 3.03)	0.019
						Subgroup: Lymphovascular invasion	1.83 (1.11, 3.03)	0.019
Steward 2014 (63)	USA	TNBC	414	Mean (SD): 68.2 (36.4) months	Factors for DFS (multivariate)	T1	Ref group	NR
						T2	HR (95%CI): 2.51 (1.52–4.13)	0.0003
						T3	HR (95%CI): 2.41 (1.01–5.76)	0.0411
						T4	HR (95%CI): 1.64 (0.61–4.39)	0.3267
						N0	Ref group	NR

Study name	Country	Patient population	N	Follow-up	Outcome	Outcome subtypes/parameter	Results	*p*-value
						N1	HR (95%CI): 1.68 (1.01–2.81)	0.0466
						N2/N3	HR (95%CI): 2.99 (1.61–5.54)	0.0005
						Adjuvant chemotherapy	Ref group	NR
						Neoadjuvant chemotherapy	HR (95%CI): 2.14 (1.36–3.38)	0.0011

Abbreviations: DFS: disease free survival; HR: hazard ratio; NR: not reported; RFS: Relapse-free survival; SD: standard deviation; TNBC: triple-negative breast cancer; USA: United States of America.

Appendix S7. Summary of included evidence (n=82)

Table 68: Study characteristics of included studies in the clinical review (n = 75 studies)

Study name	Indication	Study objectives	Study design	Publication type	Country	Data source	Study setting	Time frame	Follow up
**Bladder cancer (16 studies)**									
Garg 2021 (22)	Bladder cancer	Predict risk stratified NMIBC recurrence and progression	Retrospective observational study (Registry based)	Journal article	USA	Community-based health systems	Multicentre	1994 to 2015	Median: 29.4 months
Parsons 2020 (23)	NMIBC	Create a snapshot of patients first diagnosed with bladder transitional cell carcinoma	Retrospective study	Journal article	UK	Institutional databases	Single centre	Dec 2012 to Jun 2016	Mean (range): 23.4 (0.6–59.4) months
Rasmussen 2019 (1)	Bladder cancer	Ensure timely detection of new cancer events, knowledge is warranted on the timing of cancer recurrence and second primary cancer, including risk factors for complete response	Population-based prospective cohort study	Journal article	Denmark	Danish Cancer Register	Single centre	2008 to 2016	NR
Ratanapornsompon 2019 (24)	NMIBC	Evaluate the progression of T1 high-grade non-muscle invasive bladder cancer and prognostic factors for muscle invasive disease	Retrospective study	Journal article	Thailand	Institutional databases	Single centre	2007 to 2017	Median (range): 40.1 (2.0 to 252.8) months

Study name	Indication	Study objectives	Study design	Publication type	Country	Data source	Study setting	Time frame	Follow up
Lee 2019 (25)	Bladder cancer	Validate the significance of the entity of "very-low-risk" bladder cancer	Prospective study	Journal article	Singapore	Institutional databases	Single centre	June 1991 to July 2015	Median (IQR): 79 (47-118) months
Simon 2019 (6)	NMIBC	Assess the prognostic value of multiple recurrences on the risk of progression	Retrospective cohort study	Journal article	France	Institutional databases	Single centre	1986 to 2010	Median (IQR): 7.2 (4.2–10.9) years
Comperat 2015 (26)	Urinary bladder cancer	Report histological findings in urothelial bladder cancer population	Retrospective study	Journal article	France	Academic centers	Multicentre	1992 to 2013	Mean: 87 months Median 49.5 months
Canter 2014 (2)	NMIBC	Examine the natural history of HGT1 bladder cancer by analysing the recurrence and progression rates	Retrospective study	Journal article	USA	Institutional databases	Single centre	1980-2012	Mean: 50.8 months Median (range): 32.5 (2.2-261.2) months
Chamie 2013 (27)	NMIBC	Characterize the natural history of the disease, in terms of recurrence, progression, and bladder cancer-related mortality rates	Retrospective registry cohort	Journal article	USA	SEER-Medicare database	Multicentre	Jan 1992 to Dec 2002	5 years
Olsson 2013 (3)	Bladder cancer	Evaluate prognostic factors for urothelial carcinoma of the bladder	Retrospective study	Journal article	Sweden	Bladder cancer registry	Single centre	1992 to 2001	Median (range): 60 (3-192) months

Study name	Indication	Study objectives	Study design	Publication type	Country	Data source	Study setting	Time frame	Follow up
Thomas 2013 (5)	NMIBC	Compare outcomes in patients with primary, progressive, and recurrent high-risk NMIBC	Retrospective study	Journal article	UK	Royal Hallamshire; Hospital database	Single centre	Jan 1994 to Dec 2009	Recurrent high-risk tumours-Mean (IQR): 59.4 (6–188) months Progressive high-risk tumour-Mean (IQR): 59.2 (7-179) months
Chaux 2012 (4)	Bladder cancer (urothelial carcinoma of the urinary tract)	Evaluate the clinicopathologic and outcome patients with high-grade papillary urothelial carcinoma	Retrospective study	Journal article	USA	Surgical pathology database	Single centre	1998 to 2004	24 months
Yu 2021 (28)	Bladder urachal carcinoma	Evaluate the prognosis and survival predictors for bladder urachal carcinoma	Retrospective study	Journal article	South Korea	Institutional databases	Single centre	1994 to 2020	Mean: 65 months

Study name	Indication	Study objectives	Study design	Publication type	Country	Data source	Study setting	Time frame	Follow up
Chu 2021 (88)	NMIBC	Perform a prospective pilot study of the home-based, mail-in urine Cx Bladder Monitor test to inform th e urgency of surveillance cystoscopy in patients with (NMIBC)	Prospectiv e pilot study	Conference abstract	NR	Institution al databases	Single centre	March 2020 to June 2020	NR
Sultana 2021 (29)	NMIBC	Compare the local low-risk NMIBC recurrence and progression rate	Retrospec tive study	Conference abstract	Europe	Institution al databases	NR	Jan 1, 2009, to 31 Dec 2019	NR
Bhat 2020 (89)	NMIBC	Compare several surveillance strate gies of varying intensity with respect to the detection of recurrence and progressio n	Retrospec tive review	Conference abstract	USA	Institution al databases	Single centre	2004 to 2018	NR
**Gastric cancer (1 study)**									
Kraja 2021 (33)	Gastric cancer	Identify the prognostic factors related to survival outcome in gastric cancer	Retrospec tive study	Conference abstract	Albania	Institution al databases	Single centre	2016 to 2019	Median: 36±13 months
**Head and Neck cancer (9 studies)**									

Study name	Indication	Study objectives	Study design	Publication type	Country	Data source	Study setting	Time frame	Follow up
Wilson 2021 (36)	Oral tongue squamous cell carcinoma	Evaluate the clinical outcomes in a cohort of patients with early-stage oral tongue squamous cell carcinoma	Retrospec tive study	Journal article	USA	Institution al databases	Single centre	2000 to 2018	Median (range): 4 (1.5 months-17.5 years)
Kim 2012 (39)	Head and neck squamous cell carcinoma	Treatment patterns and the incremental healthcare resource utilization and economic cost burden of head and neck cancer	Retrospec tive administra tive claims data	Journal article	USA	Thomson Reuters MarketSca n Databases	Multice ntre	2004 to 2008	5 years
Kanatas 2014 (37)	UK	Primary oral squamous cell carcinoma	98	Journal article	UK	Institution al databases	Multice ntre	Jan 2005 to Dec 2008	NR
Leoncini 2015 (84)	Head and neck squamous cell carcinoma	Evaluate whether demographics, lifestyle habits, clinical data and alcohol dehydrogenase polymorphisms rs1229984 and rs1573496 associated with first primary head and neck are associated with overall survival, recurrence, and second primary cancer	73	Journal article	Italy	Institution al databases	Multice ntre	2002-2012	Median (IQR): 59 months (20-92)

Study name	Indication	Study objectives	Study design	Publication type	Country	Data source	Study setting	Time frame	Follow up
Brandstorp-Boesen 2018 (34)	Laryngeal squamous cell carcinoma	Analyse the subsite-specific risk factors for recurrence in patients treated for laryngeal squamous cell carcinoma	Retrospective cohort study	Journal article	Norway	Institutional databases	Single centre	1983 to 2010	Median (range): 3.2 (0–28.3) years
Park 2017 (35)	Salivary gland cancer	Investigate the incidence and risk factors of salivary gland cancer recurrence >5 years after treatment and associated survival	Retrospective cohort study	Journal article	Korea	University hospital	Single centre	1992-2006	Median (range): 160 (121.5-282.2) months
Jung 2014 (38)	Head and neck squamous cell carcinoma	Report the recurrence rate and the recurrence pickup rate of routine follow up visits after head and neck cancer	Retrospective study	Journal article	Korea	Institutional database	Single centre	Jan 2002 to Dec 2008	Mean (SD): 34.7 (22.8) months
Chang 2017 (85)	Head and neck squamous cell carcinoma	Explore the treatment outcomes treatment strategy for improving the likelihood of survival in patients with different recurrent cancer stages and sites	Retrospective study	Journal article	Taiwan	Data from the Taiwan National Health Insurance and cancer registry databases	Multicentre	1 Jan 2002 to 31 Dec 2011	Median (IQR): 3.15 (2.55) years
Merja 2019 (83)	Laryngeal cancer	Analyse the survival and to compare outcomes of organ sparing modality with surgery	Retrospective study	Conference abstract	NR	Institutional databases	Single centre	2011 to 2012	6-86 months

Study name	Indication	Study objectives	Study design	Publication type	Country	Data source	Study setting	Time frame	Follow up
**Melanoma (22 studies)**									
Kolla 2021 (7)	Acral lentiginous melanoma	Analyse the clinicopathologic features, melanoma-specific survival, and recurrence-free survival	Retrospective review of prospectively collected data	Journal article	USA	United States Melanoma Consortium database	Multicentre	Jan 2000 to Dec 2017	Median (range): 32 (0–259) months
Bleicher 2020 (40)	Melanoma	Define recurrence patterns for Stage II melanoma	Retrospective cohort study	Journal article	USA	Institutional databases	Single centre	Jan 2000 to Dec 2017	Median (IQR): 4.9 (2.5-7.9) years
Feigelson 2019 (10)	Melanoma	Examine incidence, recurrence, and mortality among patients with melanoma	Retrospective cohort study	Journal article	USA	Kaiser Permanente Colorado tumor registry	Multicentre	Jan 2000 to Dec 2015	Median: 4.1 years
Jang 2020 (13)	Melanoma (IIB/C or IIIA)	Evaluate real world recurrence rates, risk factors, and economic impact in melanoma	Retrospective study	Conference abstract	USA	SEER-Medicare data	Multicentre	2003 to 2014	NR

Study name	Indication	Study objectives	Study design	Publication type	Country	Data source	Study setting	Time frame	Follow up
Tarhini 2018 (43)	Non metastatic melanoma	To describe real world patterns of treatment and recurrence in patients with melanoma and to quantify healthcare resource utilization and costs associated with episodes of locoregional/ distant recurrences	Retrospective study	Journal article	USA	Truven Health MarketScan Commercial and Medicare Supplemental database	Single centre	January 2008 to 31 July 2017	Median: 23.1 months
Ertekin 2021 (51)	Melanoma	Investigate the impact of the initial stage of primary melanoma on the pattern and timing of disease recurrence and post-recurrence survival	Retrospective cohort study	Journal article	Spain	Institutional databases	Single centre	Jan 1996 to Dec 2018	Median (IQR): 6.89 (2.91-12.16) years
Loidi 2021 (8)	Melanoma	Investigate the patterns of melanoma recurrence in the local population, including factors that may influence in this event and timing of relapse	Retrospective cohort study	Journal article	Spain	Institutional databases	Single centre	2002 to 2012	Mean: 68.63 months
Leeneman 2019 (45)	Cutaneous melanoma	Investigate stage-specific survival from diagnosis, stage-specific disease recurrence, and post-recurrence survival in melanoma patients	Retrospective observational cohort study	Journal article	Netherlands	Netherlands cancer registry	Multicentre	2003 to 2011	Median: 5.4 years

Study name	Indication	Study objectives	Study design	Publication type	Country	Data source	Study setting	Time frame	Follow up
Sarac 2020 (9)	Superficially spreading melanoma, nodular melanoma, lentigo malignant melanoma, acral lentiginous melanoma, and others	Uncover the effect of the recurrence time on the progression of the disease	Retrospective cohort study	Journal article	Germany	German Central Malignant Melanoma Registry (CMMR)	Single centre	Feb 1976 to June 2015	3 months (minimum)
Rockberg 2016 (48)	Cutaneous malignant melanoma	Evaluate survival and recurrence/progression rates of melanoma patients	Retrospective study	Journal article	Sweden	Stockholm melanoma register	Single centre	1 Jan 2005 to 31 Dec 2012	Median (range): 4.43 (0-9.8) years
Rasmussen 2019 (1)	Non-metastatic malignant melanoma	Ensure timely detection of new cancer events	Population-based prospective cohort study	Journal article	Denmark	Danish Cancer Register	Single centre	2008 to 2016	NR
Osella-Abate 2015 (12)	Melanoma	Characterize patients who experienced a late recurrence and compare them to those who remained disease-free to identify possible predictive factors.	Retrospective study	Journal article	Italy	Institutional databases	Single centre	1975 to 2003	Median (range): 5.4 (0.1-30.1) years

Study name	Indication	Study objectives	Study design	Publication type	Country	Data source	Study setting	Time frame	Follow up
Quhill 2021 (42)	Uveal melanoma	Investigate clinical outcomes including recurrence, metastatic rates, and survival outcomes	Retrospective study	Conference abstract	Ireland	Northern Ireland Statistics and Research Agency	Single centre	1998 - May 2020	Median: 79.5 months
Ogata 2021 (46)	Melanoma	Investigate the impact of the changes in the completion lymph node dissection criteria and approval of adjuvant therapies on the real-world outcomes of Japanese stage III melanoma patients	Retrospective study	Journal article	Japan	Institutional databases	Multicentre	Pre-June 2017 group: Jan 2015 to June 2017 Post-July 2017 group: July 2017 to Dec 2019	Pre-June 2017 group (median): 1107 days Post-July 2017 group (median): 587.5 days
Chen 2021 (44)	Conjunctival melanoma	Understand population-specific tumor characteristics and behaviour of conjunctival melanoma	Retrospective cohort study	Journal article	Taiwan	Institutional databases	Single centre	1995 to 2015	Mean (SD): 68.7 (55.8)

Study name	Indication	Study objectives	Study design	Publication type	Country	Data source	Study setting	Time frame	Follow up
Von Schuckman 2019 (11)	Newly diagnosed Melanoma	Investigate the risk of melanoma recurrence in patients with a localized melanoma at a high risk of metastasis	Prospective cohort study	Journal article	Australia	Hospital clinics, private practices, and private pathology laboratories	Multicentre	Oct 2010 to Oct 2014	NR
Chakera 2019 (41)	Subungual melanoma	Identify clinicopathological features predictive of outcome	Retrospective study	Journal article	Australia	Institutional databases	Single centre	1953 to 2014	Median: 105 months
Varey 2017 (47)	Neurotropic melanoma	Assess outcomes and prognosis in neurotropic melanoma	Case control study	Journal article	Australia	Melanoma Institute Australia database	Single centre	1985 to 2013	Median: 3.5 years
Tas 2019 (49)	Cutaneous melanoma	Understand these factors influencing recurrence patterns	Retrospective study	Journal article	Turkey	Institutional databases	Single centre	1993 to 2017	Median (range): 733.6 (36.2–271.8) months
Tas 2017 (50)	Cutaneous melanoma	Determine the recurrence time courses, patterns, outcomes, and factors influencing the recurrence	Retrospective cohort study	Journal article	Turkey	Institutional databases	Single centre	1993 to 2015	NR

Study name	Indication	Study objectives	Study design	Publication type	Country	Data source	Study setting	Time frame	Follow up
Leung 2022 (86)	Early-stage primary cutaneous melanomas	Evaluate clinical (demographics, medical history, surgical margins) and histopathologic (synoptic features) characteristics of melanoma	Retrospective study	Conference abstract	NR	Multi-institutional database	Multicentre	2000 to 2020	NR
Burns 2019 (52)	Melanoma	Understand a subset of Stage lll melanoma patients with low lymph node tumor burden	Retrospective study	Conference abstract	NR	Institutional databases	Single centre	1998 to 2007	Median: 5.3 years
**NSCLC (7 studies)**									
Karacz 2020 (14)	NSCLC	Determine the type and timing of recurrent lung cancer	Retrospective cohort study	Journal article	USA	University of Texas Southwestern Tumor Registry	Single centre	2000 to 2017	NR
Buck 2015 (57)	NSCLC	Describe recent treatment patterns and health resource utilization in NSCLC	Retrospective observational study	Journal article	USA	Vector Oncology Data Warehouse	Multicentre	Jan 2007 to Jan 2014	NR

Study name	Indication	Study objectives	Study design	Publication type	Country	Data source	Study setting	Time frame	Follow up
Martin 2022 (15)	NSCLC	Evaluate the treatment patterns and clinical outcomes for LATAM from the pre-immuno-oncology era	Retrospective study	Journal article	Argentina, Chile, Colombia, Dominican Republic, Mexico, Peru and Uruguay	The LATAM subset of the real-world, global KINDLE study	Multicentre	Jan 2013 to Dec 2017	Median (range): 660 (7–2404) days
Chouaid 2018 (53)	NSCLC	Describe treatment patterns in patients with complete resection of stage IB-IIIA NSCLC	Retrospective observational study	Conference abstract	France, Germany, UK	Medical records	Multicentre	1 Jan 2009 to 31 Dec 2011	Median: 26 months
Slim 2021 [50]	NSCLC	Identify the profile of patients operated for lung cancer and to study the prognostic factors of tumor recurrence	Retrospective observational study	Journal article	Tunisia	Institutional databases	Single centre	Jan 2010 to Dec 2016	Mean (SD): 770 (79) days
Li 2019 (55)	Primary lung adenocarcinoma	Identify the important components which are determinant for early recurrence of ADC	Retrospective cohort study	Conference abstract	USA	Gene Expression Omnibus and Cancer Genome Atlas	NR	NR	NR

Study name	Indication	Study objectives	Study design	Publication type	Country	Data source	Study setting	Time frame	Follow up
Kumar 2019 (56)	NSCLC	Examine short-term outcomes and patterns of failure in a veteran population	Retrospective study	Conference abstract	USA	Institutional databases	Single centre	1 Jan 2017 to 12 Dec 2017	Surgery group: Median-12.8 months Radiation group: Median-11.4 months
**RCC (3 studies)**									
Dabestani 2016 (58)	RCC	Present the occurrence of metastases and local recurrences in RCC	Prospective study	Journal article	Sweden	National Swedish Kidney Cancer Register	Multicentre	2005 to 2009	5 years
Thorstenson 2015 (59)	RCC	Summarizes obtained results during ten years with the National Swedish Kidney Cancer Register	Retrospective registry-based study	Journal article	Sweden	National Swedish Kidney Cancer Register	Multicentre	2005 to 2013	5 years
Alvarado 2019 (60)	Tubulocystic RCC	examine the clinicopathologic and immunohistochemical features of tubulocystic RCC	Retrospective study	Conference abstract	NR	Inpatient database	Single centre	2008 to 2017	12 months to 6 years
**TNBC (18 studies)**									

Study name	Indication	Study objectives	Study design	Publication type	Country	Data source	Study setting	Time frame	Follow up
Kaplan 2017 (61)	TNBC	Evaluate the diagnostic and treatment characteristics and recurrence and disease-specific survival rates	Retrospective cohort study	Journal article	USA	Breast cancer registry database	Single centre	1990 to 2014	Median (range): 6.23 (0.4-23) years Mean: 7.34 years
Matro 2015 (19)	TNBC	Characterize the presentation, treatment, and outcomes for TNBC	Retrospective study	Journal article	USA	NCCN breast cancer outcomes database	Multicentre	1999 to 2009	Median: 29 months
Steward 2014 (63)	TNBC	Describe the outcomes of patients with TNBC in order to determine the patterns of recurrence, time to recurrence, and the impact on overall survival	Retrospective study	Journal article	USA	Institutional databases	Single centre	1 Jan 1999 to 31 Dec 2008	Mean (SD): 68.2 (36.4) months
Dawood 2012 (64)	TNBC	Determine the incidence of brain metastases as a first site of recurrence among women with TNBC	Retrospective study	Journal article	USA	Institutional databases	Single centre	1990 to 2010	Median (range): 39 (1-233) months
Başer 2012 (81)	TNBC	Examine the recurrence rate, healthcare utilization, and cost of early-stage TNBC	Retrospective observational study	Journal article	USA	Oncology management cancer registry	Single centre	1999 to 2009	Mean (SD): 664.06 (430.97)

Study name	Indication	Study objectives	Study design	Publication type	Country	Data source	Study setting	Time frame	Follow up
Haiderali 2021a (16)	TNBC	Examine the real-world HCRU and costs among patients who received neoadjuvant treatment with or without adjuvant treatment in TNBC	Retrospective observational study	Journal article	USA	ConcertAI Oncology Dataset	Single centre	Mar 2008 to Mar 2016	Median: 46.1 months
Haiderali 2021b (80)	TNBC	To examine the real-world HCRU and costs among patients who received neoadjuvant treatment with or without adjuvant treatment in patients diagnosed with early-stage (II–IIIB) triple-negative breast cancer.	Retrospective observational study	Journal article	USA	ConcertAI Oncology Dataset	Single centre	Mar 2008 to Mar 2016	Median: 46.1 months
Tecic 2020 (65)	TNBC	Identify the prognostic factors associated with TNBC	Retrospective cohort, observational, real-world study	Journal article	Croatia	Institutional databases	Single centre	Jan 2009 to Dec 2012	5 years
Ignatov 2018 (17)	TNBC	Evaluate the pattern of recurrence of breast cancer	Retrospective cohort study	Journal article	Germany	Regional cancer registry	Single centre	2000 to 2016	Median (range): 64 (1–209)

Study name	Indication	Study objectives	Study design	Publication type	Country	Data source	Study setting	Time frame	Follow up
Van 2016 (18)	TNBC	Provide a nationwide overview on the occurrence of recurrence and overall survival	Retrospective study	Journal article	Netherlands	Netherlands cancer registry	Single centre	2005 to 2008	Median: 5 years
James 2019 (62)	TNBC	Study the demographics, baseline characteristics, and survival outcomes and prognostic/ predictive factors	Prospective study	Journal article	New Zealand	New Zealand breast cancer registry database	Multicentre	May 1993 to Dec 2014	Median: 3.5 years
Gonçalves 2018 (66)	TNBC	Analyse the clinical, pathological, and sociodemographic aspects and identify potential prognostic factors	Retrospective cohort study	Journal article	Brazil	Institutional databases	Single centre	2003 to 2005	5 years
Villarreal 2021 (87)	TNBC	Describe the patterns of early recurrence in TNBC	Prospective cohort study	Conference abstract	Mexico	Accrued in the Joven & Fuerte prospective cohort	Single centre	NR	NR
Suhani 2017 (67)	TNBC	Characterize the non-metastatic TNBC patients and compare with non-TNBC	Retrospective observational study	Journal article	India	Institutional databases	Single centre	April 2000 to December 2014	Mean: 4.1 years
Gal 2018 (68)	TNBC	Define and delineate the therapeutic approach to elderly patients with early breast cancer	Retrospective observational study	Journal article	Israel	Institutional databases	Single centre	Jan 2004 to Dec 2007	Median (range): 10.6 (8–12) years

Study name	Indication	Study objectives	Study design	Publication type	Country	Data source	Study setting	Time frame	Follow up
Eralp 2014 (20)	TNBC	Evaluate the outcome, identify clinical and pathologic variables that may affect survival.	Retrospective registry study	Journal article	Turkey	Registry of medical records	Multicentre	1993 to 2007	Median: 52.2 months
Metzger-Filho 2013 (69)	TNBC	Evaluate the pattern of recurrence and outcome of TNBC	Retrospective study	Journal article	Multinational	International Breast Cancer Study Group Trials VIII and IX	Multicentre	1988 to 1999	Median: 12.5 years
Min Sun Bae 2015 (21)	TNBC	Determine the imaging and clinical-pathologic factors associated with recurrence	Retrospective study	Journal article	NR	Prospectively maintained database	Single centre	Jan 2003 to Dec 2008	Median (range): 6.1 (0.3–10.4) years

Abbreviations: TNBC: Triple negative breast cancer, NR: not reported, NSCLC: non-small cell lung cancer, ADC: adenocarcinoma, LATAM: Latin America, IQR: inter-quartile range, SD: standard deviation, RCC: Renal cell carcinoma, USA: United States of America.

Table 69: Study characteristics of the included studies in the humanistic review (n = 6 studies)

Study name	Tumor types	Study objectives	Study design	Publication type	Country	Data source	Study setting	Time frame	Follow up
**Bladder cancer**									
Smith 2022 (74)	Bladder Cancer	To characterize the quality of life of patients with bladder cancer at various time points across the continuum of bladder cancer care from non-muscle-invasive disease to metastatic bladder cancer and develop utility scores to inform cost-effective analyses.	Cross-sectional survey	Journal article	USA	Bladder Cancer Advocacy Network Patient Survey Network	Single centre	May 2019 to June 2019	NR
**Gastric cancer**									
Shin 2022 (75)	Gastric cancer	To evaluate the association of FCR of stomach cancer survivors with various factors including socio-demographic characteristics, cancer-related information, physical symptoms, psychological distress, social support, and quality of life in Korean stomach cancer survivors	Cross-sectional study	Journal article	Korea	University affiliated hospitals	Multicentre	Sep 2014 to March 2017	NR
**Melanoma**									

Study name	Tumor types	Study objectives	Study design	Publication type	Country	Data source	Study setting	Time frame	Follow up
Van 2016 (76)	Melanoma	Compare FCR severity between cancer types and identify associations between FCR, demographics, medical characteristics, information provision and health-related quality of life	Cross-sectional study	Journal article	Netherlands	Netherlands Cancer Registry	Single centre	2008 to 2010	NR
Atkinson 2013 (77)	Cutaneous Melanoma	Examine the impact of diagnosis and treatment of clinically localized melanoma on quality of life and distress in women	Cross-sectional study	Journal article	USA	Memorial Sloan-Kettering Cancer Center	Single centre	NR	NR
**Mixed tumor types (Bladder cancer and melanoma)**									
Leclair 2019 (73)	Bladder cancer, melanoma and kidney cancer	Examine FCR and 2 health behaviours, physical activity and fruit and vegetable intake, from early to long-term survivorship in a large cohort of mixed cancer survivors	Prospective longitudinal study*	Journal article	USA	American Cancer SCS-I	Nation wide population based	NR	NR

Abbreviations: SCS-1: study of cancer survivors – 1, FCR: Fludarabine, Cyclophosphamide and rituximab, NR: not reported, USA: United States of America.

Table 70: Study characteristics of the included studies in the economic review (n = 7 studies)

Study name	Tumor types	Study objectives	Study design	Publication type	Country	Data source	Study setting	Time frame	Study perspective	Cost year
**Head and neck cancer**										
Kim 2012 (39)	Head and neck cancer (HNC)	The present study aimed to enhance the literature on the treatment patterns and the incremental healthcare resource utilization and economic cost burden of HNC in the US	Case-control study (retrospective administrative claims databased)	Journal article	USA	Thomson Reuters MarketScan Databases	Multi centre	2004 to 2008	Third-party payer perspective	NR
**Melanoma**										
Leeneman 2021 (78)	Cutaneous melanoma	To provide insight into real-world healthcare costs of patients initially diagnosed with localized or regionally advanced melanoma in three Dutch hospitals between 2003 and 2011.	Retrospective cohort study	Journal article	Netherlands	Netherlands Cancer Registry	Multi centre	Jan 2003 to Dec 2011	Hospital perspective	2018
Serra 2017 (79)	Cutaneous melanoma	To assess costs related to melanoma in these 4 stages (Stage I-IV) of disease, from the time of the first visit to the dermatologist until therapeutic and follow-up processes end.	Cost of illness	Journal article	Spain	Amounts were extracted from official Spanish publications	NR	NR	NR	2015

Study name	Tumor types	Study objectives	Study design	Publication type	Country	Data source	Study setting	Time frame	Study perspective	Cost year
Jang 2020 (13)	Melanoma	To report Real world recurrence rates, risk factors, and economic impact in patients with resected stage IIB/C or IIIA melanoma.	Retrospective study	Conference abstract	USA	SEER-Medicare data	Single centre	2003 to 2014	NR	2018
Tarhini 2018 (43)	Non-Metastatic melanoma	This study aimed to describe real world patterns of treatment and recurrence in patients with melanoma and to quantify healthcare resource utilization and costs associated with episodes of locoregional/ distant recurrences	Retrospective study	Journal article	USA	Truven Health MarketScan Commercial and Medicare Supplemental database	Single centre	January 2008 to 31 July 2017	NR	2017
**TNBC**										
Haiderali 2021b (80)	TNBC	To examine the real-world HCRU and costs among patients who received neoadjuvant treatment with or without adjuvant treatment in patients diagnosed with early-stage (II–IIIB) triple-negative breast cancer.	Retrospective, observational study	Journal article	USA	ConcertAI Oncology Dataset electronic medical record (EMR) data available to ConcertAI	Single centre	Mar 2008 to Mar 2016	Patient perspective	2018

Study name	Tumor types	Study objectives	Study design	Publication type	Country	Data source	Study setting	Time frame	Study perspective	Cost year
Başer 2012 (81)	TNBC	To examine the recurrence rate, health care utilization, and cost of early-stage TNBC in the US managed care setting.	Retrospective observational study	Journal article	USA	Oncology Management cancer registry	Single centre	1999 to 2009	Managed care perspective	NR

Abbreviations: EMR: Electronic medical record; TNBC: Triple negative breast cancer, HNC: head and neck cancer, NR: not reported, SEER: Surveillance, Epidemiology, and End Results; USA: United States of America.

Table 71: Population characteristics of patients in the clinical review (n = 75 studies)

Study name	Patient population	N	Age (Years)	Gender n (%)	Race/ ethnicity	Disease duration	Stage distribution	ECOG PS	Comorbidities	Treatment history, n (%)	Surgery history, n (%)
**Bladder cancer (17 studies)**											
Garg 2021 (22)	Non-muscle invasive recurrent bladder cancer (Ta/T1 low and high grade; Tis or Ta with CIS)	2,956	Mean (SD): 69.1 (11.8)	Male: 2340 (79.2) Female: 616 (20.8)	White: 2,912 (98.5)	NR	NR	NR	NR	Perioperative intravesical chemotherapy: 364 (12.7) Induction intravesical therapy: 694 (23.5)	NR

Study name	Patient population	N	Age (Years)	Gender n (%)	Race/ ethnicity	Disease duration	Stage distribution	ECOG PS	Comorbidities	Treatment history, n (%)	Surgery history, n (%)
Canter 2014 (2)	NMIBC; High-Grade T1 Bladder Cancer	222	Mean (SD): 66.6 (11.28) Median (range): 66.8 (29.2-93)	Male: 198 (89) Female: 24 (11)	White: 199 (89.6) Others: 23 (10.4)	NR	TNM stage- T1: 191 (86) Isolated high-grade T1: 31 (14)	NR	CCI- Mean (SD): 2.63 (2)	Peri-operative mitomycin C (1 dose): 41 (18.6) Induction BCG: 175 (78.8) Induction mitomycin C: 1 (1.4) Mean (range) BCG Treatments: 5.80 (0-28)	NR
Chamie 2013 (27)	Non-metastatic, high-grade (poorly or undifferentiated), urothelial NMIBC	7,410	NR	Male: 5,597 (75.5) Female: 1,813 (24.5)	White: 6,742 (91) Black: 235 (3.2) Hispanic: 188 (2.5) Others: 245 (3.3)	NR	TNM stage- T1: 4258 (57.5) Ta: 2398 (32.3) Tis: 754 (10.2)	NR	Comorbidity score: Score 0: 4868 (65.7) Score 1: 1635 (22.1) Score 2: 587 (7.9)	NR	NR

Study name	Patient population	N	Age (Years)	Gender n (%)	Race/ ethnicity	Disease duration	Stage distribu tion	ECOG PS	Comorbi dities	Treatment history, n (%)	Surgery history, n (%)
									Score >3: 320 (4.3)		
Chaux 2012 (4)	High-grade papillary urothelial carcinoma of the urinary tract with recurrence but without progression	31	Mean: 68.7	Male: 23 (74) Female: 8 (26)	NR	NR	NR	NR	NR	NR	NR
	High-grade papillary urothelial carcinoma of the urinary tract with recurrence and progression	34	Mean: 69.1	Male: 26 (76) Female: 8 (24)							
Bhat 2020 (89)	IR-NMIBC	130	NR	Male: 94 (72.3) Female: 36 (27.7)	NR	NR	High grade: 94 (74.3) Low grade: 36 (27.7)	NR	NR	NR	NR

Study name	Patient population	N	Age (Years)	Gender n (%)	Race/ ethnicity	Disease duration	Stage distribution	ECOG PS	Comorbidities	Treatment history, n (%)	Surgery history, n (%)
							T stage- T1: 16 (12.3) Ta: 110 (84.6)				
Parsons 2020 (23)	NMIBC who were older than 85 years	102	Mean (range): 88.34 (85.02–96.48)	Male: 71 (69.6) Female: 31 (30.4)	NR	NR	NR	NR	NR	NR	NR
Thomas 2013 (5)	NMIBC with progressive tumors; Stage Pta, Tis, T1	110	Median (range): 73.1 (49-84)	Male: 87 (79) Female: 23 (21)	NR	NR	TNM stage- pTis: 32 (29) pTa: 12 (11) pT1: 66 (60) Tumor grade- 1: 7 (6) 2: 46 (42) 3: 57 (52)	NR	NR	NR	NR

Study name	Patient population	N	Age (Years)	Gender n (%)	Race/ ethnicity	Disease duration	Stage distribution	ECOG PS	Comorbidities	Treatment history, n (%)	Surgery history, n (%)
	NMIBC with recurrent high-risk tumors; Stage Pta, Tis, T1	494	Median (range): 73.2 (40-97)	Male: 384 (78) Female: 110 (22)			TNM stage- pTis: 130 (26) pTa: 139 (28) pT1: 221 (45) Tumor grade- 1: 4 (1) 2: 66 (13) 3: 424 (86)				
Simon 2019 (6)	TaG1 pure urothelial NMIBC	470	Mean (SD): 65.74 (12.5)	Male: 381 (81.1) Female: 89 (18.9)	NR	NR	NR	NR	NR	Surveillance : 463 (98.5) 2nd look: 7 (1.5)	NR
Comperat 2015 (26)	Urinary bladder cancer with recurrence (stages Tis, Ta, T1-T4)	36	Mean (range): 33 (10, 40)	Male: 26 (72) Female: 10 (28)	NR	NR	TNM stage- T1: 3 (8) T2: 4 (11) Tis: 1 (3)	NR	NR	NR	NR

Study name	Patient population	N	Age (Years)	Gender n (%)	Race/ ethnicity	Disease duration	Stage distribu tion	ECOG PS	Comorbi dities	Treatment history, n (%)	Surgery history, n (%)
							Ta: 28 ()78				
Olsson 2013 (3)	Primary stage T1 bladder tumours with recurrence	168	NR	Male: 139 (79) Female: 30 (83)	NR	NR	TNM stage: T1a: 57 (76) T1b: 71 (88) T1c: 41 (75) WHO grade-Stage 2: 30 (83) Stage 3: 139 (79)	NR	NR	NR	NR
Rasmussen 2019 (1)	Bladder cancer	1,942	Median (IQR): 63 (57,71)	Male: 1525 (75.8) Female: 417 (21.5)	NR	NR	NR	NR	NR	NR	NR
Sultana 2021 (29)	NMIBC; G1pTa Urothelial Bladder Carcinoma	428	NR	NR	NR	NR	NR	NR	NR	NR	NR

Study name	Patient population	N	Age (Years)	Gender n (%)	Race/ ethnicity	Disease duration	Stage distribution	ECOG PS	Comorbidities	Treatment history, n (%)	Surgery history, n (%)
Ratanapornsompon 2019 (24)	T1 high-grade NMIBC	70	Mean (SD): 68 (10.4)	Male: 53 (75.1) Female: 17 (24.29)	NR	NR	TNM stage-T1-70 (100)	NR	NR	Previous intravesicular BCG: 5 (7.14) Postoperative intravesicular BCG: 36 (51.43)	TURBT: 21 (30) Radical cystectomy: 22 (31.43) Early cystectomy: 14 (63.64)
Lee 2019 (25)	Primary, solitary, Ta, low-grade bladder cancer with recurrence	66	Mean (SD): 63.1 (11.9)	Male: 55 (83) Female: 11 (17)	Chinese: 51 (77); Non Chinese: 15 (23)	NR	NR	NR	Gross hematuria: 47 (71)	Intravesical mitomycin C: 29 (62)	NR
Yu 2021 (28)	Bladder urachal carcinoma (stage I-IV)	203	Mean (SD): 54.2 (10)	Male: 125 (61.6) Female: 78 (38.4)	NR	NR	Mayo staging- Stage 1: 48 (23.8) Stage 2: 108 (53.5) Stage 3: 23 (11.4)	ECOG 0: 169 (83.3) ECOG 1: 33 (16.3) ECOG 2: 1 (0.5)	Diabetes mellitus: 30 (14.8) Hypertension: 50 (24.6)	Conservative: 0 Surgery alone: 136 (67.0) Neoadjuvant therapy + surgery: 1 (0.5)	Open: 133 (65.5) Laparoscopic: 40 (19.7) Robotic: 30 (14.8) Mass excision method, n (%)

Study name	Patient population	N	Age (Years)	Gender n (%)	Race/ ethnicity	Disease duration	Stage distribution	ECOG PS	Comorbidities	Treatment history, n (%)	Surgery history, n (%)
							Stage 4: 23 (11.4)			Surgery + adjuvant therapy: 66 (32.5) Chemotherapy alone: 0 Radiotherapy alone: 0 Chemotherapy+ Radiotherapy: 0 Neoadjuvant treatment Chemotherapy: 0 Radiotherapy: 1 (100%) Adjuvant treatment Chemotherapy: 64 (97) Radiotherapy: 1 (1.5) Chemotherapy+ Radiotherapy: 1 (1.5)	Partial cystectomy: 168 (82.8) Radical cystectomy: 23 (11.3) TUR-B + partial cystectomy: 12 (5.9) Umbilectomy + median umbilical ligament resection: 12 (5.9) PLND: 47 (23.2)

Study name	Patient population	N	Age (Years)	Gender n (%)	Race/ ethnicity	Disease duration	Stage distribution	ECOG PS	Comorbidities	Treatment history, n (%)	Surgery history, n (%)
										Adjuvant chemotherapy regimen None: 138 (68); Cisplatin + paclitaxel + ifosfamide: 1 (0.5) 5-Fluorouracil + doxorubicin + etoposide: 3 (1.5) 5-Fluorouracil + doxorubicin + mitomycin: 3 (1.5) Methotrexate + 5-Fluorouracil + epirubicin + cisplatin: 4 (2)	

Study name	Patient population	N	Age (Years)	Gender n (%)	Race/ ethnicity	Disease duration	Stage distribu tion	ECOG PS	Comorbi dities	Treatment history, n (%)	Surgery history, n (%)
										Cisplatin + 5-fluorouracil: 22 (10.8) Methotrexate+ vinblastine+ doxorubicin + cisplatin (MV/AC): 5 (2.5) Gemcitabine+ cisplatin (GC): 14 (6.9) Leucovorin +5-fluorouracil +irinotecan (FOLFORI): 2 (1) Folinic acid+ fluorouracil + oxaliplatin (FOLFOX): 6 (3)	

Study name	Patient population	N	Age (Years)	Gender n (%)	Race/ ethnicity	Disease duration	Stage distribution	ECOG PS	Comorbidities	Treatment history, n (%)	Surgery history, n (%)
										5-Fluorouracil + leucovorin: 1 (0.5) Etoposide+ cisplatin (EP): 2 (1) Gemcit + paclitaxel: 2 (1)	
Chu 2021 (88)	NMIBC	52	Median (range): 71 (65-78)	Male: 38 (73) Female: 14 (27)	NR	NR	NR	NR	NR	NR	NR
**Gastric cancer (1 study)**											
Kraja 2021 (33)	Gastric cancer (stage IB-III)	180	Mean (SD; range): 58.9 (9.8; 26-80)	NR	NR	NR	Stages- Stage I: NR (6.5) Stage II: (38.4) Stage III: NR (55)	NR	NR	Adjuvant treatment: NR (88.3) Neoadjuvant treatment: NR (8.9) Palliative treatment: NR (1.1)	Subtotal gastrectomy: NR (57.8) Total gastrectomy: NR (33.9) Inoperable: NR (8.3)

Study name	Patient population	N	Age (Years)	Gender n (%)	Race/ ethnicity	Disease duration	Stage distribution	ECOG PS	Comorbidities	Treatment history, n (%)	Surgery history, n (%)
										Adjuvant chemoradiotherapy: NR (35)	R1 resection: 16 (8.9)<br>Incomplete node dissection: NR (46.1)<br>D1 dissection: NR (6.1)<br>D2 dissection: NR (39.4)<br>Incomplete node dissection: NR (46.1)<br>D1 dissection: NR (6.1)

Study name	Patient population	N	Age (Years)	Gender n (%)	Race/ ethnicity	Disease duration	Stage distribution	ECOG PS	Comorbidities	Treatment history, n (%)	Surgery history, n (%)
											D2 dissection: NR (39.4%) D2 dissection: NR (39.4)
**Head and Neck cancer (9 studies)**											
Wilson 2021 (36)	Early-stage oral tongue squamous cell carcinoma with recurrence (pT1N0-pT3N0)	20	NR	Male: 10 (50) Female: 10 (50)	NR	NR	AJCC (7th) stage- T1N0: 15 (75) T1N0: 5 (25) AJCC (8th) stage- T1N0: 13 (65) T2N0: 28 (29) T3N0: 10 (10)	NR	CCI score- ≤4: 17 (85) >4: 3 (15)	Concurrent chemotherapy for those on radiation: 3 (NR)	NR

Study name	Patient population	N	Age (Years)	Gender n (%)	Race/ ethnicity	Disease duration	Stage distribution	ECOG PS	Comorbidities	Treatment history, n (%)	Surgery history, n (%)
Kim 2012 (39)	Head and neck squamous cell carcinoma with local recurrence	324	Mean (SD): 67.4 (10.8)	Male: 232 (71.6) Female: 92 (28.4)	NR	NR	NR	NR	**CCI-** Mean (SD): 0.7 (1.0)	NR	NR
Kanatas 2014 (37)	UK	Primary oral squamous cell carcinoma	98	Male: 167 (57) Female: 125 (43)	NR	NR	T stage- T3-T4: 90 (31) Clinical stage- 3-4: 119 (41) Overall clinical stage- 0-1: 98 (NR) 2: 73 (NR) 3: 28 (NR) 4: 91 (NR)	NR	NR	Operation and adjuvant radiotherapy: 90 (31), Chemoradiotherapy alone: 22 (8)	Operation alone: 180 (62)

Study name	Patient population	N	Age (Years)	Gender n (%)	Race/ ethnicity	Disease duration	Stage distribution	ECOG PS	Comorbidities	Treatment history, n (%)	Surgery history, n (%)
Leoncini 2015 (84)	Head and neck squamous cell carcinoma (stage I-IV)	801	73	Male: 637 (79.5) Female: 164 (20.5)	NR	NR	Stage- I: 124 (25.6) II: 107 (22.1) III: 91 (18.8) IV: 163 (33.6)	NR	NR	NR	Surgical: 208 (53.1) Surgical, Radiation: 104 (26.5) Surgical, Radiation and Chemotherapy: 80 (20.4)
Brandstorp-Boesen 2018 (34)	Laryngeal squamous cell carcinoma with recurrence (stage I-IV)	368	NR	Male: 322 (88) Female: 46 (12)	NR	NR	AJCC stage- I: 87 (24) II: 107 (29) III: 76 (21) IV: 98 (26)<br><br>Early stage: 194(53)	NR	NR	Radiotherapy: 271 (74) Transoral lasermicrosurgery: 38 (10) Total laryngectomy: 41 (11) Chemo-radiotherapy: 18 (5) Palliative/ no treatment: 0	NR

Study name	Patient population	N	Age (Years)	Gender n (%)	Race/ ethnicity	Disease duration	Stage distribution	ECOG PS	Comorbidities	Treatment history, n (%)	Surgery history, n (%)
							Advanced: 174 (47) TNM stage- T1: 90 (24) T2: 120 (33) T3: 85 (23) T4: 73 (20) N0: 300 (82) N1: 23 (6) N2+: 45 (12) M0: 368 (100) M1: 0				
Park 2017 (35)	Previously untreated salivary gland cancer (stage I-IV)	240	Median (range): 52 (13-80)	Male: 127 (53) Female: 113 (47)	NR	NR	TNM stage- T1: 61 (25.4) T2: 70 (29.2)	NR	Charlson-comorbidity scale: 0–2: 223 ≥3: 17	Surgery + RT: 150 (62.5) Surgery + CRT: 13 (5.4)	Surgery: 77 (32.1)

Study name	Patient population	N	Age (Years)	Gender n (%)	Race/ ethnicity	Disease duration	Stage distribution	ECOG PS	Comorbidities	Treatment history, n (%)	Surgery history, n (%)
							T3: 79 (32.9) T4: 30 (12.5) N0: 179 (74.6) N1: 7 (2.9) N2: 54 (22.5)				
Jung 2014 (38)	Head and neck squamous cell carcinoma (stage I-IV)	520	NR	NR	NR	NR	NR	NR	NR	NR	NR
Chang 2017 (85)	Head and neck squamous cell carcinoma, Non-locoregional recurrent (stage I-IV)	28,664	NR	Male: 26174 (85.29) Female: 2490 (88.52)	NR	NR	NR	NR	NR	NR	NR

Study name	Patient population	N	Age (Years)	Gender n (%)	Race/ ethnicity	Disease duration	Stage distribution	ECOG PS	Comorbidities	Treatment history, n (%)	Surgery history, n (%)
	Head and neck squamous cell carcinoma, Locoregional recurrent (stage I-IV)	4,839	NR	Male: 4516 (14.71) Female: 323 (11.48)	NR	NR	NR	NR	NR	NR	NR
Merja 2019 (83)	Laryngeal cancer (stage I-IV; T1-T4)	71	NR	NR	NR	NR	NR	NR	NR	NR	NR
**Melanoma (22 studies)**											
Kolla 2021 (7)	Primary acral lentiginous melanoma (stage IIB-IIIC)	433	Mean: 65 Median (range): 66 (8 to 97)	Male: 202 (46.7) Female: 231 (53.3)	White: 358 (82.7) Black: 51 (11.8) Asian: 7 (1.6) Hispanic: 12 (2.8) Others: NR (1.2)	NR	AJCC stage- Stage 0: 19 (4.4) Stage I: 151 (34.9) Stage II: 118 (27.2) Stage III: 110 (25.4) TNM stage-	NR	NR	Immunotherapy: 7 (6.1) interferon: 9 (7.8) Chemotherapy: 5 (4.3) vaccine trial: 4 (3.5) None: 35 (30.4)	Lymph node dissection (for stage III and IV): 67 (58.3)

Study name	Patient population	N	Age (Years)	Gender n (%)	Race/ ethnicity	Disease duration	Stage distribution	ECOG PS	Comorbidities	Treatment history, n (%)	Surgery history, n (%)
							Tis or T IA: 105 (24.3)				
Bleicher 2020 (40)	Stage II melanoma	580	Median (range): 62 (48-74)	Male: 352 (60.7) Female: 228 (39.3)	NR	NR	AJCC stage- I: 3 (0.6) II: 3 (0.6) III: 39 (7.1) IV/V: 501 (91.8)	NR	NR	NR	NR
Feigelson 2019 (10)	Invasive melanoma (Stage I-IV)	1931	NR	Male: 1,116 (57.8) Female: 815 (42.2)	Non-hispanic whites: 1,888 (97.8) Hispanic: 21 (1.1) Others: 22 (1.1)	NR	NR	NR	Comorbidity score- Score 0: 1110 (57.5) Score 1-2: 533 (27.6) Score 3+: 288 (14.9)	Radiation: 24 (1.2) Chemotherapy: 16 (0.8) Biologic response modulators: 67 (3.5)	Surgery: 1887 (97.7)
Jang 2020 (13)	Melanoma (IIB/C or IIIA)	1,316	NR	NR	NR	NR	AJCC stage-	NR	NR	NR	NR

Study name	Patient population	N	Age (Years)	Gender n (%)	Race/ ethnicity	Disease duration	Stage distribution	ECOG PS	Comorbidities	Treatment history, n (%)	Surgery history, n (%)
							IIB/C: 1174 (NR) IIIA: 142 (NR)				
Tarhini 2018 (43)	Non-metastatic melanoma	6,400	Mean (SD): 53.8 (13.5)	Male: 3,476 (54.3) Female: 2,924 (45.7)	NR	NR	NR	NR	Comorbidities affecting >10% of the study sample- Hypertension: 2405 (37.6) Actinic keratosis: 960 (15) Diabetes, uncomplicated: 745 (11.6) Cardiac arrhythmias: 615 (9.6)	NR	NR

Study name	Patient population	N	Age (Years)	Gender n (%)	Race/ ethnicity	Disease duration	Stage distribu tion	ECOG PS	Comorbi dities	Treatment history, n (%)	Surgery history, n (%)
Ertekin 2021 (51)	Cutaneous melanoma (initial stage IA-IIID) with recurrence	784	Mean (SD): 58.27 (16.75)	Male: 454 (57.9) Female: 330 (42.1)	Caucasian: 99 (NR)	NR	AJCC stage- Stage I: 30 (4.9) Stage II: 283 (46.5) Stage III: 239 (39.3) Stage IV: 56 (9.2)	NR	NR	NR	NR
Loidi 2021 (8)		Locoregional recurrence: 48	Mean (SD): 59.78 (13.35) Median: 62.92	Male: 24 (50) Female: 24 (50)	NR	NR	AJCC stage- I: 11 (24) II: 16 (35) III: 19 (41)	NR	NR	NR	NR

Study name	Patient population	N	Age (Years)	Gender n (%)	Race/ ethnicity	Disease duration	Stage distribution	ECOG PS	Comorbidities	Treatment history, n (%)	Surgery history, n (%)
	Patients with melanoma who underwent sentinel lymph node biopsy who had recurrence (stage I-III)	Haemato-visceral recurrence: 46	Mean (SD): 54.15 (14.52) Median: 49.91	Male: 31 (67) Female: 15 (33)	NR	NR	AJCC stage- I: 3 (7) II: 10 (22) III: 32 (71)	NR	NR	NR	NR
Leeneman 2019 (45)	Localized and regionally advanced cutaneous melanoma (stage I-III)	Stage I: 2,299 Stage II: 565 Stage III: 229	Median (IQR): Stage I: 54 (43-64) Stage II: 63 (50-74) Stage III: 58 (45-69)	Male: Stage I: 915 (40) Stage II: 286 (51) Stage III: 138 (60) Female: Stage I: 1,384 (60) Stage II: 279 (49)	NR	NR	AJCC stage- Stage I: 2,299 (100) Stage II: 565 (100) Stage III: 229 (100)	NR	NR	NR	NR

Study name	Patient population	N	Age (Years)	Gender n (%)	Race/ ethnicity	Disease duration	Stage distribution	ECOG PS	Comorbidities	Treatment history, n (%)	Surgery history, n (%)
				Stage III: 91 (40)							
Sarac 2020 (9)	Superficially spreading melanoma, nodular melanoma, lentigo malignant melanoma, acrolentiginous melanoma, and others who had metastasized locoregionally or distantly (Stage IA-IIC)	1,537	NR	Male: 798 (51.9) Female: 739 (48.1)	NR	NR	AJCC stage- IA: 267 (17.4) IB: 513 (33.4) IIA: 385 (25) IIB: 283 (18.4) IIC: 89 (5.8) Clinical stage- Distant: 457 (29.7) Locoregional: 1,080 (70.3)	NR	NR	NR	NR

Study name	Patient population	N	Age (Years)	Gender n (%)	Race/ ethnicity	Disease duration	Stage distribu tion	ECOG PS	Comorbi dities	Treatment history, n (%)	Surgery history, n (%)
Rockberg 2016 (48)	Cutaneous malignant melanoma (stage I-IV)	3,554	Mean (SD): 61.3 (62.9)	NR	NR	NR	AJCC stage- I: 2,523 (71) II: 746 (21) III: 239 (6.7) IV: 46 (1.3)	NR	NR	NR	Excision with suture: 3216 (90.5) Excision with skin graft: 93 (2.6) Excision with pedicle flap: 38 (1.1) Other: 80 (2.3) Unspecif ied surgery: 64 (1.8) Amputat ion: 25 (0.7)
Rasmussen 2019 (1)	Non-metastatic malignant melanoma	13,50 9	Median (IQR): 67 (58,74)	Male: 6181 (45.8)	NR	NR	NR	NR	NR	NR	NR

Study name	Patient population	N	Age (Years)	Gender n (%)	Race/ ethnicity	Disease duration	Stage distribution	ECOG PS	Comorbidities	Treatment history, n (%)	Surgery history, n (%)
				Female: 7328 (54.2)							
Osella-Abate 2015 (12)	Stage I to II melanoma, late recurrence	77	Median (range): 48 (23-79)	Male: 31 (40) Female: 46 (60)	NR	NR	AJCC stage- II: 9 (NR) III: 47 (NR) IV: 20 (NR)	NR	NR	NR	NR
	Stage I to II melanoma, disease free	1,295	Median (range): 50 (12-84)	Male: 515 (40) Female: 780 (60)			AJCC stage- II: 412 (NR) III: 751 (NR) IV: 123 (NR)				
Quhill 2021 (42)	Uveal melanoma (metastatic excluded)	182	NR	NR	NR	NR	NR	NR	NR	Globe sparing treatment: NR (74)	NR
Ogata 2021 (46)	Stage III sentinel node (SN)-positive melanoma	119	Mean (SD): 57.5 (17.2)	Male: 61 (51.3)	NR	NR	AJCC stage- Stage IIIA: 17 (14.3)	NR	NR	Adjuvant therapy: 30 (25.2) Nivolumab: 14 (NR)	NR

Study name	Patient population	N	Age (Years)	Gender n (%)	Race/ ethnicity	Disease duration	Stage distribution	ECOG PS	Comorbidities	Treatment history, n (%)	Surgery history, n (%)
				Female: 58 (48.7)			Stage III B: 18 (15.1) Stage III C: 74 (62.2) Stage III D: 10 (8.4)			pembrolizumab: 6 (NR) Ipilimumab: 1 (NR) Dabrafenib/trametinib: 9 (NR)	
Chen 2021 (44)	Primary conjunctival melanoma (stage T1-T3)	20	Mean (SD): 57.8 (15.9)	Male: 12 (60) Female: 8 (40)	NR	NR	TNM stage- T1: 4 (20) T2: 7 (35) T3: 9 (45)	NR	NR	NR	NR

Study name	Patient population	N	Age (Years)	Gender n (%)	Race/ ethnicity	Disease duration	Stage distribution	ECOG PS	Comorbidities	Treatment history, n (%)	Surgery history, n (%)
Von Schuckman 2019 (11)	Newly diagnosed localized melanoma at a high risk of metastasis, with recurrence according to AJCC 8 or AJCC 7 (stage IB-IIC)	AJCC 8: 94 AJCC 7: 99	NR	AJCC 8- Male: 65 (69.1) Female: 29 (30.9) AJCC 8-NR	NR	NR	AJCC 8 group- IB: 20 (26) IIA: 29 (37.7) IIB: 18 (23.4) IIC: 10 (13) AJCC 7 group- IB: 24 (5.5) IIA: 30 (16.9) IIB: 18 (19.1) IIC: 5 (24.4) TNM stage- *AJCC 8 group*- T1b: 6 (6.4) T2a:17 (18.1)	NR	NR	NR	NR

Study name	Patient population	N	Age (Years)	Gender n (%)	Race/ ethnicity	Disease duration	Stage distribution	ECOG PS	Comorbidities	Treatment history, n (%)	Surgery history, n (%)
							T2b:14 (14.9)<br>T3a:21 (22.3)<br>T3b: 12 (12.8)<br>T4a: 8 (8.5)<br>T4b: 16 (17.0)<br>*AJCC 7 group*-<br>T1b: 10 (NR)<br>T2a: 17 (NR)<br>T2b: 14 (NR)<br>T3a: 22 (NR)<br>T3b: 12 (NR)<br>T4a: 8 (NR)<br>T4b: 16 (NR)				

Study name	Patient population	N	Age (Years)	Gender n (%)	Race/ ethnicity	Disease duration	Stage distribution	ECOG PS	Comorbidities	Treatment history, n (%)	Surgery history, n (%)
Chakera 2019 (41)	Subungual melanoma of the hand (stage IA-IIIB/C)	103	Median (range): 58 (6-93)	Male: 63 (61) Female: 40 (39)	White: 101 (98.1) Asian: 1 (0.97) Australian aboriginal heritage: 1 (0.97)	NR	AJCC stage- IA: 8 (8) IB: 15 (15) IB/II: 6 (6) IIA: 15 (15) IIB: 18 (18) IIC: 10 (10) IIIA: 2 (2) IIIB: 2 (2) IIIC: 17 (17) IIIB/C: 1 (1)	NR	NR	NR	Distal amputation: 77 (75) Proximal amputation: 21 (21) Amputation level unknown: 1 Wide excision skin graft: 3 (3) Local excision only: 1 (1)
Varey 2017 (47)	Neurotropic and non-neurotropic cutaneous melanoma (stage I-III)	Neurotropic: 671	Mean (SD)- Neurotropic: 65 (15)	Male- Neurotropic: 484 (72)	NR	NR	AJCC stage- Neurotropic: I/II: 608 (92)	NR	NR	NR	NR

Study name	Patient population	N	Age (Years)	Gender n (%)	Race/ ethnicity	Disease duration	Stage distribution	ECOG PS	Comorbidities	Treatment history, n (%)	Surgery history, n (%)
		Non-neurotropic: 718	Non-neurotropic: 56 (17) Median (range) - Neurotropic: 67 (17-96) Non-neurotropic: 57 (15-93)	Non-neurotropic: 403 (56) Female-Neurotropic: 187 (28) Non-neurotropic: 315 (44)			III: 55 (8) Missing: 8 (NR) Non-neurotropic: I/II: 659 (92) III: 54 (8) Missing: 5 (NR)				
Tas 2019 (49)	Local (stage I–II) and regional (stage III) cutaneous melanoma, relapsed	365	NR	Male: 228 (62.5) Female: 137 (37.5)	NR	NR	NR	NR	NR	Adjuvant therapy: 153 (41.9)	NR

Study name	Patient population	N	Age (Years)	Gender n (%)	Race/ ethnicity	Disease duration	Stage distribution	ECOG PS	Comorbidities	Treatment history, n (%)	Surgery history, n (%)
Tas 2017 (50)	Early-stage cutaneous melanoma with recurrence (stage I-III)	332	Median (range): 50 (16-87)	Male: 207 (62.3) Female: 125 (37.7)	NR	NR	AJCC stage- I/II: 176 (53) III: 156 (47) Stage (not specified)- 1: 82 (52.6) ≥2: 74 (47.4)	NR	NR	NR	NR
Leung 2022 (86)	Early-stage primary cutaneous melanomas (stage I or II)	1,244	NR	NR	NR	NR	NR	NR	NR	NR	NR
Burns 2019 (52)	Stage III melanoma	370	NR	NR	NR	NR	NR	NR	NR	NR	NR
**NSCLC (7 studies)**											
Karacz 2020 (14)	NSCLC (stage I-III)	332	NR	NR	NR	NR	NR	NR	NR	NR	NR

**Study name**	**Patient population**	**N**	**Age (Years)**	**Gender n (%)**	**Race/ ethnicity**	**Disease duration**	**Stage distribu tion**	**ECOG PS**	**Comorbi dities**	**Treatment history, n (%)**	**Surgery history, n (%)**
Buck 2015 (57)	Stage IB to IIIA NSCLC	609	Mean (SD): 64.75 (9.9)	Male: 322 (52.9) Female: 287 (49.8)	White: 358 (58.8) Black: 61 (10) Asian: 6 (1) Hispanic: 2 (0.3) Others: 5 (0.8)	NR	NR	NR	NR	Adjuvant therapy: 345 (56.7); Second adjuvant therapy: 42 (6.9); Third adjuvant therapy: 5 (0.8);	Bilobect omy: 27 (4.4); Lobecto my: 512 (84.1); Pneumo nectomy: 53 (8.7); Sleeve lobectom y: 2 (0.3) Not specified : 15 (2.5)

Study name	Patient population	N	Age (Years)	Gender n (%)	Race/ ethnicity	Disease duration	Stage distribution	ECOG PS	Comorbidities	Treatment history, n (%)	Surgery history, n (%)
										Adjuvant Therapy, Most Common First Regimen: Carboplatin: 23 (6.7); Carboplatin, paclitaxel: 103 (29.9); Carboplatin, pemetrexed: 18 (5.2); Cisplatin: 48 (13.9); Cisplatin, docetaxel: 39 (11.3); Cisplatin, pemetrexed: 25 (7.2); Cisplatin, vinorelbine: 28 (8.1); Other: 61 (17.7);	

Study name	Patient population	N	Age (Years)	Gender n (%)	Race/ ethnicity	Disease duration	Stage distribution	ECOG PS	Comorbidities	Treatment history, n (%)	Surgery history, n (%)
										Adjuvant Therapy, Most Common Second Regimen: Carboplatin: 8 (19.0); Carboplatin, docetaxel: 4 (9.5); Carboplatin, paclitaxel: 11 (26.2); Carboplatin, pemetrexed: 5 (11.9) Other: 14 (33.3)	
Martin 2022 (15)	Stage III NSCLC	231	Median (range): 65 (21-89)	Male: 140 (60.6) Female: 91 (39.4)	Caucasian: NR (29) East Asian: 0.4 (23) Hispanic: 23 (10)	NR	AJCC stage: Stage III: 231 (100) Stage IIIA: 103 (53.4)	ECOG 0: 65 (48.5) ECOG 1: 54 (40.3) ECOG >2: 15 (11.2)	NR	Chemotherapy alone: 50 (24.8) Concurrent chemoradiotherapy: 35 (17.3)	Surgical resection: overall: 58 (28.7) Other surgery: NR (7.9)

Study name	Patient population	N	Age (Years)	Gender n (%)	Race/ ethnicity	Disease duration	Stage distribution	ECOG PS	Comorbidities	Treatment history, n (%)	Surgery history, n (%)
					Others: 1 (0.4)		Stage IIIB: 90 (46.6)<br>TNM stage:<br>T1a: 9 (3.9)<br>T1b: 5 (2.2)<br>T1c: 3 (1.3)<br>T2a: 34 (14.9)<br>T2b: 22 (9.6)<br>T3: 74 (32.5)<br>T4: 65 (28.5)<br>N0: 15 (6.6)<br>N1: 31 (13.6)			Sequential chemoradiotherapy: 21 (10.4)<br>Radiotherapy alone: NR (6.4)	Concurrent chemoradiotherapy + surgery: NR (4)<br>Surgery+ chemotherapy: NR (5.9)<br>Surgery alone: NR (6.4)

Study name	Patient population	N	Age (Years)	Gender n (%)	Race/ ethnicity	Disease duration	Stage distribution	ECOG PS	Comorbidities	Treatment history, n (%)	Surgery history, n (%)
Chouaid 2018 (53)	Completely resected stage IB-IIIA NSCLC	831	NR	Male: 513 (61.7) Female: 318 (38.3)	NR	NR	Stage-IB: 239 (28.8) IIA: 179 (21.5) IIB: 165 (19.9) IIIA: 248 (29.8)	ECOG 0 or 1: 416 (95.6)	Cardiovascular disease: 333 (40.1) COPD or asthma: 215 (25.9) History of cancers other than NSCLC: 91 (11) Other or no data available: 611 (73.5)	NR	Systematic lymph node dissection: 598 (72)
Slim 2021 [50]	Patients operated for NSCLC (stage pT IA-pT IV; resectable)	67	Mean (SD): 61 (8)	Male: 64 (95.5) Female: 3 (4.5)	NR	NR	TNM stage: pT IA: 10 (14.9) pT IB: 4 (5.9)	NR	NR	Neoadjuvant CT+ surgery: 2 (2.9)	Lobectomy: 42 (62.68) Pneumonectomy: 8 (11.94)

Study name	Patient population	N	Age (Years)	Gender n (%)	Race/ ethnicity	Disease duration	Stage distribution	ECOG PS	Comorbidities	Treatment history, n (%)	Surgery history, n (%)
							pT IIA: 6 (8.9) pT IIB: 18 (26.9) IIIA: 25 (37.31) IIIB: 1 (1.5) T4: 3 (4.47)			Surgery+ adjuvant CT: 33 (49.2) Neoadjuvant CT+ surgery + adjuvant CT: 6 (8.95) Surgery+ adjuvant CT+ adjuvant RT: 6 (8.95) Surgery+ adjuvant RT: 2 (2.9) Cerebral surgery + cerebral RT+ thoracic Surgery: 1 (1.49)	Bilobectomy: 4 (5.97) Lobectomy + pariectomy: 4 (5.97) Lobectomy+ tumorectomy: 2 (2.9) Lobectomy + Fowler resection: 1 (1.49) Lobectomy + cumen resection: 1 (1.49)

Study name	Patient population	N	Age (Years)	Gender n (%)	Race/ ethnicity	Disease duration	Stage distribution	ECOG PS	Comorbidities	Treatment history, n (%)	Surgery history, n (%)
										Neoadjuvant CT+ surgery+ adjuvant CT+ adjuvant RT: 1 (1.49) Neoadjuvant CT+ Neoadjuvant RT+ Surgery+ Adjuvant CT: 1 (1.49)	Lobectomy + azygos vein arch resection: 1 (1.49) Tumorectomy: 1 (1.49) Wedge resection + lobectomy: 2 (2.9)
Li 2019 (55)	Primary lung adenocarcinoma (stage I-IIIA)	882	NR	NR	NR	NR	NR	NR	NR	NR	NR
Kumar 2019 (56)	NSCLC (stage I)	33	NR	NR	NR	NR	NR	NR	NR	NR	Surgery-wedge resection: 22 (NR)

Study name	Patient population	N	Age (Years)	Gender n (%)	Race/ ethnicity	Disease duration	Stage distribution	ECOG PS	Comorbidities	Treatment history, n (%)	Surgery history, n (%)
											Radiation-definitive radiation: 11 (NR)
**RCC (3 studies)**											
Dabestani 2016	Newly diagnosed patients with RCC (non-metastatic)	3,107	Mean: 67 Median (range): 67 (9-97)	Male: 1857 (59.7) Female: 1250 (40.2)	NR	NR	TNM stage- T1a: 1,003 (NR) T1b: 826 (NR) T2: 595 (NR) T3: 624 (NR) T4: 35 (NR) Tx: 24 (NR) N0: 2134 (NR)	NR	NR	NR	Radical nephrectomy: 2507 (NR) Partial nephrectomy: 383 (NR) MITs: 77 (NR) No surgical treatment: 140 (NR)

Study name	Patient population	N	Age (Years)	Gender n (%)	Race/ ethnicity	Disease duration	Stage distribu tion	ECOG PS	Comorbi dities	Treatment history, n (%)	Surgery history, n (%)
							N1-N2: 123 (NR) Nx:850 (NR)				
Thorstenson 2015 (59)	Newly diagnosed RCC (T0-T4)	8,556	Median (range): 67 (23-105)	Male: 5256 (61) Female: 3300 (39)	NR	NR	TNM stage- T1a: 2511 (29) T1b: 2007 (23) T2: 1506 (18) T3: 2017 (24) T4: 287 (3) Tx: 213 (2) Fuhrman grade- 1: 926 (12)	NR	NR	NR	Radical nephrectomy: NR (74) Partial nephrectomy: (NR) 23 Minimally invasive treatments- Cryotherapy and Radiofrequency ablation: NR (2)

Study name	Patient population	N	Age (Years)	Gender n (%)	Race/ ethnicity	Disease duration	Stage distribution	ECOG PS	Comorbidities	Treatment history, n (%)	Surgery history, n (%)
							2: 3496 (44)<br>3: 2072 (26)<br>4: 705 (9)<br>GX: 657 (8)				
Alvarado 2019 (60)	Tubulocystic RCC (pT2-pT4)	25	Mean (range): 52 (26-72)	NR	NR	NR	NR	NR	NR	NR	NR
**TNBC (18 studies)**											
Kaplan 2017 (61)	Primary TNBC (stage I-III)	771	Median (range): 53 (25-93)	Female: 771 (100)	White: 84%	NR	AJCC Stage I-<br>Age <65: NR (31)<br>Age 65-74: NR (48)<br>Age 75+: NR (3)	NR	NR	NR	NR

Study name	Patient population	N	Age (Years)	Gender n (%)	Race/ ethnicity	Disease duration	Stage distribution	ECOG PS	Comorbidities	Treatment history, n (%)	Surgery history, n (%)
Matro 2015 (19)	Overall breast cancer (No subgroup data for TNBC); Stage III with recurrence	175	Median (range): 53 (22-91)	Female: 175 (100)	NR	NR	NR	NR	NR	NR	NR
Steward 2014 (63)	Stage I–III TNBC, Locoregional recurrence	19	NR	NR	White: 11 (57.9) African-American: 8 (42.1)	NR	Clinical Stage- I: 6 (40) II: 7 (46.7) III: 2 (13.3)<br><br>TNM stage- T1: 6 (37.5) T2: 8 (50) T3: 1 (6.25) T4: 1 (6.25) N0: 12 (75)	NR	NR	Radiation: 14 (73.7) Adjuvant treatment: 10 (76.9) Neoadjuvant therapy: 3 (23.1)	Partial mastectomy: 10 (52.6) Simple mastectomy: 3 (15.8) Modified radical mastectomy: 6 (31.6)

Study name	Patient population	N	Age (Years)	Gender n (%)	Race/ ethnicity	Disease duration	Stage distribu tion	ECOG PS	Comorbi dities	Treatment history, n (%)	Surgery history, n (%)
							N1: 4 (25) N2/N3-0				
	Stage I–III TNBC, Distant recurrence	70			White: 36 (51.4) African-America n: 33 (47.1) Others: 1 (1.4)		Clinical Stage- I: 12 (19) II: 33 (52.4) III: 18 (28.6) TNM stage- T1: 15 (23.8) T2: 34 (54) T3: 7 (11.1) T4: 7 (11.1) N0: 39 (59.1) N1: 17 (25.8) N2/N3: 10 (15.1)			Radiation:47 (67.1) Adjuvant treatment: 28 (43.1) Neoadjuvant therapy: 37 (56.9)	Partial mastectomy: 37 (53.6) Simple mastectomy: 4 (5.8) Modified radical mastectomy: 28 (40.6)

Study name	Patient population	N	Age (Years)	Gender n (%)	Race/ ethnicity	Disease duration	Stage distribution	ECOG PS	Comorbidities	Treatment history, n (%)	Surgery history, n (%)
	Locoregional and distant recurrence	21			White: 13 (61.9) African-American: 7 (33.3) Others: 1 (4.76)		Clinical Stage- I: 1 (5.2) II: 9 (47.4) III: 9 (47.4) TNM stage- T1: 3 (16.7) T2: 7 (38.9) T3: 4 (22.2) T4: 4 (22.2) N0: 7 (35) (59.1) N1: 11 (55) (25.8) N2/N3: 2 (10)			Radiation: 11 (52.4) Adjuvant treatment: 7 (39.0) Neoadjuvant therapy: 11 (61)	Partial mastectomy: 8 (40.0) Simple mastectomy: 1 (5) Modified radical mastectomy: 11 (55)

Study name	Patient population	N	Age (Years)	Gender n (%)	Race/ ethnicity	Disease duration	Stage distribution	ECOG PS	Comorbidities	Treatment history, n (%)	Surgery history, n (%)
Dawood 2012 (70)	Stage I to III TNBC	2,448	Median (range): 50 (21-97)	Female: 2,448 (100)	White: 1596 (65.19) Black: 437 (17.85) Others: 415 (16.95)	NR	Stage- Stage I: 616 (25.2) Stage II: 1256 (51.3) Stage III: 576 (23.5)	NR	NR	Anthracyclines: 1950 (79.6) Taxane: 1614 (65.9) Adjuvant radiation: 1579 (NR)	Breast conservation surgery: 1162 (NR) Mastectomy: 1218 (NR)
Başer 2012 (81)	Early-stage TNBC (stage I-III)	403	Mean (SD): 53.68 (11.31)	Female: 403 (100)	NR	NR	ICD-9-CM- Stages I/II: 299 (74.19) Stage III: 104 (25.81)	NR	Quan-Charlson Comorbidity Index- Mean (SD): 1.9 (2.26)	NR	NR

Study name	Patient population	N	Age (Years)	Gender n (%)	Race/ ethnicity	Disease duration	Stage distribution	ECOG PS	Comorbidities	Treatment history, n (%)	Surgery history, n (%)
Haiderali 2021a (16)	TNBC stage II-IIIB)	308	Mean (SD): 52.1 (11.3)	Female: 308 (100)	White: 175 (56.8) African American: 110 (35.7) Others: 23 (7.4)	NR	Stages according to ICD-9 code of 174.x or ICD-10 code of C50.x- II: 205 (66.6) III: 103 (33.4)	NR	Weighted index of comorbid conditions- Mean (SD): 0.3 (0.7)	NR	NR
Haiderali 2021b	TNBC (stage II-IIIB)	308	Mean (SD): 52.1 (11.3)	Female: 308 (100)	White: 175 (56.8) African American: 110 (35.7) Others: 23 (7.4)	NR	Stages according to ICD-9 code of 174.x or ICD-10 code of C50.x- II: 205 (66.6) III: 103 (33.4)	NR	Weighted index of comorbid conditions- Mean (SD): 0.3 (0.7)	NR	NR

Study name	Patient population	N	Age (Years)	Gender n (%)	Race/ ethnicity	Disease duration	Stage distribution	ECOG PS	Comorbidities	Treatment history, n (%)	Surgery history, n (%)
Tecic 2020 (65)	Early-stage TNBC (stage T1-T3; N0-N3)	152	Median (range): 58 (47-70)	Female: 152 (100)	NR	NR	TNM stage- T1: 57 (37.5) T2: 83 (54.6) T3: 12 (7.9) N0: 90 (59.2) N1: 35 (23) M0: 12 (7.9) M1: 15 (9.9)	NR	With comorbidities: 89 (61)	Chemotherapy- Anthracyclines: 71 (55.5) Anthracyclines and taxanes: 43 (33.6) Cyclophosphamide-methotrexate-5-fluorouracil): 11 (8.6) Taxanes only: 1 (0.8) Other chemotherapy: 2 (1.6)	Conservative surgery: 93 (61.2) Radical surgery: 59 (38.8) Axillary dissection: 150 (98.7)
Ignatov 2018 (17)	Non-metastatic, primary TNBC	1,374	Median (range): 60 (25-96)	Female: 1,374 (100)	NR	NR	Stage- I: 23 (1.7) II: 300 (22.5) III: 1,012 (75.8)	NR	NR	Radiotherapy: 970 (67.1) Chemotherapy- Anthracycline-based: 308 (23.1)	Breast conserving: 898 (50.1) Mastectomy: 444 (48.4)

Study name	Patient population	N	Age (Years)	Gender n (%)	Race/ ethnicity	Disease duration	Stage distribution	ECOG PS	Comorbidities	Treatment history, n (%)	Surgery history, n (%)
							Missing: 39 (NR) Histologic grade- 1: 23 (1.7) 2: 300 (22.5) 3: 1012 (75.8) Missing: 39			Taxane-based: 71 (5.3) Both: 593 (44.4) Other: 29 (2.2) Missing: 39	Missing: 17 (NR)
Van 2016	TNBC (Stage T1-2N0)	2,548	Mean (SD): 56 (14)	Female: 2,548 (100)	NR	NR	TNM stage- pN0: 1929 (75.7) pN0(i+): 72 (2.8) pN1mi: 115 (4.5) pN1: 313 (12.3) N2: 64 (2.5)	NR	NR	Adjuvant chemotherapy: 1,534 (NR)	Axillary lymph node dissection (ALND): 54 (2.1) SLNB and completion ALND: 508 (19.9)

Study name	Patient population	N	Age (Years)	Gender n (%)	Race/ ethnicity	Disease duration	Stage distribution	ECOG PS	Comorbidities	Treatment history, n (%)	Surgery history, n (%)
							N3: 27 (1.1) Unknown: 2.8 (1.1)				Breast conserving therapy: 1624 (63.7) Mastectomy: 761 (29.9) Mastectomy and radiotherapy: 118 (4.6) Lumpectomy-only: 45 (1.8)
James 2019 (62)	Non-metastatic TNBC	1,390	Median (range): 55 (2-95)	Female: 1390 (100)	NR	NR	TNM stage- T1: 606 (44) T2: 631 (45) T3: 95 (6) T4: 51 (4)	NR	NR	Adjuvant: 877 (63) Neoadjuvant:71 (5) Unknown: 8 (1) No treatment: 434 (31)	Wide local excision: 610 (44) Mastectomy: 761 (55)

Study name	Patient population	N	Age (Years)	Gender n (%)	Race/ ethnicity	Disease duration	Stage distribution	ECOG PS	Comorbidities	Treatment history, n (%)	Surgery history, n (%)
							Tx: 7 (1) N0: 843 (60) N1: 319 (23) N2: 119 (9) N3: 86 (6) Nx: 23 (2) Tumor grade- 1: 29 (2) 2: 239 (17) 3: 1095 (79) Unknown: 27 (2)			Radiotherapy: 911 (66)	Axillary surgery only: 5 (0.003) No primary surgery: 14 (1)
Gonçalves 2018 (66)	TNBC (stage I-III)	87	Mean (SD): 57 (13)	Female: 87 (100)	White: 57 (67.9) Non-White: 27 (32.1)	NR	Stage- I: 18 (20.9) II: 35 (40.7) III: 33 (38.4)	NR	NR	Chemotherapy: 73 (83.9)	Radical Surgery: 53 (61.6) Conservative surgery: 33 (38.4)

Study name	Patient population	N	Age (Years)	Gender n (%)	Race/ ethnicity	Disease duration	Stage distribution	ECOG PS	Comorbidities	Treatment history, n (%)	Surgery history, n (%)
							Tumor grade- I: 10 (14.9) II: 33 (49.3) III: 24 (35.8)				
Villarreal 2021 (87)	TNBC (stage II-III)	54	Median: 36 (range: 21-40)	Female: 54 (100)	NR	NR	NR	NR	NR	NR	NR
Suhani 2017 (67)	Non-metastatic TNBC (stage I-III)	98	Mean: 44.4	Female: 98 (100)	NR	NR	AJCC stage- I: 2 (2) II: 45 (45.9) III: 49 (50) Unknown: 2 (2)	NR	NR	Neoadjuvant chemotherapy: 60 (61.2) Taxanes (among neoadjuvant chemotherapy): 27 (27.6)	Upfront surgery: 38 (38.8) Mastectomy: 23 (60.5) Breast conservative surgery: 15 (39.5)

Study name	Patient population	N	Age (Years)	Gender n (%)	Race/ ethnicity	Disease duration	Stage distribution	ECOG PS	Comorbidities	Treatment history, n (%)	Surgery history, n (%)
Gal 2018	Older patients with early-stage TNBC (non-metastatic; T1-T4; N0-N4)	NR	NR	NR	NR	NR	NR	NR	NR	NR	NR
Eralp 2014 (20)	Non-metastatic TNBC (stage I-III)	316	Mean (range): 49.7 (24-82)	Female: 316 (100)	NR	NR	Pathological stage-Stage 1: 84 (26.6) Stage 2: 164 (51.9) Stage 3: 68 (21.3)<br><br>Grade 1: 6 (1.9)	NR	NR	NR	Mastectomy: 178 (56.3) Breast conserving surgery: 138 (43.6)
Metzger-Filho 2013 (69)	Early-stage TNBC	310	Mean (SD): 52.6 (10.2)	Female: 310 (100)	NR	NR	NR	NR	NR	Chemotherapy: 130 (41.9)	Mastectomy: 163 (52.6)

Study name	Patient population	N	Age (Years)	Gender n (%)	Race/ ethnicity	Disease duration	Stage distribution	ECOG PS	Comorbidities	Treatment history, n (%)	Surgery history, n (%)
											Breast conservation: 147 (47.4) Surgery with radiotherapy planned: 122 (39.4) Surgery without radiotherapy planned: 25 (8.1)
Min Sun Bae 2015 (21)	TNBC with Recurrence (stage I-II)	63	NR	NR	NR	NR	AJCC stage- Stage I: 19 (30.2) Stage II: 44 (69.8)	NR	NR	Adjuvant radiation therapy: 40 (93) Adjuvant chemotherapy:58 (92.1)	Breast conservation:43 (68.3) Mastectomy:20 (31.7)

Study name	Patient population	N	Age (Years)	Gender n (%)	Race/ ethnicity	Disease duration	Stage distribu tion	ECOG PS	Comorbi dities	Treatment history, n (%)	Surgery history, n (%)
										Chemothera py- Adriamycin -based: 49 (84.5) CMF: 9 (15.5)	

Abbreviations: SD: standard deviation, ECOG: eastern cooperative oncology group, NMIBC: non muscle invasive bladder cancer, TNML Tumor, node and metastasis, NR: not reported, BCG: Bacillus Calmette-Guerin, IR NMIBC: intermediate-risk non muscle invasive bladder cancer, WHO: World Health Organization, IQR: inter quartile range, AJCC: American joint committee on cancer, NSCLC: non-small cell lung cancer, COPD: chronic obstructive pulmonary disease.

Table 72: Population characteristics of patients in the humanistic review (n = 5 studies)

Study name	Patient population	N	Age (Years)	Gender (n (%)	Race/ ethnicity	Disease duration	Stage distribution	ECOG performance status	Comorbidities (n (%))	Treatment history	Surgery history
**Bladder cancer (1 study)**											
Smith 2022 (74)	Overall	911	Mean (SD): 67.6 (8.7); Median (Range): 68 (NR)	Male: 468(0.51); Female: 320(35)	White/ Caucasian: 772 (79); Black or African American: 12 (1); American Indian or Alaska Native: 3 (0.3); Asian: 9 (0.9); Native Hawaiian or Pacific Islander: 0 (0); Missing: 175 (18); Hispanic: 16 (2); non-Hispanic: 748 (82); Missing: 147 (16)	NR	NR	NR	None: 282 (31%) 1: 223 (24%) 2: 192 (21%) 3 or more: 196 (22%) Missing: 18 (2%)	Chemotherapy IV: 227 (25); Immunotherapy IV: 55 (6); Radiation therapy: 36 (4); Intravesical therapy: 544 (60); Ureteroscopy w/ biopsy or fulguration: 46 (5); Other: 64 (7)	TURBT: 710 (78); Radical cystectomy: 394 (43); Partial cystectomy: 15 (2); Nephroureterectomy: 42 (5)

	Non-recurrent NMIBC	306	Mean (SD): 68.3(9); Median: 69	Male: 166(0.54); Female: 93(30)	White/Caucasian: 244 (80); Black or African American: 6 (2); American Indian or Alaska Native: 2 (0.6); Asian: 2 (0.6); Native Hawaiian or Pacific Islander: 1 (0.3); Missing: 51 (17); Hispanic: 5 (1.6); non-Hispanic: 247 (81); Missing: 54 (18)	NR	NR	NR	None: 85 (28)<br>1: 85 (28)<br>2: NR<br>3 or more: 63 (21)<br>Missing: 14 (5)	Chemotherapy IV: 11 (4); Immunotherapy IV: 6 (2); Radiation therapy: 1 (0.3); Intravesical therapy: 224 (73); Ureteroscopy w/ biopsy or fulguration: 16 (5); Other: NR	TURBT: 254 (83); Radical cystectomy: 36 (12); Partial cystectomy: 4 (1); Nephroureterectomy: 8 (3)

	Recurrent NMIBC	272	Mean (SD): 66.7(8.4); Median: 68	Male: 127(47); Female: 112(41)	White/Caucasian: 229 (84); Black or African American: 2 (0.7); American Indian or Alaska Native: 1 (0.4); Asian: 2 (0.7%); Native Hawaiian or Pacific Islander: 0 (0); Missing: 38 (14); Hispanic: 5 (2); non-Hispanic: 225 (83); Missing: 42 (15)	NR	NR	NR	None: 88 (32);<br>1: 54 (20)<br>3 or more: 65 (24)<br>Missing: 0	Chemotherapy IV: 13 (5); Immunotherapy IV: 10 (4) Radiation therapy: 2 (0.7); Intravesical therapy: 229 (84); Ureteroscopy w/biopsy or fulguration: 20 (7); Other: NR	TURBT: 252 (93); Radical cystectomy:87 (32); Partial cystectomy: 2 (0.7); Nephroureterectomy: 13 (5)
**Gastric cancer (1 study)**											

Shin 2022 (75)	Stomach cancer survivors who completed primary treatment without metastasis or recurrence	363	Mean (SD): 56.9(9.6); Median (Range): NR(NR)	Male: 193(53.2); Female: 170(46.8)	NR	Mean (SD): 7.3 (NR)	Stage I: 242(66.7); Stage II: 67(18.5); Stage III: 54(1.9)	NR	0: 315 (86.8)<br>1: 35 (9.6)<br>≥2: 13 (3.6)	Surgery + chemotherapy 60 (16.5) Surgery+ Chemotherapy Radiotherapy: 79 (21.8) Adjuvant therapy: 143 (72.58)	Only surgery: 224 (66.7)
	Non-clinical FCRI	221	Mean (SD): NR(NR) Median (Range): NR(NR)	Male: 126(65.3); Female: 95(55.9)	NR	Mean (SD): (NR)	Stage I: 146(60.3); Stage II: 45(67.2); Stage III: 30(55.6)	NR	0: 193 (61.1)<br>1: 19 (57.6)<br>≥2: 9 (64.3)	Surgery + chemotherapy 34 (56.7) Surgery+ Chemotherapy Radiotherapy: 60 (63.3); Adjuvant chemotherapy: 30 (15.2)	Only surgery: 137 (61.20

	Clinical FCRI	142	Mean (SD): NR<br><br>Median (Range): NR	Male: 67(34.7); Female: 75(44.1)	NR	Mean (SD): (NR)	Stage I: 96(39.7); Stage II: 22(32.8); Stage III: 24(44.4)	NR	0: 123 (38.9)<br>1: 14 (42.4)<br>≥2: 5 (35.7)	Surgery + chemotherapy 26 (43.3) Surgery + chemotherapy + radiotherapy: 29 (36.7); Adjuvant chemoradiotherapy: 103 (52.3	Only surgery: 87 (38.8)
**Melanoma (2 studies)**											
Atkinson 2013 (77)	Women within ten days to two years of completing surgical treatment for primary cutaneous melanoma	100	Mean (SD): 54.34(15.44); Median (Range): 53.51(21-90)	Female: 100(100)	Others: Caucasian: 100(100)	Mean (SD): 194.15 (NR); SE: 20.81	Stage I: 8(NR)Stage II: 11(NR) Stage III: 12(NR) Stage IV: 58(NR) Unknown: 4(NR)	NR	NR	NR	NR

Van 2016 (76)	Early stage or locally advanced disease (stage I/II) melanoma	469	Mean (SD): 52.3 (13.6);	Male: 177(38); Female: 292(62)	NR	NR	I/II	NR	None: 246 (56); 1-2: 178 (40); ≥3: 19 (4)	NR	Surgery: 468 (100)
**Mixed tumor types (Bladder cancer and melanoma) (1 study)**											
Leclair 2019 (73)	All cancers	2337	Mean (SD): 56.22(11.19);	Male: 936(39.6); Female: 1411(60.4)	White: Caucasian: 2,100 (Caucasian: 89.9); Hispanic: 66 (2.8); A-A: 116 (5); Others: 45 (1.9); Not indicated/ missing: 22 (0.9)	NR	NR	NR	NR	NR	NR
	Melanoma	139	NR	NR	NR	NR	I-III	NR	NR	NR	NR
	Bladder cancer	89	NR	NR	NR	NR	NR	NR	NR	NR	NR

Abbreviations: ECOG: eastern cooperative oncology group; FCRI: fear of Cancer Recurrence Inventory; NR: not reported SD: standard deviation; TURBT: transurethral resection of bladder tumour, NMIBC: non-muscle invasive bladder cancer, IR-NMIBC: intermediate—risk non-muscle invasive bladder cancer, BCG : Bacillus Calmette-Guerin, TNM: Tumor, node and metastasis, WHO: World Health Organization, IQR: inter quartile range: TUR-B: Transurethral bladder resection, PLND: Pelvic lymph node dissection, AJCC: American joint committee on cancer, CCI: Charlson Comorbidity Index, NSCLC: non-small cell lung cancer, RCC: renal cell carcinoma, TNBC: triple negative breast cancer , ICD: International Statistical Classification of Diseases , ALND: Axillary lymph node dissection.

Table 73: Population characteristics of patients in the economic review (n = 7 studies)

Study name	Tumor types	Study objectives	Study design	Publication type	Country	Data source	Study setting	Time frame	Study perspective	Cost year
**Head and neck cancer (1 study)**										
Kim 2012 (39)	Head and neck cancer (HNC)	The present study aimed to enhance the literature on the treatment patterns and the incremental healthcare resource utilization and economic cost burden of HNC in the US	Case-control study (retrospective administrative claims databased)	Journal article	USA	Thomson Reuters MarketScan Databases	Multi centre	2004 to 2008	Third-party payer perspective	NR
**Melanoma (4 studies)**										
Leeneman 2021 (78)	Cutaneous melanoma	To provide insight into real-world healthcare costs of patients initially diagnosed with localized or regionally advanced melanoma in three Dutch hospitals between 2003 and 2011.	Retrospective cohort study	Journal article	Netherlands	Netherlands Cancer Registry (NCR)	Multi centre	Jan 2003 to Dec 2011	Hospital perspective	2018
Serra 2017 (79)	Cutaneous melanoma	To assess costs related to melanoma in these 4 stages (Stage I-IV) of disease, from the time of the first visit to the dermatologist until therapeutic and follow-up processes end.	Cost of illness	Journal article	Spain	Amounts were extracted from official Spanish publications	NR	NR	NR	2015

Study name	Tumor types	Study objectives	Study design	Publication type	Country	Data source	Study setting	Time frame	Study perspective	Cost year
Jang 2020 (13)	Melanoma	To report Real world recurrence rates, risk factors, and economic impact in patients with resected stage IIB/C or IIIA melanoma.	Retrospective study	Conference abstract	USA	SEER-Medicare data	Single centre	2003 to 2014	NR	2018
Tarhini 2018 (43)	Non-Metastatic melanoma	This study aimed to describe real world patterns of treatment and recurrence in patients with melanoma and to quantify healthcare resource utilization (HRU) and costs associated with episodes of locoregional/ distant recurrences	Retrospective study	Journal article	USA	Truven Health MarketScan Commercial and Medicare Supplemental database	Single centre	January 2008 to 31 July 2017	NR	2017
**Triple negative breast cancer (TNBC) (2 studies)**										
Haiderali 2021 (80)	TNBC	To examine the real-world HCRU and costs among patients who received neoadjuvant treatment with or without adjuvant treatment in patients diagnosed with early-stage (II–IIIB) triple-negative breast cancer.	Retrospective, observational study	Journal article	USA	ConcertAI Oncology Dataset electronic medical record (EMR) data available to ConcertAI	Single centre	Mar 2008 to Mar 2016	Patient perspective	2018

Study name	Tumor types	Study objectives	Study design	Publication type	Country	Data source	Study setting	Time frame	Study perspective	Cost year
Başer 2012 (81)	TNBC	To examine the recurrence rate, health care utilization, and cost of early-stage TNBC in the US managed care setting.	Retrospective observational study	Journal article	USA	Oncology Management cancer registry	Single centre	1999 to 2009	Managed care perspective	NR

Abbreviations: AA: African American; AJCC: American joint committee on cancer; Ad T: adjuvant therapy; CP: carboplatin; CM: clinical modification; CCI: Charlson comorbidity index; CT: chemotherapy; ECOG: Eastern cooperative oncology group; ICD: international classification of disease; IF: Interferon alfa; RT: radiotherapy; RCT: radio chemotherapy; TURBT: transurethral resection of bladder tumour., USA: United States of America.

Appendix S8. Quality assessment methodology

Table 74. Quality assessment of cohort and cross-sectional studies

Study name	Bladder cancer	Gastric cancer	HNC	Melanoma	NSCLC	RCC	TNBC	Total score	Interpretation
Garg 2021 (22)	✓	-	-	-	-	-	-	******	Medium quality
Parsons 2020 (23)	✓	-	-	-	-	-	-	****	Medium quality
Rasmussen 2019 (1, 90)	✓	-	-	-	-	-	-	****	Medium quality
Ratanapornsompon 2019 (24)	✓	-	-	-	-	-	-	****	Medium quality
Lee 2019 (25)	✓	-	-	-	-	-	-	****	Medium quality
Simon 2019 (6)	✓	-	-	-	-	-	-	*****	Medium quality
Comperat 2015 (26)	✓	-	-	-	-	-	-	*****	Medium quality
Canter 2014 (2)	✓	-	-	-	-	-	-	*****	Medium quality
Chamie 2013 (27)	✓	-	-	-	-	-	-	****	Medium quality
Olsson 2013 (3)	✓	-	-	-	-	-	-	****	Medium quality

Study name	Bladder cancer	Gastric cancer	HNC	Melanoma	NSCLC	RCC	TNBC	Total score	Interpretation
Thomas 2013 (5)	✓	-	-	-	-	-	-	*****	Medium quality
Chaux 2012 (4)	✓	-	-	-	-	-	-	****	Medium quality
Yu 2021 (28)	✓	-	-	-	-	-	-	****	Medium quality
Bhat 2020 (89)	✓	-	-	-	-	-	-	****	Medium quality
Chu 2021 (88)	✓	-	-	-	-	-	-	***	Low quality
Sultana 2021 (29)	✓	-	-	-	-	-	-	***	Low quality
Kraja 2021 (33)	-	✓	-	-	-	-	-	****	Medium quality
Wilson 2021 (36)	-	-	✓	-	-	-	-	****	Medium quality
Park 2017 (35)	-	-	✓	-	-	-	-	****	Medium quality
Chang 2017 (85)	-	-	✓	-	-	-	-	*****	Medium quality
Brandstorp-Boesen 2016 (34)	-	-	✓	-	-	-	-	*****	Medium quality
Leoncini 2015 (84)	-	-	✓	-	-	-	-	*****	Medium quality
Kanatas 2014 (37)	-	-	✓	-	-	-	-		Medium quality

Study name	Bladder cancer	Gastric cancer	HNC	Melanoma	NSCLC	RCC	TNBC	Total score	Interpretation
Kim 2012 (39)	-	-	✓	-	-	-	-	******	Medium quality
Merja 2019 (83)	-	-	✓	-	-	-	-	****	Medium quality
Ogata 2021 (46)	-	-	-	✓	-	-	-	******	Medium quality
Ertekin 2021 (51)	-	-	-	✓	-	-	-	*******	High quality
Chen 2021 (44)	-	-	-	✓	-	-	-	*****	Medium quality
Kolla 2021 (7)	-	-	-	✓	-	-	-	****	Medium quality
Loidi 2021 (8)	-	-	-	✓	-	-	-	*****	Medium quality
Bleicher 2020 (40)	-	-	-	✓	-	-	-	*****	Medium quality
Sarac 2020 (9)	-	-	-	✓	-	-	-	******	Medium quality
Feigelson 2019 (10)	-	-	-	✓	-	-	-	****	Medium quality
Leclair 2019 (73)	-	-	-	✓	-	-	-	***	Low quality
Von Schuckman 2019 (11)	-	-	-	✓	-	-	-	*	Low quality
Leeneman 2019 (45)	-	-	-	✓	-	-	-	*****	Medium quality

Study name	Bladder cancer	Gastric cancer	HNC	Melanoma	NSCLC	RCC	TNBC	Total score	Interpretation
Chakera 2019 (41)	-	-	-	✓	-	-	-	****	Medium quality
Tas 2019 (49)	-	-	-	✓	-	-	-	****	Medium quality
Varey 2017 (47)	-	-	-	✓	-	-	-	*****	Medium quality
Tas 2017 (50)	-	-	-	✓	-	-	-	****	Medium quality
Rockberg 2016 (48)	-	-	-	✓	-	-	-	****	Medium quality
Osella-Abate 2015 (12)	-	-	-	✓	-	-	-	*****	Medium quality
Tarhini 2018 (43)	-	-	-	✓	-	-	-	*****	Medium quality
Leung 2022 (86)	-	-	-	✓	-	-	-	***	Low quality
Quhill 2021 (42)	-	-	-	✓	-	-	-	****	Medium quality
Jang 2020 (13)	-	-	-	✓	-	-	-	****	Medium quality
Burns 2019 (52)	-	-	-	✓	-	-	-	****	Medium quality
Martin 2022 (15)	-	-	-	-	✓	-	-	****	Medium quality
Slim 2021 (54)	-	-	-	-	✓	-	-	**	Low quality

Study name	Bladder cancer	Gastric cancer	HNC	Melanoma	NSCLC	RCC	TNBC	Total score	Interpretation
Karacz 2020 (14)	-	-	-	-	✓	-	-	****	Medium quality
Chouaid 2018 (53)	-	-	-	-	✓	-	-	****	Medium quality
Buck 2015 (55, 57)	-	-	-	-	✓	-	-	***	Low quality
Li 2019 (55)	-	-	-	-	✓	-	-	****	Medium quality
Kumar 2019 (56)	-	-	-	-	✓	-	-	****	Medium quality
Dabestani 2016 (58)	-	-	-	-	-	✓	-	*****	Medium quality
Thorstenson 2015 (59)	-	-	-	-	-	✓	-	****	Medium quality
Alvarado 2019 (60)	-	-	-	-	-	✓	-	****	Medium quality
Haiderali 2021a (16)	-	-	-	-	-	-	✓	*****	Medium quality
Haiderali 2021b (80)	-	-	-	-	-	-	✓	*****	Medium quality
Tecic 2020 (65)	-	-	-	-	-	-	✓	****	Medium quality
James 2019 (62)	-	-	-	-	-	-	✓	*****	Medium quality
Gonçalves 2018 (66)	-	-	-	-	-	-	✓	*****	Medium quality

Study name	Bladder cancer	Gastric cancer	HNC	Melanoma	NSCLC	RCC	TNBC	Total score	Interpretation
Ignatov 2018 (17)	-	-	-	-	-	-	✓	****	Medium quality
Gal 2018 (68)	-	-	-	-	-	-	✓	****	Medium quality
Kaplan 2017 (61)	-	-	-	-	-	-	✓	****	Medium quality
Suhani 2017 (67)	-	-	-	-	-	-	✓	****	Medium quality
Van 2016 (18)	-	-	-	-	-	-	✓	****	Medium quality
Matro 2015 (19)	-	-	-	-	-	-	✓	****	Medium quality
Steward 2014 (63)	-	-	-	-	-	-	✓	****	Medium quality
Eralp 2014 (20)	-	-	-	-	-	-	✓	*****	Medium quality
Metzger-Filho 2013 (69)	-	-	-	-	-	-	✓	****	Medium quality
Dawood 2012 (70)	-	-	-	-	-	-	✓	****	Medium quality
Başer 2012 (81)	-	-	-	-	-	-	✓	*****	Medium quality
Min Sun Bae 2015 (21)	-	-	-	-	-	-	✓	****	Medium quality
Villarreal 2021 (87)	-	-	-	-	-	-	✓	****	Medium quality

Abbreviations: HNC: head and neck cancer; NSCLC: non-small cell lung cancer; RCC: renal cell cancer; TNBC: triple-negative breast cancer

Table 75. Quality assessment of included studies – Larg-Moss quality assessment scale (cost of illness studies)

Study name	1.Analytical framework			2.Methodology and data					3.Analysis and reporting							
	Q1	Q2	Q3	Q1	Q2	Q3	Q4	Q5	Q1	Q2	Q3	Q4	Q5	Q6	Q7	Q8
Serra 2017 (79)	Assumed to be healthcare (Hospital) perspective	Yes	Not clear	(ii) only costs specific to (caused by) the health problem were included (confounders controlled)	No resource use was reported	No	Not clear	Not clear	Yes	No	Unclear	No	Yes	Yes	Yes	Yes

1: Q1: What was the motivation and perspective of the study?; Q2: Was the appropriate epidemiologic approach taken?; Q3: (a) Was an appropriate method(s) of quantification- used; 2: Q1: (a) Was an appropriate method(s) of quantification-used; Q2: (b) Was the resource quantification-method(s) well executed?; Q3: (c) Were healthcare resources valued appropriately?; Q4: d) Was the approach for valuing production losses justified, and assumptions valid?; Q5: (e) Was the inclusion of intangible- costs appropriate; 3: Q1: (a) Did the analysis address the study question?; Q2: (b) Was a range of estimates presented?; Q3: (c) Were the main uncertainties identified?; Q4: (d) Was a sensitivity analysis performed; Q5: (e) Was adequate documentation given; Q6: (f) Was uncertain- ty around the estimates and its implications adequately discussed?; Q7: (g) Were important limitations discussed?; Q8: (h) Were the results presented?

References

1. Rasmussen LA, Jensen H, Virgilsen LF, Falborg AZ, Møller H, Vedsted P. Time from incident primary cancer until recurrence or second primary cancer: risk factors and impact in general practice. European Journal of Cancer Care. 2019;28(5):e13123; doi:10.1111/ecc.13123.

2. Canter DJ, Revenig LM, Smith ZL, Dobbs RW, Malkowicz SB, Issa MM, et al. Re-examination of the natural history of high-grade T1 bladder cancer using a large contemporary cohort. International Brazilian Journal of Urology. 2014;40:172-8; doi:10.1590/S1677-5538.IBJU.2014.02.06.

3. Olsson H, Hultman P, Rosell J, Jahnson S. Population-based study on prognostic factors for recurrence and progression in primary stage T1 bladder tumours. Scandinavian Journal of Urology. 2013;47(3):188-95; doi:10.3109/00365599.2012.719539.

4. Chaux A, Karram S, Miller JS, Fajardo DA, Lee TK, Miyamoto H, et al. High-grade papillary urothelial carcinoma of the urinary tract: a clinicopathologic analysis of a post–World Health Organization/International Society of Urological Pathology classification cohort from a single academic center. Human Pathology. 2012;43(1):115-20; doi:10.1016/j.humpath.2011.04.013.

5. Thomas F, Noon AP, Rubin N, Goepel JR, Catto JW. Comparative outcomes of primary, recurrent, and progressive high-risk non–muscle-invasive bladder cancer. European Urology. 2013;63(1):145-54; doi:10.1016/j.euros.2022.02.011.

6. Simon M, Bosset P-O, Rouanne M, Benhamou S, Radulescu C, Molinié V, et al. Multiple recurrences and risk of disease progression in patients with primary low-grade (TaG1) non–muscle-invasive bladder cancer and with low and intermediate EORTC-risk score. PloS One. 2019;14(2):e0211721; doi:10.1371/journal.pone.0211721.

7. Kolla AM, Vitiello GA, Friedman EB, Sun J, Potdar A, Daou H, et al. Acral lentiginous melanoma: a United States multi-center substage survival analysis. Cancer Control. 2021;28:10732748211053567; doi:10.1177/10732748211053567.

8. Loidi-Pascual L, Lecumberri-Biurrun MJ, Arozarena-Martinicorena I, Goñi-Gironés E, Yanguas-Bayona JI. Study of cutaneous melanoma recurrences after sentinel node biopsy: Patterns of dissemination and use of complementary test in follow-up. European Journal of Cancer Care. 2021;30(1):e13344; doi:10.1111/ecc.13344.

9. Sarac E, Wilhelmi J, Thomas I, Leiter U, Keim U, Eigentler T, et al. Late recurrence of melanoma after 10 years–Is the course of the disease different from early recurrences? Journal of the European Academy of Dermatology and Venereology 2020;34(5):977-83; doi:10.1111/jdv.16106.

10. Feigelson HS, Powers JD, Kumar M, Carroll NM, Pathy A, Ritzwoller DP. Melanoma incidence, recurrence, and mortality in an integrated healthcare system: A retrospective cohort study. Cancer Medicine. 2019;8(9):4508-16; doi:10.1002/cam4.2252.

11. Von Schuckmann LA, Hughes MCB, Ghiasvand R, Malt M, Van Der Pols JC, Beesley VL, et al. Risk of melanoma recurrence after diagnosis of a high-risk primary tumor. JAMA Dermatology. 2019;155(6):688-93; doi:10.1001/jamadermatol.2019.0440.

12. Osella-Abate S, Ribero S, Sanlorenzo M, Maule MM, Richiardi L, Merletti F, et al. Risk factors related to late metastases in 1,372 melanoma patients disease free more than 10 years. International Journal of Cancer. 2015;136(10):2453-7; doi:10.1002/ijc.29281.

13. Jang S, Poretta T, Bhagnani T, Deering K, Burke M, Rao S. Real-world (RW) recurrence rates and economic impact in patients with resected early-stage melanoma. Pigment Cell and Melanoma Research. 2020;33(1):219; doi:10.1111/pcmr.12834.

14. Karacz CM, Yan J, Zhu H, Gerber DE. Timing, sites, and correlates of lung cancer recurrence. Clinical Lung Cancer. 2020;21(2):127-35. e3; doi:10.1016/j.cllc.2019.12.001.

15. Martin CM, Puello-Guerrero A, Mas-Lopez LA, Campos-Gómez S, Orlando-Orlandi FJ, Tejado Gallegos LF, et al. Real-world KINDLE-Latin America subset data on treatment patterns and clinical outcomes in patients with stage III non-small-cell lung cancer. Cancer Medicine. 2023;12(2):1247-59; doi:10.1002/cam4.4990.

16. Haiderali A, Rhodes WC, Gautam S, Huang M, Sieluk J, Skinner KE, et al. Real-world treatment patterns and effectiveness outcomes in patients with early-stage triple-negative breast cancer. Future Oncology. 2021;17(29):3819-31; doi:10.2217/fon-2021-0530.

17. Ignatov A, Eggemann H, Burger E, Ignatov T. Patterns of breast cancer relapse in accordance to biological subtype. Journal of Cancer Research Clinical Oncology. 2018;144(7):1347-55; doi:10.1007/s00432-018-2644-2.

18. Van Roozendaal LM, Smit LH, Duijsens GH, de Vries B, Siesling S, Lobbes MB, et al. Risk of regional recurrence in triple-negative breast cancer patients: a Dutch cohort study. Breast Cancer Research Treatment. 2016;156:465-72; doi:10.1007/s10549-016-3757-4.

19. Matro JM, Li T, Cristofanilli M, Hughes ME, Ottesen RA, Weeks JC, et al. Inflammatory breast cancer management in the national comprehensive cancer network: the disease, recurrence pattern, and outcome. Clinical Breast Cancer. 2015;15(1):1-7; doi:10.1016/j.clbc.2014.05.005.

20. Eralp Y, Kılıç L, Alço G, Başaran G, Doğan M, Dinçol D, et al. The outcome of patients with triple negative breast cancer: The Turkish Oncology Group Experience. The Journal of Breast Health. 2014;10(4):209-15; doi:10.5152/tjbh.2014.1904.

21. Bae MS, Moon H-G, Han W, Noh D-Y, Ryu HS, Park I-A, et al. Early stage triple-negative breast cancer: imaging and clinical-pathologic factors associated with recurrence. Radiology. 2016;278(2):356-64; doi:10.1148/radiol.2015150089.

22. Garg T, McMullen CK, Leo MC, O'Keeffe-Rosetti MC, Weinmann S, Nielsen ME. Predicting risk of multiple levels of recurrence and progression after initial diagnosis of nonmuscle-invasive bladder cancer in a multisite, community-based cohort. Cancer. 2021;127(4):520-7; doi:10.1002/cncr.33300.

23. Parsons S, Hill G, Warren K, Burden H. Bladder cancer in the elderly: A retrospective analysis of bladder cancer in individuals older than 85 years. Journal of Clinical Urology. 2020;13(2):110-5; doi:10.1177/20514158198517.

24. Ratanapornsompong W, Kongcharoensombat W, Sangkum P, Pacharatakul S. Progression and Prognostic Factors of T1 High-Grade Non-Muscle Invasive Bladder Cancer, 10-Year Follow-Up at Ramathibodi Hospital. Journal of the Medical Association of Thailand. 2019;102(5).

25. Lee A, Lee HJ, Huang HH, Ho H, Chen K. Low-risk non-muscle-invasive bladder cancer: Further prognostic stratification into the "very-low-risk" group based on tumor size. International Journal of Urology. 2019;26(4):481-6; doi:10.1111/iju.13913.

26. Compérat E, Larré S, Roupret M, Neuzillet Y, Pignot G, Quintens H, et al. Clinicopathological characteristics of urothelial bladder cancer in patients less than 40 years old. Virchows Archiv. 2015;466:589-94; doi:10.1007/s00428-015-1739-2.

27. Chamie K, Litwin MS, Bassett JC, Daskivich TJ, Lai J, Hanley JM, et al. Recurrence of high-risk bladder cancer: a population-based analysis. Cancer. 2013;119(17):3219-27; doi:10.1002/cncr.28147.

28. Yu YD, Ko YH, Kim JW, Jung SI, Kang SH, Park J, et al. The prognosis and oncological predictor of urachal carcinoma of the bladder: a large scale multicenter cohort study analyzed 203 patients with long term follow-up. Frontiers in Oncology. 2021;11:683190; doi:10.3389/fonc.2021.683190.

29. Sultana A, Patel D, Ehsanullah S, Shahzad S. Natural course of low-risk non-muscle-invasive bladder cancers (NIMBC): A 10 year follow-up and comparative study to European Association of Urology (EAU). European Urology. 2021;31:S4.

30. Ratanapornsompong W, Kongcharoensombat W, Sangkum P, Pacharatakul SJJotMAoT. Progression and Prognostic Factors of T1 High-Grade Non-Muscle Invasive Bladder Cancer, 10-Year Follow-Up at Ramathibodi Hospital. 2019;102(5).

31. Canter DJ, Revenig LM, Smith ZL, Dobbs RW, Malkowicz SB, Issa MM, et al. Re-examination of the natural history of high-grade T1 bladder cancer using a large contemporary cohort. 2014;40:172-8.

32. Olsson H, Hultman P, Rosell J, Jahnson SJSJoU. Population-based study on prognostic factors for recurrence and progression in primary stage T1 bladder tumours. 2013;47(3):188-95.

33. Kraja F, Dervishi J, Hoti A, Karaulli E, Akshija I, Hafizi E, et al. Prognostic factors affecting survival in gastric cancer patients in Albania: A retrospective study. Radiotherapy and Oncology. 2021;161:S1011-S2; doi:10.1016/S0167-8140(21)07674-X.

34. Brandstorp-Boesen J, Sørum Falk R, Folkvard Evensen J, Boysen M, Brøndbo K. Risk of recurrence in laryngeal cancer. PLoS One. 2016;11(10):e0164068; doi:10.1371/journal.pone.0164068.

35. Park G, Roh JL, Cho KJ, Jin M, Choi SH, Nam S, et al. Incidence and risk factors of late recurrence in patients with salivary gland cancer. Clinical Otolaryngology. 2017;42(2):416-24; doi:10.1111/coa.12808.

36. Wilson JM, Lumley C, Tan X, Shen C, Coniglio A, Weissler M, et al. Clinical outcomes of patients with pT1-T2N0 oral tongue squamous cell carcinoma. American Journal of Clinical Oncology. 2021;44(5):200-5; doi:10.1097/COC.0000000000000806.

37. Kanatas A, Bala N, Lowe D, Rogers SN. Outpatient follow-up appointments for patients having curative treatment for cancer of the head and neck: are the current arrangements in need of change? British Journal of Oral Maxillofacial Surgery. 2014;52(8):681-7; doi:10.1016/j.bjoms.2014.06.017.

38. Jung YH, Song CM, Park JH, Kim H, Cha W, Hah JH, et al. Efficacy of current regular follow-up policy after treatment for head and neck cancer: need for individualized and obligatory follow-up strategy. Head Neck. 2014;36(5):715-21; doi:10.1002/hed.23364.

39. Kim Le T, Winfree KB, Yang H, Marynchenko M, Yu AP, Frois C, et al. Treatment patterns and economic burden of metastatic and recurrent locally-advanced head and neck cancer patients. Journal of Medical Economics. 2012;15(4):786-95; doi:10.3111/13696998.2012.682632.

40. Bleicher J, Swords DS, Mali ME, McGuire L, Pahlkotter MK, Asare EA, et al. Recurrence patterns in patients with Stage II melanoma: The evolving role of routine imaging for surveillance. Journal of Surgical Oncology. 2020;122(8):1770-7; doi:10.1002/jso.26214.

41. Chakera AH, Quinn MJ, Lo S, Drummond M, Haydu LE, Bond JS, et al. Subungual melanoma of the hand. Annals of Surgical Oncology. 2019;26(4):1035-43; doi:10.1245/s10434-018-07094-w.

42. Quhill H, Jefferis JM, Rennie IG, Salvi S, Gavin AT, Rundle P. Uveal melanoma in Northern Ireland. Ophthalmologica. 2021;244(SUPPL 1).

43. Tarhini A, Ghate SR, Ionescu-Ittu R, Manceur AM, Ndife B, Jacques P, et al. Postsurgical treatment landscape and economic burden of locoregional and distant recurrence in patients with operable nonmetastatic melanoma. Melanoma Research. 2018;28(6):618; doi:10.1097/CMR.0000000000000507.

44. Chen P-Y, Liao Y-L, Chu Y-C, Tsai Y-J. Conjunctival melanoma: A 20-year survey in a comprehensive medical center. Journal of the Formosan Medical Association. 2021;120 (1 Pt 1)(1):250-5; doi:10.1016/j.jfma.2020.04.032.

45. Leeneman B, Franken MG, Coupé VM, Hendriks MP, Kruit W, Plaisier PW, et al. Stage-specific disease recurrence and survival in localized and regionally advanced cutaneous melanoma. European Journal of Surgical Oncology. 2019;45(5):825-31; doi:10.1001/jamadermatol.2023.3256.

46. Ogata D, Tanese K, Nakamura Y, Otsuka M, Namikawa K, Funakoshi T, et al. Impact of the changes in the completion lymph node dissection criteria and approval of adjuvant therapies on the real-world outcomes of Japanese stage III melanoma patients. International Journal of Clinical Oncology. 2021;26:2338-46; doi:10.1007/s10147-021-02029-0.

47. Varey AH, Goumas C, Hong AM, Mann GJ, Fogarty GB, Stretch JR, et al. Neurotropic melanoma: an analysis of the clinicopathological features, management strategies and survival outcomes for 671 patients treated at a tertiary referral center. Modern Pathology. 2017;30(11):1538-50; doi:10.1038/modpathol.2017.76.

48. Rockberg J, Amelio JM, Taylor A, Jörgensen L, Ragnhammar P, Hansson J. Epidemiology of cutaneous melanoma in Sweden—stage-specific survival and rate of recurrence. International Journal of Cancer. 2016;139(12):2722-9; doi:10.1002/ijc.30407.

49. Tas F, Erturk K. Relapse patterns in patients with local and regional cutaneous melanoma. Clinical Translational Oncology. 2019;21:412-9; doi:10.1007/s12094-018-1938-9.

50. Tas F, Erturk K. Recurrence behavior in early-stage cutaneous melanoma: pattern, timing, survival, and influencing factors. Melanoma Research. 2017;27(2):134-9; doi:10.1097/CMR.0000000000000332.

51. Ertekin SS, Podlipnik S, Riquelme-Mc Loughlin C, Barreiro-Capurro A, Arance A, Carrera C, et al. Initial stage of cutaneous primary melanoma plays a key role in the pattern and timing of disease recurrence. Acta Dermato-Venereologica. 2021;101(7); doi:10.2340/00015555-3832.

52. Burns W, Durham A, Dossett L, Hughes T, Chang A, Bichakjian C, et al. Clinical Outcomes of Melanoma Patients with Low Sentinel Lymph Node Tumor Burden. Annals of Surgical Oncology. 2019;26(Suppl 1):S44-S; doi:10.1245/s10434-019-07174-5.

53. Chouaid C, Danson S, Andreas S, Siakpere O, Benjamin L, Ehness R, et al. Adjuvant treatment patterns and outcomes in patients with stage IB-IIIA non-small cell lung cancer in France, Germany, and the United Kingdom based on the LuCaBIS burden of illness study. Lung Cancer. 2018;124:310-6; doi:10.1016/j.lungcan.2018.07.042.

54. Slim A, Kamoun H, Hadidene Y, Smadhi H, Meddeb A, Megdiche ML. Postoperative recurrence of primary lung cancer: anatomo-clinical and therapeutic study. La Tunisie Medicale. 2021;99(5):560.

55. Li X, Cheng M, Yang J, Wu Y, Zhang X, Hou J. P2. 04-38 Tumor-Associated Neutrophils as a Potential Predictor for Early Recurrence in Resectable I-IIIA Lung Adenocarcinoma. Journal of Thoracic Oncology. 2019;14(10):S723; doi:10.1016/j.jtho.2019.08.1543.

56. Kumar A, Kan C, Traughber B. Outcomes and Patterns of Failure for Stage I Non-Small Cell Lung Cancer in a Veteran Population. International Journal of Radiation Oncology Biology Physics. 2019;103(5):E38-E9; doi:10.1016/S0360-3016(19)30498-5.

57. Buck PO, Saverno KR, Miller PJ, Arondekar B, Walker MS. Treatment patterns and health resource utilization among patients diagnosed with early stage resected non–small cell lung cancer at US community oncology practices. Clinical Lung Cancer. 2015;16(6):486-95; doi:10.1016/j.cllc.2014.12.010.

58. Dabestani S, Thorstenson A, Lindblad P, Harmenberg U, Ljungberg B, Lundstam S. Renal cell carcinoma recurrences and metastases in primary non-metastatic patients: a population-based study. World Journal of Urology. 2016;34:1081-6; doi:10.1007/s00345-016-1773-y.

59. Thorstenson A, Harmenberg U, Lindblad P, Holmström B, Lundstam S, Ljungberg B. Cancer characteristics and current treatments of patients with renal cell carcinoma in Sweden. BioMed Research International. 2015;2015; doi:10.1155/2015/456040.

60. Alvarado-Cabrero I, Ramirez-Gonzalez D, Estevez-Castro R, Elena Martin-Aguilar A, Valencia-Cedillo R. Aggressive Tubulocystic Carcinomas of the Kidney: A Clinical, Pathological and Immunohistochemical Study. Modern Pathology. 2019;32(3).

61. Kaplan HG, Malmgren JA, Atwood MK. Triple-negative breast cancer in the elderly: Prognosis and treatment. The Breast Journal. 2017;23(6):630-7; doi:10.1111/tbj.12813.

62. James M, Dixit A, Robinson B, Frampton C, Davey V. Outcomes for patients with non-metastatic triple-negative breast cancer in New Zealand. Clinical Oncology. 2019;31(1):17-24; doi:10.1016/j.clon.2018.09.006.

63. Steward L, Conant L, Gao F, Margenthaler JA. Predictive factors and patterns of recurrence in patients with triple negative breast cancer. Annals of Surgical Oncology. 2014;21:2165-71; doi:10.1245/s10434-014-3546-4.

64. Dawood S, Lei X, Litton JK, Buchholz TA, Hortobagyi GN, Gonzalez-Angulo AM. Incidence of brain metastases as a first site of recurrence among women with triple receptor–negative breast cancer. Cancer. 2012;118(19):4652-9; doi:10.1002/cncr.27434.

65. Tečić Vuger A, Šeparović R, Vazdar L, Pavlović M, Lepetić P, Šitić S, et al. Characteristics and prognosis of triple-negative breast cancer patients: a Croatian single institution retrospective cohort study. Acta Clinica Croatica. 2020;59(1):97-107; doi:10.20471/acc.2020.59.01.12.

66. Gonçalves Jr H, Guerra MR, Duarte Cintra JR, Fayer VA, Brum IV, Bustamante Teixeira MT. Survival study of triple-negative and non–triple-negative breast cancer in a Brazilian cohort. Clinical Medicine Insights: Oncology. 2018;12:1179554918790563; doi:10.1177/1179554918790563.

67. Parshad R, Kazi M, Seenu V, Mathur S, Dattagupta S, Haresh K. Triple-negative breast cancers: are they always different from nontriple-negative breast cancers? An experience from a tertiary center in India. Indian Journal of Cancer. 2017;54(4):658-63; doi:10.4103/ijc.IJC_348_17.

68. Gal O, Ishai Y, Sulkes A, Shochat T, Yerushalmi R. Early breast cancer in the elderly: characteristics, therapy, and long-term outcome. Oncology. 2018;94(1):31-8; doi:10.1159/000480087.

69. Metzger-Filho O, Sun Z, Viale G, Price KN, Crivellari D, Snyder RD, et al. Patterns of recurrence and outcome according to breast cancer subtypes in lymph node–negative disease: results from International Breast Cancer Study Group Trials VIII and IX. Journal of Clinical Oncology. 2013;31(25):3083; doi:10.1200/JCO.2012.46.1574.

70. Dawood S, Lei X, Litton JK, Buchholz TA, Hortobagyi GN, Gonzalez-Angulo AM. Impact of body mass index on survival outcome among women with early stage triple-negative breast cancer. Clinical Breast Cancer. 2012;12(5):364-72; doi:10.1016/j.clbc.2012.07.013.

71. Garg T, McMullen CK, Leo MC, O'Keeffe-Rosetti MC, Weinmann S, Nielsen MEJC. Predicting risk of multiple levels of recurrence and progression after initial diagnosis of nonmuscle-invasive bladder cancer in a multisite, community-based cohort. 2021;127(4):520-7.

72. Lee A, Lee HJ, Huang HH, Ho H, Chen KJIJoU. Low-risk non-muscle-invasive bladder cancer: Further prognostic stratification into the "very-low-risk" group based on tumor size. 2019;26(4):481-6.

73. Séguin Leclair C, Lebel S, Westmaas JL. The relationship between fear of cancer recurrence and health behaviors: A nationwide longitudinal study of cancer survivors. Health Psychology. 2019;38(7):596; doi:10.1037/hea0000754.

74. Smith AB, McCabe S, Deal AM, Guo A, Gessner KH, Lipman R, et al. Quality of life and health state utilities in bladder cancer. Bladder Cancer. 2022;8(1):55-70; doi:10.3233/BLC-211615.

75. Shin J, Shin DW, Lee J, Hwang J, Lee JE, Cho B, et al. Exploring socio-demographic, physical, psychological, and quality of life-related factors related with fear of cancer recurrence in stomach cancer survivors: a cross-sectional study. BMC Cancer. 2022;22(1):1-10; doi:10.1186/s12885-022-09507-2.

76. van de Wal M, van de Poll-Franse L, Prins J, Gielissen M. Does fear of cancer recurrence differ between cancer types? A study from the population-based PROFILES registry. Psycho-Oncology. 2016;25(7):772-8; doi:10.1002/pon.4002.

77. Atkinson TM, Noce NS, Hay J, Rafferty BT, Brady MS. Illness-related distress in women with clinically localized cutaneous melanoma. Annals of Surgical Oncology. 2013;20:675-9; doi:10.1245/s10434-012-2635-5.

78. Leeneman B, Blommestein HM, Coupé VM, Hendriks MP, Kruit WH, Plaisier PW, et al. Real-world healthcare costs of localized and regionally advanced cutaneous melanoma in the Netherlands. Melanoma Research. 2021;31(3):249-57; doi:10.1097/CMR.0000000000000732.

79. Serra-Arbeloa P, Rabines-Juárez Á, Álvarez-Ruiz M, Guillén-Grima F. Cost of cutaneous melanoma by tumor stage: a descriptive analysis. Actas Dermosifiliográficas. 2017;108(3):229-36; doi:10.1016/j.ad.2016.09.010.

80. Haiderali A, Rhodes WC, Gautam S, Huang M, Sieluk J, Skinner KE, et al. Healthcare resource utilization and cost among patients treated for early-stage triple-negative breast cancer. Future Oncology. 2021;17(29):3833-41; doi:10.2217/fon-2021-0531.

81. Başer O, Wei W, Henk HJ, Teitelbaum A, Xie L. Burden of early-stage triple-negative breast cancer in a US managed care plan. Health Outcomes Research in Medicine. 2012;3(2):e57-e65; doi:10.1016/j.ehrm.2012.03.001.

82. Lee Y, Chen K, Ho H, Huang H, Lee H. Further stratification of low-risk nonmuscle invasive bladder cancer into" very-low-risk" group based on initial tumour size. BJU International. 2019;123:4-.

83. Merja M, Chaudhary M, Kasbekar P, Pandya S. Comparing the outcomes of organ preserving modalities with surgery in locally advanced laryngeal cancers: A retrospective institutional study. European Journal of Surgical Oncology. 2019;45(2):e123-e4.

84. Leoncini E, Vukovic V, Cadoni G, Pastorino R, Arzani D, Bosetti C, et al. Clinical features and prognostic factors in patients with head and neck cancer: Results from a multicentric study. Cancer Epidemiology. 2015;39(3):367-74; doi:10.1016/j.canep.2015.02.004.

85. Chang J-H, Wu C-C, Yuan KS-P, Wu AT, Wu S-Y. Locoregionally recurrent head and neck squamous cell carcinoma: incidence, survival, prognostic factors, and treatment outcomes. Oncotarget. 2017;8(33):55600; doi:10.18632/oncotarget.16340.

86. Leung B, Nguyen N, Choi M, DeSimone M, Wan G, Zhang S, et al. 658 Clinical and histopathologic risk factors for early-stage melanoma recurrence. Journal of Investigative Dermatology. 2022;142(8):S113; doi:10.1016/j.jid.2022.05.669.

87. Villarreal-Garza C, Becerril Gaitan A, Vaca-Cartagena B, Mesa-Chavez F, Ferrigno AS, Platas A, et al. High-early recurrence rate in a cohort of young women with breast cancer in Mexico. Journal of Clinical Oncology. 2021;39:e12560-e; doi:10.1200/JCO.2021.39.15_suppl.e12560.

88. Chu C, Li K, Meng M, Porten S. PD63-12 Use of Cxbladder monitor during the COVID-19 pandemic to reduce the frequency of surveillance cystoscopy. Journal of Urology. 2021;206(Supplement 3):e1142; doi:10.1097/JU.0000000000002107.12.

89. Bhat A, Kwon D, Soodana-Prakash N, Banerjee I, Bhattu A, Atluri V, et al. MP24-09 Surveillance intensity and detection of recurrence and progression in intermediate risk non muscle invasive bladder cancer (NMIBC). The Journal of Urology. 2020;203(Supplement 4):e350-e1; doi:10.1097/JU.0000000000000857.09.

90. Rasmussen LA, Jensen H, Virgilsen LF, Falborg AZ, Møller H, Vedsted P. Healthcare utilisation in general practice and hospitals in the year preceding a diagnosis of cancer recurrence or second primary cancer: a population-based register study. BMC Health Services Research. 2019;19:1-11; doi:10.1186/s12913-019-4757-y.
